# Integrated analysis of miRNA/mRNA network in placenta identifies key factors associated with labor onset of Large White and Qingping sows

**DOI:** 10.1038/srep13074

**Published:** 2015-08-14

**Authors:** Huanan Li, Bin Wu, Junnan Geng, Jiawei Zhou, Rong Zheng, Jin Chai, Fenge Li, Jian Peng, Siwen Jiang

**Affiliations:** 1 Key Laboratory of Swine Genetics and Breeding of Agricultural Ministry and Key Laboratory of Agricultural Animal Genetics, Breeding and Reproduction of Ministry of Education, College of Animal Science and Technology, Huazhong Agricultural University, Wuhan 430070, People’s Republic of China; 2 Department of Animal Nutrition and Feed Science, College of Animal Science and Technology, Huazhong Agricultural University, Wuhan 430070, People’s Republic of China; 3 The Cooperative Innovation Center for Sustainable Pig Production, Wuhan 430070, China People’s Republic of China

## Abstract

Labour onset is a very complex physiological process, and its mechanism is poorly understood. Here, we obtained the mRNA and miRNA expression profiles from the placentas of four groups of sows: Qingping sows 112 days after insemination with signs of labour onset (QS), Qingping sows 114 days after insemination with signs of labour onset (QL), Large White sows 114 days after insemination with signs of labour onset (LL) and Large White sows 112 days after insemination without signs of labour onset (LN). A set of differentially expressed genes, including 2164 mRNAs and 39 miRNAs, were found. A DAVID analysis of these differentially expressed genes revealed their critical roles in response to hormone stimulus, immune response. Cytoscape Network analysis of the functional genes found node mRNAs and that the regulatory network between the node mRNAs and miRNAs was established. A comparison of the sequencing data from the shorter gestation period (QS) and the normal gestation period (QL) indicated that these genes were responsible for the quicker and more sensitive reaction to the regulation of labour onset. This research not only detected the key factors that were involved in labour onset but also provided useful information for the research of gynaecological diseases.

Delivery indicates the end of the gestation period. Within a species, the duration of pregnancy always remains within a definite narrow range^1^, but the mechanism of labour onset and regulation remains obscure. Many factors are involved in the onset of labour, such as various hormones and inflammatory mediators during labour, which remain a dominant research topic in this field^2^,^3^,^4^,^5^,^6^. Progesterone is one of the most important hormones for sow pregnancy maintenance^7^, and the theory of Progesterone Withdrawal is the most popular yet proposed. The increase in prostaglandin (PG) reduces progesterone before term, followed by the initiation of labour^8^. Inflammatory cytokines and chemokines in the placenta have been extensively investigated in the context of normal and abnormal labours. These inflammatory cytokines regulate the release of uterotonics, such as prostaglandins^9^. It is urgent both to identify sensitive and specific genes and miRNAs that are associated with labour and to investigate the mechanism underlying labour.

As a compound tissue, the placenta, which consists of amnion, chorion and decidua, performs the functions of exchange for oxygen, nutrients, antibodies, hormones, and waste products between the mother and foetus and may carry valuable information about pregnancy^10^. Pigs have a non-invasive placenta that is classified as diffuse, and almost the entire surface of the allantochorion is involved in the formation of the placenta.

MicroRNAs (miRNAs) are a family of approximately 22 nucleotide small RNA molecules that play important roles in regulating post-transcriptional translation by binding to the target mRNAs’ 3′-untranslated regions^11^. miRNAs are involved in the maintenance of the myometrial quiescence and the timing of labour among hormonal, inflammatory and physical factors. Recent evidence suggests that pregnancy and labour in the myometrium, cervix and foetal membranes are influenced by miRNAs, including miR-200^12^,^13^, miR-199a-3p, miR-214^14^, miR-223, miR-34b, miR-34c^15^, miR-338^16^ and miR-223^17^. These studies indicate that miRNAs play key collaborative roles in the hormonal control of myometrial quiescence and contractility during pregnancy and labour. A comprehensive network of miRNA/mRNA expression profiles, which compares the quiescent pregnant mouse myometrium with the contractile one, has been established^12^. However, a comprehensive miRNA/mRNA regulatory network associated with pregnancy and labour in the placenta has not been constructed due to a lack of simultaneously profiled miRNAs and mRNAs across different pig breeds at labour. Pigs are closer to people in size, anatomy and physiology; therefore, pigs are considered an ideal model for human-related diseases. An increasing number of miRNAs have been identified from swine tissues, such as muscle and adipose tissue^18^,^19^,^20^,^21^, but fewer have been identified from the porcine placenta. Qingping pig is a Chinese local pig breed with black fur and has a shorter gestation length, but the mechanism of this trait remains to be elucidated.

In the present study, we performed mRNA and miRNA profiling using Qingping and Large White placentas before and at labour onset and then constructed an interaction network of functional genes and an miRNA/mRNA regulatory network. The Database for the Annotation, Visualisation, and Integrated Discovery (DAVID) analysis of differentially expressed genes (DEGs) among LL, QL, QS and LN revealed a set of genes that are related to internal secretion, the extracellular matrix, the inflammatory response, the immune response and cellular apoptosis. The functional interaction network analysis of the DEGs indicated that FOS, APOE, MMP1, SMAD3, HSPA8, CXCR4 and PARK2 were the key node genes; then, the regulatory network between the node mRNAs and miRNAs was established. Moreover, a DAVID analysis of miRNA target DEG analysis highlighted the critical roles of five overlapping miRNA signatures (ssc-miR-132, ssc-miR-323, ssc-miR-19a, ssc-miR-664-3p and ssc-miR-296-5p) in the extracellular matrix, response to hormone stimulus, positive regulation of cell adhesion, focal adhesion, positive regulation of immune system process, and arachidonic acid metabolism. Additionally, a comparison of the sequencing data from Qingping pigs with a shorter gestation period of 112 days (QS) and a normal gestation period of 114 days (QL) revealed that the genes that are associated with immunity, signal peptide, and cell adhesion were up-regulated in QS pigs, which resulted in a quicker and more sensitive regulation of labour onset. These results have expanded the number of porcine miRNAs and suggest that pigs can be used as a model for human childbirth initiation to establish the biological basis for studying the role of miRNAs in regulating labour onset.

## Results

### mRNA and miRNA expression profiles in the placenta

**Figure 1:**
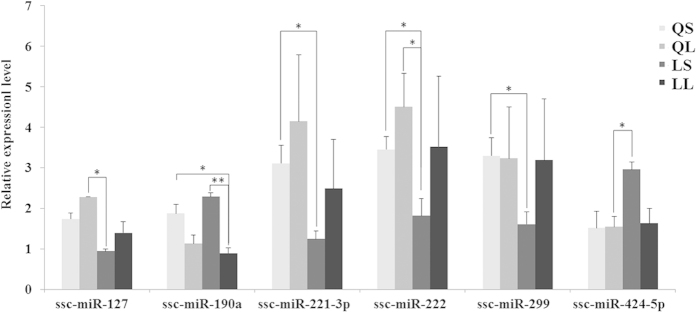
Quantitative RT-PCR validation of the differential expression of miRNAs. The expression levels of ssc-miR-127, ssc-miR-190a, ssc-miR-221-3p, ssc-miR-222, ssc-miR-299 and ssc-miR-424-5p in LN, LL, QS and QL were detected by qRT-PCR. All of the error bars indicate SD. *P < 0.05, **P < 0.01.

To examine the genes that are related to the onset of labour, we determined the gene expression profiles in the placentas of LL, QL, QS and LN using Illumina HiSeq 2000. Compared with those in LN, among the 930 differentially expressed genes (DEGs) in LL (*P *≤ 0.01, fold change ≤ 0.5 or fold change ≥ 2), 395 genes were up-regulated and 535 were down-regulated (Supplementary Table S1); among the 1591 DEGs in QL (*P *≤ 0.01; fold change ≤ 0.5 or fold change ≥ 2), 690 and 901 genes were up- and down-regulated, respectively (Supplementary Table S2); and among the 1074 DEGs in QS (*P *≤ 0.01; fold change ≤ 0.5 or fold change ≥ 2), 503 genes were up-regulated and 571 were down-regulated (Supplementary Table S3). Compared with the LN placenta, 9 up- and 10 down-regulated miRNAs were found in the LL placenta (*P *≤ 0.01; fold change ≤ 0.5 or fold change ≥ 2), 13 up- and 10 down-regulated miRNAs were found in the QL placenta(*P *≤ 0.01; fold change ≤ 0.5 or fold change ≥ 2), and 14 up- and 7 down-regulated miRNAs were found in the QS placenta (*P *≤ 0.01; fold change ≤ 0.5 or fold change ≥ 2) (Supplementary Table S4). The unified set of differentially expressed genes containing a total of 2164 mRNAs and 36 miRNAs was found by the comparison of LL, QL and QS with LN. Remarkably, 182 genes were overlapping among the LL, QL and QS, 76 of which were up-regulated and 106 were down-regulated. In addition, 2 (ssc-miR-132, ssc-miR-323) and 3 (ssc-miR-19a, ssc-miR-664-3p and ssc-miR-296-5p) miRNAs were commonly detected in all of the regulated groups across the comparisons of LL, QL, and QS with LN. To validate the reliability of the mRNA and miRNA profiling results, RT-PCR was performed to detect the levels of 13 randomly selected differential mRNAs and 7 miRNAs from the differential expression data using the primer sequences listed in Supplementary Table S5. The RT-PCR results showed that most of the miRNAs were consistent with the miRNAs profiling results, except for miR-22 (Fig. 1). The RT-PCR results also showed that mRNAs agreed most with the mRNA profiling results (Table 1).

**Figure 2:**
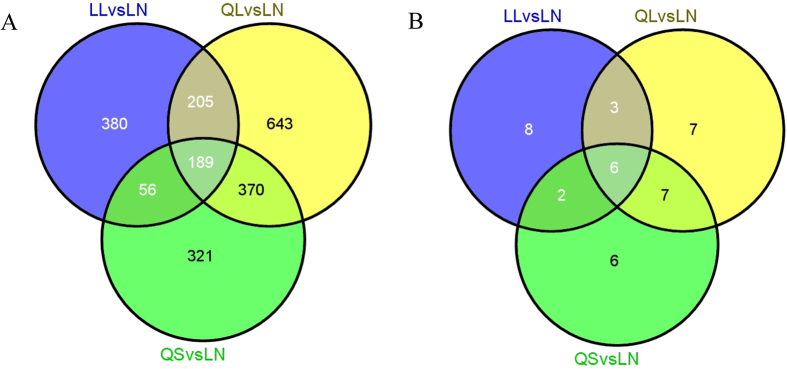
Venn diagram of LL, QS, QL compared with LN diff-genes, indicating the numbers of genes in the different or overlapping groups. (**A**) mRNA, (**B**) miRNA.

The Venn diagrams of the differentially expressed genes of the subgroups are presented in Fig. 2A (mRNAs) and 2B (miRNAs). Specifically, overlapping genes among the three groups accounted for 54%, 54% and 66% of the LL, QL, and QS mRNA diff-genes, respectively, which suggests the internal relationships of the subgroups in the number of genes within the unified diff-gene set. Additionally, 58%, 70% and 68% of LL, QL and QS miRNA diff-genes were overlapping with one another.

**Figure 3:**
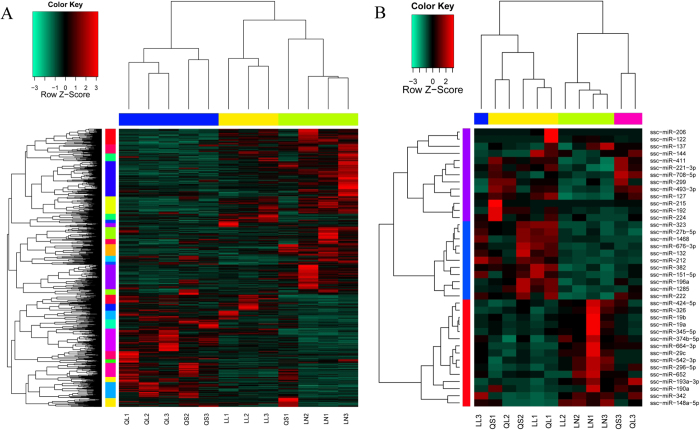
The mRNA and miRNA expression patterns of a unified set of differentially expressed genes and miRNAs. (**A**) Hierarchical clustering of a unified set of differentially expressed genes. (**B**) Hierarchical clustering of a unified set of differentially expressed miRNAs.

The hierarchical clustering results revealed that the subgroups could be well differentiated from our unified set of differentially expressed mRNAs, except for QS1 (Fig. 3A), which suggests significant differences among subgroups and the good generality of our diff-genes, though they were identified from relatively small datasets. miRNA diff-genes performed less accurately than did mRNA diff-genes (Fig. 3B). Because the miRNA and mRNA data shared the same sample set, this difference was likely due to the complexity of the conversion from miRNA expression to the phenotypic differences among different pig subgroups, which is mediated and influenced by many factors, such as posttranscriptional regulation and generally a large number of varieties of miRNA gene targets. Additionally, the difference could also be attributed to the effects of the small sample size.

### Functional analysis of differentially expressed genes

**Figure 4:**
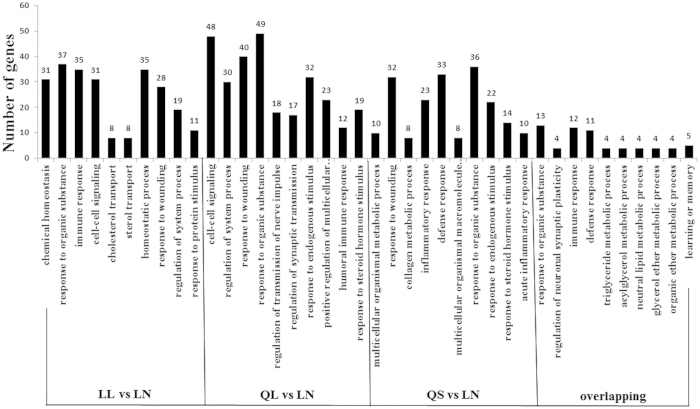
The Top 10 GO (biological process) Term analyses of DEGs of LL, QL, and QS, compared with LN placenta and overlapping.

**Figure 5:**
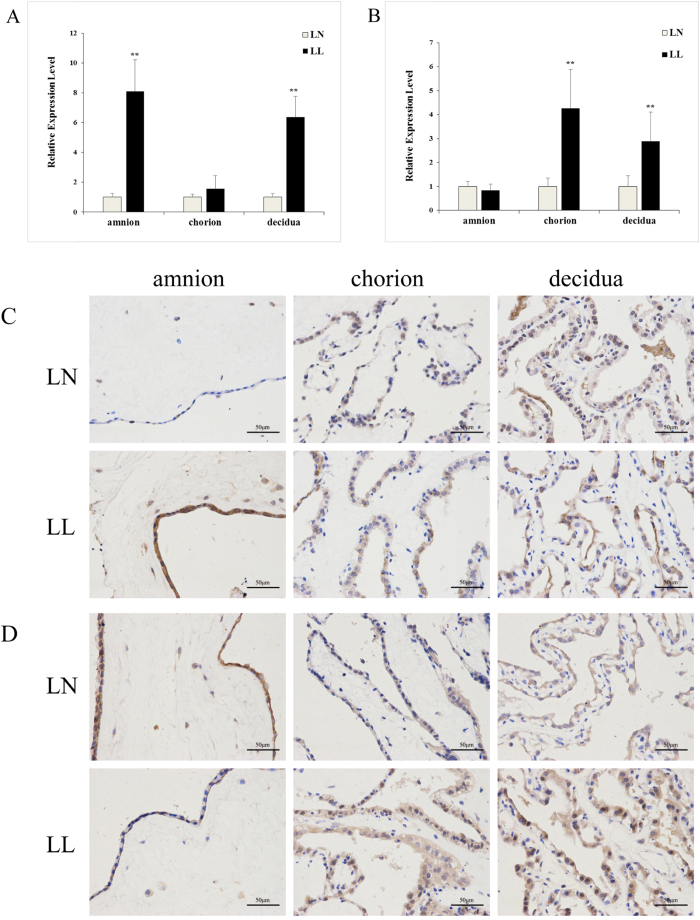
COX-2 and FOS expression in the amnion, chorion and decidua of LL and LN. (**A**) COX-2 mRNA level in the amnion, chorion and decidua of LL and LN as determined by qRT-PCR. (**B**) The expression levels of FOS in the amnion, chorion and decidua of LL and LN were detected by qRT-PCR. (**C**) COX-2 immunohistochemical results in the amnion, chorion and decidua of LL and LN. Original magnification, 400×. (**D**) FOS immunohistochemical result in the amnion, chorion and decidua of LL and LN. Original magnification, 400×. All of the error bars indicate SD. *P < 0.05, **P < 0.01.

To study the DEmRNA functions, we performed a GO analysis of LL, QL, QS and overlapping DEGs. The top 10 GO terms are shown in Fig. 4. For DEGs, the GO term with the highest annotation value is involved in hormone stimulus, including “response to organic substance”, “response to endogenous stimulus”, “response to hormone stimulus”, “response to oestrogen stimulus” and “response to steroid hormone stimulus”, demonstrating the key roles of various hormones in the onset of labour, which is consistent with the previous theory (8,22). In addition, some representative functional terms are involved in immune function, such as “immune response”, “defence response”, and “inflammatory response”, and some enriched terms are connected with the triglyceride metabolic process, cytokine activity and regulation of cell growth. Cyclooxygenase 2 (COX2), also known as prostaglandin G/H synthase 2 (PGHS-2), is the rate-limiting enzyme in the biosynthesis of prostaglandins (PGs). According to the RNA-seq data, there is a 4-fold expression in LL compared with that in LN, and the high expression of COX2 leads to an increase in PGs. The relative quantitative expression of COX2 is significantly higher in LL than that in LN, even over 8-fold for amnion (Fig. 5A), which was confirmed by immunohistochemistry (Fig. 5C).

To identify the commonly influenced mechanisms of the miRNAs and mRNAs, miRNA putative targets and DEGs were analysed using DAVID bioinformatics resources according to GO terms. Because miRNAs tend to downregulate target mRNAs, the expression of a genuine target mRNA is expected to be anticorrelated with miRNA expression. As a result, a total of 161, 189 and 340 inversely correlated genes were obtained by a comparison of LL, QS and QL with LN (Supplementary Table S7) and were defined as DEmiRNA targets. To further understand the miRNA regulatory function of labour onset in different pig breeds, five overlapping DEmiRNAs (ssc-miR-132, ssc-miR-323, ssc-miR-19a, ssc-miR-664-3p and ssc-miR-296-5p) were used for the gene ontology and pathway analyses in the DAVID software. For the overlapping DEmiRNA target genes, the annotation cluster with the highest score was involved in hormone stimulus, including “response to organic substance”, “response to endogenous stimulus”, “response to steroid hormone stimulus” and cell adhesion. The KEGG pathway analysis showed that most enriched pathways are associated with the inflammation response and hormone metabolism, such as “MAPK signalling pathway”, “p53 signalling pathway”, “cytokine-cytokine receptor interaction”, “androgen and oestrogen metabolism”, and “steroid hormone biosynthesis”. In addition, apoptosis and the adherens junction pathway are enriched. Interestingly, the overlapping DEmiRNA target-enriched GO terms and KEGG pathways were similar to the results that were obtained from the differentially expressed gene profile, indicating that the miRNA regulation of gene targets may have a tremendous effect on the overall gene signature in terms of hormone stimulus and immune response to labour onset.

### Network analysis of differentially expressed genes

**Figure 6:**
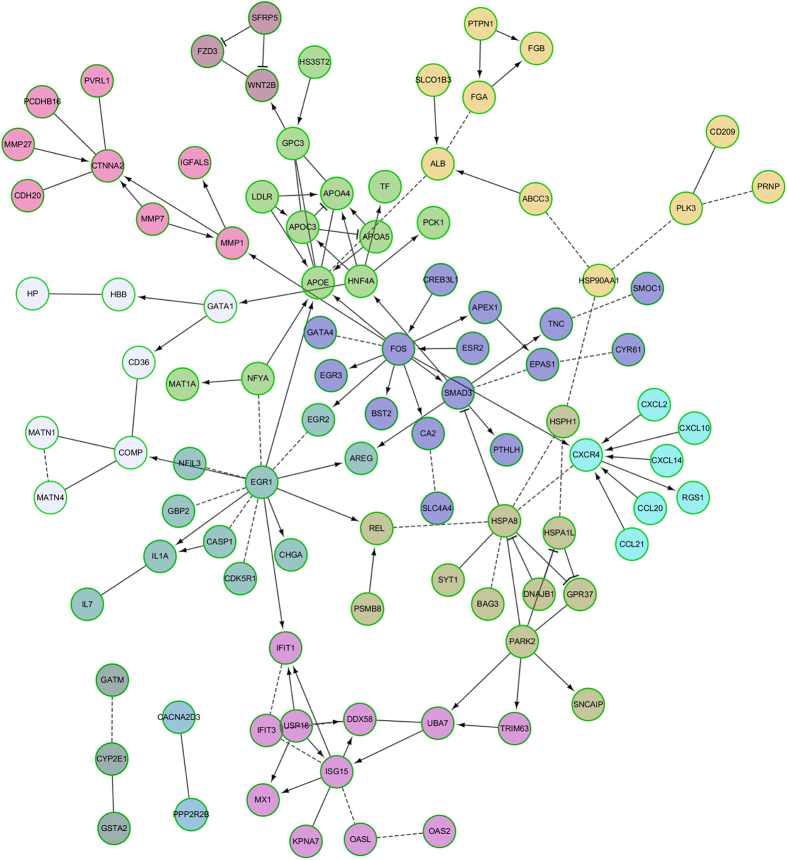
Functional interaction network of DEGs (IFs) in LL compared to those in LN. The effect of the interaction is represented by arrows, bar-headed lines, straight line and imaginary line. “→” for activating/catalysing, “-|” for inhibition, “-” for FIs that were extracted from complexes or inputs, and “---” for predicted FIs.

The Reactome plug-in in the Cytoscape software was used to visualise the functional interaction (FI) network of DEGs and to further identify major functional genes. The FI network of DEGs is shown in Fig. 6. A number of differentially expressed genes, such as FOS, SMAD3, HSPA8, CXCR4, EGR1, HNPF4, CTNNA2, APOE and PARK2, were found to be key node genes. Then, the network was constructed using the enriched genes that are involved in hormone stimulus and the immune response (Supplementary Fig. S1-2), with FOS, SMAD3, HNF4A, HSPA8 and CXCR4 as the central node genes. The regulatory direction of these node genes was also checked in QS and QL, where FOS and HNF4A were altered in the same direction in QL, LL and QS and SMAD3 was altered in the same direction in LL and QS. SMAD3 proteins are signal transducers and transcriptional modulators that mediate multiple signalling pathways. The HSPA8 gene encodes a member of the heat shock protein 70 family, which can activate the MAPK signalling pathway. FOS proteins have been implicated as regulators of cell proliferation, differentiation, and transformation. The relative quantitative expression of the FOS gene was tested by qPCR and showed a significant difference between LN and LL in the chorion and decidua tissue (Fig. 5B). The relatively high expression in LL was also confirmed by immunohistochemistry (Fig. 5D).

### Construction of the miRNA/mRNA network

**Figure 7:**
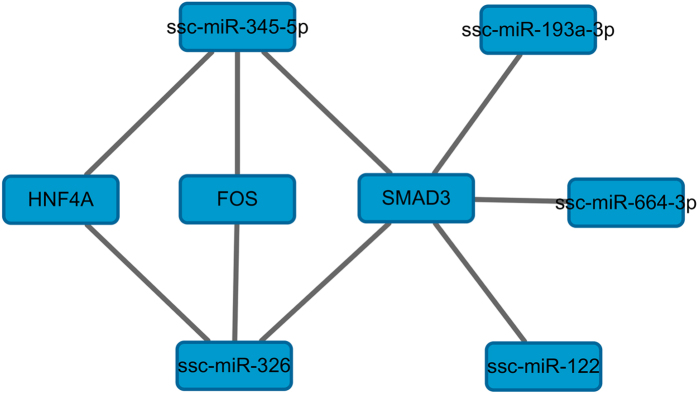
DEmiRNAs and their target node genes.

To further elucidate the interactive relationship between the node mRNAs and miRNAs, we searched for potential regulating miRNAs. The inversely correlated miRNAs were identified by comparing the miRNAs from target prediction with those from miRNA expression profiling, and miRNA/mRNA networks were constructed using the Cytoscape software. For further research, each miRNA/mRNA pair was scored to examine the target relationship and negative correlation of miRNA/mRNA networks (Fig. 7), and we found that miR-345-5p and miR-326 correlate with targets HNF4A, FOS and SMAD3, whereas miR-193a-3p, miR-664-3p and miR-122 correlate with target SMAD3. These results indicate that miRNA/mRNA networks indirectly reflect the expression patterns of node mRNAs and miRNAs and that understanding the structure of the miRNA/mRNA networks could provide more insight into labour onset.

### Identify the essential factors that are associated with a shorter gestation period

The essential factors that are associated with a shorter gestation period were identified by a functional classification of the 544 DEGs from QS/QL (*P *≤ 0.01; fold change ≤ 0.5 or fold change ≥ 2). Among the 544 DEGs, 307 genes were up-regulated, and 237 were down-regulated (Supplementary Table S6). These DEGs were analysed using DAVID bioinformatics resources according to GO terms. The up-regulated DEGs derived from QS/QL are enriched in immune-related terms. For example, CRP, CCL21, C5, ESR2, PENK, REG3G and SCG2 are genes that are involved in the initiation of the inflammatory response and in the effective stage of the inflammatory response. The level of immunoglobulin (lgG) in Qingping pig is remarkably higher than that detected in other species^23^. The expression of selected genes, including PTPRD, NTRK2 and PTPRS, is enriched in the term “immunoglobulin”.

## Discussion

To our knowledge, this is the first comprehensive report about the miRNA and mRNA signatures in the placenta, and the overall findings in the study will facilitate our understanding of the molecular events underlying the transition of the placenta from a refractory to a contractile state, the roles of microRNAs, their targets, and their transcriptional and hormonal regulation.

Transcriptome analysis of sows’ placentas. Generally, the transcriptome includes all of the RNA transcripts in the cell. The transcriptome reflects the genes and sRNAs that are actively expressed at any given time. The placental RNAs and sRNAs of sows before labour onset and during labour were sequenced in a novel way. The original sequencing images were converted into raw reads by base calling, and clean reads were then obtained from the raw reads. In the present study, 12 libraries of miRNAs and mRNAs were created from high-throughput sequencing data, and the molecular mechanisms and signalling pathways controlling the onset of labour were investigated by analysing the transcriptome sequencing data of sows’ placentas, which, in theory, could be a powerful tool for explaining the pivotal factors that are involved in the shorter gestation period of Qingping sows and could be considered the models of choice for research into human obstetrics. However, why Qingping sows have the unique characteristic of a shorter gestation period remains to be further elucidated and could provide new clues for preterm delivery.

An alteration in the hormone level is associated with the onset of labour. Hormone metabolism plays an essential role in the initiation of labour. Because of its relaxation effect, progesterone is considered one of the primary hormones that are responsible for the maintenance of pregnancy^7^. It has been widely accepted that progesterone withdrawal caused by the regression of the corpus luteum through a high level of prostaglandins is associated with the initiation of labour. The GO and KEGG pathway analyses of the DEGs and DEmiRNAs provided substantial evidence that DEGs and DEmiRNAs can alter the hormone level associated with the onset of labour. Numerous genes, including CPS1, CA2, CFTR, LDLR, OXTR and FOS, are enriched in the DAVID term of hormone stimulus. In LL, the expressions of COX2, IL-1β, IL-6 and IL-8 are markedly increased. It is noteworthy that the type-2 cyclo-oxygenase enzyme is the expression product of COX2, which suggests that the up-regulation of COX2 corresponds to the increasing concentration of PG. TNF-α and TGF-β, which are both highly expressed in the placenta, regulate the secretion of hormones as related to labour^9^. Arachidonic acid is the precursor to assemble PGs and is inextricably intertwined with the initiation of labour. The DEGs of CBR3, CYP2E1, GPX3 and GPX5 are enriched in the function of arachidonic acid metabolism. As previously reported, PGF2α induces P450c17 expression in the corpus luteum^24^. Because P450c17 stimulates the transformation from progesterone to androstenedione, increased P450c17 prompts the synthesis of oestrogen followed by the initiation of labour. Obviously, the transformation from progesterone to androstenedione leads to a reduction in the progesterone concentration in the serum^25^, which has an inductive effect on the initiation of labour. In previous studies, miR-122, miR-132, miR-137 and miR-151-5p have been shown to affect progesterone production^26^, and P4 could regulate the expression of DEmiRNAs, such as miR-345-5p and miR-193a-3p^27^. miR-19a and miR-19b are influenced by E2^28^. Our results show that a significant number of DEGs and DEmiRNAs play a vital role in regulating the interaction between the hormone level and labour onset.

Immunity is involved in the process of labour onset. During pregnancy, the function of maternal immunity changes within the uterus, where innate, pro-inflammatory immune responses are advantageously regulated to prevent immunological resistance of foetal allograft^29^. Immunity is related to the initiation of term and preterm labour^30^,^31^,^32^. The destruction of the balance of cytokines by bacteria or other factors increases the production of pro-inflammatory cytokines at the placenta and activates the process of delivery^33^. In the present study, a number of immune-related differentially expressed genes are identified, and among them, interleukin gene family (IL) and chemokine (CCL, CXCR, CXCL) are differentially expressed. Tissue macrophages synthesise neutrophil chemoattractants CXCL1/CXCL2 in response to inflammatory stimulation, with CXCL1/CXCL2 neutrophil recruitment as an important early step in controlling tissue infections or injury^34^. Inflammatory cytokines may affect the activity of receptors in the placenta and uterus^9^,^32^, which results in prenatal environment revulsion. As a critical factor mediating the process of immunity and inflammation, TGF-β induces the combination of FOS and JUN to form the protein complex and exerts its influence on proliferation, differentiation and apoptosis^35^,^36^. Interestingly, many immune- and inflammatory-related DEGs and DEmiRNAs have a high expression. The enriched GO term of genes, such as immune response, immune cell activation and immunoglobulin, and KEGG pathways, such as “MAPK signalling pathway”, “p53 signalling pathway”, and “cytokine-cytokine receptor interaction”, also indicate their immune function in placenta, which strengthens in the early stage of labour onset.

Cell adhesion molecules (CAMs) are proteins that are located on the cell surface and bind either with other cells or with the extracellular matrix (ECM) in a process called cell adhesion^37^, which is also related to the onset of labour^38^. Several cell adhesion-related genes are up-regulated in both LN and LL, including cadherins (CDH20, CDHR4, PCDH19, and CDHB16) and selectins (SELL). In fact, CAMs are signals in the inflammatory response^39^. DEmiRNA targets are also enriched in the adheren junction pathway and cytokine-cytokine receptor interaction. Pro-inflammatory cytokines, such as TNF-α and IL6, are released by neutrophils and macrophages to induce the expression of adhesion molecules and to recruit leukocytes^40^. Simply put, CAMs induce labour onset, through which the inflammatory response is mediated.

Apoptosis is related to the onset of labour. Apoptosis is the process of programmed cell death and may occur in multicellular organisms. This process is regulated by conserved genes, such as Bcl-2 family, caspase family, oncogene C-myc and anti-oncogene P53^41^, some of which were detected as DEGs in LN/LL, with the BCL2-associated athanogene 3 (BAG3) and FOS genes being down-regulated. Moreover, BAG3, SMAD3, CXCR4, NR4A2 and TNFRSF12A are enriched in the term “regulation of cell death”. SMAD3 is a key molecule that mediates TGF-beta activity, which leads to inflammation and apoptosis^42^. Two theories have been suggested regarding the initiation of the apoptotic mechanism: the TNF-induced (tumour necrosis factor) model and the Fas-Fas ligand-mediated model^43^, both of which involve receptors of the TNF receptor (TNFRSF12A in the DEGs) family coupled to extrinsic signals. According to previous studies, the premature rupture of foetal membranes is induced by apoptosis in human placenta^44^. Overall, apoptosis should be a prerequisite to the start of delivery.

Essential factors for the shorter gestation period. According to the DEGs and the specific functions mentioned above, two distinct gene-expressing models have been developed from the two groups of Qingping sows with different gestation periods. The up-regulated DEGs derived from QS/QL are enriched in immune-related terms. For example, CRP, CCL21^45^, C5^46^, ESR2, PENK^47^, REG3G^48^ and SCG2^49^ are genes that are involved in the initiation of inflammatory response and the effective stage of the inflammatory response. Functional studies reveal that CRP, one of the marker genes of inflammation, is capable of activating the NF-κB signalling pathway and stimulating the secretion of interleukin IL-6 and monocyte chemoattractant protein (MCP-1), which lead to the activation of platelet and induction of apoptosis^50^. Given that the oestrogen receptor genes ESR2 and ESR1 are connected to antigen-antibody reaction, it is widely accepted that ESR2 can also be treated as a marker gene of inflammation^51^.

The level of immunoglobulin (lgG) is remarkably higher in Qingping pigs than in other species^23^. The expression of selected genes, including PTPRD, NTRK2 and PTPRS, is enriched in the term of “immunoglobulin”. Because the upregulated genes are associated with the immune response and apoptosis, the shorter gestation period can be partly explained by the stronger immune system, which is well supported by the fact that Qingping pigs are less vulnerable to various diseases in practice. The stronger immune system intensifies the immune response that is linked to the initiation of labour and accelerates the initial process of labour, resulting in a shorter gestation period compared with that of other species. Therefore, more effective functions in the immune response are crucial factors that are associated with the shorter gestation period of Qingping sows.

One decade ago, the shorter gestation period of the Qingping sow was considered one of its physiological characteristics; we can now reveal the internal factors at the molecular level. From this point of view, these factors that are associated with shorter gestation period can serve as hints for the pathogenesis of preterm labour in humans when the inflammation or immune response play a leading role in preterm labour^52^. Accordingly, a stronger inflammation or immune response can increase susceptibility to preterm labour.

Signalling during labour. The observation of the DEGs that are involved in isolated pathways and the gene functional interaction network indicate that the synergic interaction of these genes plays a vital role in the efficiency of their functions. When the maternal nutrient provision can hardly meet the foetus’ requirements, which occurs with the development of the foetus during the latter gestation period^53^, the stress response is triggered, which in turn leads to the secretion of signalling factors, such as “stress” hormone and chemotactic factors. Through blood circulation, signalling factors are transported to the placenta, where some hormone receptors and unspecific receptors are located. The expressions of certain transcriptional factors and signalling transduction molecules are regulated when receptors are aware of the alteration in the concentration of signalling factors. The differential expression of FOS, SMAD3, HNF4A and TNF-α is observed between LN and LL, which is consistent with our RT-PCR results. The synthesis and release of TNF-α and TGF-β by foetal membranes at term gestation are regulated by several hormones that are potentially involved either in the maintenance of pregnancy or in the parturition process^54^. Therefore, it can be concluded that inadequate maternal nutrient supply is likely to be the original signal that is responsible for the initiation of labour.

In conclusion, a significant number of commonly dysregulated mRNAs and a subset of commonly dysregulated miRNAs are obtained by comparing the mRNA and miRNA expression profiles of labour onset. These dysregulated mRNAs and miRNAs are involved in the response to hormone stimulus, immune response, and apoptosis and may thus represent good candidate targets for future research. The overall results of this study facilitate our understanding of the mechanism underlying labour onset by expanding the number of porcine miRNAs and establishing the biological basis for the study of miRNAs in regulating labour onset.

## Materials and Methods

### Animal and tissue collection

Four groups (n = 3) of multiparous sows with a parity of three were selected for this study. Group one consisted of Large White sows 112 days after insemination with no sign of labour (LN); group two was composed of Large White sows 114 days after insemination with distinct signs of labour and before the birth of the first cub (LL). In addition, two groups of Qingping sows 112 (QS) and 114 (QL) days after insemination with the same signs of LL were selected. After the sows described above were slaughtered, the amnion, chorion and decidua tissues were separated and collected from twelve sows. All of the studies involving animals were conducted according to the regulation (No. 5 proclamation of the Standing Committee of Hubei People’s Congress) approved by the Standing Committee of Hubei People’s Congress, P. R. China. The sample collection was approved by the Ethics Committee of Huazhong Agricultural University with the permit number No. 30700571 for this study. The animals were allowed access to feed and water ad libitum under the same normal conditions and were humanely sacrificed as necessary to ameliorate suffering. The methods were carried out in accordance with the approved guidelines.

### RNA extraction

The total RNA was extracted from each of the three parts of the placenta using TRIzol reagent (Invitrogen, USA) according to the manufacturer’s recommendations. The total RNA was treated with RQ1 DNase (Promega, USA) to remove DNA. The quality and quantity of the purified RNA were determined by measuring the absorbance at 260 nm/280 nm (A260/A280) using SmartSpec Plus (Bio-Rad, USA). The RNA integrity was further verified by 1.5% agarose gel electrophoresis. The total RNA from the amnion, chorion and decidua of the same sow was pooled equally.

### mRNA sequencing and statistical analysis

For each of the twelve pooled samples, 10 μg of RNA was used for RNA-seq library preparation. Polyadenylated mRNAs were purified and concentrated with oligo(dT)-conjugated magnetic beads (Invitrogen, USA) before being used for directional RNA-seq library preparation. The purified mRNAs were iron-fragmented at 95 °C, followed by end repair and 5′ adaptor ligation. Then, reverse transcription was performed with an RT primer harbouring a 3′ adaptor sequence and a randomised hexamer. The cDNAs were purified and amplified by PCR, followed by purifying and quantifying the PCR products corresponding to 200–500 bp. For high-throughput sequencing, paired-end 100-bp sequencing of the cDNAs was performed on the Illumina HiSeq 2000 system (Illumina, USA). The obtained sequence reads (Fastq files) were checked by FastQC software. Clean reads with a minimum length of 16 nt were obtained by trimming the raw reads and deleting the low-quality reads, and then the clean reads were mapped to the porcine reference genome sequence (Sscrofa10.2) with 2-bp mismatch using the TopHat software^55^. We estimated the gene expression level using RNA-Seq by the reads per kilobase of genes per million mapped reads (RPKM). EdgeR^56^ was used to filter differentially expressed genes (DEGs). An integrated analysis of different functional databases was performed using the “functional annotation clustering” tool of the database for annotation, visualisation, and integrated discovery^57^. For gene interaction network analysis, the Reactome plug-in^58^ in the Cytoscape software^59^ was used to visualise the general relationship between differentially expressed genes.

### miRNA profiling and statistical analysis

A total of 3 μg of total RNA was used for small RNA cDNA library preparation with the Balancer NGS Library Preparation Kit for small/microRNA (GnomeGen) by following the manufacturer’s instructions. Briefly, RNAs were ligated to 3′ and 5′ adaptors sequentially, reverse transcribed to cDNA and then PCR amplified. The entire library was tested by 10% native PAGE gel electrophoresis, and bands corresponding to microRNA insertion were cut and eluted. After ethanol precipitation and washing, the purified small RNA libraries were quantified with Qubit Fluorometer (Invitrogen) and used for cluster generation and 36 nt single end sequencing analysis using the Illumina GAIIx (Illumina, San Diego, CA, USA) according to the manufacturer’s instructions. The resulting images were analysed to generate raw digital-quality data. After masking adaptor sequences and removing the contaminated reads, the clean reads were selected and aligned with the pig reference genome (ftp://ftp.ncbi.nlm.nih.gov/genomes/Sus_scrofa), followed by calculating the reads in different regions of the genome distribution. The clean reads were compared with the Rfam database (ftp://selab.janelia.org/pub/Rfam) to match the known lncRNA, miRNA, rRNA, snRNA, snRNA and tRNA sequences and were then compared with the pig mature miRNAs database in miRBase to identify mature miRNAs and count their reads. The clean reads that were not mapped to the mature miRNA database were further compared with the pre-miRNA database of pigs. The numbers of miRNA reads were normalised by transcripts per million (TPM) values (TPM=(miRNA total reads/total clean reads) × 10^6^). The differentially expressed miRNAs (DEmiRNAs) between samples were identified by the EdgeR program^56^ at *P *≤ 0.01 and fold change ≥ 2 or ≤0.5.

### miRNA target predictions and DAVID analysis

Because the online target gene prediction software contains no pig genes and cannot predict miRNA potential target genes, we used the miRanda software to find the DEmiRNA potential target genes in all of the pigs (+ CDS to the back of the exon were observed as 3′UTR; - to the CDS front of exon were observed as 3′UTR). KEGG pathway and GO Term analyses of these target genes were performed using DAVID bioinformatics resources.

### mRNA and miRNA analysis by synthesis

To identify the functional regulations from miRNAs to mRNAs, we combined the computational target predictions at the sequence level and the inverse expression relationships between miRNAs and mRNAs in context. Specifically, functional regulations were detected using a subset of miRNAs and genes that was characterised by (i) both miRNAs and genes being associated with labour onset, (ii) a regulatory relationship according to computational predictions, and (iii) expression profiles were strongly negatively correlated. Then, the miRNA/mRNA regulatory network was constructed using the popular Cytoscape software, and KEGG pathway and GO Term analyses of the target genes in the miRNA/mRNA network were performed to explore the functional roles of miRNAs using the DAVID^57^ bioinformatics resources.

### qRT-PCR analysis of miRNAs and mRNAs

RNAs were first reverse transcribed to cDNAs with the Thermo Scientific Maxima First Strand cDNA Synthesis Kit K1622 (Thermo, USA). Quantitative real-time PCR (qPCR) reactions were performed by Bio-RAD CFX Connect (Bio-RAD, USA) with twelve biological replicates, and each reaction was performed in triplicate. U6 RNA and β-actin were used as endogenous internal controls, and the relative expression levels were calculated with the 2^−ΔΔCT^ method.

### Immunohistochemistry

The three parts of the placenta were stored in 4% paraformaldehyde, dehydrated and embedded in paraffin. Then, 5-μm sections were cut on the microtome and placed onto clean, positively charged microscope slides, followed by heating the slides in a tissue-drying oven for 45 minutes at 60 °C, washing them with xylene 3 times for 5 minutes and hydrating them separately in 95%, 70%, 50%, and 30% ethanol for 2 minutes. Incubation with the primary antibodies (Santa Cruz Biotechnology, FOS: sc-253, COX2: sc-1746) was performed at room temperature for 2 h. DAB was used for visualisation after incubation with the secondary antibody (IgG), and the sections were then counterstained with hematoxylin and mounted for microscopic examination.

## Additional Information

**How to cite this article**: Li, H. *et al.* Integrated analysis of miRNA/mRNA network in placenta identifies key factors associated with labor onset of Large White and Qingping sows. *Sci. Rep.* **5**, 13074; doi: 10.1038/srep13074 (2015).

## Supplementary Material

Supplementary Information

## Figures and Tables

**Table 1:** Quantitative RT-PCR validation of differentially expressed genes.

Gene	LW-short VS LW-long				QP-short VS LW-short				QP-long VS LW-short			
	RNA-seq	*P* value	qPCR	*P* value	RNA-seq	*P* value	qPCR	*P* value	RNA-seq	*P* value	qPCR	*P* value
GGTA1	−1.95	<0.01	−2.17	<0.01	2.41	<0.01	1.33	0.08	1.46	<0.01	1.61	<0.01
LIPG	3.84	<0.01	4.35	<0.01	−1.96	<0.01	−9.46	<0.01	−1.42	0.03	−3.93	<0.01
CXCL14	−3.59	<0.01	−5.47	<0.01	2.97	<0.01	2.83	<0.01	3.04	<0.01	1.21	<0.01
APOE	3.46	<0.01	1.19	<0.01	−4.22	<0.01	−1.76	<0.01	−3.18	<0.01	−3.94	<0.01
RERG	−2.6	<0.01	−7.76	<0.01	2.58	<0.01	1.04	<0.01	1.55	0.11	−1.74	0.37
HSPA4	−1.47	0.01	−2.29	<0.01	1.43	0.01	1.65	0.02	1.11	0.31	1.05	0.27
IGFBP2	−1.53	0.02	−2.62	0.03	1.24	0.03	1.06	0.09	−1.84	<0.01	−4.33	0.04
TNFRSF12A	−2.07	<0.01	−3.52	0.03	2.08	<0.01	−2.14	<0.01	1.75	<0.01	−2.51	0.65
RAMP1	−1.08	0.07	−6.32	0.04	−1.46	<0.01	−31.96	<0.001	−4.14	<0.01	−7.96	<0.01
HBB	−2.74	<0.01	−1.62	0.05	1.91	0.02	1.67	0.05	−1.4	0.01	−1.95	0.07
HSP70	−18.97	0.31	−12.06	0.04	12.22	0.01	2.25	0.04	5.23	<0.01	1.32	0.43
SERPINA3-2	−4.03	0.01	−1.47	0.07	4.06	0.03	2.86	0.04	3.12	0.05	−1.46	0.33
MMP1	−4.3	0.25	−12.25	<0.01	3.46	<0.01	3.4	<0.001	1.45	0.08	1.56	0.04

qRT-PCR-based validation of GGTA1, LIPG, CXCL14, APOE, RERG, HSPA4, IGFBP2, TNFRSF12A, RAMP1, HBB, HSP70, SERPINA3-2, MMP1, CYP2C36 and PGHS-2 expression levels in LN, LL, QS and QL.
