# Supplementary material for: Integrated analysis of miRNA/mRNA network in placenta identifies key factors associated with labor onset of Large White and Qingping sows
Source: Sci Rep. 2015 Aug 14;5:13074. doi: 10.1038/srep13074 (PMC4536519; doi:10.1038/srep13074)
Supplement: Supplementary Information [file srep13074-s1.doc]

**Supplementary Information for:**

**Integrated analysis of miRNA/mRNA network in placenta identifies key factors associated with labour onset of Large White and Qingping sows**

Huanan Li^1^, Bin Wu^1^, Junnan Geng^1^, Jiawei Zhou^1^, Rong Zheng^1^, Jin Chai^1^, Fenge Li^1^, Jian Peng^2, 3^*, Siwen Jiang^1, 3^*

^1^Key Laboratory of Swine Genetics and Breeding of Agricultural Ministry and Key Laboratory of Agricultural Animal Genetics, Breeding and Reproduction of Ministry of Education, College of Animal Science and Technology, Huazhong Agricultural University, Wuhan 430070, People’s Republic of China

^2^Department of Animal Nutrition and Feed Science, College of Animal Science and Technology, Huazhong Agricultural University, Wuhan 430070, People’s Republic of China

^3^The Cooperative Innovation Center for Sustainable Pig Production, Wuhan 430070, China People’s Republic of China

* [jiangsiwen@mail.hzau.edu.cn](mailto:jiangsiwen@mail.hzau.edu.cn)


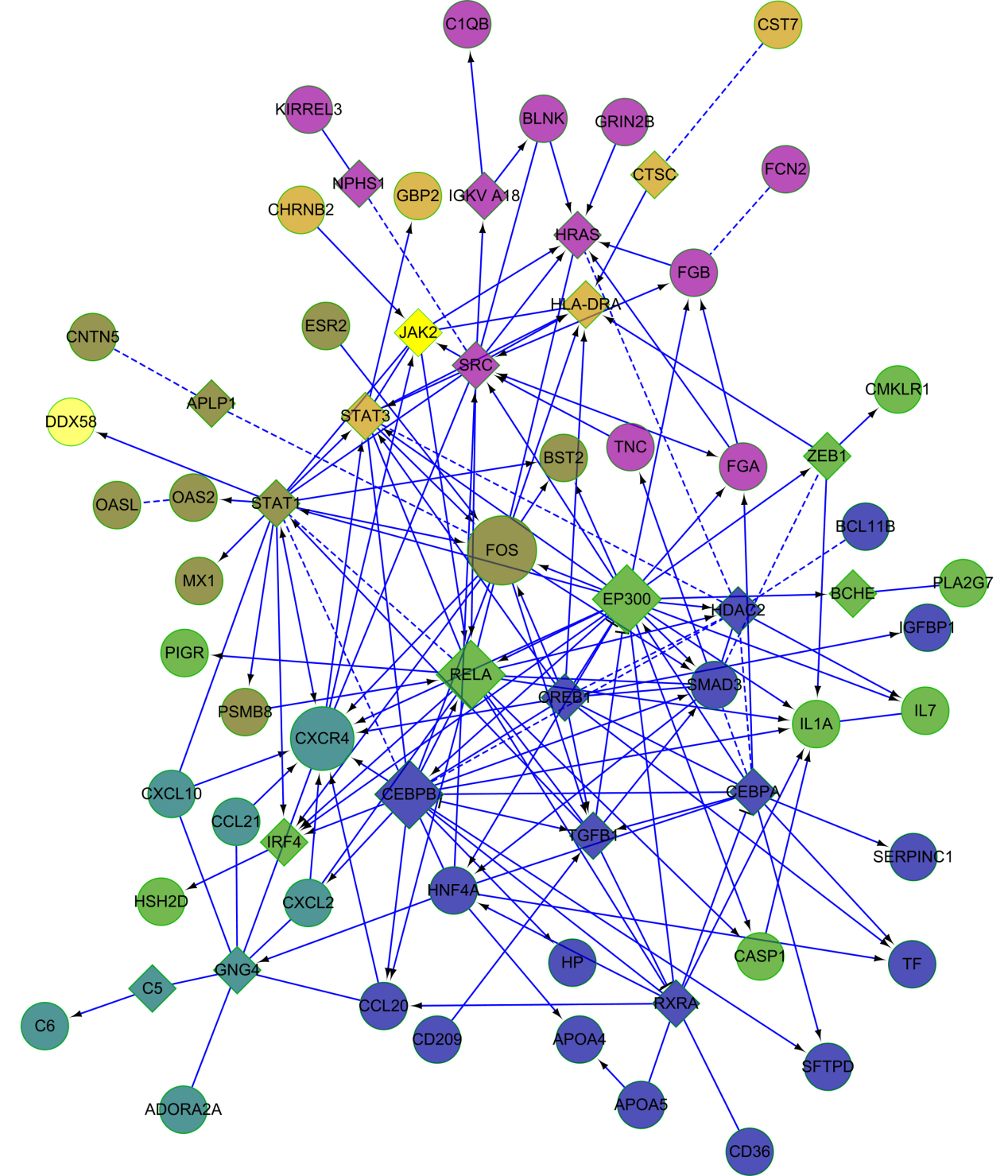
Supplementary Figure S1: Functional network of Genes related to hormone stimulus in LL/ LN. The effect of the interaction is represented by arrows, bar-headed lines, straight line and imaginary line. "→" for activating/catalysing, "-|" for inhibition, "-" for FIs that were extracted from complexes or inputs, and "---" for predicted FIs.


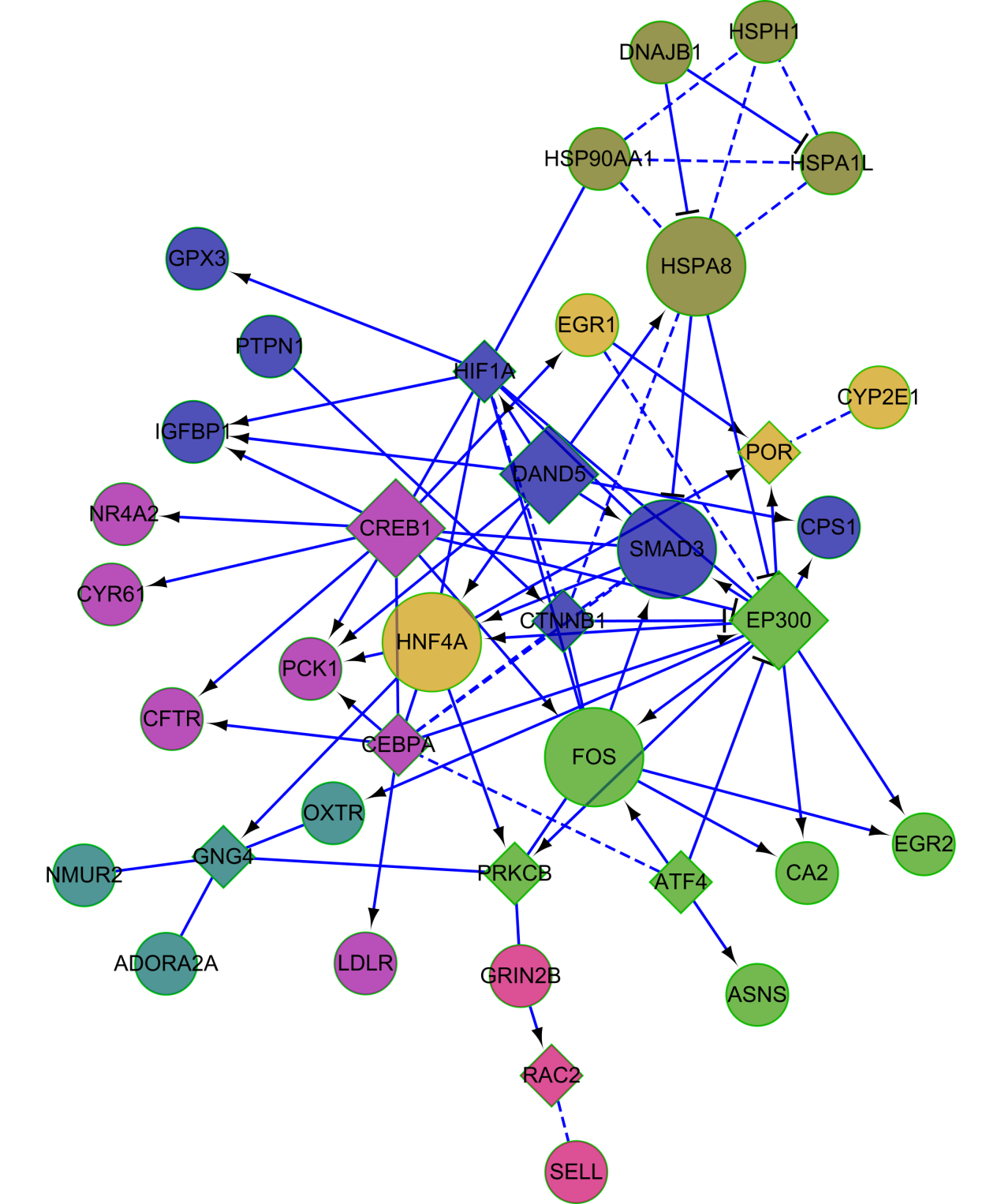


Supplementary Figure S2: Functional network of genes related to immune and inflammatory response in LL/ LN. The effect of the interaction is represented by arrows, bar-headed lines, straight line and imaginary line. "→" for activating/catalysing, "-|" for inhibition, "-" for FIs that were extracted from complexes or inputs, and "---" for predicted FIs.

Supplementary Table S1: Differentially expressed genes (DEGs) in LL

| Gene | logFC | PValue | LL1 | LL2 | LL6 | LN1 | LN2 | LN3 | QS1 | QS2 | QS3 | QL1 | QL2 | QL3 | Name |
| --- | --- | --- | --- | --- | --- | --- | --- | --- | --- | --- | --- | --- | --- | --- | --- |
| gene10031 | -2.984862817 | 0.003094802 | 0 | 0.02 | 0.04 | 0.25 | 0 | 0.4 | 0.1 | 0.16 | 0.05 | 0.05 | 0.05 | 0.18 | LOC100515144 |
| gene10072 | 1.108213063 | 0.000375603 | 14.25 | 6.74 | 7.45 | 4.11 | 4.62 | 5.54 | 6.71 | 9.7 | 5.77 | 10.19 | 20.41 | 14.78 | LOC100522873 |
| gene10124 | -1.809209727 | 2.07E-06 | 0.14 | 0.37 | 0.19 | 0.95 | 1.2 | 0.56 | 0.49 | 0.15 | 0.35 | 0.23 | 0.71 | 0.87 | LOC100738531 |
| gene10144 | -1.152295467 | 0.000936852 | 1.53 | 2.03 | 3.5 | 4.99 | 2.48 | 8.92 | 2.06 | 3.48 | 1.88 | 2.58 | 2.99 | 4.54 | C1QB |
| gene10150 | -1.03340045 | 0.002419529 | 1.6 | 1.78 | 2.09 | 6.4 | 2 | 3.43 | 2.88 | 3.54 | 2.87 | 2.66 | 2.21 | 0.86 | LOC100739392 |
| gene10191 | -1.296929563 | 0.003990746 | 0.24 | 0.41 | 0.36 | 1.18 | 0.73 | 0.65 | 0.27 | 1.34 | 1.43 | 1.04 | 1.02 | 1.19 | TRIM63 |
| gene10194 | -1.287915571 | 6.94E-05 | 2.06 | 3.04 | 3.94 | 7.03 | 12.4 | 3.28 | 7.01 | 2.17 | 2.58 | 4.8 | 3.07 | 4.87 | CATSPER4 |
| gene10257 | -1.95857451 | 8.95E-07 | 0.14 | 0.49 | 0.37 | 0.59 | 1.75 | 1.79 | 0.59 | 0.23 | 0.05 | 0.13 | 0.13 | 0.32 | MATN1 |
| gene10270 | 2.838743896 | 3.26E-09 | 0.16 | 2.42 | 0.22 | 0.25 | 0.09 | 0.12 | 0.15 | 0.32 | 0.38 | 0.18 | 0.33 | 1.38 | LOC100520618 |
| gene10309 | -1.191588218 | 0.00020514 | 2.1 | 3.33 | 3.3 | 7.49 | 5.67 | 7.86 | 6.39 | 2.42 | 7.3 | 4.03 | 2.67 | 4.53 | SYNC |
| gene10317 | 5.349920213 | 0.000292606 |  |  |  |  |  |  |  |  |  |  |  |  |  |
| gene10342 | 1.082304933 | 0.008841983 | 10.68 | 5.87 | 9.59 | 6.1 | 3.6 | 3.34 | 6.19 | 8.8 | 8.89 | 7.49 | 8.98 | 3.93 | LOC100737219 |
| gene10344 | -1.463463696 | 0.004463852 | 0.18 | 0.16 | 0 | 0.07 | 0.24 | 0.89 | 0.47 | 0.43 | 0.31 | 0.29 | 0.28 | 0.07 | LOC100525228 |
| gene10373 | -1.685370817 | 0.000210101 | 0.13 | 0.64 | 0.81 | 2.13 | 1.45 | 1.67 | 1.52 | 0.65 | 1.18 | 1.53 | 1.17 | 1.65 | HPCAL4 |
| gene10413 | 2.515012495 | 1.29E-07 | 1.47 | 2.83 | 1.64 | 0.21 | 0.54 | 0.34 | 0.19 | 1.41 | 0.62 | 2.58 | 1.94 | 1.26 | LOC100623971 |
| gene10459 | 1.71294485 | 6.65E-06 | 1.58 | 1.71 | 3.28 | 0.57 | 1.07 | 0.4 | 0.33 | 1.32 | 0.78 | 0.5 | 0.65 | 0.84 | LOC100525586 |
| gene10498 | -2.284716405 | 4.20E-08 | 0.04 | 0.18 | 0.17 | 0.86 | 0.3 | 0.88 | 0.49 | 0.17 | 0.17 | 0.47 | 0 | 0.14 | LOC100515669 |
| gene10499 | -1.982502209 | 7.11E-09 | 0.18 | 0.58 | 0.91 | 1.95 | 2.24 | 2.56 | 0.98 | 0.42 | 1.29 | 0.91 | 0.8 | 0.93 | DSG1 |
| gene10511 | -2.017274273 | 1.32E-07 |  |  |  |  |  |  |  |  |  |  |  |  |  |
| gene10512 | -1.530008241 | 9.08E-07 | 17.54 | 17.31 | 29.49 | 24.69 | 31.72 | 141.43 | 88.83 | 21.06 | 15.34 | 17.7 | 23.26 | 7.6 | MEP1B |
| gene10561 | -2.564387021 | 0.000423826 | 0.02 | 0.03 | 0.06 | 0.17 | 0.31 | 0.25 | 0.32 | 0.31 | 0.08 | 0.12 | 0.11 | 0.27 | TTLL7 |
| gene10568 | -1.473776309 | 3.26E-06 | 6.72 | 6.75 | 5.38 | 35.46 | 6.4 | 14.2 | 6.69 | 11.56 | 7.56 | 11.68 | 4.67 | 5.39 | IFI44 |
| gene10574 | 1.130213802 | 0.000251474 | 185.89 | 37.56 | 53.04 | 43.29 | 42.47 | 52.99 | 33.42 | 39.16 | 38.88 | 135.89 | 40.69 | 61.29 | LOC100525703 |
| gene10607 | 1.171572737 | 0.000646401 | 1.76 | 4.7 | 6.02 | 1.61 | 1.91 | 2.33 | 2.11 | 3.1 | 3.14 | 1.29 | 4.01 | 2.26 | CTH |
| gene10613 | -1.112601248 | 0.008161315 | 3.23 | 1.57 | 3.44 | 3.04 | 8.56 | 6.02 | 4.81 | 3.83 | 3.09 | 3.16 | 2.55 | 2.32 | LOC100738593 |
| gene10636 | -1.012066315 | 0.005993213 | 7.72 | 6.29 | 4.66 | 15.92 | 9.69 | 15.19 | 5.07 | 23.28 | 13.09 | 14.41 | 9.74 | 2.34 | LOC100623941 |
| gene10677 | 1.046755121 | 0.007820408 | 2.22 | 2.61 | 2.41 | 1.61 | 1.25 | 0.8 | 3.42 | 2.68 | 2.04 | 2.32 | 2.51 | 3.86 | TMEM61 |
| gene10679 | 2.120570447 | 3.08E-08 | 4.78 | 0.41 | 0.6 | 0.36 | 0.46 | 0.69 | 0.34 | 0.7 | 0.61 | 1.5 | 0.79 | 0.18 | LOC100620608 |
| gene1075 | -1.680356831 | 5.41E-07 | 1.96 | 1.88 | 1.81 | 3.72 | 12.44 | 2.48 | 6.09 | 0.49 | 0.87 | 2.32 | 2.5 | 1.28 | SERPINB7 |
| gene1076 | -1.364774478 | 1.42E-05 | 8.14 | 9.74 | 11.71 | 15.59 | 32.41 | 31.99 | 33.84 | 2.03 | 5.43 | 6.58 | 6.64 | 6.31 | LOC100153783 |
| gene10766 | -1.227254838 | 0.009959106 | 0.68 | 0.26 | 0.76 | 1.55 | 1.31 | 1.06 | 1.12 | 0.99 | 0.18 | 0.17 | 0.58 | 0.52 | LOC100525230 |
| gene10768 | 1.279447888 | 3.35E-05 | 1873.3 | 3010.1 | 1567.1 | 746.19 | 1434.5 | 608.21 | 578.92 | 1203.2 | 1050.6 | 1646.8 | 941.89 | 1358.7 | TSPAN1 |
| gene10791 | 1.057017161 | 0.000779939 | 17.42 | 7.42 | 6.44 | 3.99 | 4.16 | 8.3 | 7.83 | 8.3 | 8.62 | 6.06 | 14.09 | 15.5 | PLK3 |
| gene10827 | -1.080629888 | 0.008696071 | 1.37 | 0.72 | 0.52 | 0.92 | 1.35 | 3.94 | 1.2 | 0.82 | 0.57 | 2.33 | 1.64 | 0.47 | LOC100520438 |
| gene10849 | -1.282739313 | 0.009039798 | 0.13 | 0.28 | 0.13 | 0.35 | 0.73 | 0.43 | 0.67 | 0.31 | 0.38 | 0.7 | 0.26 | 0.28 | LOC100524028 |
| gene10856 | -1.332955672 | 0.000625885 | 0.52 | 0.39 | 0.53 | 0.53 | 2.06 | 1.09 | 0.36 | 0.43 | 0.68 | 0.51 | 0.42 | 0.69 | LOC100526058 |
| gene10887 | -1.595846265 | 0.005840954 | 0.31 | 0.12 | 0.05 | 0.67 | 0.48 | 0.5 | 0.34 | 0.16 | 0.2 | 0.12 | 0.36 | 0.11 | LOC100156092 |
| gene10895 | -2.112967968 | 0.002559034 | 0.03 | 0.07 | 0 | 0.24 | 0.19 | 0.16 | 0.24 | 0.14 | 0.09 | 0 | 0 | 0.4 | LOC100625760 |
| gene1091 | -1.230644982 | 0.005270094 | 0.1 | 0.13 | 0.29 | 0.34 | 0.51 | 0.37 | 0.17 | 0.41 | 0.25 | 0.19 | 0.12 | 0.15 | CDH20 |
| gene10925 | -1.254086339 | 5.00E-05 | 47.5 | 23.12 | 61.3 | 82.03 | 162.5 | 85.4 | 82.37 | 26.85 | 56.67 | 33.74 | 35.63 | 32.96 | LOC100153368 |
| gene10945 | 1.321397726 | 0.003426128 | 1.63 | 0.46 | 1.02 | 0.72 | 0.39 | 0.22 | 0.35 | 1.11 | 0.88 | 0.81 | 0.46 | 0.26 | LOC100154575 |
| gene10951 | -1.277738581 | 3.53E-05 | 104.76 | 52.68 | 95.62 | 147.11 | 330.39 | 170.8 | 177.96 | 76.55 | 85.64 | 44.38 | 65.84 | 49.76 | MYLIP |
| gene10957 | -2.605661567 | 1.55E-08 | 1.57 | 0.97 | 1.31 | 2.09 | 14.19 | 7.06 | 1.26 | 0.83 | 0.35 | 0 | 1.87 | 0.85 | LOC100522879 |
| gene10978 | 1.397655593 | 6.39E-06 | 219.85 | 348.38 | 209.75 | 104.84 | 21.17 | 191.31 | 28.68 | 338.72 | 179.7 | 199.1 | 214.69 | 268.43 | LOC100512542 |
| gene10979 | 1.45648519 | 2.66E-06 | 1248.3 | 2116.2 | 1382.1 | 557.22 | 153.77 | 1142.5 | 263.88 | 1188.7 | 673.06 | 1247.2 | 730.22 | 884.69 | LOC100512721 |
| gene10995 | 1.10983644 | 0.001455301 | 1.02 | 0.72 | 1.62 | 0.44 | 0.49 | 0.72 | 0.45 | 0.88 | 0.62 | 2.05 | 0.85 | 1.31 | LOC100154661 |
| gene11010 | 2.20226678 | 0.000551445 | 2.92 | 0.43 | 0 | 0.35 | 0.11 | 0.29 | 0.32 | 1.85 | 0 | 0.94 | 1.41 | 0.14 | LOC100156127 |
| gene11113 | -2.524904939 | 0.001088723 | 0 | 0.05 | 0.11 | 0.66 | 0.1 | 0.31 | 0 | 0.05 | 0.15 | 0.07 | 0 | 0 | GPX5 |
| gene11129 | -1.042720294 | 0.001979279 | 2.76 | 1.79 | 1.91 | 4.06 | 4.13 | 6.03 | 4.65 | 4.32 | 2.49 | 2.18 | 2.87 | 7.3 | SLA-5 |
| gene1113 | -1.665294019 | 1.61E-05 | 0.3 | 0.13 | 0.23 | 0.99 | 0.5 | 0.66 | 0.19 | 0.19 | 0.39 | 0.14 | 0.2 | 0.44 | CILP |
| gene11189 | 2.884283169 | 0.009931868 | 0.41 | 0.02 | 0 | 0 | 0 | 0.05 | 0 | 0 | 0.07 | 0 | 0 | 0 |  |
| gene11255 | 1.643011256 | 1.41E-07 | 162.88 | 571.14 | 355.48 | 176.26 | 78.32 | 110.85 | 160.69 | 375.75 | 385.05 | 297.79 | 465.03 | 445.5 | LOC100154770 |
| gene11259 | 1.301360198 | 0.001946227 | 10.11 | 2.15 | 2.55 | 1.32 | 2.76 | 2.47 | 3.86 | 0.75 | 1.4 | 1.47 | 2.56 | 3.07 | LOC100520390 |
| gene11327 | 2.903632855 | 0.009931868 | 0.08 | 0.09 | 0.03 | 0.02 | 0 | 0 | 0 | 0.02 | 0.03 | 0 | 0.09 | 0 | LOC100513868 |
| gene11335 | 2.339291942 | 0.007905898 | 0.19 | 0.06 | 0.7 | 0 | 0.11 | 0 | 0 | 0.4 | 0.08 | 0.91 | 0.53 | 0 | LOC100519552 |
| gene11338 | -2.817922216 | 6.88E-17 | 5.91 | 2.54 | 3.56 | 41.08 | 40.14 | 7.59 | 10.76 | 20.76 | 4.29 | 7.77 | 5.52 | 10.07 | SLA-DRB1 |
| gene11347 | -1.407942956 | 0.000248823 | 0.87 | 0.75 | 1.45 | 4.77 | 1.89 | 1.58 | 1.17 | 3.31 | 2.07 | 1.76 | 2 | 2.48 | PSMB8 |
| gene11349 | -1.422128435 | 0.007855034 | 0.17 | 0.32 | 0.08 | 0.81 | 0.53 | 0.5 | 0.69 | 0.48 | 0.1 | 0.19 | 0 | 0.33 | LOC100520085 |
| gene1137 | 1.090530035 | 0.000747144 | 38.88 | 35.33 | 34.86 | 20.05 | 14.78 | 19.84 | 38.86 | 20.43 | 52.23 | 22.37 | 25.43 | 25.66 | SMAD3 |
| gene11386 | 1.827954935 | 1.09E-08 | 63.15 | 23.34 | 48.95 | 14.9 | 14.17 | 11.7 | 71.62 | 31.5 | 32.61 | 35.67 | 42.77 | 32.42 | LOC100522554 |
| gene11392 | -1.093963369 | 0.003150879 | 0.35 | 0.29 | 0.4 | 0.71 | 0.47 | 1.15 | 0.42 | 0.77 | 0.39 | 1.48 | 0.6 | 0.3 | COL21A1 |
| gene11424 | -1.882052483 | 1.85E-05 | 0.4 | 0.23 | 0.54 | 0.48 | 1.62 | 2.12 | 1.52 | 0.24 | 0.07 | 0.31 | 0.75 | 0.05 | SPDEF |
| gene11459 | -1.276616239 | 6.33E-05 | 11.4 | 14.42 | 17.79 | 29.49 | 56.95 | 23.13 | 28.28 | 27.7 | 26.57 | 21.35 | 24.25 | 55.21 | LOC100523258 |
| gene11488 | -1.563721426 | 9.42E-05 | 0.82 | 0.5 | 0.73 | 2.05 | 3.58 | 0.24 | 3.8 | 1.48 | 5.28 | 1.85 | 1.67 | 3.36 | LOC100152855 |
| gene11496 | 1.733881114 | 2.18E-07 | 3.87 | 7.54 | 5.64 | 1.57 | 0.92 | 3.01 | 1.27 | 1.6 | 2.32 | 3.27 | 3.09 | 6.32 |  |
| gene11497 | 1.611341605 | 1.27E-06 | 9.12 | 12.26 | 17.48 | 3.64 | 3.88 | 5.91 | 5.58 | 3.8 | 4.08 | 10.27 | 9.1 | 10.63 | LOC100155551 |
| gene11500 | 1.107910996 | 0.001366385 | 3.88 | 3.3 | 3.58 | 1.91 | 1.66 | 1.76 | 2.49 | 0.46 | 2.33 | 2.1 | 1.4 | 2.06 | NFYA |
| gene11503 | -1.284439153 | 0.000718315 | 0.3 | 1.64 | 0.95 | 1.96 | 0.76 | 5.41 | 1 | 1.94 | 1.15 | 1.16 | 0.85 | 1.19 | LOC100737974 |
| gene11526 | -2.16915104 | 8.63E-05 | 0.52 | 0.08 | 0.43 | 0.92 | 1.66 | 1.61 | 0.42 | 0.25 | 1.07 | 0.53 | 0.1 | 0.66 | LOC100739200 |
| gene11580 | 1.576025291 | 0.002647641 | 1.97 | 0.75 | 3.61 | 0.81 | 1.03 | 0.21 | 4.38 | 0.48 | 0.07 | 0.97 | 0.67 | 1.96 | LOC100622190 |
| gene11607 | 1.488569262 | 1.73E-06 | 81.8 | 114.51 | 95.74 | 38.33 | 19.96 | 52.55 | 7.45 | 44.95 | 28.13 | 52.91 | 52.88 | 56.19 | PLA2G7 |
| gene11611 | 1.280924599 | 6.88E-05 | 2.52 | 7.2 | 2.46 | 1.22 | 3.17 | 0.86 | 1.35 | 3.18 | 0.27 | 1.81 | 1.38 | 1.14 | GPR110 |
| gene11624 | 1.715713371 | 0.001114045 | 1.02 | 1.44 | 0.2 | 0 | 0.77 | 0.07 | 0.76 | 0.98 | 1.43 | 0.67 | 0.41 | 4.36 | CRISP3 |
| gene11636 | -2.831462454 | 0.000131923 | 0.2 | 0.18 | 0 | 0.81 | 0.63 | 1.72 | 1.59 | 3.18 | 0.35 | 0.81 | 1.26 | 0.57 | LOC100628022 |
| gene11724 | -1.611633665 | 0.001769008 |  |  |  |  |  |  |  |  |  |  |  |  |  |
| gene11769 | -1.530007216 | 0.000222588 | 1.32 | 1.3 | 1.24 | 2.89 | 4.7 | 4.07 | 1.4 | 5.39 | 3.87 | 3.68 | 1.57 | 1.5 | LOC100516621 |
| gene11818 | -1.244042578 | 0.000100405 | 1.94 | 2.56 | 1.5 | 1.4 | 11.07 | 2.37 | 1.16 | 0.37 | 1.97 | 1.43 | 0.58 | 1.51 | ISLR2 |
| gene11917 | -1.899042045 | 0.000768113 | 0.25 | 0.24 | 0 | 0.42 | 0.65 | 1.32 | 0.36 | 0.66 | 0.43 | 0.4 | 0.87 | 0.49 | CBLN3 |
| gene12022 | 1.314404749 | 2.25E-05 | 179.16 | 96.88 | 74.71 | 41.41 | 40.04 | 71.89 | 77.49 | 147.63 | 93.52 | 90.06 | 197.08 | 153.48 | PNP |
| gene12024 | 1.052156203 | 0.000670336 | 46.45 | 85.28 | 56.82 | 28.2 | 23.75 | 44.5 | 37.18 | 53.33 | 43.56 | 55.43 | 49.3 | 45.09 | APEX1 |
| gene12182 | -1.644679771 | 0.001442926 | 0.17 | 0.16 | 0.31 | 0.59 | 0.42 | 1.07 | 0.32 | 0.46 | 0.15 | 0.33 | 0.27 | 0.19 | ST8SIA2 |
| gene12185 | -1.801554226 | 0.000184489 | 0.13 | 0.12 | 0.4 | 0.57 | 1.28 | 0.22 | 0.8 | 1.6 | 1.16 | 1.65 | 1.24 | 1.67 |  |
| gene12237 | -1.410602857 | 8.52E-06 | 1.18 | 4.17 | 4.24 | 10.48 | 7.38 | 8.68 | 3.54 | 0.96 | 4.08 | 1.84 | 1.19 | 2.02 | SMOC1 |
| gene12268 | 1.29015701 | 3.49E-05 | 28.81 | 22.97 | 45.28 | 21.18 | 10.81 | 9.67 | 16.09 | 14.28 | 11.22 | 30.28 | 12.15 | 8.67 | ACOT4 |
| gene12300 | 1.152190868 | 0.000184138 | 162.82 | 207.56 | 197.92 | 52.67 | 138.1 | 76.88 | 316.59 | 251.38 | 128.23 | 251.07 | 277.55 | 568.05 | FOS |
| gene12312 | -1.478133363 | 8.68E-05 | 0.75 | 0.46 | 0.88 | 1.45 | 3.16 | 1.16 | 1.18 | 0.35 | 0.49 | 0.68 | 0.64 | 0.6 | ESRRB |
| gene12388 | 1.091953903 | 0.001713401 | 0.78 | 1.19 | 1.76 | 0.21 | 0.25 | 1.42 | 4.87 | 7.42 | 2.78 | 2.09 | 4.04 | 3.73 | LOC100512175 |
| gene12400 | 1.029584941 | 0.000812567 | 146.69 | 97.02 | 146.99 | 39.52 | 70.63 | 93.58 | 5.32 | 37.84 | 42.4 | 57.82 | 86.93 | 43.53 | CHGA |
| gene12428 | -2.084964913 | 5.28E-05 | 0.1 | 0.12 | 0.31 | 0.27 | 0.15 | 2.01 | 3.38 | 0.1 | 0.17 | 0.33 | 0.63 | 0.43 | SERPINA11 |
| gene12429 | -1.34217138 | 1.39E-05 | 183.69 | 965.76 | 2014 | 1365.4 | 2375 | 4515.9 | 2505.8 | 138.43 | 226 | 225.45 | 486.07 | 174.87 | UFBP |
| gene12435 | 2.70176612 | 1.19E-13 | 7.59 | 0.05 | 10.34 | 0.22 | 1.73 | 0.96 | 4.26 | 1.6 | 1.23 | 4.27 | 0.99 | 0.9 | SERPINA3-1 |
| gene12436 | -5.037578245 | 3.31E-22 | 0.12 | 0.02 | 0.08 | 2.28 | 3.53 | 1.16 | 1.05 | 4.16 | 0.47 | 10.56 | 2.63 | 1.66 | LOC100153899 |
| gene12438 | -2.370423495 | 0.001326447 | 0.12 | 0.04 | 0.16 | 0.52 | 0 | 0.82 | 0.07 | 0.13 | 0 | 1.32 | 0 | 0 |  |
| gene12439 | -1.21012157 | 0.002869761 | 0.89 | 0.04 | 1.48 | 1.48 | 2.34 | 1.47 | 18.12 | 3.53 | 2.95 | 17.14 | 3.41 | 13.3 | LOC100156325 |
| gene12440 | 2.068411427 | 6.26E-11 | 112.78 | 183.88 | 162.31 | 50.09 | 53.83 | 9.89 | 192.07 | 82.21 | 80.6 | 216.34 | 99.74 | 146.15 | SERPINA3-2 |
| gene12461 | -1.231357901 | 0.006840744 | 0.06 | 0.16 | 0.04 | 0.25 | 0.18 | 0.31 | 0.46 | 0.19 | 0.35 | 0.3 | 0.22 | 0.24 | BCL11B |
| gene12474 | 1.426145665 | 6.12E-06 | 24.43 | 17.44 | 20.35 | 7.31 | 11.8 | 5.35 | 5.45 | 4.07 | 27.05 | 19.61 | 16.48 | 34.31 | LOC100513744 |
| gene12492 | 1.165195768 | 0.000151915 | 3010.7 | 1159.6 | 1963.1 | 952.88 | 1125.9 | 852.51 | 746.79 | 1220.4 | 1385.7 | 2754.7 | 1467.2 | 1405 | HSP90AA1 |
| gene12538 | -1.204331475 | 0.002144717 | 0.37 | 0.35 | 1.34 | 1.93 | 0.53 | 2.37 | 0.94 | 0.09 | 0 | 0.27 | 0.14 | 0.25 | LOC100624633 |
| gene12619 | 1.365952585 | 0.000193232 | 7.09 | 1.07 | 1.95 | 2.36 | 1.23 | 0.63 | 0.45 | 0.71 | 2.17 | 1.08 | 0.45 | 2.64 | GSTA2 |
| gene12672 | 1.146609468 | 0.007601856 | 0.96 | 1.79 | 1.16 | 0.27 | 0.84 | 0.79 | 0.52 | 0.96 | 0.72 | 1.79 | 1.16 | 1.11 | LOC100737053 |
| gene12674 | -5.360689116 | 0.000292606 |  |  |  |  |  |  |  |  |  |  |  |  |  |
| gene12686 | -1.201079401 | 0.000174731 | 3.79 | 3.24 | 3.41 | 11.15 | 3.56 | 11.01 | 7.33 | 8.09 | 1.31 | 3.99 | 6.8 | 2.39 | LOC100521979 |
| gene12753 | -1.236122535 | 0.000179514 | 2.96 | 1.54 | 2.63 | 5.88 | 7.69 | 3.93 | 5.29 | 11.17 | 3.8 | 4.47 | 2.52 | 3.52 | LOC100737183 |
| gene12781 | -1.617461146 | 0.000207878 | 0.19 | 0.11 | 0.33 | 0.5 | 0.23 | 1.13 | 0.52 | 0.35 | 0.06 | 0.79 | 0.56 | 0.29 | LOC100521908 |
| gene1279 | 1.451003963 | 6.00E-05 | 3.22 | 3.42 | 3.19 | 2.18 | 0.92 | 0.79 | 1.32 | 4.84 | 3.36 | 3.68 | 2.09 | 4.3 | LOC100513515 |
| gene12799 | -1.321792051 | 0.000646368 | 0.76 | 0.69 | 1.07 | 2.31 | 0.83 | 3.48 | 1 | 1.39 | 0.63 | 0.92 | 0.19 | 0.23 | LOC100525349 |
| gene12829 | 1.498001139 | 0.000451642 | 0.75 | 0.83 | 0.86 | 0.03 | 0.46 | 0.44 | 0.98 | 0.76 | 0.08 | 0.15 | 0.93 | 0.88 | BEND4 |
| gene12844 | -2.776976685 | 0.003477837 | 0 | 0.03 | 0 | 0.16 | 0.05 | 0.13 | 0 | 0.05 | 0 | 0.11 | 0.04 | 0.03 | LOC100622712 |
| gene12920 | -3.429265963 | 1.38E-06 | 0 | 0.06 | 0 | 0.47 | 0.29 | 0.27 | 0.15 | 0.1 | 0.11 | 0.15 | 0.1 | 0.18 | TDO |
| gene12925 | 1.239202581 | 0.00836245 | 1.01 | 0.84 | 1.11 | 0.29 | 0.56 | 0.42 | 0.67 | 1.25 | 1.12 | 0.88 | 0.39 | 1.4 | GLRB |
| gene12928 | -1.803781558 | 1.85E-05 | 2.48 | 1.68 | 0.41 | 4.52 | 9.21 | 3.48 | 2.82 | 5.5 | 1.75 | 1.54 | 1.97 | 2.33 | LOC100516145 |
| gene12948 | -1.047647281 | 0.00310429 | 2.2 | 2.82 | 3.02 | 7.37 | 5.78 | 3.98 | 5.24 | 5.36 | 3.15 | 6.42 | 3.23 | 3.78 | LOC100518849 |
| gene12974 | 2.385586245 | 5.74E-13 | 3.7 | 114.41 | 2.12 | 1.88 | 13.05 | 9.33 | 2.22 | 2.05 | 2.29 | 5.87 | 7.22 | 4.78 | LOC100523263 |
| gene12980 | 1.602899419 | 0.005840954 | 0.14 | 0.29 | 0.71 | 0.07 | 0.13 | 0.16 | 0.2 | 0.44 | 0.39 | 0.09 | 0.27 | 0.36 | LOC100523077 |
| gene1299 | -1.65313778 | 0.00084307 | 0.03 | 0.12 | 0.16 | 0.2 | 0.21 | 0.64 | 0.34 | 0.23 | 0.26 | 0.28 | 0.32 | 0.15 | LOC100622535 |
| gene12991 | 2.395460104 | 0.004566391 | 0.3 | 0.36 | 0.33 | 0 | 0.11 | 0.07 | 0.16 | 0.16 | 0.43 | 0.16 | 0.08 | 0.35 | LOC100525236 |
| gene13008 | 2.394236305 | 1.32E-10 | 3.19 | 0.93 | 3.67 | 0.87 | 0.3 | 0.38 | 0.35 | 0.93 | 1.47 | 0.08 | 0.99 | 0.27 | LOC100513296 |
| gene13012 | -2.46204817 | 0.000766471 | 0.03 | 0.07 | 0 | 0.58 | 0 | 0.04 | 0.04 | 0 | 0 | 0.09 | 0.04 | 0 | TMPRSS11B |
| gene13041 | 1.742707695 | 1.27E-06 | 9.5 | 17.03 | 11.97 | 2.49 | 3.31 | 6.85 | 9.44 | 7.99 | 15.32 | 18.31 | 11.25 | 12.33 | LOC100739482 |
| gene13042 | 1.519898287 | 2.80E-06 | 3.21 | 5.5 | 3.43 | 0.79 | 1.34 | 2.41 | 2.4 | 3.93 | 2.43 | 6.57 | 3.05 | 3.41 | SLC4A4 |
| gene13043 | -3.975330834 | 2.78E-18 | 0.18 | 0.2 | 0.05 | 2.53 | 2.08 | 3.51 | 0.69 | 0.04 | 0 | 0 | 0 | 0.1 | LOC100739516 |
| gene13044 | -2.212106606 | 1.26E-08 | 0.5 | 0.31 | 0.6 | 3.47 | 1.99 | 1.04 | 1.1 | 1.77 | 0.7 | 2.43 | 0.5 | 0.61 | GC |
| gene13050 | -5.725833643 | 1.39E-43 | 0.1 | 0.23 | 0.25 | 0.88 | 6.12 | 26.56 | 0.27 | 0.07 | 0.11 | 84.56 | 0.13 | 0.27 | ALB |
| gene13061 | 1.647234532 | 1.36E-06 | 14.09 | 24.59 | 14.96 | 5.46 | 7.6 | 5.07 | 11.26 | 29.8 | 10.03 | 14.39 | 25.5 | 19.44 | CXCL2 |
| gene13064 | 1.646514727 | 6.91E-07 | 12.44 | 8.77 | 14.03 | 4.1 | 3.83 | 4.01 | 6.35 | 5.28 | 8.29 | 9.21 | 7.31 | 10.53 | AREG |
| gene13081 | -3.149942898 | 1.08E-07 | 0 | 1.27 | 0 | 13.62 | 1.03 | 0.43 | 1.67 | 5.58 | 5.69 | 4.85 | 4.7 | 2.14 | CXCL10 |
| gene13088 | -2.134451844 | 0.003475872 | 0 | 0.05 | 0.11 | 0.32 | 0.16 | 0.24 | 0.02 | 0 | 0 | 0.13 | 0 | 0.08 | LOC100512296 |
| gene13112 | -2.131373628 | 2.38E-08 | 0.21 | 0.32 | 0.85 | 2.1 | 1.03 | 2.8 | 5.59 | 0.04 | 0.08 | 12.99 | 0.11 | 0.24 | LOC100626178 |
| gene13113 | -2.884923626 | 0.002067442 | 0 | 0.04 | 0 | 0.18 | 0.17 | 0.12 | 0.81 | 0 | 0 | 0.62 | 0.09 | 0 | FGB |
| gene13144 | 1.342852266 | 0.008270607 | 3.57 | 2.67 | 2.45 | 0.85 | 1.65 | 1.11 | 4.05 | 2.8 | 3.46 | 1.45 | 2.53 | 3.59 | LOC100620683 |
| gene13193 | -1.228718902 | 0.004045014 | 0.33 | 0.47 | 0.19 | 0.22 | 0.25 | 2.22 | 0.15 | 0.08 | 0.38 | 0.24 | 0.12 | 0.26 | LOC100512242 |
| gene13220 | 1.034231652 | 0.008083686 | 1.04 | 0.14 | 0.35 | 0.28 | 0.19 | 0.34 | 0.48 | 0.46 | 0.26 | 0.11 | 0.33 | 0.11 | LOC100620123 |
| gene13222 | -1.011155334 | 0.006630788 | 0.79 | 2.12 | 2.11 | 2.72 | 3.19 | 4.69 | 2.23 | 3.04 | 3.58 | 0.86 | 3.4 | 1.96 | LOC100515958 |
| gene13263 | -3.276229772 | 0.001681609 | 0 | 0.02 | 0 | 0.13 | 0.18 | 0.16 | 0.03 | 0.23 | 0 | 0 | 0 | 0.15 | QRFPR |
| gene13265 | 1.997275095 | 0.002077547 | 0.28 | 0.46 | 0.69 | 0.11 | 0.05 | 0.21 | 0 | 0.55 | 0.08 | 0.31 | 1.19 | 0.54 | LOC100738704 |
| gene13303 | -2.356094498 | 1.86E-08 | 0.22 | 0.53 | 0.23 | 1.73 | 2.99 | 0.94 | 1.56 | 2.13 | 0.61 | 1.13 | 0.34 | 0.56 | LOC100513102 |
| gene13327 | -2.496108563 | 8.01E-15 | 67.96 | 11.13 | 82.67 | 222.25 | 554.77 | 178.11 | 374.38 | 44.68 | 29.8 | 15.03 | 24.01 | 27.48 | LOC100519324 |
| gene1335 | -3.088810784 | 4.37E-19 | 1.96 | 0.63 | 1.94 | 7.21 | 26.26 | 5.59 | 12.12 | 2.63 | 1.33 | 0.62 | 1.05 | 0.58 | ESR2 |
| gene13371 | -1.237559553 | 6.12E-05 | 83.12 | 45.02 | 52.58 | 131.45 | 118.39 | 209.71 | 188.97 | 50.35 | 19.57 | 51.25 | 52.39 | 38.17 | LOC100524695 |
| gene13376 | -1.290592128 | 0.000207022 | 4.94 | 5.85 | 3.47 | 5.05 | 18.18 | 13.86 | 10.16 | 3.88 | 1.92 | 5.8 | 2.6 | 4.33 | LOC100628129 |
| gene13379 | -1.764354605 | 1.14E-07 | 15.08 | 18.59 | 9.91 | 8.78 | 35.55 | 118.16 | 17.31 | 4.11 | 1.38 | 4.27 | 5.4 | 1.88 | LOC100620154 |
| gene13392 | -1.200005245 | 0.001008796 | 0.86 | 1.57 | 0.75 | 1.46 | 4.83 | 1.57 | 1.55 | 1.98 | 0.41 | 2.04 | 0.67 | 1.06 | ADH1C |
| gene13393 | -1.595759065 | 0.000217771 | 0.62 | 0.24 | 0.13 | 1.47 | 0.88 | 0.86 | 0.21 | 1.63 | 0.52 | 0.54 | 0.63 | 0.33 | ADH4 |
| gene13412 | -2.676831975 | 4.67E-13 |  |  |  |  |  |  |  |  |  |  |  |  |  |
| gene13424 | 1.020683263 | 0.000891125 | 123.12 | 135.04 | 121.87 | 54.26 | 84.44 | 58.91 | 64.13 | 70.53 | 145.9 | 121.79 | 129 | 228.49 | LOC100517722 |
| gene13430 | -1.070755094 | 0.005704497 | 1.54 | 0.7 | 0.57 | 2.75 | 2.11 | 1.29 | 2.59 | 1.65 | 1.42 | 1.4 | 1.4 | 0.61 | LOC100519855 |
| gene13440 | -1.052070461 | 0.000624448 | 40.02 | 40.98 | 52.56 | 66.38 | 116.01 | 109.13 | 77.88 | 85.29 | 92.38 | 129.24 | 109.14 | 122.84 | SPARCL1 |
| gene13442 | 1.002061892 | 0.001207123 | 52.15 | 49.41 | 97.66 | 27.36 | 31.21 | 45.97 | 16.46 | 36.36 | 45.16 | 34.23 | 68.62 | 79.49 | LOC100520923 |
| gene13488 | -1.207756595 | 0.00074705 | 0.45 | 1.02 | 0.74 | 2.63 | 0.64 | 2.18 | 0.64 | 0.48 | 0.69 | 0.75 | 0.42 | 0.19 | BMP3 |
| gene13518 | -1.058993933 | 0.002654788 | 0.37 | 0.5 | 0.59 | 0.71 | 0.79 | 1.69 | 0.71 | 0.45 | 0.86 | 0.87 | 0.8 | 0.67 | SCUBE2 |
| gene13533 | -1.092377372 | 0.005579862 | 0.25 | 0.55 | 0.46 | 0.92 | 1.26 | 0.61 | 0.34 | 0.13 | 0.26 | 0.16 | 0.31 | 0.64 | TUB |
| gene13565 | -1.924799224 | 0.002375721 | 0.42 | 0.25 | 0.89 | 1.53 | 1.59 | 2.41 | 1.15 | 0.93 | 0.73 | 0 | 1.31 | 0.25 | LOC100622619 |
| gene13567 | -1.496832491 | 8.69E-05 | 0.67 | 0.19 | 1.01 | 1.29 | 1.56 | 2.4 | 1.16 | 0.6 | 0.21 | 0.7 | 0.75 | 1.17 | LOC100622122 |
| gene13568 | -1.055701473 | 0.005424771 | 0.26 | 0.49 | 0.79 | 0.85 | 1.33 | 1.05 | 1.52 | 0.84 | 1.3 | 1.37 | 1.68 | 0.97 | SYT9 |
| gene13573 | -1.518365945 | 0.004488427 | 0.06 | 0.17 | 0.28 | 0.41 | 0.35 | 0.6 | 0.09 | 0.09 | 0.06 | 0.05 | 0.05 | 0.08 |  |
| gene13657 | 1.354970457 | 0.006840744 | 0.79 | 0.22 | 1.05 | 0.56 | 0.1 | 0.17 | 0.58 | 1.06 | 0 | 1.49 | 0.77 | 0.22 | LOC100525239 |
| gene13736 | 1.035988977 | 0.000790893 | 249.55 | 52.11 | 111.64 | 56.85 | 78.49 | 83.7 | 26.6 | 55.43 | 45.12 | 189.31 | 50 | 167.55 |  |
| gene13737 | 1.576853211 | 4.38E-07 | 459.4 | 95.55 | 184.59 | 62.5 | 102.31 | 105.15 | 52.48 | 68.14 | 72.25 | 229.15 | 65.27 | 220.07 | HBB |
| gene13820 | -1.626893907 | 0.007589559 |  |  |  |  |  |  |  |  |  |  |  |  |  |
| gene13839 | -1.097187785 | 0.000554453 | 8.16 | 2.56 | 18.75 | 10.26 | 36.5 | 17.7 | 4.79 | 3.27 | 3.37 | 6.96 | 2.28 | 3.15 | LOC100513029 |
| gene13840 | -1.345977854 | 1.88E-05 | 12.68 | 2.37 | 29.43 | 24.58 | 58.41 | 33.24 | 5.4 | 4.8 | 2.96 | 3.84 | 1.26 | 3.25 | P2RY6 |
| gene13934 | 1.645164441 | 0.002780863 | 1.44 | 3.38 | 0.15 | 0.91 | 0.38 | 0.48 | 0.16 | 1.21 | 0.39 | 0.36 | 0.7 | 0.48 | LOC100625887 |
| gene14013 | 2.296305369 | 1.05E-11 | 4.78 | 6.23 | 14.38 | 0.99 | 0 | 4.57 | 0.02 | 0.93 | 0.08 | 0.68 | 1.06 | 1.37 | LOC100513220 |
| gene14016 | 1.273992123 | 4.58E-05 | 70.31 | 65.19 | 105.7 | 35.05 | 42.95 | 26.31 | 26.22 | 12.74 | 23.52 | 12.31 | 12.7 | 17.04 | LOC100517177 |
| gene14018 | -1.31542874 | 0.000840952 | 0.35 | 0.23 | 0.2 | 0.47 | 1.36 | 0.18 | 1.96 | 0.66 | 1.04 | 0.55 | 0.65 | 0.49 | PIWIL4 |
| gene14040 | 1.567116785 | 0.005868841 | 1.24 | 1.03 | 0.86 | 0.52 | 0.41 | 0.13 | 1.62 | 1.51 | 0.77 | 1.59 | 0.46 | 1.61 |  |
| gene14041 | -1.013320252 | 0.003364667 | 0.58 | 0.39 | 1.06 | 0.97 | 1.78 | 1.44 | 0.97 | 1.08 | 1.46 | 1.06 | 0.95 | 1.14 | CNTN5 |
| gene14055 | 1.204295379 | 0.000106282 | 80.42 | 110.76 | 182.76 | 125.77 | 5.25 | 39.71 | 64.66 | 497.41 | 107.98 | 187.66 | 154.47 | 70.24 | MMP7 |
| gene14057 | 1.6040692 | 0.001220734 | 0.45 | 0.41 | 0.66 | 0.09 | 0.11 | 0.34 | 0.1 | 0.39 | 0 | 0.16 | 0.12 | 0.43 | MMP27 |
| gene14059 | 2.171951169 | 1.06E-11 | 13.13 | 92.52 | 25.83 | 16.66 | 6.5 | 7.42 | 4.69 | 24.53 | 15.24 | 48.25 | 29.89 | 27.74 | MMP1 |
| gene14075 | -1.033833677 | 0.003385398 | 1.35 | 1.12 | 1.32 | 4.18 | 1.11 | 2.97 | 0.94 | 1.86 | 0.74 | 1.23 | 1.2 | 0.81 | CASP1 |
| gene14102 | 2.004184394 | 0.00033075 |  |  |  |  |  |  |  |  |  |  |  |  |  |
| gene14153 | -3.006309694 | 9.91E-08 | 0.21 | 0.19 | 0.69 | 2.4 | 0.12 | 5.54 | 0.17 | 0.11 | 0.55 | 0.34 | 0.49 | 0.15 | NNMT |
| gene14164 | -2.503789965 | 6.01E-05 | 0.03 | 0.11 | 0.05 | 0.81 | 0.24 | 0.3 | 0.14 | 0.11 | 0.24 | 2.2 | 0.11 | 0 | APOA5 |
| gene14165 | -1.303409519 | 0.001671727 | 0.23 | 0.5 | 0.31 | 0.74 | 0.11 | 2.14 | 0.66 | 0.03 | 0.06 | 4.56 | 0.1 | 1.04 | APOA4 |
| gene14166 | -3.325102552 | 0.000483434 | 0 | 0 | 0.45 | 0.43 | 0.85 | 2.24 | 0.07 | 0.54 | 0 | 10.19 | 0 | 0.75 | APOC3 |
| gene14229 | 1.026050859 | 0.005475954 | 1.54 | 1.48 | 1.27 | 0.56 | 0.52 | 1.22 | 0.97 | 0.36 | 0.71 | 0.28 | 0.83 | 1.67 | PVRL1 |
| gene14236 | -1.457651082 | 0.00162692 | 0.2 | 0.13 | 0.17 | 0.47 | 0.32 | 0.66 | 0.1 | 0.4 | 0.29 | 0.27 | 0.23 | 0.21 | LOC100523504 |
| gene14249 | 1.102818912 | 0.00033464 | 290.07 | 189.51 | 183.17 | 92.01 | 139.11 | 98.21 | 58.86 | 67.37 | 127.57 | 245.36 | 103.45 | 92.71 | HSPA8 |
| gene14293 | -1.068062442 | 0.000538031 | 7.3 | 18.82 | 13.77 | 24.61 | 35.54 | 27.08 | 36.38 | 17.8 | 41.52 | 21.87 | 13.8 | 28.51 | VWA5A |
| gene14303 | -2.359100325 | 0.007905898 | 0 | 0.05 | 0.05 | 0.58 | 0 | 0 | 0.06 | 0.08 | 0.2 | 0.06 | 0.12 | 0 | LOC100515044 |
| gene14323 | -1.906051273 | 0.003069733 | 0 | 0.19 | 0 | 0.58 | 0.37 | 0.03 | 0.07 | 0.45 | 0.35 | 0.16 | 0 | 0 | LOC100523913 |
| gene14327 | -1.970359932 | 0.001387593 | 0.03 | 0.14 | 0 | 0.33 | 0.38 | 0.14 | 0.1 | 0.17 | 0.12 | 0.32 | 0 | 0.14 | LOC100524268 |
| gene14328 | -1.157784046 | 0.005379382 |  |  |  |  |  |  |  |  |  |  |  |  |  |
| gene14369 | -2.220025778 | 3.34E-11 | 1.74 | 0.36 | 2.24 | 7.58 | 9.72 | 3.32 | 6.12 | 0.99 | 2.05 | 2.23 | 1.2 | 1.31 | KIRREL3 |
| gene14373 | 2.433400929 | 2.10E-13 | 15.97 | 38.16 | 5.4 | 3.86 | 1.43 | 6.72 | 2.31 | 44.68 | 22.04 | 43.52 | 26.55 | 56.23 | KCNJ1 |
| gene14408 | 3.219969802 | 0.002067442 | 0.18 | 0.09 | 0 | 0 | 0 | 0.03 | 0.06 | 0.04 | 0 | 0.19 | 0 | 0.06 | GLB1L3 |
| gene14417 | -1.100000357 | 0.000482336 | 5.25 | 3.05 | 3.48 | 12.11 | 9.15 | 5.61 | 4.85 | 26 | 6.51 | 8.1 | 5.11 | 6.32 | GUCY1A2 |
| gene14427 | -1.321687902 | 2.06E-05 | 22.86 | 28.66 | 31.82 | 67.66 | 78.78 | 72.56 | 39.31 | 21.54 | 25.75 | 53.2 | 32.07 | 40.26 | LOC100526237 |
| gene14459 | -1.020947464 | 0.001533071 | 2.72 | 2.7 | 2.77 | 3.2 | 9.45 | 4.77 | 2.55 | 4.51 | 2.48 | 3.9 | 3.3 | 2.2 | MFSD4 |
| gene14470 | 1.452439383 | 8.68E-05 | 1.79 | 8.2 | 3.92 | 1.91 | 2.23 | 1.3 | 5.34 | 2.71 | 3.96 | 2.34 | 3.27 | 0.99 | LOC100518270 |
| gene14487 | -1.379815458 | 0.004308967 | 0.18 | 0.12 | 0.11 | 0.05 | 0.18 | 0.98 | 0.35 | 0.04 | 0 | 0.16 | 0.06 | 0.03 | PIGR |
| gene14489 | -1.822997688 | 0.000555871 | 0.1 | 0.08 | 0.24 | 0.13 | 0.13 | 1.16 | 0.6 | 0.17 | 0.15 | 0.07 | 0.01 | 0.11 | LOC100522888 |
| gene14493 | 1.789631878 | 3.99E-08 | 26.83 | 7.8 | 8.6 | 2.13 | 3.21 | 8.53 | 9.42 | 10.05 | 11.54 | 8.08 | 22.5 | 25.83 | YOD1 |
| gene14510 | 1.531452404 | 8.44E-05 | 1.31 | 2.24 | 2.08 | 0.83 | 0.52 | 0.7 | 0.63 | 2.5 | 1.37 | 2.31 | 0.88 | 0.75 | STEAP1 |
| gene14511 | 2.144224422 | 0.000441185 | 0.2 | 0.61 | 0.27 | 0.12 | 0.06 | 0.15 | 0.3 | 0.23 | 0 | 0.53 | 0 | 0.34 | LOC100623287 |
| gene14515 | 2.258686351 | 4.84E-11 | 11.43 | 27.93 | 12.88 | 3.38 | 3.92 | 4.3 | 2.48 | 44.91 | 13.77 | 25.22 | 5.3 | 13.1 | LOC100518208 |
| gene14538 | 1.475728347 | 6.88E-05 | 13.52 | 10.18 | 9.81 | 4.63 | 1.99 | 6.7 | 12.36 | 5.96 | 8.48 | 3.91 | 9.82 | 7.38 | LOC100627476 |
| gene14571 | 1.549267563 | 0.000658586 | 2.06 | 2.83 | 2.52 | 1.47 | 0.44 | 0.7 | 0.7 | 0.1 | 1.18 | 4.5 | 1.98 | 3.37 | LOC100525179 |
| gene14572 | 1.054652785 | 0.000632842 | 18.46 | 55.36 | 56.34 | 13.55 | 37.61 | 13.14 | 32.3 | 20.99 | 35.91 | 42.45 | 32.42 | 25.85 | ASNS |
| gene14577 | -1.022509133 | 0.001260503 | 4.39 | 5.41 | 5.57 | 10.28 | 13.61 | 8.93 | 7.92 | 9.33 | 8.7 | 7.84 | 5.3 | 10.6 | GLCCI1 |
| gene14629 | 1.098748521 | 0.000488203 | 1.42 | 1.37 | 1.59 | 0.6 | 0.55 | 1.03 | 0.32 | 0.91 | 1.11 | 1.32 | 1.23 | 2.25 |  |
| gene14661 | -1.109220077 | 0.004784863 | 0.13 | 0.29 | 0.9 | 0.75 | 0.78 | 1.27 | 1.14 | 0.49 | 0.2 | 0.27 | 0.18 | 0.21 | GRM3 |
| gene14677 | -1.210625478 | 0.000125144 | 8.16 | 4.57 | 5.67 | 15.88 | 10.86 | 18.94 | 6.72 | 11.69 | 8.77 | 16.1 | 8.46 | 11.31 | CD36 |
| gene14691 | -1.368293852 | 0.000106511 | 1.41 | 1.14 | 4.23 | 4.82 | 3.25 | 9.57 | 2.05 | 2.85 | 2.59 | 2.23 | 2.32 | 1.4 | LOC100737774 |
| gene14768 | -2.512384 | 0.009931868 | 0 | 0.02 | 0 | 0.05 | 0.11 | 0.02 | 0.02 | 0 | 0 | 0 | 0.02 | 0.02 | LOC100737901 |
| gene14819 | -1.294365296 | 0.001963435 | 0.3 | 0.43 | 0.21 | 0.57 | 0.77 | 1.14 | 0.77 | 0.49 | 0.19 | 1.84 | 0.43 | 0.08 | SERPINC1 |
| gene14830 | -1.217530698 | 8.27E-05 | 4.26 | 13.93 | 8.27 | 13.64 | 33.14 | 17.04 | 12.93 | 0.97 | 3.45 | 4.32 | 2.71 | 4.1 | TNN |
| gene14837 | -1.314566905 | 0.003512644 | 0.53 | 1.02 | 0.24 | 0.37 | 2.28 | 2.45 | 0.5 | 0.83 | 0.78 | 1.61 | 1.79 | 2.82 | LOC100737260 |
| gene14840 | 4.83957665 | 0.0030599 |  |  |  |  |  |  |  |  |  |  |  |  |  |
| gene14851 | 1.02822114 | 0.002488739 | 2.91 | 4.7 | 6.47 | 2.21 | 2.3 | 2.82 | 4.33 | 5.08 | 11.59 | 8.41 | 7.89 | 10.67 | RALGPS2 |
| gene14858 | -1.397352503 | 0.000300383 | 0.1 | 0.79 | 0.42 | 0.76 | 1.36 | 1.76 | 0.88 | 1.39 | 0.92 | 7.58 | 0.52 | 0.43 | LOC100523986 |
| gene14859 | -1.905475181 | 0.003069852 | 0.04 | 0.02 | 0.04 | 0.16 | 0.09 | 0.14 | 0.08 | 0.02 | 0.04 | 0.11 | 0 | 0.03 | LOC100524163 |
| gene1490 | 1.537999221 | 0.001092942 | 0.23 | 0.6 | 0.29 | 0.1 | 0.15 | 0.17 | 0.05 | 0.4 | 0.26 | 0.7 | 0.21 | 0.17 | LOC100739045 |
| gene14921 | 2.042291364 | 9.85E-11 | 187.21 | 338.97 | 253.29 | 85.49 | 54.84 | 58.97 | 58.29 | 181.77 | 145.75 | 216.51 | 131.18 | 122.54 | PGHS-2 |
| gene14922 | 1.248818516 | 5.25E-05 | 75.39 | 133.36 | 79.17 | 36.76 | 46.93 | 44.1 | 17.67 | 72.98 | 65.41 | 76.12 | 58.72 | 87.6 | LOC100520687 |
| gene14971 | -1.492649107 | 0.005792782 | 0.22 | 0.16 | 0.04 | 0.68 | 0.11 | 0.65 | 0.16 | 0.6 | 0.28 | 0.37 | 0.1 | 0.05 | LOC100739636 |
| gene14990 | -1.907390451 | 8.13E-09 | 1.46 | 3.33 | 4.05 | 2.76 | 4.75 | 27.59 | 4.12 | 0.41 | 0.2 | 0.32 | 1.03 | 0.55 | LOC100515158 |
| gene14997 | -1.522634836 | 2.18E-05 | 0.86 | 1.37 | 0.28 | 1.38 | 2.42 | 4.5 | 5.29 | 2.26 | 0.89 | 0.62 | 1.53 | 2.16 | DDC |
| gene1500 | 1.555644457 | 8.27E-06 | 6 | 7.21 | 1.26 | 1.78 | 2.5 | 1.08 | 1.7 | 2.77 | 6.31 | 4.85 | 3.99 | 4.44 | CD274 |
| gene15018 | -1.746688847 | 7.80E-05 | 0.35 | 1.85 | 0.65 | 6.17 | 3.88 | 0.63 | 0.59 | 0.94 | 0 | 4.84 | 0.99 | 3.13 | LOC100628072 |
| gene15019 | -3.708730211 | 5.53E-06 | 0.09 | 0.15 | 0.32 | 0 | 1.96 | 5.75 | 0.39 | 0.46 | 0.43 | 1.19 | 1.53 | 1.04 | LOC100738451 |
| gene15024 | 1.195087065 | 0.000106003 | 683.87 | 154.43 | 294.81 | 123.03 | 219.93 | 193.89 | 348.76 | 379.47 | 216.1 | 481.51 | 425.6 | 415.63 | RGS2 |
| gene15025 | 1.428485218 | 2.04E-05 | 9.9 | 8.19 | 14.59 | 3.01 | 3.25 | 6.76 | 5.45 | 6.79 | 12.86 | 21.36 | 15.33 | 37.68 | RGS1 |
| gene15044 | 2.487035321 | 3.78E-12 | 24.92 | 6.34 | 4.1 | 3.11 | 1.34 | 2.73 | 5.99 | 11.05 | 4.04 | 4.54 | 2.67 | 4.83 | LOC100622556 |
| gene15066 | 1.460380334 | 3.76E-06 | 42.28 | 6.87 | 4.85 | 6.36 | 8.2 | 7.15 | 14.44 | 5.53 | 9.64 | 10.11 | 6.68 | 8.34 | DUSP10 |
| gene15075 | -1.731117964 | 5.89E-08 | 8.07 | 19.46 | 12.98 | 25 | 69.83 | 45.69 | 143.14 | 11.94 | 8.58 | 13.62 | 20.61 | 14.76 | LOC100518506 |
| gene15110 | 1.265718527 | 0.001195974 | 2.19 | 0.74 | 0.8 | 0.42 | 0.86 | 0.39 | 0.48 | 1.89 | 0.83 | 2.78 | 4.19 | 2.2 | LOC100522753 |
| gene15119 | 2.486048072 | 0.007905898 | 0 | 0.15 | 0.22 | 0 | 0.06 | 0 | 0.15 | 0.05 | 0.17 | 0.12 | 0.04 | 0.1 | LOC100739202 |
| gene1513 | 1.32048103 | 2.08E-05 | 36.88 | 85.47 | 52.61 | 23.24 | 32.9 | 16.95 | 30.12 | 29.02 | 40.31 | 78.72 | 48.29 | 78.52 | SLC1A1 |
| gene15133 | -1.524421969 | 6.20E-05 | 1.04 | 1.73 | 3.41 | 10.09 | 3.58 | 4.28 | 0.97 | 3.81 | 6.58 | 4.2 | 3.46 | 2.23 | LOC100514177 |
| gene15149 | -1.637484441 | 5.28E-07 | 1.67 | 1.81 | 5.68 | 14.57 | 6.63 | 7.85 | 2.52 | 11.61 | 5.07 | 3.66 | 3.38 | 2.55 | LOC100516995 |
| gene15157 | -1.389340037 | 0.000498329 |  |  |  |  |  |  |  |  |  |  |  |  |  |
| gene15165 | -5.367907277 | 0.000292606 | 0 | 0 | 0 | 1.41 | 2.08 | 1.3 | 1.45 | 0.63 | 1.58 | 0.98 | 0 | 0 | LOC100626028 |
| gene15197 | 1.035877483 | 0.001448361 | 1.75 | 2.07 | 1.66 | 0.78 | 1.03 | 1.05 | 0.94 | 0.85 | 0.95 | 1.13 | 1.26 | 1.62 | KIF21B |
| gene15205 | -1.986804972 | 2.82E-08 | 0.74 | 0.12 | 0.79 | 0.96 | 4.71 | 0.82 | 1.81 | 0.14 | 0.39 | 0.36 | 0.35 | 0.24 |  |
| gene15210 | 1.785700423 | 2.90E-07 | 6.17 | 0.36 | 1.42 | 0.67 | 0.67 | 1.26 | 0.51 | 2.28 | 0.91 | 4.6 | 2.67 | 0.82 | LOC100524949 |
| gene15232 | 1.213284869 | 0.000670551 |  |  |  |  |  |  |  |  |  |  |  |  |  |
| gene15262 | -1.017969603 | 0.001615357 | 1.7 | 2.23 | 1.82 | 2.89 | 4.25 | 5.24 | 5.96 | 0.59 | 0.1 | 0.37 | 0.41 | 0.51 | SLC28A3 |
| gene15275 | -1.167714229 | 0.001483023 | 0.21 | 3.03 | 2.82 | 2.55 | 3.18 | 8.95 | 2.63 | 0 | 0.65 | 0.19 | 0 | 0 | CCL21 |
| gene15304 | -2.031408256 | 2.72E-05 | 1.02 | 0.62 | 1.48 | 2.71 | 5.91 | 3.91 | 1.25 | 4.46 | 1.81 | 6.92 | 1.98 | 1.46 | SPINK4 |
| gene15313 | -1.037657631 | 0.000934835 | 3.85 | 3.25 | 2.26 | 9.03 | 4.05 | 7.59 | 3.33 | 2.46 | 2.72 | 4.16 | 1.48 | 2.91 | DDX58 |
| gene15322 | -1.244985571 | 0.004745633 | 0.25 | 0.19 | 0.36 | 0.46 | 0.84 | 0.6 | 0.33 | 0.41 | 0.51 | 0.94 | 0.33 | 0.33 | LINGO2 |
| gene15424 | 2.919908977 | 1.08E-07 | 5.52 | 2.23 | 1.69 | 0.17 | 1.01 | 0.26 | 5.25 | 3.02 | 4.86 | 2.81 | 5.34 | 1.15 | LOC100519970 |
| gene15427 | -1.551175504 | 0.000618217 | 0.19 | 0.35 | 0.26 | 0.87 | 0.81 | 0.87 | 1.38 | 0.36 | 0.42 | 0.45 | 0.54 | 0.92 | LOC100623946 |
| gene15430 | -1.510770066 | 0.00027807 | 0.1 | 0.16 | 0.35 | 0.14 | 0.72 | 0.91 | 0.47 | 0.54 | 0.39 | 0.46 | 0.28 | 0.1 | ARMC3 |
| gene15448 | -1.614712568 | 0.001069866 | 0.45 | 0.29 | 0.6 | 1.83 | 0.81 | 1.61 | 0.8 | 2.24 | 1.27 | 1.75 | 0.92 | 1.23 | LOC100525122 |
| gene15495 | -1.735727049 | 0.002780863 | 0.09 | 0.04 | 0.33 | 0.24 | 0.96 | 0.04 | 0.16 | 0 | 0.04 | 0 | 0 | 0 | LOC100517730 |
| gene15500 | 1.747498415 | 1.62E-07 | 3.47 | 19.82 | 2.29 | 2.12 | 3.28 | 2.74 | 6.86 | 109.9 | 11.17 | 162.25 | 114.77 | 6.52 | LOC733634 |
| gene15519 | 1.008450452 | 0.001070252 | 36.25 | 58.88 | 49.28 | 20.23 | 28.25 | 27.01 | 36.25 | 33.02 | 44.27 | 43.93 | 50.84 | 60.53 | PFKP |
| gene15530 | -1.269399422 | 0.00496937 | 7.37 | 0.4 | 6.44 | 6.09 | 16.39 | 9.36 | 14.34 | 2.04 | 3.21 | 6.32 | 2.55 | 15.72 | LOC100625534 |
| gene15627 | -1.05437084 | 0.007401926 | 0.72 | 0.67 | 0.55 | 1.02 | 0.61 | 2.83 | 0.58 | 0.71 | 0.73 | 0.61 | 0.48 | 0.32 | GPR12 |
| gene15643 | 2.146263758 | 8.37E-05 | 0.31 | 0.17 | 0.26 | 0.06 | 0.03 | 0.07 | 0.11 | 0.18 | 0.18 | 0.15 | 0.16 | 0.11 | FLT3 |
| gene15655 | 1.299225029 | 0.001687186 | 2 | 0.57 | 0.21 | 0.53 | 0.48 | 0.18 | 0.98 | 0.94 | 0.67 | 0.83 | 0.76 | 0.78 | LOC100738037 |
| gene15663 | -1.256050942 | 0.002707848 | 0.11 | 0.57 | 0.49 | 0.65 | 0.37 | 2.14 | 1.05 | 0.93 | 0.31 | 0.8 | 0.39 | 1.11 | LOC100516873 |
| gene15664 | 1.652320117 | 1.19E-07 | 1412.4 | 407.81 | 817.21 | 305.6 | 385.18 | 209.5 | 141.01 | 295.67 | 378.4 | 1455.4 | 330.77 | 346.32 | HSPH1 |
| gene15665 | 1.61156263 | 2.24E-07 | 561.04 | 164.04 | 306.27 | 115.24 | 153.41 | 94.26 | 53.73 | 119.14 | 205.97 | 627.51 | 178.96 | 183.99 | HSPH1 |
| gene15716 | -1.100224506 | 0.003538473 | 0.33 | 0.38 | 0.85 | 0.75 | 1 | 1.67 | 0.34 | 0.45 | 0.26 | 1.28 | 0.88 | 0.37 | NEK5 |
| gene1574 | 1.020584959 | 0.000938667 | 142.06 | 142.08 | 177.54 | 77.75 | 84.99 | 77.27 | 93.1 | 129.68 | 145.22 | 120.15 | 176.21 | 139.05 | LOC100520822 |
| gene15743 | 1.36179047 | 0.002332015 |  |  |  |  |  |  |  |  |  |  |  |  |  |
| gene15783 | -1.10506491 | 0.000378601 | 11.15 | 20.33 | 12.82 | 48.16 | 16.22 | 37.07 | 20.19 | 32.79 | 33.52 | 26.13 | 34.1 | 36.6 | LOC100625050 |
| gene15797 | 1.540508604 | 0.004490554 |  |  |  |  |  |  |  |  |  |  |  |  |  |
| gene15852 | 1.397598917 | 2.96E-05 |  |  |  |  |  |  |  |  |  |  |  |  |  |
| gene15882 | -1.974477156 | 1.08E-06 | 0.04 | 0.12 | 0.62 | 0.56 | 0.83 | 1.5 | 0.19 | 0.2 | 0.29 | 0.22 | 0.19 | 0.45 | SLITRK6 |
| gene15908 | 1.263879239 | 5.29E-05 | 31.83 | 86.02 | 46.98 | 20.48 | 6.11 | 46.9 | 26.33 | 16.28 | 7.97 | 23.89 | 26.89 | 15.77 | CLDN10 |
| gene15941 | -1.806225213 | 6.33E-05 | 0.08 | 0.09 | 0.05 | 0.33 | 0.21 | 0.32 | 0.07 | 0.04 | 0.02 | 0.02 | 0 | 0.01 | LOC100524825 |
| gene16012 | -1.729180171 | 1.59E-05 | 0.41 | 0.52 | 0.92 | 2.01 | 1.88 | 2.47 | 1.97 | 0.43 | 0.57 | 0 | 0.64 | 0.58 | LOC100523990 |
| gene16066 | 5.428129455 | 9.47E-05 | 0.16 | 0.22 | 0.27 | 0 | 0 | 0 | 0.2 | 0.3 | 0.28 | 0.13 | 0.06 | 0.23 | LOC100738453 |
| gene16142 | -1.166246672 | 0.001336991 | 0.96 | 1.62 | 1.98 | 4.43 | 2.26 | 4.14 | 2.87 | 2.78 | 1.49 | 1.21 | 1.76 | 1.44 | CD300C |
| gene16147 | -1.192143211 | 0.003419526 | 0.26 | 0.19 | 0.43 | 0.36 | 0.24 | 1.42 | 0.35 | 0.32 | 0.11 | 0.26 | 0.15 | 0.4 | DNAI2 |
| gene16158 | 2.015506037 | 0.000490244 | 0.17 | 0.58 | 0.87 | 0.16 | 0.04 | 0.23 | 0.14 | 0.11 | 0.22 | 0.5 | 0.11 | 0.15 | SSTR2 |
| gene16163 | -1.401908995 | 0.000515237 | 0.9 | 0.39 | 0.7 | 1.5 | 2.16 | 1.64 | 2.71 | 1.68 | 0.73 | 1.58 | 0.95 | 0.37 | LOC100625946 |
| gene16173 | -2.179394552 | 0.002077547 | 0.15 | 0 | 0.15 | 0.39 | 0.3 | 0.53 | 0 | 0.08 | 0.26 | 0.12 | 0.29 | 0.21 | LOC100521038 |
| gene16183 | -1.701079523 | 2.84E-05 | 1.14 | 0.89 | 3.18 | 8.74 | 1.44 | 6.03 | 4.78 | 0.66 | 0 | 1.57 | 0.33 | 0.3 | LOC100522637 |
| gene16187 | -4.647471628 | 2.17E-12 | 0 | 0.04 | 0.05 | 0.81 | 0.34 | 2 | 0.03 | 0.15 | 0 | 3.85 | 0.06 | 0.05 | LOC100522100 |
| gene16242 | 2.909614697 | 0.009931868 | 0.19 | 0.13 | 0 | 0 | 0 | 0.04 | 0.07 | 0.1 | 0.05 | 0.15 | 0.05 | 0.09 | CRHR1 |
| gene16299 | -1.013491492 | 0.006294664 | 1.4 | 1.33 | 1.28 | 1.28 | 4.67 | 2.48 | 2.67 | 3.51 | 0.73 | 2.53 | 1.23 | 1.56 | LOC100515853 |
| gene163 | 1.161705197 | 0.000224735 | 2.08 | 7 | 10.85 | 2.98 | 2.85 | 3.38 | 3.2 | 2.32 | 3.02 | 2.01 | 2.03 | 2.46 | GPR126 |
| gene16300 | -2.916483967 | 0.001088723 | 0 | 0.01 | 0.06 | 0.13 | 0.05 | 0.37 | 0.03 | 0.14 | 0.08 | 0 | 0.1 | 0.09 | MPP3 |
| gene16316 | -1.302865167 | 0.001483946 | 1.61 | 1.33 | 1.39 | 2.44 | 6.63 | 1.87 | 1.9 | 1.19 | 0.5 | 3.39 | 1.03 | 2.89 | LOC100624863 |
| gene16430 | -2.041313379 | 0.007905898 | 0 | 0.15 | 0 | 0.35 | 0.28 | 0.35 | 0.34 | 0 | 0.42 | 0 | 0.28 | 0.09 | LOC100737459 |
| gene16504 | 1.106270963 | 0.000330291 | 84.38 | 78.54 | 114.12 | 44.87 | 47.2 | 43.46 | 27.67 | 51.5 | 60.93 | 43.16 | 55.96 | 102.37 | B4GALNT2 |
| gene16507 | 1.858534591 | 7.03E-08 | 5.46 | 6.98 | 6.73 | 1.78 | 2.07 | 1.83 | 0.63 | 5.09 | 4.85 | 5.34 | 6.43 | 9.23 | LOC100621753 |
| gene16531 | 1.035190799 | 0.002560574 | 7.29 | 8.53 | 5.86 | 4.82 | 4.32 | 2.12 | 3.92 | 5.27 | 2.14 | 5.52 | 9.6 | 1.7 | LOC100515686 |
| gene16547 | -5.813620244 | 1.84E-05 | 0 | 0 | 0 | 0 | 0.41 | 0 | 0 | 0 | 0 | 0 | 0 | 0 | LOC100737860 |
| gene16553 | 1.107957461 | 0.000330064 | 18.54 | 26.52 | 21.45 | 12.12 | 8.12 | 12.47 | 12.98 | 15.91 | 18.64 | 29.62 | 18.44 | 24.88 | ABCC3 |
| gene16557 | -1.676450146 | 0.001483053 | 0.03 | 0.1 | 0.06 | 0.21 | 0.32 | 0.15 | 0.19 | 0.05 | 0.18 | 0.07 | 0.02 | 0.02 | LOC100512012 |
| gene16590 | -1.460821205 | 0.004467375 | 0.06 | 0.08 | 0.13 | 0.27 | 0.17 | 0.37 | 0.15 | 0.37 | 0.11 | 0.21 | 0.08 | 0.18 | ANKFN1 |
| gene16598 | -1.071566089 | 0.000722938 | 6.61 | 4.74 | 3.38 | 2.49 | 25.7 | 4.37 | 9.96 | 5.68 | 5.76 | 7.36 | 6.34 | 6.98 | DGKE |
| gene1665 | -1.017123005 | 0.00310429 |  |  |  |  |  |  |  |  |  |  |  |  |  |
| gene16670 | 2.169149883 | 0.001882678 |  |  |  |  |  |  |  |  |  |  |  |  |  |
| gene16692 | 1.010481318 | 0.00131672 | 39.65 | 28.9 | 26 | 16.04 | 16.63 | 17.59 | 16.1 | 23.25 | 26.68 | 39.28 | 45.47 | 40.94 | LOC100623524 |
| gene16701 | -1.828825939 | 0.000371748 | 0.1 | 0.73 | 0.4 | 1.4 | 1.27 | 2.31 | 3.6 | 1.44 | 2.24 | 6.81 | 1.1 | 0.28 | LOC100515857 |
| gene16705 | -2.195081018 | 0.000768113 | 0.25 | 0.16 | 0.23 | 1.18 | 0.97 | 1.16 | 0.88 | 2.3 | 0.15 | 2.34 | 2 | 0.73 | LOC100516396 |
| gene16734 | 1.298464539 | 0.00193675 | 2.61 | 0.9 | 0.5 | 0.43 | 0.75 | 0.63 | 1.72 | 1.14 | 0.93 | 0.86 | 1.34 | 1.75 | CDK5R1 |
| gene16759 | 2.975982325 | 1.76E-16 | 33.68 | 37.83 | 13.04 | 6.56 | 3.97 | 1.16 | 1.13 | 2.6 | 6.16 | 8.05 | 4.26 | 26.28 | LOC100511472 |
| gene16765 | -1.130958735 | 0.0035757 | 1.26 | 0.6 | 0.66 | 2.56 | 1.8 | 1.38 | 0.73 | 2.26 | 1.39 | 1.85 | 1.2 | 2.07 | NLK |
| gene16813 | -1.322898373 | 0.000524637 | 0.93 | 0.79 | 0.83 | 3.61 | 1.06 | 2.16 | 1.11 | 0.83 | 0.73 | 0.68 | 1.21 | 2.45 | LOC100516758 |
| gene16917 | -1.057029684 | 0.000764123 | 6.11 | 7.14 | 3.05 | 12.92 | 13.49 | 9.86 | 13.76 | 6.89 | 2.9 | 4.43 | 6.68 | 3.34 | LOC100523750 |
| gene16958 | -1.118833692 | 0.004097841 | 0.38 | 1.32 | 0.58 | 1.48 | 1.16 | 3.17 | 2.37 | 2.36 | 1.65 | 4.27 | 1.09 | 2.43 | VMO1 |
| gene16961 | 1.028521764 | 0.004744939 | 2.07 | 0.36 | 0.56 | 0.58 | 0.52 | 0.52 | 0.47 | 0.58 | 0.83 | 0.8 | 0.56 | 1.03 | ALOX12 |
| gene16974 | -1.053129128 | 0.004224081 | 1.32 | 1.08 | 0.33 | 2.08 | 1.37 | 2.88 | 0.76 | 4.35 | 2.96 | 3.4 | 3.64 | 1.08 | LOC100739379 |
| gene1707 | -1.37198632 | 0.000941021 | 0.06 | 0.46 | 0.65 | 0.07 | 1.75 | 1.33 | 0.51 | 0.5 | 0 | 0.49 | 0.63 | 0.28 | LOC100515185 |
| gene17126 | 4.6923011 | 0.005613942 |  |  |  |  |  |  |  |  |  |  |  |  |  |
| gene17212 | -1.021389608 | 0.001113303 | 38.58 | 21.11 | 26.08 | 90.76 | 55.71 | 39.36 | 24.69 | 102.53 | 45.49 | 50.24 | 27.83 | 19.65 | LOC100739397 |
| gene17276 | -1.34756944 | 0.000255043 | 0.78 | 0.86 | 0.99 | 1.12 | 2.13 | 3.71 | 1.36 | 1.84 | 0.68 | 0.81 | 0.67 | 1.06 | LOC100622126 |
| gene17285 | -1.720003758 | 0.005603004 | 0.27 | 0.23 | 0.23 | 0.62 | 0.83 | 1.15 | 0.07 | 0.93 | 0 | 1.01 | 0.28 | 0.51 | LOC100739305 |
| gene17344 | 1.430604649 | 7.96E-06 | 26.75 | 12.13 | 11.11 | 8.84 | 4.05 | 7.3 | 3.88 | 19.42 | 7.46 | 14.34 | 9.42 | 10.32 | LOC100620777 |
| gene17417 | -1.09435546 | 0.00331454 | 0.51 | 1.02 | 1.07 | 1.09 | 1.52 | 3.34 | 0.35 | 0.96 | 0.98 | 0.78 | 0.13 | 0.69 | LOC100155669 |
| gene17453 | -1.312252393 | 0.002725852 | 0.03 | 0.22 | 0.28 | 0.43 | 0.44 | 0.48 | 0.2 | 0.15 | 0.08 | 0.47 | 0.08 | 0.18 | CDHR4 |
| gene17455 | -1.301782058 | 0.000129371 | 0.44 | 0.64 | 0.93 | 2.13 | 1.28 | 1.71 | 1.22 | 0.89 | 0.53 | 0.74 | 0.33 | 0.59 | UBA7 |
| gene17476 | -1.029607935 | 0.008083686 | 0.36 | 0.59 | 0.51 | 0.97 | 0.51 | 1.82 | 1.22 | 0.78 | 0.19 | 0.58 | 0.57 | 0.55 | LOC100518334 |
| gene17490 | -2.962865689 | 0.005613942 |  |  |  |  |  |  |  |  |  |  |  |  |  |
| gene17520 | -1.078467394 | 0.009820052 | 0.19 | 0.56 | 0.48 | 0.81 | 0.57 | 1.45 | 0.63 | 0.45 | 1.32 | 0.75 | 0.68 | 0.61 | PHF7 |
| gene17532 | -1.332313292 | 0.002332015 | 0.09 | 0.19 | 0.12 | 0.23 | 0.16 | 0.8 | 0.16 | 0.05 | 0.08 | 0.23 | 0.13 | 0.13 | ITIH1 |
| gene17534 | -2.505014552 | 2.94E-05 | 0.25 | 0.3 | 0.35 | 3.65 | 0.69 | 1.41 | 0.82 | 0.51 | 0 | 3.66 | 0 | 0.7 | LOC100737917 |
| gene17536 | -1.198442411 | 0.002850159 | 0.21 | 0.28 | 0.28 | 0.67 | 0.4 | 0.86 | 0.23 | 0.27 | 0.15 | 1.68 | 0.27 | 0.25 | ITIH4 |
| gene17551 | -1.797635179 | 2.64E-07 | 0.54 | 0.67 | 1.72 | 2.41 | 5.42 | 2.25 | 0.51 | 0.56 | 0.54 | 0.53 | 0.69 | 0.34 | CACNA2D3 |
| gene17573 | 1.398069822 | 8.34E-06 | 25.82 | 36.58 | 35.7 | 12.65 | 14.55 | 11.97 | 8.91 | 17.99 | 18.59 | 24.26 | 23.68 | 30.7 | ABHD6 |
| gene17596 | -1.361689754 | 0.002327812 | 0.67 | 0.88 | 0.57 | 0.83 | 1.4 | 3.77 | 0.87 | 0.89 | 0 | 0.64 | 0.85 | 0.61 | LOC100519514 |
| gene17661 | -1.205031944 | 0.00470442 |  |  |  |  |  |  |  |  |  |  |  |  |  |
| gene17664 | 2.054306797 | 9.84E-10 | 11.28 | 18.7 | 8.86 | 1.36 | 1.58 | 7.4 | 1.49 | 18.36 | 11.07 | 21.41 | 6.81 | 16.68 | LOC100621522 |
| gene17666 | 1.70238764 | 5.73E-08 | 93.65 | 97.12 | 94.73 | 6.11 | 5.94 | 83.1 | 9.51 | 49.59 | 116.44 | 119.96 | 75.03 | 148.34 | OXTR |
| gene17667 | -1.186751346 | 0.001448125 | 1.47 | 1.63 | 3.43 | 6.06 | 4.76 | 4.08 | 5.74 | 11.28 | 3.22 | 3.77 | 1.75 | 2.11 | LOC100623103 |
| gene17752 | -1.475729682 | 0.003513179 | 0 | 0.22 | 0.13 | 0.5 | 0.16 | 0.51 | 0.51 | 0.62 | 0.5 | 0.58 | 0.19 | 0.64 | LOC100622009 |
| gene17791 | -1.020529654 | 0.002413446 | 0.78 | 0.96 | 2.28 | 2.36 | 1.19 | 4.89 | 1.75 | 4.3 | 0.19 | 0.51 | 1.25 | 1.22 | LOC100521929 |
| gene17800 | -1.492294991 | 1.09E-05 | 0.99 | 0.85 | 0.56 | 1 | 2.82 | 3.5 | 0.16 | 0.37 | 0.52 | 7.7 | 0.53 | 0.2 | TF |
| gene17833 | -1.403236811 | 9.49E-06 |  |  |  |  |  |  |  |  |  |  |  |  |  |
| gene17849 | -1.046658072 | 0.000911516 | 10.74 | 18.7 | 36.82 | 40.12 | 34.92 | 67.13 | 23.61 | 39.05 | 32.87 | 39.21 | 50.08 | 35.02 | RBP1 |
| gene17853 | -1.278888348 | 0.003831012 | 0.23 | 0.19 | 0.09 | 0.29 | 0.66 | 0.4 | 0.61 | 0.44 | 0.46 | 0.17 | 0.49 | 0.17 | TRIM42 |
| gene17875 | -1.338512452 | 1.86E-05 | 12.94 | 4.96 | 16.48 | 20.08 | 53.03 | 17.4 | 20.12 | 8.74 | 8.43 | 6.33 | 9.91 | 11.48 | LOC100626514 |
| gene17896 | -1.073659283 | 0.000471852 | 118.41 | 100.53 | 225.94 | 176.28 | 141.84 | 676.56 | 264.51 | 19.78 | 30.07 | 41.27 | 121.55 | 24.19 | CP |
| gene17922 | -1.133726189 | 0.002527684 | 0.17 | 0.16 | 0.14 | 0.12 | 0.77 | 0.16 | 0.31 | 0.28 | 0.12 | 0.49 | 0.08 | 0.4 | IGSF10 |
| gene1794 | 1.580764938 | 7.95E-07 | 7.32 | 10.93 | 6.57 | 3.01 | 2.99 | 2.82 | 3.11 | 5.75 | 8.42 | 10.88 | 6.06 | 8.57 | SUSD1 |
| gene17991 | -1.107824327 | 0.009361298 | 3.49 | 3.82 | 7.53 | 18.6 | 4.73 | 8.45 | 1.09 | 2.91 | 4.53 | 3.74 | 3.11 | 1.21 | BST2 |
| gene18 | 1.714418391 | 9.52E-08 | 8.48 | 10.46 | 8.85 | 3.25 | 1.58 | 4.26 | 2.53 | 2.5 | 4.6 | 7.88 | 7.4 | 4.36 | LOC100512933 |
| gene18004 | 1.504528908 | 1.64E-06 | 26.97 | 39.24 | 38.5 | 11.31 | 11.28 | 16.38 | 5.45 | 4.56 | 8.5 | 10.55 | 15.02 | 13.1 | LOC100154352 |
| gene18007 | 1.118185351 | 0.001491517 | 1.44 | 3.8 | 1.56 | 1.15 | 1.23 | 0.97 | 1.51 | 0.91 | 0.98 | 1.46 | 1.06 | 3.69 | LOC100153543 |
| gene1802 | 2.246354706 | 9.90E-12 | 7.32 | 27.37 | 6.98 | 2.5 | 1.74 | 5.22 | 0.9 | 12.29 | 4.39 | 16.43 | 14.02 | 19.86 | LOC100157182 |
| gene18024 | -1.929341928 | 0.009254525 | 0 | 0.18 | 0.16 | 0.67 | 0.21 | 0.35 | 0.46 | 0.13 | 0 | 0.57 | 0 | 0.09 | CLDN11 |
| gene18073 | 4.623809077 | 2.82E-25 | 5.82 | 4.97 | 5.53 | 0.52 | 0 | 0.21 | 0 | 2.17 | 0.66 | 1.07 | 2.16 | 3.97 | SOX2 |
| gene18083 | 1.073803536 | 0.000524642 | 41.77 | 16.75 | 14.16 | 13.27 | 9.75 | 14.68 | 22.76 | 43.64 | 23.84 | 26.75 | 54.46 | 32.74 | LOC100624866 |
| gene1809 | 1.690814347 | 1.81E-07 | 27.73 | 36.85 | 38 | 6.35 | 13.45 | 13.89 | 8.22 | 8.56 | 19.08 | 21.51 | 16.93 | 19.68 | LOC100622399 |
| gene18105 | -1.210827979 | 0.005572719 | 0.11 | 0.13 | 0.04 | 0.14 | 0.47 | 0.1 | 0.08 | 0.08 | 0 | 0 | 0.05 | 0.12 | VWA5B2 |
| gene1814 | 1.517845695 | 3.10E-05 | 3.65 | 5.78 | 5.9 | 1.69 | 1.93 | 2.02 | 1.95 | 1.19 | 4.04 | 2.14 | 1.74 | 4.28 | CTR1 |
| gene18169 | -1.279954676 | 0.000383824 | 1.12 | 2.83 | 3.19 | 5.17 | 3.04 | 10.22 | 4.69 | 5.7 | 3.5 | 9.76 | 4.67 | 4.47 | LOC100626084 |
| gene18199 | 1.161785578 | 0.0003219 | 6.21 | 3.15 | 3.19 | 1.05 | 3.18 | 1.78 | 2.98 | 1.83 | 2.26 | 2.08 | 3.14 | 4.06 | MFI2 |
| gene18231 | -1.487808029 | 0.001835693 | 0.7 | 0.95 | 0.9 | 3.05 | 2.73 | 1.23 | 3.23 | 1.32 | 0.84 | 0.38 | 0.56 | 0.68 | LOC100738833 |
| gene1824 | -1.208326658 | 0.003630658 | 0.57 | 0.52 | 0.14 | 1.53 | 0.75 | 0.83 | 0.7 | 0.6 | 0.25 | 4.24 | 0.17 | 0.26 | AMBP |
| gene18285 | -1.146171768 | 0.000690295 | 0.86 | 0.7 | 1.65 | 1.61 | 2.31 | 3.45 | 1.81 | 1.9 | 1.3 | 1.84 | 1.24 | 1.75 | LOC100154779 |
| gene18301 | -1.3621983 | 0.000280313 |  |  |  |  |  |  |  |  |  |  |  |  |  |
| gene18302 | -1.548285214 | 0.009580523 |  |  |  |  |  |  |  |  |  |  |  |  |  |
| gene18328 | -1.598353834 | 7.78E-06 | 0.67 | 0.99 | 1.74 | 3.41 | 2.2 | 4.94 | 1.68 | 2.98 | 1.31 | 0.97 | 1.18 | 1.25 | CD200R1 |
| gene18329 | -2.152877497 | 4.65E-05 | 0.28 | 0 | 0.67 | 2.46 | 0.32 | 0.91 | 0.61 | 0.43 | 0.22 | 0.07 | 0.07 | 0.37 | CD200R1L |
| gene18332 | -1.158177565 | 0.000204529 | 4.1 | 4.51 | 7.28 | 13.97 | 7.07 | 16.35 | 6.27 | 5.5 | 7.38 | 5.59 | 4.77 | 8.68 | CCDC80 |
| gene18336 | -1.388862418 | 0.002831539 |  |  |  |  |  |  |  |  |  |  |  |  |  |
| gene1838 | -1.061898475 | 0.000556584 | 12.41 | 54.21 | 92.89 | 130.84 | 111.94 | 98.95 | 112.35 | 23.42 | 67.77 | 36.15 | 36.15 | 64.75 | TNC |
| gene18381 | -1.617511861 | 3.78E-07 | 1.19 | 3.53 | 2.98 | 9 | 6.81 | 8.93 | 3.32 | 1.54 | 1.58 | 3.9 | 1.94 | 4.05 | ABI3BP |
| gene18382 | -1.380000025 | 1.57E-05 | 6.9 | 12.76 | 11.08 | 33.01 | 20.4 | 31.01 | 11.74 | 7.86 | 11.77 | 12.29 | 8.75 | 10.63 | LOC100624376 |
| gene1840 | -1.351636264 | 0.001108318 | 0.58 | 0.6 | 0.65 | 2.15 | 1.8 | 0.78 | 0.86 | 2.62 | 1.25 | 1.38 | 1.48 | 1.28 | LOC100737165 |
| gene1841 | -1.059124212 | 0.000568217 | 5.81 | 27.42 | 39.94 | 57.54 | 52.94 | 46.15 | 56.06 | 6.19 | 46.42 | 15.64 | 19.82 | 34.75 | LOC100737237 |
| gene18468 | 1.24949557 | 0.00836245 | 0.94 | 0.42 | 0.5 | 0.33 | 0.25 | 0.27 | 0.25 | 0.2 | 0.11 | 0.25 | 0.24 | 0.27 | LOC100156650 |
| gene18477 | -1.520423912 | 0.007707738 | 0.46 | 0.62 | 0.3 | 3.46 | 0.78 | 0.16 | 0.09 | 4.29 | 0.8 | 0.37 | 1.97 | 0.33 | LOC100738961 |
| gene18537 | -1.256342245 | 0.002699304 |  |  |  |  |  |  |  |  |  |  |  |  |  |
| gene18538 | 1.268342453 | 4.63E-05 | 11.65 | 24.74 | 20.23 | 3.98 | 6.37 | 14.54 | 5.08 | 5.81 | 10.92 | 11.86 | 12.6 | 25.78 | ADAMTS1 |
| gene18539 | 1.133279029 | 0.000314394 | 7.01 | 23.53 | 11.52 | 4.51 | 3.04 | 12.94 | 2.85 | 5.56 | 6.52 | 8.3 | 9.37 | 15.73 | LOC100738012 |
| gene18540 | -1.109074178 | 0.000339091 | 5.76 | 9.96 | 13.77 | 26.84 | 21.75 | 17.57 | 9.44 | 2.87 | 10.19 | 5.71 | 4.41 | 10.31 | ADAMTS5 |
| gene18557 | -2.048820867 | 1.29E-05 | 0.27 | 0.37 | 1.19 | 0.65 | 3.15 | 2.8 | 0.33 | 0.14 | 0 | 0 | 0.22 | 0 | LOC100624997 |
| gene18598 | 1.010995173 | 0.00105952 | 69.95 | 46.87 | 47.18 | 26.18 | 32.31 | 28.46 | 24.38 | 36.33 | 28.69 | 88.11 | 33.81 | 35.02 | LOC100510984 |
| gene186 | 1.051281014 | 0.001998719 | 4.22 | 3.14 | 5.1 | 2.58 | 1.15 | 2.66 | 0.6 | 5.1 | 1.88 | 4 | 1.92 | 3.58 | LOC100622457 |
| gene18602 | -1.666706064 | 4.24E-05 | 1.84 | 0.95 | 1.42 | 4.54 | 6.91 | 1.95 | 2.98 | 0.81 | 1.53 | 1.74 | 2.18 | 1.62 | LOC100621502 |
| gene18609 | 3.229362709 | 0.003477837 |  |  |  |  |  |  |  |  |  |  |  |  |  |
| gene18613 | -1.029899969 | 0.002539965 | 1.82 | 2.22 | 2.98 | 4.99 | 6.09 | 3.81 | 3.64 | 2.38 | 1.53 | 2.95 | 3.78 | 2.37 | CBR3 |
| gene18648 | -1.626774721 | 2.12E-07 | 15 | 8.41 | 6.17 | 56.05 | 11.81 | 31.59 | 11.77 | 18.95 | 13.86 | 25.9 | 13.78 | 14.81 | MX1 |
| gene18664 | -2.408936963 | 4.03E-10 | 0.77 | 0.07 | 1 | 1.37 | 1.74 | 6.4 | 0.45 | 0 | 0 | 0 | 0.44 | 0.04 | LOC100627826 |
| gene18672 | 1.00161789 | 0.001234773 | 44.49 | 27.42 | 35.54 | 21.48 | 17.84 | 17.99 | 40.85 | 26.07 | 31.28 | 39.07 | 37.2 | 60.51 | LOC100621265 |
| gene1871 | 1.047817652 | 0.000643071 | 493.55 | 571.61 | 402.55 | 219.5 | 273.31 | 260.7 | 242.96 | 302.61 | 553.54 | 498.52 | 535.28 | 782.64 | GGTA1 |
| gene18738 | 1.496970002 | 1.49E-06 | 248.61 | 40.03 | 38.16 | 28.79 | 41.86 | 57.97 | 47.94 | 35.25 | 28.11 | 42.38 | 55.6 | 52.48 | NFIL3 |
| gene18744 | 1.878541881 | 1.37E-06 | 3.38 | 0.38 | 0.36 | 0.49 | 0.43 | 0.29 | 0.22 | 0.47 | 0.43 | 0.12 | 0.27 | 0.21 | LOC100739134 |
| gene18776 | 3.050880988 | 1.13E-09 | 1.68 | 1.52 | 0.72 | 0.17 | 0.08 | 0.31 | 0.46 | 0.57 | 0.88 | 1.17 | 0.57 | 1.79 | EGR3 |
| gene18781 | 1.760041683 | 6.66E-05 | 3.91 | 3.94 | 4.43 | 1.02 | 1.57 | 1.07 | 3.44 | 2.47 | 1.5 | 5.81 | 3.71 | 2.82 | LOC100517193 |
| gene18805 | -1.294280218 | 0.002670728 | 0.04 | 0.19 | 0.3 | 0.61 | 0.33 | 0.43 | 0.79 | 2.44 | 2.01 | 0.24 | 2.2 | 1.88 | LOC100625407 |
| gene18826 | 1.542965653 | 0.005655889 | 0.32 | 0.78 | 0.08 | 0.27 | 0.07 | 0.14 | 1.4 | 0.63 | 0.15 | 0.56 | 0.36 | 0.45 | GULO |
| gene18840 | 1.347993771 | 4.12E-05 | 2.76 | 5.75 | 4.17 | 1.19 | 1.57 | 2.56 | 1 | 2.98 | 2.29 | 3.87 | 2.82 | 3.36 | FZD3 |
| gene18858 | -1.139615833 | 0.001077014 | 0.77 | 1.19 | 0.96 | 2.79 | 1.32 | 2.82 | 1.17 | 1.59 | 2.09 | 1.85 | 1.08 | 0.53 | GATA4 |
| gene18870 | -1.63385607 | 0.000638655 |  |  |  |  |  |  |  |  |  |  |  |  |  |
| gene18897 | -1.979463675 | 2.94E-06 | 1.11 | 0.07 | 0.36 | 0.32 | 5.28 | 0.34 | 1.06 | 0.2 | 0.41 | 0.25 | 0.12 | 1.19 | LOC100627945 |
| gene18919 | 1.977194923 | 0.000869732 | 0.34 | 0.06 | 0.41 | 0.1 | 0.02 | 0.06 | 0.17 | 0.2 | 0.07 | 0.28 | 0.3 | 0.73 | MMP17 |
| gene18976 | 1.006977502 | 0.002373739 | 6.14 | 8.98 | 9.44 | 4.97 | 3.7 | 4.31 | 4.89 | 2.31 | 4.14 | 3.03 | 4.36 | 7.82 | LOC100155773 |
| gene19054 | -1.137867132 | 0.006486446 | 0.53 | 0.3 | 0.37 | 0.84 | 1.22 | 0.69 | 0.88 | 0.35 | 0.34 | 0.64 | 0.53 | 0.55 | RNFT2 |
| gene19063 | 2.004259791 | 6.83E-09 | 8.98 | 3.52 | 6.23 | 1.26 | 2.25 | 1.44 | 1.08 | 1.69 | 1.95 | 2.5 | 2.36 | 1.1 | SDS |
| gene19072 | -1.005621738 | 0.001181118 | 6.82 | 11.93 | 16.63 | 42.91 | 6.64 | 25.06 | 11.11 | 11.38 | 7.91 | 18.31 | 8.71 | 6.96 | OAS2 |
| gene19108 | -1.296450603 | 0.001046647 | 0.85 | 0.87 | 1.24 | 1.46 | 2.43 | 3.69 | 1.29 | 0.76 | 1.84 | 3.11 | 1.23 | 0.97 | LOC100157460 |
| gene19115 | -2.051678007 | 5.64E-06 | 0.18 | 0.33 | 0.1 | 0.13 | 0.33 | 2.72 | 0.23 | 0.45 | 0.32 | 0.41 | 0.17 | 0.51 | OASL |
| gene19136 | 1.500986849 | 0.001930084 | 0.58 | 1.26 | 0.53 | 0.43 | 0.29 | 0.16 | 0.53 | 0.91 | 0.57 | 1.35 | 0.68 | 0.36 | DAO1 |
| gene19147 | -1.073685726 | 0.005547224 | 0.24 | 0.74 | 1.21 | 1.08 | 1.1 | 2.71 | 0.91 | 0.83 | 0.55 | 0.85 | 0.97 | 1.21 | CMKLR1 |
| gene19157 | 1.546346652 | 0.005792782 | 0.12 | 0.14 | 0.23 | 0.03 | 0.04 | 0.12 | 0.34 | 0.27 | 0.06 | 0.14 | 0.2 | 0.05 | SEZ6L |
| gene19191 | 1.683817267 | 2.74E-07 | 5.84 | 10.67 | 23.8 | 4.09 | 3.02 | 6 | 1.75 | 50.52 | 13.96 | 24.48 | 18.07 | 20.79 | LOC100154446 |
| gene19192 | 1.382613114 | 2.08E-05 | 9.21 | 25.7 | 20.03 | 5.31 | 6.88 | 10.03 | 8.03 | 12.46 | 14.35 | 15.44 | 12.99 | 8.07 | LIF |
| gene19200 | 1.176498125 | 0.000640364 | 6.13 | 8.36 | 8.55 | 2.93 | 3.75 | 4.13 | 7.3 | 6.23 | 3.5 | 7.14 | 6.19 | 4.01 | LOC100157687 |
| gene19214 | -1.201514746 | 0.000112486 | 28.95 | 44.3 | 46.17 | 51.5 | 112.5 | 124.27 | 88.64 | 51.65 | 25.65 | 41.37 | 63.62 | 65.56 | SELM |
| gene19251 | 1.328885939 | 6.40E-05 | 7.22 | 4.52 | 3.7 | 2.21 | 2.09 | 2.38 | 1.98 | 4.23 | 1.65 | 7.51 | 3.11 | 2.33 | ADORA2A |
| gene19304 | -1.178878396 | 0.009213575 | 0.03 | 0.36 | 0.3 | 0.26 | 0.53 | 0.85 | 0.05 | 0.44 | 0.33 | 0.46 | 0.31 | 0.4 | TUBA3D |
| gene19324 | -1.414840463 | 4.94E-05 | 1.72 | 2.39 | 2.1 | 2.76 | 7.33 | 7.37 | 3.67 | 1.62 | 1.19 | 1.14 | 0.74 | 2.17 | CLDN5 |
| gene19425 | 1.445861526 | 7.24E-06 | 11.62 | 19.67 | 7.92 | 2.9 | 6.61 | 5.87 | 4.03 | 20 | 15.93 | 39.01 | 16.12 | 21.68 | PHYHIPL |
| gene19440 | -2.019372088 | 0.001864807 | 0.03 | 0.02 | 0.37 | 0.65 | 0.54 | 0.09 | 0.15 | 0.45 | 0.33 | 0.1 | 0.05 | 0.17 | ZNF365 |
| gene19443 | 2.110727561 | 8.91E-07 | 1.58 | 0.79 | 0.75 | 0.31 | 0.28 | 0.19 | 0.93 | 0.48 | 0.98 | 0.51 | 0.68 | 3.32 | EGR2 |
| gene19483 | -1.497684908 | 4.45E-06 | 1.45 | 1.58 | 1.93 | 5.77 | 4.21 | 4.78 | 4.83 | 4.87 | 1.98 | 3.11 | 4.24 | 3.17 | LOC100157199 |
| gene19551 | -1.102077886 | 0.005270178 | 1.17 | 1.38 | 1.34 | 2.3 | 2.85 | 3.79 | 1.47 | 6.2 | 2.56 | 1.07 | 1.61 | 2.07 | LOC100737674 |
| gene19565 | 2.988235009 | 0.009931868 | 0.07 | 0.2 | 0.09 | 0.05 | 0 | 0 | 0.05 | 0.03 | 0 | 0 | 0 | 0 | SFTPD |
| gene19568 | -1.880912256 | 1.30E-06 | 0.48 | 0.57 | 0.34 | 1.19 | 2.58 | 1.6 | 1.13 | 0.68 | 0.56 | 0.57 | 0.65 | 0.65 | MAT1A |
| gene19585 | 2.511014822 | 0.001088723 | 0.08 | 0.17 | 0.19 | 0 | 0.07 | 0 | 0.07 | 0.06 | 0 | 0.07 | 0.16 | 0.06 | LRIT1 |
| gene19602 | 1.457378922 | 0.000116757 | 11.11 | 3.09 | 2.25 | 1.38 | 3.09 | 2.15 | 1.34 | 3.23 | 1.22 | 5.98 | 2.82 | 4.42 | SNCG |
| gene19667 | -2.869218494 | 0.002067442 | 0 | 0.02 | 0 | 0.3 | 0 | 0 | 1.01 | 1.18 | 0.58 | 1.04 | 1.28 | 0.55 |  |
| gene19693 | -2.085746757 | 6.48E-05 | 0.06 | 0.1 | 0.03 | 0.27 | 0.35 | 0.41 | 0.44 | 0.49 | 0.1 | 0.09 | 0.12 | 0.33 | A1CF |
| gene19696 | -1.071437342 | 0.008952958 |  |  |  |  |  |  |  |  |  |  |  |  |  |
| gene19704 | -1.890823536 | 3.76E-08 | 1.77 | 1.29 | 0.13 | 8.05 | 1.09 | 4.07 | 1 | 0.46 | 0.77 | 0.41 | 0.2 | 0.05 | LOC100156277 |
| gene19714 | -1.633646769 | 2.71E-07 | 4.42 | 5.91 | 5.31 | 17.56 | 4.65 | 29.76 | 4.19 | 8.65 | 6.32 | 4.51 | 4.5 | 4.4 | IFIT3 |
| gene19715 | -1.105829578 | 0.000394091 | 9.14 | 8.6 | 7.09 | 24.23 | 7.61 | 25.67 | 4.83 | 18.76 | 10.26 | 15.65 | 7.97 | 12.82 | IFIT1 |
| gene19717 | -1.646514201 | 2.36E-07 | 6.85 | 2.98 | 7.42 | 10.62 | 29.82 | 16.09 | 19.8 | 3 | 5.67 | 3.63 | 3.39 | 4.71 | SLC16A12 |
| gene19752 | 1.646130689 | 2.12E-07 | 20.98 | 43.15 | 21.52 | 8.59 | 11.33 | 8.95 | 14.09 | 25.81 | 21.52 | 38.15 | 23.99 | 26.81 | O3FAR1 |
| gene19765 | -2.524003619 | 0.000327802 |  |  |  |  |  |  |  |  |  |  |  |  |  |
| gene19785 | -1.15285133 | 0.000237991 | 7.56 | 6.77 | 9.21 | 14.82 | 22.46 | 17.77 | 7.82 | 8.88 | 7.8 | 7.9 | 6.61 | 7.2 | BLNK |
| gene19795 | -1.138376354 | 0.009630217 | 0.08 | 0.08 | 0.09 | 0.29 | 0.16 | 0.13 | 0.08 | 0.35 | 0.24 | 0.2 | 0.21 | 0.31 |  |
| gene19810 | 2.473115964 | 1.74E-13 | 13.25 | 17.49 | 21.13 | 3.76 | 2.23 | 3.97 | 1.28 | 14.17 | 4.17 | 34.32 | 13.31 | 27.99 | SFRP5 |
| gene19840 | -1.411941522 | 0.003242503 |  |  |  |  |  |  |  |  |  |  |  |  |  |
| gene19841 | -1.24634347 | 0.000101681 | 7.36 | 3.93 | 5.3 | 15.21 | 7.42 | 19.72 | 22 | 15.12 | 22.91 | 5.72 | 12.86 | 4.85 | KAZALD1 |
| gene19868 | -2.113057282 | 0.009254525 | 0.05 | 0.07 | 0.06 | 0.22 | 0.24 | 0.4 | 0.11 | 0.2 | 0.16 | 0.23 | 0.15 | 0.13 | ELOVL3 |
| gene1989 | -1.330693628 | 0.000228451 |  |  |  |  |  |  |  |  |  |  |  |  |  |
| gene19899 | -1.1394619 | 0.001501589 | 0.32 | 0.28 | 0.52 | 0.39 | 0.46 | 1.76 | 0.73 | 0.71 | 0.6 | 0.48 | 0.77 | 1.05 | WDR96 |
| gene19900 | -1.369403694 | 0.002835258 | 0.22 | 0.38 | 0.2 | 0.28 | 0.59 | 1.49 | 0.72 | 0.62 | 0.86 | 0.6 | 0.64 | 0.48 |  |
| gene19914 | 1.759890069 | 2.32E-08 | 104.54 | 29.52 | 27.5 | 15.64 | 13.99 | 22.89 | 34.5 | 30.5 | 43.23 | 29.49 | 40.82 | 68.51 | DUSP5 |
| gene19917 | 1.237326058 | 0.00013901 | 3.73 | 1.12 | 2.8 | 0.9 | 1.31 | 1.28 | 1.23 | 1.67 | 2.2 | 1.8 | 1.69 | 1.21 | RBM20 |
| gene19927 | 1.170120244 | 0.000801225 | 1.48 | 2.14 | 1.13 | 0.78 | 0.89 | 0.56 | 0.58 | 1.67 | 0.71 | 1.29 | 0.61 | 1.3 | ACSL5 |
| gene1993 | -1.741464291 | 0.007610742 |  |  |  |  |  |  |  |  |  |  |  |  |  |
| gene19952 | -4.246614141 | 3.77E-06 | 0 | 0.02 | 0 | 0.52 | 0 | 0.22 | 0 | 0 | 0 | 0.05 | 0.09 | 0 | PNLIP |
| gene19981 | -1.155207622 | 0.000302956 | 6.47 | 10.24 | 6.55 | 15.12 | 12.84 | 27.53 | 11.96 | 13.24 | 13.22 | 13.22 | 8.66 | 10.98 | LOC100152559 |
| gene19983 | 1.675884954 | 7.78E-08 | 538.1 | 105.51 | 136.5 | 94.07 | 97.55 | 75.98 | 96.64 | 76.76 | 119.82 | 427.7 | 106.7 | 172.83 | BAG3 |
| gene19997 | 1.482227745 | 0.005792782 | 0.38 | 0.62 | 0.57 | 0.23 | 0.27 | 0.06 | 0.22 | 0.08 | 0.14 | 0.25 | 0.24 | 0.39 | LOC100155225 |
| gene20091 | -2.246421124 | 0.000231484 | 0.1 | 0.06 | 0 | 0.21 | 0.15 | 0.63 | 0.1 | 0.08 | 0.04 | 0.99 | 0.04 | 0 | CYP2E1 |
| gene20125 | 1.193646136 | 0.004276423 |  |  |  |  |  |  |  |  |  |  |  |  |  |
| gene20151 | 1.176916417 | 0.001336342 | 2.19 | 1.68 | 3.06 | 0.95 | 1.09 | 1.23 | 1.13 | 2.31 | 0.85 | 2.06 | 1.15 | 1.28 | CXCR4 |
| gene20294 | -2.641121783 | 1.13E-07 | 0.07 | 0.44 | 0 | 0.68 | 0 | 4.23 | 2.12 | 0.08 | 1.27 | 0.46 | 0.28 | 0.41 | LOC100623527 |
| gene20295 | -1.680867611 | 0.001070993 | 0.16 | 0.25 | 0.28 | 0.71 | 0.14 | 1.57 | 1.12 | 0.08 | 0.57 | 0.49 | 0.26 | 0.29 | LOC100736983 |
| gene20312 | -1.122734931 | 0.000263446 | 116.91 | 99.83 | 93.96 | 254.7 | 227.84 | 238.37 | 103.8 | 290.8 | 110.84 | 77.45 | 109.87 | 122.82 | LOC100049687 |
| gene20315 | -1.012310032 | 0.009375769 | 2.21 | 2.26 | 3.25 | 4.61 | 4.79 | 6.84 | 4.29 | 3.58 | 4.36 | 3.6 | 3.23 | 3.91 | LOC100737453 |
| gene20323 | 1.392107752 | 1.55E-05 | 13.94 | 6.32 | 8.58 | 2.81 | 4.14 | 4.92 | 2.23 | 7.97 | 3.34 | 3.23 | 3.9 | 11.09 | LOC100737812 |
| gene20380 | 2.446148956 | 6.58E-14 | 80.43 | 6.49 | 5.41 | 7.29 | 5.63 | 6.07 | 7.56 | 7.85 | 9.1 | 7.02 | 14.27 | 8.66 | LOC100514374 |
| gene20418 | 1.514352706 | 4.83E-06 | 6.52 | 4.35 | 3.66 | 1.21 | 1.28 | 3.09 | 2.45 | 2.36 | 4.89 | 7.2 | 4.98 | 17.31 | NR4A2 |
| gene20458 | -1.365257121 | 2.43E-05 | 1.82 | 1.84 | 1.98 | 4.99 | 7.06 | 3.14 | 4.11 | 4.41 | 2.8 | 3.16 | 2.28 | 2.26 | LOC100736811 |
| gene20465 | -3.542612009 | 2.41E-06 | 0.08 | 0.1 | 0 | 0.6 | 0.78 | 1.83 | 0.62 | 0.44 | 0.52 | 0.14 | 0 | 0 | LOC100513690 |
| gene20470 | -1.006304127 | 0.004636465 | 0.49 | 0.8 | 0.74 | 1.97 | 0.96 | 1.4 | 0.48 | 1.97 | 0.79 | 1.08 | 0.41 | 0.77 | CSRNP3 |
| gene20491 | 2.100192867 | 0.000109086 | 2.47 | 0.22 | 0.07 | 0.06 | 0.12 | 0.52 | 0.46 | 0.43 | 0.27 | 0.59 | 1.46 | 1.33 | LOC100519057 |
| gene20493 | 1.496270644 | 1.25E-05 | 6.52 | 5.67 | 3.85 | 1.4 | 2.12 | 2.66 | 4.27 | 10.18 | 14.77 | 7.27 | 13.65 | 36.63 | LOC100519223 |
| gene20506 | -1.26678188 | 0.000506962 | 2.71 | 0.89 | 0.93 | 1.53 | 0.29 | 10.36 | 2.8 | 4.11 | 3.07 | 3.83 | 4.59 | 4.38 | LOC100155783 |
| gene20547 | -1.406999062 | 0.007372582 | 0.81 | 0.19 | 1.43 | 1.33 | 2.27 | 2.52 | 1.15 | 1.7 | 1.87 | 1.86 | 0.89 | 1.63 | LOC100625611 |
| gene20618 | -1.059586167 | 0.0005998 | 39.66 | 46.68 | 59.09 | 108.36 | 96.97 | 113.92 | 71.5 | 58.54 | 42.26 | 103.65 | 57.21 | 38.63 |  |
| gene20623 | -2.522310627 | 0.009931868 | 0 | 0.02 | 0 | 0 | 0.11 | 0.07 | 0.02 | 0.01 | 0 | 0.04 | 0.02 | 0 | ANKAR |
| gene20638 | -1.337491708 | 0.001021442 | 2.68 | 1.37 | 2.42 | 8.91 | 3.63 | 3.85 | 1.27 | 2.07 | 4.09 | 5.57 | 4.36 | 3.2 | LOC100515418 |
| gene20647 | -2.467015306 | 0.000766471 | 0.06 | 0.11 | 0 | 1.07 | 0.14 | 0.18 | 0.25 | 0.53 | 0 | 0.4 | 0.29 | 0.35 | LOC100515932 |
| gene20684 | 1.233831295 | 0.000294029 | 20.73 | 4.32 | 3.11 | 4.64 | 3.78 | 4.99 | 11.93 | 7.74 | 5.17 | 4.03 | 6.48 | 3.88 | LOC100521691 |
| gene20708 | -1.243289657 | 0.00836245 | 0.12 | 0.25 | 0.44 | 0.39 | 0.45 | 1.13 | 0.77 | 0.63 | 0.37 | 1.05 | 0.52 | 0.39 | LOC100625172 |
| gene20741 | -4.994911671 | 0.001681609 | 0 | 0 | 0 | 0.64 | 1.35 | 0.27 | 0 | 0.87 | 0 | 0 | 0 | 0.45 | LOC100516115 |
| gene20745 | -1.057997569 | 0.001281659 | 2.36 | 3.21 | 2.68 | 4.9 | 8.97 | 4.08 | 3.56 | 2.07 | 1.76 | 3.42 | 2.81 | 0.92 | GPR1 |
| gene20766 | -2.376496677 | 0.004944215 | 0.13 | 0.04 | 0 | 0.35 | 0.15 | 0.65 | 1.61 | 3.8 | 0 | 2 | 0 | 0.74 | LOC100623528 |
| gene20772 | 1.005066406 | 0.001356732 | 2.68 | 3.4 | 3.41 | 1.56 | 2.3 | 1.1 | 2.33 | 2.39 | 3.93 | 4.1 | 4.24 | 4.12 | MAP2 |
| gene20775 | 1.338877758 | 0.004045014 | 1.38 | 1.04 | 1.23 | 0.38 | 0.57 | 0.63 | 0.44 | 0.35 | 0.68 | 0.27 | 0.61 | 0 | LOC100522764 |
| gene20788 | 1.786785159 | 4.03E-06 | 0.24 | 0.92 | 0.15 | 0.26 | 0.08 | 0.08 | 0.87 | 0.01 | 0.35 | 1.79 | 0.39 | 1.62 | CPS1 |
| gene20821 | -1.17870311 | 0.002139996 | 0.15 | 0.53 | 0.41 | 0.95 | 1.49 | 0.19 | 6.02 | 0.23 | 0.35 | 0.57 | 0.35 | 0.24 | RUFY4 |
| gene20844 | -1.635998195 | 0.002893636 | 0.02 | 0.1 | 0.02 | 0.19 | 0.06 | 0.3 | 0.11 | 0.09 | 0.03 | 0.1 | 0.13 | 0.16 | CCDC108 |
| gene20867 | -1.493743889 | 1.67E-06 | 23.15 | 24.97 | 30.24 | 51.41 | 31.56 | 153.02 | 32.09 | 46.77 | 37.96 | 75.26 | 33.96 | 39.48 | LOC100623329 |
| gene20870 | -1.38455419 | 0.002835264 | 0.09 | 0.2 | 0.1 | 0.28 | 0.62 | 0.24 | 0.37 | 0.21 | 0.2 | 0.09 | 0.15 | 0.19 | LOC100737498 |
| gene20887 | 1.616009225 | 0.007857094 | 0.15 | 0.07 | 0.43 | 0.08 | 0.02 | 0.09 | 0.64 | 0.52 | 0.53 | 1.68 | 0.82 | 1.36 | LOC100625740 |
| gene20916 | 4.555316519 | 2.75E-16 | 1.69 | 9.54 | 8.14 | 0.46 | 0 | 0.38 | 0.21 | 1.5 | 0.46 | 1.29 | 3.7 | 1.37 | CCL20 |
| gene20935 | 1.172499223 | 0.003462061 |  |  |  |  |  |  |  |  |  |  |  |  |  |
| gene20980 | 1.484610054 | 0.000510886 | 0.34 | 0.51 | 0.24 | 0.08 | 0.25 | 0.08 | 0.1 | 0.07 | 0.08 | 0.33 | 0.14 | 0.15 | LOC100152603 |
| gene21012 | 1.248881097 | 0.002086393 | 1.78 | 4.13 | 7.7 | 1.58 | 1.04 | 3.59 | 2.08 | 1.77 | 0.38 | 0.51 | 1.91 | 0.3 | MLPH |
| gene21031 | 1.186413476 | 0.006486446 | 0.49 | 0.88 | 0.88 | 0.29 | 0.14 | 0.64 | 0.3 | 0.62 | 0.64 | 0.24 | 0.46 | 0.56 | LOC100512723 |
| gene21033 | 1.918110652 | 9.13E-07 | 0.8 | 0.94 | 0.99 | 0.13 | 0.05 | 0.66 | 0.17 | 0.88 | 1.32 | 1.37 | 1.41 | 1.34 |  |
| gene21089 | 3.60887165 | 1.24E-10 | 0.08 | 0.45 | 7.96 | 0.19 | 0.19 | 0.24 | 9.56 | 15.22 | 0 | 7.87 | 13.33 | 24.55 | LOC100513317 |
| gene2109 | 3.308221197 | 0.001233377 | 0.44 | 1.2 | 1.74 | 0 | 0.25 | 0.05 | 0.52 | 0.23 | 0.84 | 1.42 | 0.49 | 0 | LOC100514333 |
| gene21095 | -1.258211718 | 0.000687142 | 19.45 | 5.65 | 7.8 | 28.91 | 23.95 | 29.52 | 6.51 | 5.49 | 9.88 | 3.31 | 10.18 | 2.89 | OCA2 |
| gene21102 | -1.142469214 | 0.003630658 | 0.12 | 0.7 | 0.2 | 0.18 | 1.93 | 0.48 | 1.03 | 0.54 | 0.05 | 0.64 | 0.38 | 0.22 | LOC100517616 |
| gene21106 | -1.090346097 | 0.001224409 | 0.96 | 1.01 | 0.75 | 1.97 | 0.92 | 3.35 | 1.17 | 0.61 | 0.92 | 1.15 | 1.28 | 0.89 | LOC100736955 |
| gene21109 | 1.354585236 | 6.05E-05 | 0.54 | 0.47 | 0.8 | 0.13 | 0.17 | 0.46 | 0.32 | 0.32 | 0.19 | 0.63 | 0.4 | 0.35 | LOC100623921 |
| gene21206 | -2.242330162 | 2.05E-12 | 98.98 | 269.75 | 248.58 | 387.66 | 604.81 | 2087.4 | 1454.4 | 11.79 | 32.85 | 182.44 | 103.87 | 30.31 | LOC100737864 |
| gene21207 | -2.908419857 | 9.92E-13 | 0.15 | 0.14 | 0.26 | 0.47 | 1.09 | 2.81 | 0.47 | 0.16 | 0.12 | 0.46 | 0.08 | 0.09 | C6 |
| gene21208 | -1.288797364 | 9.57E-05 | 3.29 | 2.8 | 1.92 | 9.94 | 4.28 | 6.79 | 2.25 | 12.41 | 7.74 | 7.65 | 3.32 | 3.02 | PLCXD3 |
| gene21256 | 1.25203593 | 0.000691603 | 3.25 | 3.25 | 6.96 | 1.59 | 1.96 | 2.38 | 0.22 | 2.89 | 2.38 | 2.95 | 2.66 | 2.08 | LOC100739671 |
| gene21276 | -2.218510882 | 4.80E-10 | 0.75 | 0.42 | 0.83 | 1.07 | 2.46 | 6.07 | 2.95 | 0.33 | 0.55 | 0.3 | 0.46 | 0.39 | ANKRD55 |
| gene21284 | 1.171964543 | 0.001145152 | 2.61 | 2.94 | 2.51 | 0.99 | 2.27 | 0.52 | 2.32 | 3.07 | 1.61 | 3.39 | 3.04 | 1.55 | LOC100737888 |
| gene21294 | 1.690648022 | 0.000155192 | 8.73 | 9.4 | 5.79 | 3.02 | 1.78 | 3.35 | 4.23 | 4.13 | 4.81 | 9.58 | 10.31 | 11.91 | LOC100523871 |
| gene21313 | -1.203555827 | 0.002096901 | 7.04 | 5.94 | 3.63 | 25.87 | 8.38 | 6.12 | 13.85 | 1.88 | 21.18 | 4.99 | 0.31 | 5.72 | LOC100739127 |
| gene21385 | 1.045471752 | 0.000812818 | 35.71 | 40.29 | 37.15 | 19.69 | 18.03 | 20.51 | 27.31 | 19.63 | 30.47 | 29.4 | 32.8 | 30.14 | LOC100523218 |
| gene21388 | 1.060438159 | 0.001357443 | 3.39 | 18.25 | 9.42 | 6.55 | 3.77 | 5.52 | 4.87 | 10.07 | 4.14 | 6.58 | 5.93 | 6.34 | LOC100525013 |
| gene21399 | 1.758137261 | 8.09E-05 | 2.09 | 0.33 | 0.58 | 0.23 | 0.22 | 0.55 | 1.68 | 0.35 | 0.17 | 0.35 | 0.21 | 0.24 | FOXI1 |
| gene21401 | -1.23564379 | 0.008270607 | 0.41 | 0.56 | 0.15 | 0.93 | 1.67 | 0.42 | 0.73 | 1.76 | 0.51 | 0.76 | 0.64 | 0.42 | LOC100739454 |
| gene21433 | -1.24183557 | 9.72E-05 | 8.49 | 6.73 | 5.15 | 15.36 | 18.09 | 18.14 | 4.45 | 2.4 | 3.42 | 5 | 3.31 | 4.94 | C1QTNF2 |
| gene21459 | -1.862045444 | 8.70E-05 | 0.07 | 0.27 | 0.4 | 1.22 | 0.16 | 1.31 | 0.53 | 1.02 | 0.53 | 0.6 | 1.11 | 0.27 | LOC100523827 |
| gene21480 | 1.685929835 | 0.007855034 | 0.26 | 0.67 | 0.35 | 0.15 | 0.2 | 0.12 | 1 | 0.09 | 0 | 0.14 | 0.14 | 0.25 |  |
| gene21482 | 4.850059767 | 0.0030599 | 0 | 0.15 | 0.24 | 0 | 0 | 0 | 0.05 | 0 | 0 | 0 | 0 | 0 | NMUR2 |
| gene21494 | -1.282463273 | 3.88E-05 | 15.37 | 11.72 | 21.1 | 27.51 | 53.81 | 41.58 | 40.83 | 13.1 | 15.73 | 15.78 | 15.08 | 17.19 | SLC36A2 |
| gene21501 | -1.029550526 | 0.000817788 | 138.85 | 80.14 | 97.61 | 246 | 275.94 | 166.59 | 248.45 | 184.78 | 171.26 | 264.49 | 181.68 | 180.2 | GPX3 |
| gene21529 | 1.213227833 | 0.000401939 | 2.16 | 2.69 | 0.25 | 0.36 | 1.43 | 0.56 | 1.98 | 0.52 | 0.33 | 0.99 | 0.39 | 0.13 |  |
| gene21553 | 3.101570936 | 0.00586914 | 0 | 0.11 | 0.14 | 0 | 0.02 | 0 | 0.07 | 0 | 0.18 | 0.03 | 0.17 | 0.15 | LOC100622160 |
| gene21556 | -1.13645633 | 0.001161692 | 15.22 | 9.09 | 12.39 | 33.24 | 15.24 | 37.63 | 15.24 | 11.66 | 11.04 | 12.2 | 13.72 | 10.3 | LOC100523337 |
| gene21588 | -1.49090912 | 0.002311575 | 0.46 | 0.16 | 0.2 | 0.63 | 1.05 | 0.82 | 1.04 | 1.51 | 0.85 | 2.24 | 0.23 | 0.43 | LOC100516651 |
| gene216 | 2.828423423 | 1.11E-06 | 0.26 | 1.38 | 0.54 | 0 | 0.04 | 0.32 | 0.15 | 0.55 | 0.07 | 1.24 | 0.82 | 0.59 | LOC100157645 |
| gene21640 | 1.379286294 | 1.11E-05 | 26.21 | 49.03 | 34.82 | 16.52 | 12.81 | 15.35 | 11.6 | 27.41 | 30.17 | 33.61 | 23.91 | 33.44 | LOC100511905 |
| gene21643 | -1.737527993 | 6.14E-06 | 0.12 | 0.11 | 0.16 | 0.67 | 0.21 | 0.48 | 0.32 | 0.79 | 0.45 | 0.35 | 0.68 | 0.37 |  |
| gene21644 | -1.621138038 | 0.007589559 |  |  |  |  |  |  |  |  |  |  |  |  |  |
| gene21647 | 1.121675253 | 0.000265689 | 477.59 | 541.67 | 531.62 | 183.77 | 316.7 | 250.48 | 231.44 | 262.86 | 366.07 | 358.2 | 355.84 | 756.97 | PRNP |
| gene21657 | -1.370108944 | 0.000191683 | 1.13 | 1.28 | 1.56 | 4.23 | 3.19 | 3.21 | 2.12 | 3.26 | 2.56 | 3.46 | 2 | 0.8 | LOC100739819 |
| gene21694 | -1.323094116 | 0.000259184 | 0.81 | 0.35 | 0.54 | 0.78 | 2.55 | 1.11 | 0.52 | 0.08 | 0.32 | 0.42 | 0.12 | 0.29 | SEL1L2 |
| gene21697 | -2.610827106 | 3.51E-14 | 3.26 | 1.63 | 3.78 | 19.09 | 26.03 | 8.61 | 23.65 | 5.65 | 2.39 | 3.71 | 4.66 | 4.82 | LOC100738215 |
| gene21731 | 2.202092769 | 0.000963035 | 0.09 | 0.92 | 0.08 | 0.07 | 0.07 | 0.18 | 0.05 | 0.03 | 0.11 | 0.3 | 0 | 0.22 | LOC100511966 |
| gene21756 | -3.126009385 | 0.0030599 | 0 | 0.05 | 0 | 0.34 | 0.4 | 0.37 | 0.16 | 0.62 | 0 | 0 | 0 | 0 | CST7 |
| gene21769 | 1.147132524 | 0.000211387 | 75.26 | 36.67 | 44.59 | 15.2 | 12.55 | 49.29 | 21.84 | 56.4 | 43.03 | 24.25 | 91.67 | 80.51 | SMOX |
| gene21781 | -1.419094837 | 0.000305861 | 0.04 | 0.14 | 0.18 | 0.34 | 0.25 | 0.44 | 0.19 | 0.21 | 0.13 | 0.29 | 0.29 | 0.19 | SIGLEC-1 |
| gene21812 | 2.27635094 | 4.37E-10 | 3.19 | 0.62 | 4.32 | 1.01 | 0.55 | 0.15 | 0.71 | 1.56 | 0.04 | 1.23 | 0.79 | 0.25 | LOC100523828 |
| gene21813 | 2.17642225 | 3.93E-10 | 7.11 | 2.84 | 18.73 | 3.73 | 1.7 | 1.03 | 3.05 | 4.13 | 1.05 | 5.41 | 6.9 | 2.43 | LOC100739609 |
| gene2182 | -1.159399657 | 0.003462061 | 0.25 | 0.23 | 0.22 | 0.39 | 0.54 | 0.69 | 0.94 | 0.33 | 0.1 | 0.09 | 0.11 | 0.15 | SARDH |
| gene21854 | 1.986106738 | 1.10E-09 | 51.54 | 118.88 | 51.95 | 6.75 | 3.98 | 50.55 | 0.53 | 19.81 | 5.08 | 13.22 | 22.83 | 13.04 | LOC100521049 |
| gene21859 | 3.522081169 | 4.34E-25 | 143.06 | 426.65 | 136.55 | 40.79 | 4.85 | 19.83 | 6.47 | 145.6 | 105.48 | 234.58 | 185.58 | 285.06 | LOC100738325 |
| gene2189 | -1.299513688 | 0.002465929 | 0.16 | 0.34 | 1.45 | 1.7 | 1.22 | 1.57 | 0.25 | 0.16 | 0.27 | 0.7 | 0 | 0.39 | FCN2 |
| gene21931 | 1.317898259 | 7.83E-05 | 10.13 | 8.78 | 9.2 | 1.96 | 4.86 | 5.19 | 4.75 | 5.25 | 5.45 | 7.87 | 7.36 | 10.86 | LOC100152247 |
| gene2196 | -1.946282733 | 5.28E-05 | 0.42 | 0.2 | 0 | 0.38 | 0.98 | 1.51 | 0.33 | 0.13 | 0.09 | 0.15 | 0 | 0.06 | LOC100519464 |
| gene2197 | -1.516046121 | 1.03E-06 | 521.21 | 729.17 | 868.8 | 997.46 | 1328.7 | 4101.8 | 1860.2 | 76.82 | 82.44 | 71.05 | 504.43 | 59.48 | UCAL-P19 |
| gene21973 | 2.735470139 | 0.001218083 | 0.82 | 0.11 | 0.16 | 0.14 | 0 | 0 | 0.85 | 0.06 | 0.2 | 0 | 0.09 | 0 | C20orf118 |
| gene22024 | -1.268966343 | 0.000151162 | 0.56 | 0.96 | 6.54 | 12.88 | 2.24 | 4.05 | 3.43 | 3.75 | 1.74 | 4.06 | 2.28 | 1.43 | LOC100157757 |
| gene22034 | 4.174339946 | 2.08E-05 | 0.16 | 0.37 | 0.18 | 0 | 0 | 0.04 | 0.37 | 0.08 | 0.09 | 0.39 | 0.44 | 0.11 | HNF4A |
| gene22053 | -1.865765368 | 1.93E-07 | 0.21 | 0.79 | 0.12 | 1.87 | 1.22 | 1.62 | 8.36 | 0.16 | 0.15 | 0.49 | 0.53 | 0.59 | MATN4 |
| gene22066 | -1.489457217 | 0.003368493 | 0.69 | 0.15 | 1.41 | 2.61 | 1.79 | 1.16 | 4.92 | 5.16 | 0.55 | 0.51 | 3.75 | 1.53 | LOC100738160 |
| gene22078 | -1.377547605 | 1.38E-05 | 15.65 | 16.08 | 13.32 | 59.81 | 31.68 | 33.06 | 22.26 | 24.47 | 27.83 | 32.37 | 19.55 | 19.2 | TNNC2 |
| gene2208 | 1.387767875 | 0.002815316 | 0.55 | 0.15 | 0.27 | 0.11 | 0.14 | 0.13 | 0.08 | 0.08 | 0.08 | 0.32 | 0.26 | 0.54 | LOC100154175 |
| gene22136 | 1.146287262 | 0.000201689 | 143.1 | 117.73 | 92.11 | 53.28 | 54.61 | 62.81 | 67.21 | 71.45 | 92.03 | 103.36 | 120.54 | 150.08 | PTPN1 |
| gene22158 | 2.50872337 | 2.99E-07 | 6.38 | 3.58 | 1.21 | 1.07 | 0.75 | 0.19 | 2.39 | 0.96 | 3.19 | 3.6 | 2.47 | 2.24 | LOC100514080 |
| gene22159 | 1.295251721 | 5.23E-05 | 8.02 | 15.22 | 4.11 | 4.77 | 3.09 | 4.07 | 2.68 | 5.67 | 2.79 | 5.96 | 5.42 | 4 | CYP24A1 |
| gene22181 | 3.040354879 | 1.25E-19 | 25.53 | 38.21 | 0.86 | 1.02 | 5.71 | 1.64 | 2.28 | 1.87 | 13.84 | 31.03 | 20.02 | 35.88 | PCK1 |
| gene22182 | -1.299549625 | 0.000208966 | 0.66 | 1.26 | 0.65 | 2.71 | 1.85 | 2.29 | 1.13 | 0.83 | 1.03 | 1.31 | 0.93 | 0.78 | ZBP1 |
| gene22210 | -2.199867784 | 1.50E-07 | 0.66 | 0.34 | 4.12 | 14.58 | 4.72 | 1.92 | 7.22 | 2.19 | 3.1 | 0.72 | 1.22 | 15.42 | LOC100626187 |
| gene22234 | -8.003923749 | 2.62E-17 | 0 | 0 | 0 | 0 | 1.84 | 0.95 | 1.97 | 0.93 | 2.42 | 3.25 | 1.12 | 3.23 | LOC100512191 |
| gene22273 | -1.716005244 | 0.001622192 | 0.22 | 0.2 | 0.37 | 0.83 | 0.57 | 1.43 | 0.44 | 0.06 | 0.48 | 0 | 0 | 0.08 | LOC100624234 |
| gene22274 | -1.202820083 | 0.001486344 | 0.57 | 0.75 | 0.94 | 1.52 | 1.28 | 2.6 | 0.93 | 0.6 | 0.5 | 0.58 | 0.23 | 0.31 | LOC100739295 |
| gene22319 | 1.164175221 | 0.000297495 | 3.14 | 5.1 | 5.81 | 3.37 | 1.51 | 1.72 | 2.28 | 3.51 | 2.38 | 4.38 | 3.27 | 5.68 | KEL |
| gene22323 | -1.810636459 | 0.002031086 | 0.13 | 0.11 | 0.35 | 0.57 | 0.61 | 0.9 | 0.5 | 0.14 | 0 | 0.07 | 0.07 | 0.57 | LOC100622463 |
| gene22377 | 1.357450323 | 0.000465793 | 8.7 | 1.54 | 2.58 | 2.68 | 1.68 | 0.84 | 6.66 | 0.81 | 0.34 | 2.04 | 1.84 | 1.81 | LOC100517620 |
| gene22388 | -1.452534356 | 2.97E-06 | 68.34 | 85.15 | 143.97 | 340.72 | 192.56 | 321.07 | 173.72 | 80.11 | 57.41 | 198.89 | 87.88 | 128.42 | PTF-BETA |
| gene22390 | 3.290725252 | 5.03E-05 | 0.63 | 1.9 | 0.5 | 0.02 | 0.29 | 0 | 0.1 | 0.79 | 0.88 | 0.41 | 0.8 | 0 | LOC100621475 |
| gene22392 | -1.386685434 | 4.78E-05 | 1.57 | 3.73 | 4.45 | 8.58 | 5.75 | 12.56 | 4.88 | 3.72 | 7.4 | 8.25 | 3.82 | 5.53 | LOC100737678 |
| gene22404 | -1.012925199 | 0.006518351 | 0.42 | 0.57 | 0.68 | 0.79 | 0.68 | 2.17 | 0.72 | 1.18 | 0.58 | 0.96 | 0.52 | 0.94 | LOC100525318 |
| gene22487 | -1.189541877 | 0.001871417 | 0.33 | 0.46 | 0.3 | 0.77 | 0.62 | 1.39 | 0.91 | 1.44 | 0.32 | 1.27 | 0.68 | 0.62 | GPR37 |
| gene22513 | -1.236097168 | 0.00836245 | 0.26 | 0.32 | 0.57 | 0.3 | 0.3 | 2.3 | 2 | 0.83 | 2.48 | 0.8 | 0.47 | 0.62 | LOC100512382 |
| gene22514 | -2.692519602 | 0.001218083 | 0 | 0.06 | 0.05 | 0.2 | 0.08 | 0.53 | 1.13 | 0.3 | 1.23 | 0.58 | 0.05 | 0.33 | KCND2 |
| gene22518 | 1.253324653 | 5.25E-05 | 10.04 | 18.6 | 18.3 | 5 | 7.84 | 7.75 | 5.4 | 13.67 | 7.8 | 15.25 | 10.75 | 17.28 | LOC100517505 |
| gene22519 | 2.662002021 | 2.32E-16 | 23.39 | 36.02 | 24.2 | 5.34 | 2.01 | 6.78 | 0.86 | 22.68 | 12.91 | 23.65 | 15.84 | 22.7 | CFTR |
| gene22614 | 1.079578617 | 0.00093178 | 10.62 | 18.94 | 25.44 | 10.27 | 9.43 | 7.38 | 11.12 | 9.35 | 9.64 | 17.11 | 8.54 | 6.03 | LOC100513831 |
| gene22651 | -1.162794967 | 0.002309826 | 0.58 | 0.82 | 2.02 | 2.21 | 2.65 | 2.76 | 2.49 | 1.61 | 1.49 | 1.46 | 1.63 | 1.54 | LOC100622673 |
| gene22668 | -1.545917111 | 0.001534653 | 0.27 | 0.21 | 0.36 | 1.55 | 0.49 | 0.56 | 0.74 | 1.78 | 1.02 | 1.45 | 1.65 | 0.67 | LOC100525319 |
| gene22671 | 1.574402521 | 7.15E-07 | 63.94 | 8.5 | 18.75 | 12.92 | 7.02 | 13.96 | 13.86 | 80.73 | 25.96 | 8.39 | 107.14 | 38.96 | IGFBP1 |
| gene22673 | 2.750150674 | 1.48E-12 | 3.15 | 0.23 | 0.22 | 0.1 | 0.18 | 0.36 | 0.31 | 0.66 | 0.61 | 0.96 | 0.58 | 0.05 | LOC100624934 |
| gene22737 | -1.051365669 | 0.001329389 | 2.43 | 1.51 | 2.12 | 4.39 | 6.25 | 2.5 | 5.19 | 2.07 | 2.5 | 2.6 | 1.75 | 1.62 | STS |
| gene22793 | 1.033666302 | 0.000767602 | 145.73 | 327.68 | 69.48 | 101.49 | 107.32 | 71.82 | 72.89 | 135.06 | 152.45 | 345.52 | 101.01 | 109.24 | LOC100520595 |
| gene22805 | -1.3077844 | 0.000182772 | 4.79 | 2.96 | 4.66 | 7.62 | 17.51 | 6.34 | 7.08 | 6.2 | 6.12 | 7.49 | 4.9 | 4.44 | LOC100738313 |
| gene22810 | -1.49434395 | 2.53E-06 | 5.1 | 2.88 | 4.82 | 9.71 | 16.09 | 12.25 | 11.45 | 6.8 | 4.97 | 4.23 | 4.12 | 6.67 | PPEF1 |
| gene22818 | -1.258640255 | 6.56E-05 | 6.86 | 2.08 | 4.44 | 17.23 | 3.04 | 14.38 | 7.43 | 14.76 | 6.93 | 3.67 | 15.46 | 5.92 | MAP7D2 |
| gene22884 | -1.093304189 | 0.003180982 | 1.39 | 1.35 | 1.17 | 4.36 | 1.5 | 3.19 | 1.04 | 3.24 | 3.33 | 1.57 | 1.95 | 0.81 | LOC100738503 |
| gene22897 | 4.006559092 | 1.42E-05 |  |  |  |  |  |  |  |  |  |  |  |  |  |
| gene22946 | -1.167006189 | 0.009820052 | 0.56 | 0.16 | 0.24 | 1.5 | 0.27 | 0.45 | 0.27 | 1.71 | 0.32 | 0.21 | 1.68 | 0.37 | NYX |
| gene22998 | -1.934127178 | 5.90E-07 | 0.45 | 0.16 | 0.32 | 0.74 | 1.77 | 1.06 | 0.45 | 0.24 | 0.42 | 0.31 | 0.13 | 0.25 |  |
| gene23008 | -1.640465007 | 0.001255674 | 0.18 | 0.2 | 0.04 | 0.61 | 0.6 | 0.31 | 0.27 | 0.16 | 0.11 | 0.74 | 0.48 | 0.09 | GATA1 |
| gene23070 | 1.258886315 | 0.000304016 | 4.79 | 7.76 | 4.72 | 2.12 | 2.86 | 2.77 | 3.81 | 2.54 | 3.95 | 4.77 | 4.71 | 5.25 | LOC100521307 |
| gene23080 | 1.264946554 | 6.74E-05 | 16.44 | 2.72 | 7.05 | 4.1 | 3.59 | 4.19 | 1.8 | 2.38 | 3.23 | 8.67 | 2.31 | 12.54 | LOC100518817 |
| gene23093 | -1.098583251 | 0.000545451 | 33.65 | 34.88 | 40.93 | 74.64 | 104.76 | 65.87 | 88.15 | 63.04 | 35.64 | 37.75 | 30.74 | 33.88 | LOC100152387 |
| gene23112 | -1.319863404 | 0.000824407 | 0.07 | 0.23 | 0.1 | 0.33 | 0.34 | 0.48 | 0.11 | 0.45 | 0.77 | 0.23 | 0.19 | 0.16 | LOC100512938 |
| gene23156 | 1.279480557 | 3.94E-05 | 52.98 | 70.68 | 63.04 | 29.94 | 14.74 | 37.28 | 5.84 | 20.21 | 12.47 | 23.23 | 37.2 | 36.7 | LOC100519581 |
| gene23290 | 1.119318816 | 0.008376346 | 0.23 | 0.88 | 0.47 | 0.26 | 0.2 | 0.35 | 0.4 | 0.61 | 0.73 | 1 | 0.69 | 0.48 | PCDH19 |
| gene23323 | -1.574628359 | 0.002192047 | 1.52 | 1.05 | 1.16 | 5.63 | 2.76 | 2.83 | 2.45 | 10.53 | 6.48 | 1.46 | 6.53 | 3.43 | LOC100522115 |
| gene23346 | -3.632690467 | 0.000292606 | 0 | 0.08 | 0 | 0.59 | 1.15 | 0.18 | 0.2 | 0.66 | 0.44 | 0.2 | 0 | 0 | LOC100524125 |
| gene23351 | -1.534267639 | 0.009580523 | 0.63 | 0.12 | 0.08 | 0.52 | 1.01 | 1.09 | 0.3 | 0.72 | 0.22 | 0.53 | 1.79 | 0.54 | LOC100524295 |
| gene23430 | -1.117612999 | 0.004912703 | 0.23 | 0.3 | 0.47 | 0.68 | 0.65 | 0.96 | 1.13 | 0.89 | 0.61 | 0.69 | 0.72 | 1.01 | LRCH2 |
| gene23463 | 1.615412683 | 2.62E-07 | 21.79 | 33.46 | 24.09 | 9.38 | 8.3 | 9.74 | 7.4 | 11.9 | 22.33 | 20.07 | 20.2 | 36.52 | LONRF3 |
| gene23516 | 1.013672049 | 0.002267579 | 2.31 | 1.55 | 4.85 | 2.6 | 1.26 | 0.54 | 0.46 | 5.07 | 1.19 | 2.3 | 1.02 | 1.18 | FRMD7 |
| gene23521 | 1.500146488 | 0.001638873 | 0.76 | 1.5 | 0.28 | 0.21 | 0.37 | 0.47 | 0.43 | 0.26 | 0.19 | 0.76 | 0.45 | 0.51 | LOC100153213 |
| gene23525 | -1.061513538 | 0.000614143 | 11.55 | 11.94 | 19.28 | 29.08 | 26.25 | 38.61 | 13.36 | 20.65 | 11.6 | 18.85 | 13.49 | 12.29 | GPC3 |
| gene23526 | 2.046188282 | 4.82E-08 | 3.08 | 5.67 | 3.64 | 0.78 | 0.8 | 1.77 | 0.95 | 2.53 | 2.54 | 2.4 | 1.81 | 4.34 | LOC100516533 |
| gene23578 | 3.439802802 | 4.33E-17 | 2.25 | 2.32 | 0.1 | 0.07 | 0.27 | 0.13 | 2.98 | 1.14 | 0.53 | 1.33 | 0.7 | 0.46 | LOC100525800 |
| gene23616 | -1.161836626 | 0.000193938 | 11.1 | 8.75 | 11.62 | 4.99 | 59.11 | 8.73 | 31.45 | 3.29 | 5.57 | 8.92 | 6.85 | 9.18 | GABRE |
| gene23627 | 1.037439159 | 0.004126629 | 0.8 | 6.4 | 1.06 | 1.53 | 2.14 | 0.67 | 2.27 | 0.49 | 3.33 | 0.54 | 0.93 | 1.57 | LOC100737297 |
| gene23632 | -1.04843276 | 0.004692734 | 0.33 | 0.72 | 1.57 | 1.29 | 1.67 | 2.57 | 0.93 | 1.75 | 0.58 | 1.47 | 0.98 | 1.21 | LOC100623680 |
| gene23719 | 1.159703473 | 0.00022878 | 2.71 | 3.27 | 2.62 | 0.92 | 1.34 | 1.84 | 2.87 | 2.25 | 1.14 | 2.28 | 1.65 | 1.34 | LOC100625207 |
| gene23723 | 1.28433307 | 3.39E-05 | 53.86 | 55.69 | 46.12 | 14.7 | 27.45 | 25.67 | 57.54 | 39 | 21.68 | 46.05 | 14.95 | 21.89 | LOC100624590 |
| gene23733 | 1.054633378 | 0.002778907 |  |  |  |  |  |  |  |  |  |  |  |  |  |
| gene23747 | 1.195808672 | 0.000583862 | 1.7 | 9.35 | 4.2 | 1.57 | 3.33 | 2.2 | 2.33 | 6.06 | 8.24 | 4.75 | 9.47 | 3.93 | SLC7A11 |
| gene23748 | 1.418903764 | 4.69E-06 | 882.06 | 825.64 | 933.99 | 383.69 | 383.06 | 276.92 | 590.66 | 895.37 | 787.52 | 991.75 | 1130.9 | 1256.9 | LOC100628052 |
| gene23763 | 1.312706168 | 0.001168302 | 1.23 | 1.03 | 1.21 | 0.44 | 0.43 | 0.66 | 1.39 | 0.36 | 0.65 | 1.98 | 1.46 | 0.62 | IRK1 |
| gene23766 | 4.770916763 | 0.0030599 | 0.3 | 0.07 | 0.06 | 0 | 0 | 0 | 0 | 0.02 | 0 | 0 | 0.06 | 0.06 | LOC100156191 |
| gene23786 | 3.214065812 | 0.003477837 | 0.17 | 0.13 | 0.03 | 0 | 0.03 | 0 | 0.06 | 0.15 | 0 | 0.04 | 0.23 | 0.07 | NETO2 |
| gene238 | 1.811557878 | 6.30E-05 | 1.81 | 0.43 | 1.09 | 0.32 | 0.36 | 0.28 | 0.28 | 0.45 | 2.63 | 1.95 | 0.31 | 0.9 | ARG1 |
| gene23814 | -4.684136047 | 0.005613942 | 0 | 0 | 0 | 0.45 | 0 | 0.08 | 0.04 | 0 | 0 | 0 | 0.09 | 0 | LOC100626968 |
| gene239 | -1.361172321 | 0.000463817 | 3.49 | 1.1 | 3.52 | 4.1 | 11.21 | 6.07 | 5.56 | 4.03 | 3.37 | 4.89 | 2.47 | 3.27 | LOC100523651 |
| gene24019 | -1.026176933 | 0.000859185 | 41.4 | 45.35 | 31.69 | 69.48 | 90.44 | 96.76 | 74.2 | 27.97 | 39.54 | 29.55 | 30.07 | 46.88 | LOC100624383 |
| gene24032 | -1.569394632 | 0.000303702 | 0.43 | 1.83 | 1.52 | 2.69 | 3.25 | 6.61 | 2.3 | 2.25 | 1.5 | 0.93 | 1.56 | 1.84 | LOC780407 |
| gene24037 | 1.70878505 | 0.000523441 | 1.01 | 0.27 | 1.11 | 0.38 | 0.08 | 0.26 | 0.26 | 0.3 | 0.25 | 0.12 | 0.4 | 0.05 | SCL10A2 |
| gene24077 | 3.173990035 | 0.000123264 | 0.99 | 1.47 | 1.13 | 0 | 0.33 | 0 | 2.16 | 0.44 | 0.25 | 3.23 | 0 | 0 | LOC100622016 |
| gene24124 | -1.480234745 | 0.005840954 | 0.07 | 0.15 | 0.06 | 0.52 | 0.08 | 0.33 | 0.36 | 0 | 0.31 | 0.07 | 0.17 | 0.43 | LOC100626686 |
| gene24129 | 2.245846616 | 0.00392776 |  |  |  |  |  |  |  |  |  |  |  |  |  |
| gene24133 | -1.418711034 | 0.000167218 | 0.68 | 0.91 | 1.44 | 1.8 | 0.99 | 5.67 | 0.98 | 2.22 | 1.5 | 2.06 | 1.78 | 2.13 | LOC100153525 |
| gene2414 | -1.243441334 | 0.00012504 | 3.24 | 3.01 | 3.81 | 7.43 | 9.72 | 7.91 | 6.99 | 3.33 | 3.24 | 3.57 | 3.25 | 3.68 | EFCAB4A |
| gene24140 | 2.248112747 | 3.46E-09 | 1.44 | 2.29 | 1.65 | 0.53 | 0.15 | 0.56 | 0.24 | 0.16 | 0.34 | 1.15 | 0.92 | 1.18 | UNC5CL |
| gene24157 | -1.434079755 | 3.78E-05 | 0.92 | 1.22 | 2.07 | 5.11 | 5.03 | 1.4 | 0.57 | 1.39 | 0.62 | 0.2 | 0.48 | 0.6 | LOC100621966 |
| gene24271 | 4.600108863 | 0.005613942 | 0.17 | 0.1 | 0.05 | 0 | 0 | 0 | 0.03 | 0.04 | 0 | 0.17 | 0 | 0.05 | LOC100625382 |
| gene24280 | 1.374109268 | 0.005574518 |  |  |  |  |  |  |  |  |  |  |  |  |  |
| gene24299 | -2.08286404 | 6.83E-06 | 0.14 | 0.3 | 0.19 | 0.74 | 1.37 | 1.01 | 0.25 | 0.52 | 0.25 | 3.54 | 0.39 | 0.55 | LOC100620211 |
| gene24312 | 1.28356565 | 0.00010111 | 16.19 | 10.99 | 11.61 | 4.44 | 6.96 | 5.65 | 12.74 | 3.88 | 8.13 | 10.38 | 6.64 | 7.41 | LOC100621510 |
| gene2434 | 1.235969932 | 7.10E-05 | 229.1 | 129.67 | 123.34 | 87.61 | 56.37 | 77 | 120.3 | 129.67 | 182.51 | 88.45 | 137.96 | 230.16 | PHLDA2 |
| gene24467 | -5.793960165 | 1.84E-05 | 0 | 0 | 0 | 0.03 | 0 | 0.62 | 0 | 0 | 0.04 | 0 | 0 | 0 | LOC100620789 |
| gene24472 | 3.090242742 | 0.003477837 |  |  |  |  |  |  |  |  |  |  |  |  |  |
| gene24484 | 4.413588672 | 7.27E-34 | 344.17 | 9.88 | 40.06 | 1.82 | 8.5 | 10.46 | 10.92 | 94.21 | 3.62 | 135.09 | 111 | 7.78 | HSP70 |
| gene24485 | 1.139679581 | 0.001313298 | 2.5 | 0.56 | 0.72 | 0.4 | 0.57 | 0.91 | 2.37 | 5.19 | 3.32 | 2.46 | 4.21 | 1.88 | LOC100623207 |
| gene24509 | 1.722675929 | 0.007857094 | 0.51 | 0.28 | 0 | 0.13 | 0.07 | 0.08 | 0.37 | 0.3 | 0.2 | 0.28 | 0.4 | 0.36 | LOC100625723 |
| gene24511 | -3.516251934 | 0.003477837 | 0.04 | 0 | 0 | 0.17 | 0 | 0.13 | 0.01 | 0.06 | 0.13 | 0.06 | 0.09 | 0.05 | LOC100521764 |
| gene24527 | 1.70032035 | 0.004327683 | 0.53 | 0.18 | 0.24 | 0.07 | 0.1 | 0.13 | 0.31 | 0.4 | 0.26 | 0.05 | 0.37 | 0.25 | LOC100627613 |
| gene24558 | 4.20643962 | 3.55E-32 | 419.59 | 9.42 | 52.85 | 5.69 | 10.45 | 13.1 | 10.29 | 105.48 | 6.52 | 163.73 | 124.28 | 5.87 | HSP70 |
| gene24566 | -1.17673452 | 0.005930635 | 0.41 | 0.41 | 0.63 | 0.84 | 1.3 | 1.08 | 0.14 | 0.89 | 0.24 | 0.76 | 0.32 | 0.24 | LOC100623996 |
| gene24576 | -1.290393797 | 0.004208197 |  |  |  |  |  |  |  |  |  |  |  |  |  |
| gene24673 | -4.067712389 | 0.000162544 | 0 | 0 | 0.04 | 0.51 | 0 | 0.12 | 0 | 0 | 0 | 0 | 0 | 0 | LOC100627396 |
| gene24674 | -2.962305102 | 1.07E-10 | 0.37 | 0.14 | 0.2 | 0.75 | 0.35 | 4.45 | 9.01 | 0.04 | 0 | 15.34 | 0.06 | 0.05 | FGA |
| gene24703 | -1.129659119 | 0.000380704 | 12.84 | 12.63 | 16.22 | 31.33 | 32.58 | 32.02 | 19.77 | 45.45 | 22.46 | 31.22 | 20.02 | 27.78 | LOC100622406 |
| gene24715 | 4.607269515 | 0.005613942 |  |  |  |  |  |  |  |  |  |  |  |  |  |
| gene24727 | -2.083699911 | 0.002671838 | 0.03 | 0.01 | 0.02 | 0.15 | 0.06 | 0.05 | 0.03 | 0.01 | 0.04 | 0.05 | 0.03 | 0 | LOC100739447 |
| gene24750 | -1.479673374 | 6.48E-06 | 0.9 | 0.94 | 0.85 | 2.27 | 2.39 | 3.33 | 3.03 | 1.7 | 0.96 | 1.11 | 2.81 | 2.47 |  |
| gene24799 | 1.547372529 | 8.75E-06 | 0.35 | 1.13 | 2.26 | 0.62 | 0.43 | 0.24 | 0.98 | 1.67 | 2.85 | 3.45 | 3.29 | 2.08 | LOC100624264 |
| gene24807 | -1.023767956 | 0.002147911 | 3.3 | 3.09 | 2.01 | 4.08 | 7.04 | 7.28 | 12.46 | 4.93 | 2.77 | 4.23 | 3.63 | 3.67 | RDH10 |
| gene24890 | 1.794968752 | 0.005749948 |  |  |  |  |  |  |  |  |  |  |  |  |  |
| gene24907 | 1.028953576 | 0.007007405 | 5.81 | 1.79 | 2.02 | 1.79 | 1.43 | 1.84 | 2.75 | 1.96 | 2.54 | 3.7 | 0.87 | 1.58 | LOC100627583 |
| gene24922 | -1.557166585 | 0.000468689 | 0.08 | 0.19 | 0.2 | 0.39 | 0.53 | 0.52 | 0.91 | 0.12 | 0.23 | 0.37 | 0.44 | 0.16 | C1RL |
| gene24961 | 1.236454487 | 0.00358333 | 2.8 | 0.58 | 1.5 | 0.56 | 0.96 | 0.6 | 0 | 0.12 | 0 | 0 | 0 | 0 | LOC100523970 |
| gene24997 | -1.498644949 | 0.002321323 | 0.39 | 0.21 | 0.26 | 0.77 | 1.02 | 0.72 | 0.68 | 0.2 | 0.2 | 0.13 | 0.49 | 0.33 | LOC100621282 |
| gene25017 | -1.554370216 | 0.005792782 | 0.31 | 0.2 | 0.2 | 1.01 | 0.35 | 1.02 | 1.25 | 1.12 | 0.71 | 0.17 | 0.32 | 0.29 | LOC100623470 |
| gene25041 | 1.337661463 | 0.001276172 | 0.89 | 1.02 | 0.85 | 0.42 | 0.38 | 0.36 | 0.31 | 0.61 | 0.34 | 0.45 | 0.26 | 0.48 | LOC100737288 |
| gene25051 | -1.434589118 | 0.000824407 | 0.03 | 0.39 | 0.89 | 0.49 | 2.02 | 0.97 | 0.53 | 0.38 | 0.22 | 0.21 | 0.25 | 0.14 | KATNAL2 |
| gene25062 | 1.646510152 | 1.59E-06 |  |  |  |  |  |  |  |  |  |  |  |  |  |
| gene25069 | -1.128577284 | 0.005277941 | 0.04 | 0.1 | 0.31 | 0.29 | 0.36 | 0.31 | 0.14 | 0.28 | 0.11 | 0.18 | 0.07 | 0.09 |  |
| gene25189 | -5.377822274 | 0.000292606 |  |  |  |  |  |  |  |  |  |  |  |  |  |
| gene25190 | 1.084542869 | 0.000489155 | 20.72 | 32.74 | 13.78 | 11.38 | 13.57 | 8.64 | 23.37 | 27.49 | 18.35 | 30.68 | 40.18 | 58.46 | LOC100625277 |
| gene25228 | -1.077684438 | 0.000468953 | 46.25 | 45.83 | 51.76 | 87.28 | 66.2 | 170.48 | 59.95 | 81.69 | 51 | 156.76 | 76.31 | 80.77 | LOC100621533 |
| gene25295 | -1.782806686 | 0.001483053 | 0.11 | 0.12 | 0.25 | 0.27 | 0.83 | 0.61 | 0.03 | 0.56 | 0.27 | 0.62 | 0.18 | 0.22 | ITIH2 |
| gene25320 | -2.157570567 | 0.003094802 | 0 | 0.44 | 0 | 0.63 | 0.82 | 1.8 | 0.29 | 0.56 | 0.93 | 0 | 0 | 0.51 | LOC100623027 |
| gene25328 | 1.445351419 | 0.0003128 | 1.65 | 0.73 | 1.32 | 0.5 | 0.45 | 0.52 | 0.48 | 0.6 | 0.26 | 0.93 | 0.47 | 0.94 | LOC100623811 |
| gene25342 | -2.412208691 | 0.006789857 |  |  |  |  |  |  |  |  |  |  |  |  |  |
| gene25397 | 2.055486004 | 8.81E-09 | 4.07 | 9.64 | 4.47 | 1.36 | 2.04 | 1.31 | 1.79 | 6.37 | 6.95 | 7.76 | 12.23 | 4.53 | LOC100623148 |
| gene25411 | -1.710277784 | 0.005373635 | 0.04 | 0.09 | 0 | 0.14 | 0.19 | 0.24 | 0.08 | 0 | 0.07 | 0.12 | 0.03 | 0 | LOC100624562 |
| gene25420 | -1.090415951 | 0.008025692 | 0.48 | 1.09 | 1.17 | 2.96 | 1.26 | 2.06 | 1.65 | 0.43 | 0.36 | 0.78 | 0.22 | 0.2 | LOC100736973 |
| gene25469 | 1.220718833 | 0.00081979 | 5.61 | 1.12 | 1.51 | 1.59 | 0.96 | 1.33 | 1.22 | 2.77 | 1.74 | 1.28 | 2.82 | 0.94 | LOC100622645 |
| gene25470 | 1.750641172 | 0.003313156 | 0.37 | 0.56 | 0.23 | 0.16 | 0.2 | 0 | 0.19 | 0.21 | 0 | 0.22 | 0.21 | 0.1 | LOC100738290 |
| gene25512 | 4.093592447 | 6.09E-32 | 286.21 | 10.38 | 37.17 | 5.38 | 9.12 | 7.31 | 8.76 | 43.41 | 5.45 | 101.19 | 66.23 | 4.87 | HSP70 |
| gene25558 | 3.157464528 | 0.00586914 | 0.31 | 0.56 | 0.1 | 0.06 | 0.09 | 0.08 | 0.06 | 0.4 | 0.13 | 0.12 | 0.24 | 0 | LOC733664 |
| gene256 | -1.381022946 | 0.000682289 | 0.32 | 0.47 | 0.72 | 1.43 | 0.85 | 1.79 | 0.28 | 0.8 | 0.98 | 0.68 | 0.76 | 0.28 | LOC100622458 |
| gene25624 | -1.076194022 | 0.000819084 | 3.9 | 6.5 | 3.02 | 9.26 | 7.59 | 13.7 | 5.4 | 12.81 | 5.33 | 7.16 | 8.12 | 3.62 | LOC100622882 |
| gene25635 | -1.395187948 | 0.000171793 | 0.1 | 0.54 | 0.2 | 0.42 | 0.95 | 1.14 | 1.07 | 0.76 | 1.95 | 1.1 | 0.61 | 1.4 | LOC100624183 |
| gene2565 | 1.448988825 | 5.12E-06 | 30.51 | 30.27 | 23.75 | 7.96 | 5.89 | 19.66 | 25.58 | 42.63 | 29.79 | 28.75 | 63.67 | 136.55 | LOC100525205 |
| gene25673 | 1.780188729 | 1.46E-08 | 257.95 | 36.24 | 36.91 | 38.81 | 34.44 | 33.64 | 28.23 | 29.85 | 30.82 | 183.14 | 28.25 | 22.05 | LOC100627777 |
| gene25697 | -1.426741386 | 0.000150129 | 1.01 | 0.6 | 0.85 | 3 | 2.32 | 1.47 | 1.41 | 2.71 | 1 | 1.26 | 1.38 | 0.58 | LOC100622048 |
| gene25713 | -1.656824216 | 2.14E-07 | 11.77 | 14.33 | 9.2 | 64.49 | 4.74 | 51.01 | 9.18 | 21.03 | 9.21 | 17.95 | 12.21 | 12.3 | ISG15 |
| gene25735 | -2.853716093 | 0.002067442 | 0.08 | 0.05 | 0 | 0.45 | 0.71 | 0 | 0.25 | 0.24 | 0.41 | 0.25 | 0 | 0.11 | LOC100626019 |
| gene25744 | 2.963940971 | 0.001088723 | 2.34 | 0.07 | 0 | 0 | 0 | 0.32 | 0.72 | 1.75 | 0 | 0.36 | 1.13 | 0.32 | LOC100737995 |
| gene25757 | 1.463405757 | 0.009309076 | 0.24 | 0.48 | 0.43 | 0.07 | 0.25 | 0.14 | 0.14 | 0.06 | 0.3 | 0.19 | 0.09 | 0.04 | DQX1 |
| gene25771 | 1.061121342 | 0.000675634 | 20 | 28.95 | 30.24 | 12.76 | 9.49 | 17.96 | 12.85 | 27.19 | 19.75 | 22.36 | 32.54 | 25.07 | LOC100621245 |
| gene25827 | 2.400534233 | 0.00020464 | 0.8 | 3.03 | 0 | 0.4 | 0.4 | 0 | 0.46 | 0.24 | 0.2 | 0 | 0 | 0.16 | LOC396781 |
| gene25830 | 1.269652862 | 5.37E-05 | 52.7 | 63.73 | 74.93 | 32.01 | 28.03 | 23.28 | 25.62 | 26.97 | 30.18 | 67.33 | 32.12 | 34.3 | CKB |
| gene25843 | 4.75718093 | 0.005613942 | 0 | 0.58 | 0 | 0 | 0 | 0 | 0.06 | 0 | 0.27 | 0 | 1.11 | 0 | CYP2C36 |
| gene25870 | -1.055447243 | 0.008223501 | 0.45 | 0.82 | 0.62 | 0.79 | 1.53 | 1.88 | 1.15 | 0.46 | 0.22 | 0.34 | 1.46 | 1.38 | LOC100622943 |
| gene25910 | -1.245619724 | 8.94E-05 | 5.7 | 4.34 | 5.25 | 7.18 | 17.19 | 13.92 | 7.18 | 3.11 | 3.22 | 2.71 | 3.81 | 4.16 | ENOSF1 |
| gene25918 | 1.44175101 | 6.36E-06 | 33.72 | 86.6 | 127.8 | 27.3 | 27.83 | 39.97 | 53.23 | 53.84 | 31.51 | 40.53 | 54.86 | 20.56 | LOC100627868 |
| gene25939 | -1.182851 | 0.000374818 | 1.81 | 1.71 | 2.81 | 4.13 | 5.06 | 5.72 | 6.37 | 4.18 | 5.49 | 1.91 | 3.74 | 5.36 | LOC100621841 |
| gene2594 | 1.543054533 | 0.003449343 | 0.21 | 0.25 | 0.64 | 0.16 | 0.15 | 0.04 | 0.45 | 0.48 | 0.79 | 0.57 | 0.3 | 0.34 | LOC100737751 |
| gene25951 | -1.092595614 | 0.000407047 | 24.61 | 12.22 | 24.23 | 33.2 | 72.15 | 31.74 | 27.95 | 10.5 | 13.71 | 13.23 | 12.01 | 10.59 |  |
| gene26026 | -1.152709389 | 0.000178366 | 297.89 | 520.63 | 827.55 | 721.23 | 992.63 | 2118.1 | 1497.5 | 54.32 | 83.89 | 147.27 | 320.02 | 96.51 | LOC100738609 |
| gene261 | -1.297773087 | 0.002320124 | 0.16 | 0.26 | 0.14 | 0.47 | 0.65 | 0.39 | 0.32 | 0.29 | 0.29 | 0.71 | 0.29 | 0.18 | LOC100512321 |
| gene26115 | 1.247873769 | 0.00836245 | 1.8 | 1.13 | 2.16 | 1.04 | 0.66 | 0.57 | 0.39 | 0.96 | 0.43 | 1.31 | 0.51 | 0.81 | LOC100622851 |
| gene26128 | 1.288746726 | 3.31E-05 | 39.57 | 63.09 | 35.3 | 17.14 | 21.56 | 21.08 | 27.11 | 23.61 | 42.56 | 33.08 | 38.62 | 53.54 | LDLR |
| gene26140 | 1.109992511 | 0.001164501 | 10.6 | 6.83 | 7.96 | 5.5 | 4.01 | 3.04 | 6.05 | 2.21 | 3.83 | 1.45 | 0.38 | 1.62 | LOC100625729 |
| gene26141 | 1.606998335 | 0.004497305 | 0.48 | 0.5 | 0.25 | 0.07 | 0.18 | 0.23 | 0.35 | 0.69 | 0.65 | 0.61 | 0.1 | 0.25 | GPR19 |
| gene26154 | 2.492425904 | 1.44E-05 | 0.72 | 0.6 | 0.19 | 0.13 | 0 | 0.17 | 0.37 | 0 | 0 | 0.47 | 0 | 0 | LOC100626979 |
| gene2616 | 4.77460445 | 1.59E-11 | 0.85 | 5.49 | 0.39 | 0.18 | 0.19 | 0 | 0 | 1.06 | 0 | 5.2 | 0.02 | 0.99 | LOC100516058 |
| gene26178 | 1.345737641 | 1.66E-05 | 139.36 | 184.31 | 90.64 | 50.16 | 60.7 | 62.8 | 71.13 | 99.77 | 133.78 | 103.68 | 120.07 | 119.94 | LDLR |
| gene26188 | 1.961775812 | 4.43E-10 | 13133 | 1662 | 2459.9 | 1399.5 | 2034.1 | 1447.8 | 1196.5 | 2906 | 1800.5 | 8226.3 | 4202.7 | 1884.1 | HSP70.2 |
| gene26189 | 1.094098735 | 0.001032492 | 5.15 | 1.08 | 1.7 | 1.27 | 1.32 | 1.42 | 1.85 | 1.51 | 1.91 | 5.96 | 1.59 | 1.92 | HSPA1L |
| gene2628 | 1.625735929 | 0.000154986 | 0.18 | 0.24 | 0.35 | 0.08 | 0.09 | 0.1 | 0.09 | 0.16 | 0.27 | 0.24 | 0.14 | 0.13 | LOC100524236 |
| gene2664 | 1.485000478 | 0.00539841 | 0.5 | 0.92 | 0.43 | 0.31 | 0 | 0.47 | 0.39 | 0.19 | 0.19 | 0.12 | 0.17 | 0.79 | LOC100627034 |
| gene2666 | 1.566275136 | 8.18E-05 | 0.88 | 0.95 | 1.85 | 0.19 | 0.26 | 0.92 | 0.04 | 0.02 | 0.88 | 0.68 | 0.46 | 0.62 | LOC100737764 |
| gene2667 | 3.72510612 | 8.13E-24 | 3.98 | 13.7 | 6.4 | 0.61 | 0.19 | 1.23 | 0.09 | 1.11 | 2.1 | 4.8 | 5.55 | 10.27 | LOC100737802 |
| gene2669 | -2.318668175 | 1.21E-11 | 0.66 | 1.3 | 0.24 | 0.11 | 0.16 | 12.45 | 0.52 | 0.12 | 0.16 | 0.07 | 0.23 | 0.2 | LOC100737875 |
| gene2670 | 1.467418755 | 0.005307024 | 0.26 | 0.69 | 0.22 | 0.08 | 0.16 | 0.23 | 0.39 | 0.19 | 0.2 | 0.15 | 0.11 | 0 | SLC22A8 |
| gene2672 | -1.651193429 | 0.005833731 |  |  |  |  |  |  |  |  |  |  |  |  |  |
| gene2705 | -3.221886062 | 2.99E-11 | 0 | 0.25 | 2.6 | 5.32 | 0.74 | 16.16 | 0.11 | 0.28 | 0.16 | 1.13 | 0 | 2.38 | PHEROC |
| gene2717 | 1.241271603 | 0.002206476 | 0.37 | 0.37 | 0.41 | 0.19 | 0.21 | 0.13 | 0.1 | 0.3 | 0.14 | 0.2 | 0.14 | 0.26 | LOC100520659 |
| gene2756 | 2.955961222 | 0.00586914 | 0.92 | 0.56 | 0.73 | 0.22 | 0 | 0 | 0 | 0 | 0.64 | 0 | 0 | 0.26 | LOC100738187 |
| gene2847 | -2.251492635 | 0.000658779 | 0.04 | 0.05 | 0.21 | 0.69 | 0.23 | 0.4 | 0.38 | 0.21 | 0.69 | 0 | 0.37 | 0.79 | LOC100513068 |
| gene2852 | -2.214756346 | 0.003790376 | 0.16 | 0 | 0.1 | 0.19 | 0.61 | 0.34 | 0.25 | 0.17 | 0.27 | 0.32 | 0.21 | 0.45 | LOC100514032 |
| gene2894 | -4.530726949 | 6.71E-13 |  |  |  |  |  |  |  |  |  |  |  |  |  |
| gene2992 | 1.241670197 | 9.58E-05 | 2.36 | 2.4 | 3.26 | 1.01 | 1 | 1.6 | 0.72 | 1.15 | 1.23 | 2.37 | 1.67 | 2.79 |  |
| gene3003 | -1.388294109 | 8.65E-06 | 8.49 | 12.62 | 13.84 | 16.93 | 49.08 | 29.27 | 35.6 | 8.64 | 9.41 | 9.07 | 12.21 | 12.08 | CREB3L1 |
| gene3012 | -1.006393126 | 0.009161505 | 0.9 | 0.7 | 0.67 | 1.31 | 2.08 | 1.47 | 0.51 | 0.83 | 0.15 | 0.4 | 0.57 | 0.43 | LOC100623533 |
| gene3051 | 1.202954854 | 9.98E-05 | 104.08 | 134.71 | 123.61 | 45.81 | 44.11 | 77.21 | 37.48 | 89.98 | 80.93 | 99.23 | 94.63 | 129.06 | LOC100526094 |
| gene3126 | 1.815465154 | 0.000174454 | 0.51 | 0.13 | 0.93 | 0.08 | 0.22 | 0.14 | 0.09 | 0.44 | 0.2 | 0.15 | 0.55 | 0.64 | PTPN5 |
| gene3138 | -2.50215009 | 1.39E-05 | 0.1 | 0.12 | 1.02 | 3.94 | 0 | 2.21 | 4.16 | 0 | 0 | 1.25 | 0.6 | 0.14 | LOC100525856 |
| gene3244 | 1.866915359 | 5.25E-06 | 0.17 | 2.13 | 0.3 | 0.49 | 0.16 | 0.17 | 0.84 | 0.18 | 2.07 | 1.94 | 0.98 | 4.05 | LOC100525099 |
| gene3277 | 2.78624029 | 0.009931868 | 0.24 | 0 | 0.07 | 0.01 | 0.03 | 0 | 0.06 | 0.03 | 0 | 0.09 | 0 | 0 | TRIM58 |
| gene3304 | 2.228901829 | 0.005037748 |  |  |  |  |  |  |  |  |  |  |  |  |  |
| gene3313 | -1.599468741 | 0.000490064 | 0.44 | 0.57 | 0.5 | 2.6 | 0.87 | 1.62 | 0.73 | 1.64 | 0.6 | 0 | 0.6 | 0.76 | LOC100515190 |
| gene3322 | 1.716542182 | 0.003488584 | 0 | 0.31 | 0.14 | 0.05 | 0.08 | 0.03 | 0.06 | 0.02 | 0.1 | 0.05 | 0.03 | 0.12 | CILP2 |
| gene3336 | -1.735381536 | 3.04E-07 | 0.73 | 0.85 | 1.04 | 5.5 | 2.31 | 1.21 | 2.05 | 0.47 | 2.08 | 0.46 | 0.4 | 1.44 | COMP |
| gene3362 | -1.35994114 | 0.005215015 | 0.16 | 0.18 | 0.1 | 0.42 | 0.7 | 0 | 0.25 | 0.58 | 0.24 | 0.06 | 0.12 | 0.17 | SLC5A5 |
| gene3401 | -1.418878402 | 0.000246421 | 0.21 | 0.79 | 0.84 | 3.12 | 0.4 | 1.6 | 0.2 | 0.34 | 0.12 | 0.11 | 0.22 | 0.2 | HSH2D |
| gene3422 | 3.202988076 | 4.82E-07 | 0.06 | 0.79 | 0.52 | 0 | 0 | 0.17 | 0.18 | 0.12 | 0.12 | 0.27 | 0.16 | 0.04 | LOC100620265 |
| gene351 | -1.115265957 | 0.000882422 | 1.62 | 1.32 | 0.59 | 4.33 | 0.83 | 3.22 | 4.52 | 3.44 | 2.56 | 2.64 | 3.19 | 2.96 | LOC100157995 |
| gene3553 | 1.856682811 | 4.09E-09 | 138.75 | 22.39 | 30.52 | 20.18 | 20.51 | 17.55 | 16.16 | 15.34 | 16.77 | 91.73 | 20.39 | 12.4 | DNAJB1 |
| gene3557 | -1.170160666 | 0.005044899 | 1.38 | 0.9 | 1.86 | 2.7 | 3.34 | 3.21 | 1.85 | 0.6 | 1.73 | 0.8 | 0.52 | 0.77 | LOC100624148 |
| gene3559 | -1.045207755 | 0.003749264 | 0.81 | 2.43 | 1.08 | 1.24 | 2.04 | 6.82 | 2.82 | 1.5 | 3.79 | 1.32 | 0.79 | 0.53 | ASF1B |
| gene363 | -1.591723648 | 0.004467375 | 0.04 | 0.1 | 0.61 | 0.94 | 0.22 | 0.74 | 0.52 | 1.02 | 0.47 | 0.87 | 0.83 | 1.15 | LOC100517621 |
| gene37 | -1.505662353 | 8.67E-06 | 3.39 | 3.08 | 2.92 | 14.89 | 6.98 | 6.32 | 6.97 | 9.3 | 11.17 | 2.96 | 7.41 | 3.14 | PARK2 |
| gene3767 | 1.131264647 | 0.000290113 | 39.36 | 56.41 | 32.86 | 17.54 | 22.34 | 22.47 | 31.08 | 30.41 | 53.46 | 36.23 | 52.75 | 64.01 | LDLR |
| gene3782 | -1.070647456 | 0.000599395 | 14.26 | 12.39 | 18.99 | 22.58 | 19.05 | 60.56 | 20.39 | 20.04 | 12.67 | 59.24 | 27.1 | 32.75 | CNN1 |
| gene3785 | -1.1763084 | 0.00022451 | 12.49 | 21.81 | 28.22 | 24.66 | 42.97 | 80.72 | 79.05 | 2.99 | 4.24 | 6.49 | 22.38 | 5.1 | UF |
| gene38 | -1.050918636 | 0.000973425 | 11.99 | 5.13 | 8.59 | 31.31 | 14.31 | 10.98 | 9.24 | 24.13 | 29.96 | 11.1 | 12.71 | 7.23 | PARK2 |
| gene3830 | -1.51871621 | 1.46E-06 | 12.32 | 16.71 | 14.97 | 40.2 | 18.03 | 76.55 | 26.74 | 32.03 | 22.86 | 31.69 | 29.81 | 32.11 | CD209 |
| gene386 | -1.072617183 | 0.000831563 | 4.1 | 4.01 | 3.97 | 6.89 | 14.67 | 4.99 | 11.3 | 6.03 | 6.34 | 6.61 | 4.28 | 4.79 | RRAGD |
| gene3864 | 1.094715543 | 0.002846023 | 4.64 | 2.17 | 1.17 | 1.63 | 1.21 | 1.28 | 0.84 | 1.95 | 4.52 | 2.83 | 2.29 | 3.86 | LOC100736831 |
| gene3942 | 2.086274673 | 0.005373635 | 0 | 0.77 | 0.38 | 0.23 | 0.06 | 0 | 1.71 | 0.25 | 0.17 | 0.16 | 1.37 | 0.28 | LOC100737370 |
| gene3955 | 1.002015649 | 0.00260057 | 3.45 | 4.55 | 3.33 | 1.49 | 1.85 | 2.75 | 2.32 | 3.33 | 2.88 | 4.25 | 4.35 | 3.89 | LOC100516355 |
| gene4029 | -1.40426808 | 0.005574518 | 0.14 | 0.15 | 0.05 | 0.5 | 0.31 | 0.24 | 0.08 | 0.23 | 0.18 | 0.1 | 0.44 | 0.06 | LOC100523719 |
| gene4112 | 1.26672528 | 0.00024257 | 0.87 | 1.67 | 1.09 | 0.47 | 0.57 | 0.59 | 0.59 | 0.57 | 0.87 | 0.71 | 0.92 | 1.14 | LOC100525508 |
| gene4181 | 1.258177937 | 0.000159501 | 8.63 | 10.54 | 7.45 | 3.24 | 4.28 | 4.34 | 2.15 | 8.17 | 9.43 | 17.16 | 10.67 | 17.76 | HOMER1 |
| gene4182 | 2.248703184 | 1.84E-06 | 3 | 4 | 2.66 | 0.12 | 0.73 | 1.51 | 0.82 | 1.36 | 2.4 | 3.95 | 0.64 | 3.99 | HOMER1 |
| gene4190 | 1.529079362 | 3.49E-05 | 2.36 | 5.3 | 3.27 | 1.55 | 1.63 | 0.85 | 1.87 | 1.51 | 1.87 | 3.83 | 2.7 | 2.38 | LOC100737687 |
| gene4201 | 3.19700025 | 0.002067442 | 0.13 | 0.08 | 0.34 | 0.07 | 0 | 0 | 0.18 | 0.44 | 0.06 | 0.84 | 0.2 | 0.37 | ACOT12 |
| gene4227 | -1.587598965 | 0.002094939 | 0.29 | 0.35 | 0.15 | 0.54 | 1.06 | 1.16 | 0.23 | 0.48 | 0.1 | 1.03 | 0.18 | 0.66 |  |
| gene4252 | -1.805576898 | 0.000269438 | 0.11 | 0.17 | 0.15 | 0.52 | 0.23 | 0.89 | 0.52 | 0.56 | 0.54 | 0.5 | 0.88 | 0.24 | LOC100522665 |
| gene4348 | 1.248429204 | 0.000160454 | 5.07 | 2.32 | 6.94 | 2.05 | 2.42 | 1.88 | 7.52 | 3.65 | 3.68 | 1.53 | 3.58 | 6.41 | LOC100525452 |
| gene4350 | -1.090907997 | 0.000768069 | 2.36 | 1.52 | 6.14 | 5.52 | 2.11 | 14.69 | 1.41 | 1.77 | 0.93 | 1.51 | 0.71 | 2.26 | SNCAIP |
| gene4384 | -1.10378328 | 0.000343843 | 23.1 | 13.6 | 43.53 | 30.42 | 90.37 | 58.5 | 24.14 | 16.25 | 21.16 | 12.78 | 19.53 | 34.2 |  |
| gene4443 | 1.914460484 | 1.18E-09 | 462.77 | 1168 | 525.78 | 191.87 | 230.21 | 178.53 | 235.65 | 725.24 | 866.07 | 835.63 | 460.03 | 486.47 | CXCL14 |
| gene4444 | -1.927499372 | 1.52E-08 | 3.31 | 1.27 | 1.5 | 5.12 | 12.56 | 6.66 | 1.34 | 5.1 | 3.45 | 2.1 | 1.4 | 2.72 | LOC100518721 |
| gene4477 | 1.485216187 | 1.82E-06 | 60.33 | 88.45 | 78.22 | 24.6 | 36.37 | 24.07 | 79.67 | 76.67 | 94.25 | 81.23 | 79.79 | 262.87 | EGR1 |
| gene4488 | -1.066959769 | 0.000830509 | 2.2 | 3.15 | 3.83 | 3.92 | 11.04 | 4.97 | 6.26 | 2.52 | 3.84 | 3.95 | 3.85 | 3.91 | SVCT1 |
| gene4527 | -2.250098457 | 0.001653103 |  |  |  |  |  |  |  |  |  |  |  |  |  |
| gene4541 | 1.01446179 | 0.003030131 | 1.9 | 1.69 | 1.99 | 0.84 | 0.69 | 1.43 | 0.65 | 1.3 | 0.65 | 0.74 | 0.34 | 0.69 | LOC100514339 |
| gene4542 | 1.206684199 | 0.000428213 | 1.74 | 1.78 | 1.98 | 0.57 | 1.15 | 0.81 | 0.35 | 0.63 | 0.98 | 0.39 | 0.61 | 0.92 | LOC100521612 |
| gene4545 | 1.369259751 | 0.000182325 | 1.28 | 2.13 | 1.73 | 0.37 | 0.78 | 1.04 | 0.55 | 1.29 | 0.98 | 2.61 | 0.57 | 2.16 | LOC100739340 |
| gene4546 | 1.199839277 | 0.002045614 | 0.8 | 1.24 | 0.74 | 0.14 | 0.81 | 0.34 | 0.22 | 0.46 | 0.24 | 0.8 | 0.43 | 0.81 | PCDHB16 |
| gene4559 | 1.509654897 | 0.002280082 |  |  |  |  |  |  |  |  |  |  |  |  |  |
| gene4609 | -1.712184106 | 9.56E-08 | 2.84 | 5.88 | 3.92 | 28.86 | 10.27 | 4.45 | 36.19 | 7.48 | 6.08 | 2.63 | 9.19 | 7.26 | PPP2R2B |
| gene4610 | -1.646099188 | 0.002094939 | 0.58 | 0.48 | 0.26 | 3.61 | 0.67 | 0.42 | 4.23 | 0.75 | 0.58 | 0.83 | 1.53 | 0.87 | LOC100621989 |
| gene4632 | -1.173935195 | 0.000537828 | 1.03 | 0.41 | 0.44 | 2.26 | 0.98 | 1.32 | 1.81 | 1.35 | 1.09 | 1.98 | 1.17 | 1 | SH3TC2 |
| gene465 | -1.716393984 | 1.23E-07 | 4.34 | 3.12 | 9.12 | 14.33 | 28.51 | 12.83 | 12.14 | 6.52 | 3.66 | 9.91 | 6.52 | 7.56 | PPIL6 |
| gene475 | -1.742401358 | 9.58E-07 | 2.13 | 0.5 | 1.24 | 3.46 | 7.31 | 2.56 | 3.57 | 2.55 | 1.74 | 1.57 | 1.41 | 0.73 | LOC100736631 |
| gene4853 | -1.314946386 | 3.23E-05 | 8.36 | 4.38 | 3.87 | 20.89 | 9.83 | 13.87 | 12.22 | 3.96 | 3.13 | 3.66 | 3.7 | 1.91 | LOC100522669 |
| gene4858 | 1.458350211 | 3.16E-06 | 144.81 | 48.47 | 68.32 | 40.42 | 32.98 | 29.64 | 20.72 | 28.43 | 34.68 | 51.35 | 29.25 | 12.49 | LOC100521185 |
| gene4911 | 2.170356659 | 9.42E-11 | 7.64 | 8.55 | 5.28 | 1.58 | 1.75 | 1.8 | 2.87 | 0.71 | 4.21 | 10.21 | 13.55 | 8.51 | LOC100521613 |
| gene4913 | -1.151926953 | 0.006851415 | 0.32 | 0.34 | 0.31 | 0.44 | 1.24 | 0.59 | 0.58 | 0.3 | 0.35 | 0.89 | 0.27 | 0.58 | TMEM130 |
| gene4920 | -2.140330098 | 0.000711388 | 0 | 0.09 | 0 | 0.28 | 0.06 | 0.36 | 0.02 | 0 | 0 | 0.24 | 0.04 | 0.07 | KPNA7 |
| gene4946 | -1.179860687 | 0.00013896 | 26.2 | 32.93 | 25.82 | 48.13 | 69.68 | 86.25 | 76.73 | 18.55 | 20.09 | 69.41 | 31.34 | 10.46 | LOC100519648 |
| gene5086 | -1.421334513 | 0.007154103 | 0.57 | 0.36 | 0.19 | 1.44 | 0.9 | 1.13 | 1.38 | 1.31 | 0.74 | 0.79 | 0.23 | 0.21 | LOC100625623 |
| gene5098 | -1.66979211 | 9.62E-07 | 1.25 | 1.43 | 0.92 | 2.09 | 7.28 | 2.65 | 1.62 | 1.34 | 0.94 | 0.95 | 0.14 | 0.42 | 14-Sep |
| gene5126 | -1.093652691 | 0.007160613 | 0.43 | 0.65 | 0.42 | 1.65 | 1.12 | 0.7 | 0.3 | 0.9 | 0.72 | 0.79 | 1.08 | 0.45 | LOC100512651 |
| gene5177 | 5.387276764 | 9.47E-05 | 0.25 | 0.19 | 0.4 | 0 | 0 | 0 | 0.12 | 0.48 | 0.35 | 0.17 | 0.08 | 0.15 | LOC100625929 |
| gene5186 | -2.029397559 | 4.35E-10 | 8.24 | 0.99 | 9.87 | 15.02 | 52.1 | 13.34 | 32.4 | 1.39 | 0.78 | 2.94 | 5.59 | 1.43 | LOC100739420 |
| gene5201 | -1.041591281 | 0.005607726 |  |  |  |  |  |  |  |  |  |  |  |  |  |
| gene5202 | -1.752893252 | 2.84E-05 | 0.79 | 0.1 | 0.81 | 1.25 | 1.13 | 3.18 | 0.97 | 2.88 | 1.78 | 2 | 1.25 | 0.93 | HS3ST2 |
| gene5220 | -1.285503276 | 0.008518916 | 0.11 | 0.15 | 0.11 | 0.43 | 0.2 | 0.3 | 0.54 | 0.62 | 0.37 | 0.28 | 0.16 | 0.4 | ZP2 |
| gene5231 | 4.022551598 | 2.22E-16 | 0.02 | 4.14 | 0 | 0 | 0.03 | 0.3 | 0 | 0.32 | 0.08 | 0.39 | 0.83 | 0 | ACSM2B |
| gene5241 | -2.861009133 | 0.007905898 | 0.05 | 0.05 | 0.11 | 0.7 | 0.3 | 0.35 | 0.26 | 1.06 | 0.28 | 0.52 | 0.76 | 0.5 | LOC100624180 |
| gene5270 | -1.041066535 | 0.00527085 | 2.15 | 3.53 | 3.04 | 6.32 | 5.42 | 7.57 | 4.75 | 1.55 | 4 | 1.53 | 0.42 | 1.92 | PRM2 |
| gene5359 | 1.12477836 | 0.000274856 | 144.71 | 192.87 | 226.87 | 111.48 | 69.98 | 91.35 | 116.02 | 184.96 | 175.85 | 170.7 | 194.38 | 202.32 | TNFRSF12A |
| gene5364 | -3.181036104 | 7.61E-07 | 0.02 | 0 | 0.12 | 0.04 | 0.74 | 0.17 | 0.37 | 0.05 | 0.06 | 0.17 | 0.03 | 0 | ZSCAN10 |
| gene5372 | -1.146371607 | 0.005692322 | 1.43 | 1.23 | 1.56 | 4.92 | 2.7 | 1.82 | 1.68 | 2.2 | 1.17 | 3.67 | 1.59 | 1.81 | FLYWCH2 |
| gene5421 | -1.047356519 | 0.002656938 | 0.77 | 0.86 | 1.14 | 1.23 | 2.22 | 2.56 | 1.16 | 0.63 | 0.09 | 0.78 | 0.43 | 0.86 | IGFALS |
| gene546 | 1.071077097 | 0.000503758 | 179.34 | 72.76 | 62.8 | 50.45 | 46.06 | 66.92 | 70.63 | 69.49 | 89.88 | 100.57 | 124.65 | 113.44 | LOC100156873 |
| gene5483 | 1.853797685 | 1.40E-08 | 12.38 | 9.14 | 1.56 | 1.21 | 1.6 | 4.3 | 2.35 | 2.45 | 3.26 | 0.96 | 2.87 | 2.28 | LOC100738476 |
| gene5511 | -1.294672217 | 8.33E-05 | 1.05 | 1.12 | 1.84 | 4.93 | 3.02 | 2.22 | 1.39 | 3.87 | 2.01 | 1.66 | 1.29 | 1.34 | LOC100513144 |
| gene5524 | 1.855530213 | 8.39E-07 | 1.53 | 6.22 | 3.18 | 1.31 | 0.32 | 1.69 | 0.3 | 2.68 | 1.79 | 2.38 | 3.16 | 1.86 | IL1A |
| gene5563 | -2.565539101 | 2.07E-07 | 0.25 | 0.15 | 0.44 | 0.77 | 2.7 | 1.15 | 0.99 | 0.39 | 0.25 | 0.15 | 0.15 | 0 | LOC100524797 |
| gene5564 | 1.594877843 | 0.003488584 |  |  |  |  |  |  |  |  |  |  |  |  |  |
| gene557 | -1.518485243 | 0.0023315 | 1.33 | 0.41 | 1.59 | 0.62 | 5.95 | 2.88 | 0.75 | 2.22 | 1.16 | 1.08 | 0.42 | 1.14 | LOC100511295 |
| gene5646 | -1.643691863 | 1.03E-05 | 0.1 | 0.31 | 0.51 | 0.42 | 0.57 | 1.97 | 1 | 0.13 | 0.07 | 0.36 | 0.52 | 0.28 | VWA3B |
| gene5648 | -1.421778943 | 0.002325444 | 0.12 | 0.15 | 0.33 | 0.27 | 0.83 | 0.5 | 0.47 | 0.3 | 0.25 | 0.13 | 0.13 | 0.06 | LOC100739325 |
| gene5696 | -1.446611583 | 0.000389299 | 0.61 | 0.9 | 0.36 | 0.74 | 3.5 | 1.16 | 0.73 | 1.35 | 0.29 | 0.72 | 1.67 | 0.63 | LOC100522432 |
| gene5709 | 3.567114527 | 1.13E-05 | 2.38 | 0.1 | 0.22 | 0 | 0 | 0.24 | 0.53 | 0.69 | 1.87 | 0.13 | 0.39 | 0.59 | LOC100518605 |
| gene5739 | 1.130432841 | 0.000479993 | 4.83 | 5.8 | 5.33 | 2.45 | 2.92 | 2.34 | 1.17 | 3.89 | 1.54 | 4.02 | 4.21 | 2.82 | CTNNA2 |
| gene5742 | 1.20739675 | 0.000151873 | 11.18 | 2.83 | 69.63 | 9.46 | 7.17 | 20.65 | 504.54 | 6.28 | 11.44 | 0 | 4.54 | 3.01 | LOC100520832 |
| gene5745 | 1.758903336 | 1.95E-07 | 2.49 | 1.63 | 39.44 | 4.31 | 2.57 | 6.18 | 247.11 | 2.05 | 4.19 | 0 | 0.94 | 2.19 | REG3G |
| gene5785 | -1.227858097 | 7.52E-05 | 37.52 | 36.58 | 44.06 | 63.69 | 39.52 | 193.4 | 42.39 | 80.02 | 43.75 | 172.16 | 53.36 | 69.39 | LOC100520667 |
| gene5786 | -1.198444713 | 0.000356107 | 5.3 | 7.27 | 7.52 | 8.85 | 5.17 | 35.8 | 7.24 | 7.21 | 5.83 | 28.82 | 10.24 | 7.35 | LOC100621129 |
| gene5807 | -1.592194375 | 0.000403076 |  |  |  |  |  |  |  |  |  |  |  |  |  |
| gene5816 | -1.496283464 | 4.57E-05 |  |  |  |  |  |  |  |  |  |  |  |  |  |
| gene5906 | 1.074711995 | 0.000560319 | 32.35 | 9.19 | 7.5 | 9.53 | 7.39 | 8.68 | 12.01 | 13.16 | 9.58 | 10.56 | 21.69 | 13.9 | REL |
| gene5941 | -1.192574611 | 0.000179354 | 1.83 | 3.08 | 4.26 | 8.2 | 4.57 | 9.17 | 3.87 | 0.66 | 6.08 | 3.17 | 0.7 | 2.25 | GPR115 |
| gene5998 | 1.021812388 | 0.000865726 | 322.32 | 366.06 | 352.86 | 142.21 | 192.46 | 207.69 | 111.49 | 200.17 | 222.16 | 345.39 | 237.68 | 470.09 | EPAS1 |
| gene6018 | 1.755382294 | 2.78E-06 | 5.31 | 0.38 | 0.5 | 0.54 | 0.2 | 1.38 | 0.74 | 1.03 | 0.05 | 0.84 | 1.5 | 0.41 | KCNG3 |
| gene602 | -1.875654718 | 2.63E-09 | 19.86 | 17.45 | 18.35 | 43.24 | 134.22 | 36.24 | 49.38 | 54.42 | 46.17 | 21.08 | 59.11 | 28.61 | LIPG |
| gene6024 | -1.04109011 | 0.006392081 | 1.49 | 0.88 | 0.83 | 2.72 | 2 | 2.23 | 4.66 | 3.11 | 1.51 | 2.04 | 2.05 | 1.65 | LOC100738444 |
| gene6026 | -1.489229431 | 0.00132141 | 0.5 | 0.39 | 0.24 | 1.29 | 1.42 | 0.66 | 0.73 | 0.05 | 0.72 | 0.3 | 0.29 | 0.07 | LOC100738491 |
| gene6028 | -1.491946255 | 0.007707738 |  |  |  |  |  |  |  |  |  |  |  |  |  |
| gene6065 | -1.831831252 | 0.000115197 | 0.11 | 0.09 | 0.31 | 1.1 | 0.12 | 0.56 | 0.24 | 0.11 | 0.37 | 0.24 | 0.27 | 0.6 | VIT |
| gene6102 | 1.154385355 | 0.003902352 | 0.63 | 0.18 | 0.2 | 0.19 | 0.2 | 0.08 | 0.58 | 0.9 | 0.32 | 0.67 | 1.01 | 0.51 | ALK |
| gene6133 | -1.578837272 | 3.17E-05 | 0.58 | 1.38 | 1.3 | 5.55 | 2.73 | 2.15 | 2.37 | 0.48 | 1.32 | 1.35 | 1.18 | 0.74 | LOC100625689 |
| gene6169 | 1.605769624 | 2.28E-06 | 2.42 | 3.58 | 3.25 | 0.78 | 0.66 | 1.82 | 0.6 | 1.52 | 4.1 | 6.08 | 3.37 | 5.29 |  |
| gene6196 | -1.598533249 | 3.66E-05 | 0.31 | 0.54 | 0.51 | 0.45 | 2.04 | 1.8 | 1.01 | 0.81 | 0.15 | 0.54 | 0.85 | 1.2 | LOC100624534 |
| gene6204 | 1.060640178 | 0.006920854 | 2 | 0.41 | 0.37 | 0.46 | 0.57 | 0.43 | 0.32 | 0.58 | 0.22 | 0.81 | 0.75 | 0 |  |
| gene6264 | -1.868034115 | 1.16E-08 | 5 | 2.79 | 1.72 | 19.21 | 3.25 | 15.53 | 2.32 | 7.29 | 6.72 | 8.71 | 3.64 | 4.52 | IRG6 |
| gene63 | -1.087606212 | 0.000454236 | 2.18 | 3.41 | 2.48 | 1.91 | 7.47 | 8.74 | 3.79 | 0.93 | 0.99 | 3.14 | 2.86 | 2.3 |  |
| gene6434 | 1.084924021 | 0.002362952 | 0.85 | 0.99 | 0.91 | 0.39 | 0.5 | 0.48 | 0.26 | 0.84 | 0.44 | 1 | 0.51 | 0.87 | LOC100622999 |
| gene6436 | -1.539532653 | 0.001044807 | 0.07 | 0.15 | 0.18 | 0.73 | 0.15 | 0.32 | 0.07 | 0.88 | 0.15 | 0.93 | 0.82 | 0.1 | LOC100739348 |
| gene6608 | -1.419267785 | 3.38E-05 |  |  |  |  |  |  |  |  |  |  |  |  |  |
| gene6656 | 5.289886284 | 0.000520123 |  |  |  |  |  |  |  |  |  |  |  |  |  |
| gene6657 | 1.461185922 | 5.07E-06 | 27.79 | 10.4 | 9.09 | 5.4 | 3.75 | 9.79 | 9.25 | 9.7 | 7.78 | 11.07 | 13.3 | 22.52 | ATP6V0D2 |
| gene6658 | 2.202670809 | 4.34E-12 | 191 | 340.52 | 199.72 | 51.67 | 64.9 | 50.72 | 60.66 | 84.46 | 50.53 | 201.08 | 153.16 | 103.49 | CA2 |
| gene666 | -1.113363959 | 0.001366385 | 2.39 | 2.34 | 4.17 | 3.6 | 11.48 | 4.33 | 4.41 | 3.1 | 0.99 | 2.66 | 3.77 | 3.43 | DAPK2 |
| gene6684 | -1.393041225 | 9.63E-05 | 2.26 | 2.09 | 2.15 | 4.53 | 7.77 | 5.41 | 4.87 | 1.66 | 2.2 | 0.07 | 1.77 | 2.15 | IL7 |
| gene6702 | 1.340347942 | 0.000582344 | 1.66 | 1.37 | 1.07 | 0.39 | 0.61 | 0.84 | 1.2 | 1.25 | 0.29 | 0.77 | 1.18 | 0.79 | LOC100626574 |
| gene6711 | -1.104743615 | 0.004540156 | 0.81 | 0.63 | 2.01 | 0.7 | 5.25 | 1.33 | 1.55 | 0.56 | 0.54 | 0.59 | 1.05 | 0.78 | LOC100517890 |
| gene6726 | 2.125648218 | 0.007295709 | 0.26 | 0.15 | 0.21 | 0.03 | 0.09 | 0 | 0.06 | 0.19 | 0.05 | 0.21 | 0.33 | 0.07 | LOC100738208 |
| gene6728 | 2.11922086 | 0.002671838 |  |  |  |  |  |  |  |  |  |  |  |  |  |
| gene68 | -1.505117855 | 0.005655889 | 0.61 | 1.05 | 3.77 | 5.4 | 2.65 | 5.81 | 2.3 | 1.67 | 1.67 | 0.47 | 4.06 | 1.21 | LOC100521937 |
| gene6807 | 1.209675216 | 0.001481753 | 2.7 | 2.45 | 2.42 | 1.16 | 1.08 | 1.27 | 1.67 | 1.9 | 0.64 | 4.99 | 2.38 | 2.1 | LOC100156509 |
| gene6835 | 1.634527311 | 3.40E-07 | 19.05 | 20.75 | 16.03 | 3.83 | 4.13 | 11.48 | 3.52 | 4.66 | 5.41 | 3.07 | 4.14 | 4.92 | SELL |
| gene700 | 1.084741803 | 0.000519412 | 18.83 | 37.56 | 7.91 | 8.82 | 8 | 15.76 | 14.76 | 23.79 | 19.42 | 22.46 | 24.92 | 26.56 | LOC100522112 |
| gene7032 | -1.535012955 | 0.002031086 | 0.04 | 0.15 | 0.05 | 0.43 | 0.17 | 0.24 | 0.17 | 0 | 0.13 | 0.09 | 0.06 | 0 | FCRL3 |
| gene7042 | -1.051558423 | 0.004943925 | 0.65 | 0.43 | 0.83 | 1.19 | 1.25 | 1.63 | 1.45 | 0.71 | 0.51 | 0.57 | 0.57 | 0.54 | LRRC71 |
| gene7046 | 1.001492944 | 0.006292411 | 1.99 | 1.46 | 1.8 | 1.03 | 1.04 | 0.7 | 0.92 | 1.49 | 0.48 | 0.93 | 1.1 | 0.74 | LOC100153265 |
| gene7057 | -1.783775411 | 0.005603004 | 0.07 | 0.11 | 0.1 | 0.12 | 0.76 | 0.32 | 0.19 | 0.08 | 0.06 | 0.12 | 0.06 | 0 | HAPLN2 |
| gene7131 | -1.113047104 | 0.00931949 | 0.13 | 0.23 | 1.14 | 2.07 | 0.46 | 0.51 | 0.12 | 0.97 | 0.53 | 0.57 | 0.55 | 0.4 | CHRNB2 |
| gene717 | -1.021665694 | 0.001077964 | 10.28 | 14.07 | 6.84 | 21.8 | 18.99 | 27.05 | 15.3 | 12.4 | 20.7 | 17.3 | 14.14 | 9.83 | AQP9 |
| gene7198 | -1.633627232 | 1.98E-07 | 16.19 | 5.38 | 11.44 | 14.01 | 12.4 | 85.26 | 41.8 | 8.3 | 5.83 | 1.53 | 7.54 | 6.13 | CRNN |
| gene7255 | 1.050187485 | 0.000626582 | 510.57 | 271.72 | 139.51 | 141.58 | 184.76 | 156.52 | 244.54 | 234.73 | 239.16 | 348.13 | 397.57 | 605.31 | ECM1 |
| gene7269 | 5.62052572 | 1.84E-05 | 1.8 | 0.04 | 0.15 | 0 | 0 | 0 | 0.08 | 0 | 0 | 0.29 | 0 | 0 | LOC100523961 |
| gene7281 | 2.236750454 | 8.37E-09 | 17.01 | 1.02 | 1.34 | 1.15 | 2.29 | 1.09 | 6.38 | 6.35 | 1.33 | 6.09 | 10.82 | 3.39 | LOC100155404 |
| gene7282 | 1.842252862 | 0.001124718 | 2.81 | 0.06 | 1.1 | 0.43 | 0.11 | 0.66 | 1.4 | 0 | 0.65 | 1.26 | 0.73 | 0.26 | LOC100622412 |
| gene7288 | 4.789009113 | 0.0030599 | 0.04 | 0.04 | 0.17 | 0 | 0 | 0 | 0.19 | 0.18 | 0.04 | 0.17 | 0.24 | 0.8 | LOC100738895 |
| gene7317 | 1.207035803 | 0.004034591 | 22.75 | 10.29 | 12.4 | 6.18 | 7.78 | 7.22 | 3.81 | 24.53 | 11.05 | 21.38 | 13.21 | 1.51 |  |
| gene7319 | -1.351749986 | 0.000216442 | 1.08 | 1.49 | 1.69 | 1.82 | 3.98 | 5.56 | 3.13 | 1.68 | 3.44 | 2.53 | 1.81 | 3.16 | LOC100519190 |
| gene7340 | -1.562642721 | 0.00443691 | 0.25 | 0.09 | 0.29 | 0.52 | 0.58 | 0.8 | 0.41 | 1.35 | 0.66 | 0.78 | 0.42 | 0.31 | LOC100737853 |
| gene7381 | 1.072910179 | 0.003016913 | 2.21 | 2.02 | 0.86 | 0.58 | 0.69 | 1.43 | 2.47 | 1.19 | 6.15 | 0.88 | 1.52 | 1.38 | SYT6 |
| gene7404 | -1.475018269 | 1.27E-05 | 1.54 | 1.61 | 2.11 | 4.7 | 2.72 | 8.06 | 1.86 | 2.73 | 3.37 | 2.5 | 2.98 | 2.81 | WNT2B |
| gene7462 | -1.46629859 | 0.002835258 | 0.18 | 0.22 | 0.23 | 0.18 | 1.05 | 0.55 | 0.59 | 0.65 | 0.15 | 0.38 | 0.37 | 0.38 | LOC100157465 |
| gene7501 | -1.101006585 | 0.000750135 | 0.45 | 0.61 | 1.54 | 3.8 | 0.85 | 1.05 | 0.68 | 0.43 | 0.5 | 0.62 | 0.27 | 0.33 | COLL11A1 |
| gene7504 | -1.610883974 | 9.53E-06 |  |  |  |  |  |  |  |  |  |  |  |  |  |
| gene752 | 2.1113268 | 5.29E-11 | 50.28 | 29.84 | 15.41 | 5.77 | 6.05 | 12.38 | 11.57 | 14.17 | 12.52 | 15.93 | 14.77 | 29.33 | LOC100517385 |
| gene753 | 1.163289783 | 0.00016553 | 16.18 | 57.23 | 32.04 | 16.93 | 12.23 | 20.3 | 13.85 | 46.7 | 29.78 | 45.42 | 31.75 | 49.51 | LOC100153621 |
| gene7530 | -1.420717233 | 2.11E-05 | 3.02 | 1.19 | 2.11 | 4.97 | 6.62 | 6.2 | 2.39 | 0.96 | 2.72 | 1.58 | 1.3 | 1.89 | LOC100515728 |
| gene756 | 1.470217535 | 2.15E-06 | 62.78 | 77.89 | 78.08 | 17.09 | 28.73 | 37.62 | 15.72 | 33.28 | 66.06 | 79.4 | 69.8 | 128.23 | LOC100155557 |
| gene7582 | -1.050900503 | 0.001217468 | 1.56 | 2.13 | 2.55 | 5.39 | 3 | 5.21 | 4.21 | 5.17 | 3.4 | 3.09 | 4.38 | 4.06 | GBP2 |
| gene7589 | 1.177754219 | 0.00903077 | 6.9 | 6.35 | 6.17 | 4.53 | 2.38 | 2.23 | 4.55 | 4.86 | 3.16 | 4.61 | 4.47 | 3.69 | LOC100524863 |
| gene7606 | 1.149579855 | 0.000188223 | 425.33 | 136.35 | 253.24 | 159.71 | 118 | 118.49 | 311.96 | 605.46 | 131.27 | 146.97 | 670.7 | 454.19 | CYR61 |
| gene7626 | -1.490820468 | 0.006997756 | 0.1 | 0.11 | 0.4 | 0.35 | 0.08 | 1.25 | 0.32 | 0.56 | 0.2 | 0.87 | 0.53 | 0.34 | LOC100519482 |
| gene7661 | -1.196581208 | 0.001322071 | 1.87 | 1.29 | 9.64 | 10.17 | 6.34 | 12.16 | 9.9 | 5.64 | 8.19 | 17.89 | 5.35 | 3.18 | LOC100510960 |
| gene7665 | 1.134992097 | 0.00580988 | 0.08 | 5.3 | 2.67 | 3.12 | 0.19 | 0.58 | 0.13 | 2.35 | 0.14 | 0.39 | 2.67 | 1.89 | LOC100156627 |
| gene7669 | 1.146709041 | 0.003902352 | 5.79 | 4.93 | 1.55 | 1.92 | 3.46 | 0.51 | 6.07 | 8.57 | 5.32 | 0.38 | 8.2 | 8.18 | LOC100737924 |
| gene7729 | -1.157849645 | 0.000428977 | 5.13 | 6.72 | 15.67 | 16.56 | 29.45 | 16.63 | 14.3 | 14.44 | 19.75 | 6.71 | 11.83 | 4.93 | LOC100737214 |
| gene7756 | -1.445097166 | 0.000338247 | 0.93 | 1.08 | 0.78 | 2.79 | 3.14 | 2.25 | 1.51 | 2.18 | 0.8 | 0.49 | 2.18 | 0.84 | LOC100621675 |
| gene7775 | -1.261475253 | 8.87E-05 | 4.21 | 3.8 | 6.74 | 11.48 | 7.78 | 18.05 | 8.5 | 6.41 | 12.21 | 8.15 | 5.73 | 6.68 | LOC100517940 |
| gene7780 | -2.781711739 | 2.96E-12 | 0.48 | 0.14 | 0.94 | 1.09 | 7.63 | 1.35 | 1.83 | 0.2 | 0.5 | 0.36 | 0.2 | 0.09 | LOC100518305 |
| gene7788 | -1.600567429 | 1.76E-05 | 0.12 | 0.67 | 0.76 | 0.9 | 2.17 | 1.79 | 1.06 | 0.7 | 0.35 | 1.23 | 1.49 | 0.85 | BPIL2 |
| gene7817 | 2.180997874 | 5.49E-09 | 8.91 | 3.89 | 2.7 | 1.19 | 0.98 | 1.7 | 1.72 | 3.99 | 1.49 | 3.57 | 4.28 | 6.49 | LOC100737739 |
| gene7830 | 1.307778229 | 3.70E-05 |  |  |  |  |  |  |  |  |  |  |  |  |  |
| gene7832 | -1.57196843 | 5.00E-07 | 12.94 | 18.18 | 13.83 | 37.2 | 31.92 | 73.38 | 95.3 | 4 | 2.82 | 2.05 | 6.41 | 1.27 | PRPH |
| gene7887 | -1.229021883 | 9.62E-05 | 2.32 | 5.84 | 3.14 | 6.37 | 8.8 | 12.95 | 25.68 | 0.48 | 2.86 | 3.86 | 1.29 | 8.96 | LOC100737483 |
| gene7903 | -1.647740176 | 2.26E-07 | 5.14 | 2.35 | 9.43 | 12.02 | 12.1 | 31.58 | 15.94 | 0.78 | 3.39 | 4.29 | 0.45 | 16.31 | KRT4 |
| gene7966 | -1.903114846 | 6.88E-06 | 1.03 | 1 | 0.12 | 2.03 | 5.87 | 0.86 | 1.44 | 4.26 | 0.6 | 0.7 | 0.38 | 0.5 | LOC100523787 |
| gene808 | -1.665134968 | 0.002148118 | 0.56 | 0.34 | 0.43 | 0.19 | 1.79 | 2.19 | 0.49 | 0.85 | 1.54 | 0.65 | 0.5 | 1.03 | LOC100513703 |
| gene817 | 1.803374189 | 8.22E-08 | 16.31 | 5.15 | 4.52 | 4.45 | 0.49 | 3.35 | 4.36 | 19.97 | 17.36 | 25.71 | 30.52 | 14.05 | SQRDL |
| gene8215 | 1.228361534 | 0.00966605 |  |  |  |  |  |  |  |  |  |  |  |  |  |
| gene8216 | -2.96231368 | 0.004944215 | 0 | 0.04 | 0.09 | 0.24 | 0.77 | 0.19 | 0.48 | 0.42 | 0.47 | 0 | 0.11 | 0.29 | LOC100156985 |
| gene823 | -1.01413844 | 0.001835124 | 1.89 | 2.99 | 2.76 | 4.57 | 5.12 | 6.63 | 3.67 | 3.42 | 1.3 | 3.99 | 2.83 | 2.94 | GATM |
| gene8250 | 2.124184225 | 1.93E-11 | 919.05 | 599.54 | 753.16 | 187.87 | 169.67 | 199.08 | 346.33 | 363.47 | 850.44 | 1085 | 947.27 | 881.6 | CPSF6 |
| gene8264 | -1.251158448 | 0.000377742 | 1.49 | 0.97 | 1.05 | 3.28 | 3.04 | 2.5 | 6.78 | 0.92 | 1.17 | 0.69 | 1.82 | 2.09 | LOC100520307 |
| gene8292 | 1.638402266 | 1.65E-07 | 158.39 | 36.53 | 30.15 | 30.03 | 23.45 | 26.28 | 72.82 | 80.52 | 92.05 | 44.45 | 144.78 | 90.03 | LOC100523904 |
| gene832 | -1.202397996 | 0.00116323 | 0.37 | 1.13 | 0.29 | 1.23 | 1.29 | 2.31 | 0.99 | 1.06 | 0.98 | 0.36 | 1.59 | 1.27 | DUOXA1 |
| gene8323 | 1.918364041 | 1.41E-09 | 53.04 | 131.67 | 147.84 | 23.52 | 39.98 | 27.58 | 16.1 | 44.1 | 24.27 | 39.23 | 31.93 | 61.2 | PTHLH |
| gene8325 | -1.004760789 | 0.003214027 | 2.2 | 1.78 | 1.93 | 5.54 | 3.93 | 3.02 | 1.93 | 2.28 | 3.44 | 3.03 | 1.85 | 1.3 | LOC100516012 |
| gene8346 | -2.274968672 | 2.05E-08 | 0.27 | 0.3 | 0.6 | 3.04 | 1.29 | 1.35 | 0.74 | 2.51 | 0.84 | 1.92 | 2.05 | 1.04 | LOC100627442 |
| gene8368 | -1.691700166 | 1.04E-05 | 0.46 | 0.13 | 0.83 | 1.25 | 2.47 | 0.61 | 1.77 | 1.53 | 1.47 | 1.01 | 0.69 | 0.81 | SLCO1B3 |
| gene8381 | -2.192470815 | 7.76E-06 | 0.3 | 0.41 | 0.16 | 1.13 | 2.42 | 0.92 | 0.94 | 0.38 | 0.21 | 0.34 | 0.98 | 3.46 | LOC100153016 |
| gene8393 | 1.46065876 | 2.66E-06 | 209.17 | 152.74 | 211.17 | 69.55 | 78.51 | 72.73 | 41.77 | 147.43 | 153.38 | 184.25 | 176.09 | 210.1 | RERG |
| gene84 | -2.048628079 | 0.003790376 | 0.07 | 0.12 | 0.27 | 0.94 | 0.31 | 0.67 | 0.21 | 0.35 | 1.05 | 0.25 | 0.92 | 0.95 | LOC100526086 |
| gene8405 | -1.1827189 | 0.002850159 | 0.63 | 0.53 | 0.31 | 1.41 | 1.27 | 0.96 | 0.35 | 0.45 | 0.64 | 0.43 | 0.89 | 0.48 | LOC100626467 |
| gene8410 | 1.346015034 | 8.16E-05 | 0.78 | 0.75 | 1.8 | 0.49 | 0.32 | 0.56 | 0.07 | 0.12 | 0.07 | 0.1 | 0.15 | 0.06 | GRIN2B |
| gene8463 | -1.102909437 | 0.000890616 | 4.72 | 3.22 | 5.57 | 13.5 | 5.97 | 11.27 | 6 | 7.71 | 2.56 | 11.55 | 4.91 | 3.89 | LOC100520491 |
| gene8468 | -3.304526837 | 2.42E-08 | 0.02 | 0.02 | 0.02 | 0.21 | 0.05 | 0.37 | 0.05 | 0.06 | 0.01 | 0.97 | 0.01 | 0.04 | PZP |
| gene8522 | -1.877068255 | 7.01E-05 | 0.41 | 0.25 | 0.37 | 1.51 | 1.38 | 1 | 1.26 | 1.61 | 1.37 | 1.2 | 0.94 | 1.12 | LOC100520023 |
| gene8596 | -1.674455048 | 1.05E-06 | 1.58 | 1.68 | 1.72 | 9.59 | 2.78 | 4.48 | 3.23 | 3.72 | 3.56 | 4.67 | 3.46 | 2.95 | USP18 |
| gene8722 | -1.139504077 | 0.000380512 | 2.7 | 2.39 | 2.47 | 2.7 | 4.82 | 10.34 | 8.92 | 1.51 | 0.57 | 1.12 | 1.3 | 0.21 | SLC5A8 |
| gene8727 | -1.469347998 | 5.94E-06 | 5.46 | 1.78 | 5.39 | 23.79 | 8.72 | 4.12 | 12.31 | 18.27 | 37.47 | 9.1 | 24.09 | 22.25 | SLC17A8 |
| gene8803 | -1.391038811 | 0.003496302 | 0.18 | 0.08 | 0.05 | 0.2 | 0.07 | 0.67 | 0.05 | 0.03 | 0.03 | 0.15 | 0.08 | 0.11 | LOC100526053 |
| gene8806 | -1.572110734 | 2.63E-06 | 0.77 | 0.41 | 0.67 | 1.06 | 0.59 | 4.25 | 0.3 | 0.63 | 0.25 | 0.47 | 0.48 | 0.5 | LRRIQ1 |
| gene8816 | -2.11215836 | 0.009254525 | 0.03 | 0.03 | 0.03 | 0.18 | 0.06 | 0.22 | 0.26 | 0.13 | 0.09 | 0.08 | 0.18 | 0.14 | LOC100621252 |
| gene8829 | -1.342512984 | 6.65E-05 | 1.37 | 1.65 | 2.62 | 7.43 | 3.69 | 3.58 | 1.78 | 6.83 | 2 | 3.67 | 1.79 | 1.11 | SYT1 |
| gene8834 | -1.046715469 | 0.002338908 | 0.45 | 0.76 | 0.53 | 0.31 | 2.55 | 0.88 | 1.01 | 0.29 | 0.85 | 0.41 | 0.53 | 0.45 | E2F7 |
| gene8835 | 1.093105537 | 0.005788135 |  |  |  |  |  |  |  |  |  |  |  |  |  |
| gene8859 | -1.440438675 | 0.000744914 | 0.14 | 0.17 | 0.13 | 0.21 | 0.69 | 0.37 | 1.26 | 0.07 | 0.14 | 0.22 | 0.17 | 0.08 | LOC100520675 |
| gene8860 | -2.259060577 | 0.001668335 | 0.03 | 0.06 | 0 | 0.12 | 0.44 | 0.04 | 0.12 | 0.03 | 0.05 | 0.13 | 0.04 | 0 | LOC100520842 |
| gene8931 | -1.633333585 | 3.30E-07 | 2.43 | 2.11 | 2.38 | 4.46 | 12.17 | 5.87 | 10.62 | 1.44 | 1.8 | 1.74 | 1.38 | 0.55 | ATP2C2 |
| gene8959 | 2.941808907 | 0.000483434 | 0 | 3.64 | 4.16 | 0.41 | 0.4 | 0 | 0 | 1.83 | 1.81 | 2.26 | 1.11 | 3.98 | LOC100738925 |
| gene8994 | -1.438002263 | 0.000138965 | 0.33 | 0.6 | 1.11 | 1.37 | 1.32 | 2.9 | 1.33 | 0.79 | 0.44 | 0.5 | 0.44 | 0.4 | CLEC18A |
| gene9002 | -1.40585204 | 0.000471457 | 1.11 | 0.95 | 1.44 | 4.16 | 3.91 | 1.39 | 3.24 | 1.01 | 2.44 | 0.84 | 1.4 | 1.37 | LOC100737468 |
| gene9025 | -1.188472512 | 0.00011237 | 999.68 | 1737.9 | 2063 | 2301.5 | 4479.4 | 4640.7 | 7479.1 | 36.14 | 213.1 | 389.77 | 970.58 | 256.38 | HP |
| gene9072 | -1.26215193 | 0.001963847 | 0.45 | 0.52 | 0.09 | 1.26 | 0.61 | 1.04 | 0.9 | 0.31 | 0.35 | 0.91 | 0.39 | 0.52 | LOC100627282 |
| gene9152 | 1.178740377 | 0.00013565 | 637.82 | 842.29 | 691.92 | 327.11 | 310.21 | 378.81 | 229.43 | 440.22 | 355.41 | 540.76 | 400.5 | 623.36 | LOC100519152 |
| gene9169 | 1.430661237 | 0.000305919 | 1.01 | 0.94 | 1.02 | 0.39 | 0.33 | 0.51 | 0.28 | 2.09 | 0.78 | 0.54 | 2.27 | 0.74 | LOC100626670 |
| gene9199 | -1.14284354 | 0.00055477 | 1.23 | 1.07 | 1.35 | 3.74 | 1.59 | 3.24 | 1.99 | 1.25 | 1.06 | 1.57 | 0.7 | 0.77 | LRP3 |
| gene9221 | -1.328266301 | 0.000233208 | 5.28 | 2.99 | 4.29 | 7.13 | 10.66 | 15.17 | 8.76 | 12.67 | 4.99 | 29.39 | 12.22 | 12.65 | LOC100623939 |
| gene9223 | -1.185999993 | 0.001146147 | 0.6 | 0.6 | 1.09 | 1.21 | 2.15 | 1.91 | 0.73 | 1.89 | 0.76 | 1.47 | 1.14 | 1.22 | LGI4 |
| gene9302 | -1.292537274 | 0.006684863 | 0.41 | 0.52 | 0.07 | 1.39 | 0.72 | 0.73 | 0.91 | 0.22 | 0.18 | 1.01 | 1.06 | 0.44 | LOC100620402 |
| gene941 | -1.525785962 | 0.002192047 | 0.42 | 0.26 | 0.06 | 0.88 | 0.38 | 1.3 | 0.19 | 1.11 | 0.32 | 0.45 | 0.48 | 0.13 | LOC100627103 |
| gene9434 | 1.088539976 | 0.000468146 | 252.5 | 58 | 91.68 | 73.92 | 78.11 | 53.43 | 52.89 | 86.76 | 100.3 | 206.15 | 90.3 | 130.72 | LOC100623158 |
| gene9470 | 4.99552455 | 0.000931586 |  |  |  |  |  |  |  |  |  |  |  |  |  |
| gene9491 | -1.706601228 | 4.63E-08 | 134.68 | 115.41 | 217.35 | 632.85 | 271.12 | 712.4 | 211.35 | 106.3 | 190.31 | 85.69 | 73.69 | 223.78 | APOE |
| gene9562 | -1.440558781 | 0.006684863 | 0.37 | 0.15 | 0.27 | 0.52 | 1.08 | 0.65 | 0.9 | 0.04 | 0.07 | 0.13 | 0.19 | 0.16 | LOC100521627 |
| gene9633 | 1.98087253 | 5.51E-10 | 7.56 | 72.85 | 44.12 | 11.82 | 13.87 | 6.8 | 8.56 | 11.66 | 9.05 | 15.32 | 9.01 | 6.34 | LOC100523675 |
| gene9655 | -1.376089108 | 0.003496302 | 0 | 0.19 | 0.27 | 0.36 | 0.3 | 0.55 | 0.1 | 0.32 | 0.28 | 0.3 | 0.1 | 0.26 | LOC100738681 |
| gene975 | 1.515095087 | 2.27E-06 | 12.73 | 47.55 | 13.33 | 5.94 | 8.12 | 13.34 | 7.42 | 13.48 | 9.07 | 48.77 | 33.25 | 22.8 |  |
| gene981 | -1.513213114 | 0.001477359 | 0.27 | 0.38 | 0.24 | 0.96 | 1.52 | 0.26 | 0.29 | 0.38 | 0.24 | 0.67 | 0.22 | 0.39 | LOC100518709 |
| gene9852 | -1.10323455 | 0.007044681 | 1.94 | 1.44 | 1.28 | 3.26 | 3.73 | 4.02 | 5.94 | 14.14 | 5.81 | 8.88 | 3.55 | 4.75 | LOC100522375 |
| gene992 | 1.593236566 | 0.001507798 | 0.39 | 0.54 | 1.06 | 0.23 | 0.15 | 0.28 | 0.24 | 0.75 | 0.57 | 0.43 | 0.85 | 0.93 | LASS3 |
| gene9971 | 1.771404674 | 1.81E-08 | 140.4 | 59.18 | 94.69 | 23.68 | 40.91 | 27.49 | 69.75 | 77.62 | 91.74 | 75.67 | 146.96 | 199.14 | LOC100519197 |
| gene9975 | 1.58065007 | 3.84E-07 | 286.83 | 132.53 | 180.71 | 72.68 | 65.84 | 77.38 | 158.42 | 196.59 | 196.14 | 191.9 | 285.49 | 342.82 | LOC100738544 |
| gene9992 | -1.549737823 | 8.55E-07 | 54.92 | 12.14 | 40.64 | 64.42 | 192.11 | 76.72 | 42.22 | 5.39 | 18.36 | 17.2 | 20.59 | 13.77 | RBP7 |

Supplementary Table S2: Differentially expressed genes (DEGs) in QL

| Gene | logFC | PValue | LL1 | LL2 | LL3 | LN1 | LN2 | LN3 | QS1 | QS2 | QS3 | QL1 | QL2 | QL3 | Name |
| --- | --- | --- | --- | --- | --- | --- | --- | --- | --- | --- | --- | --- | --- | --- | --- |
| gene10024 | -1.046751503 | 0.001827381 | 6.13 | 6.3 | 7.86 | 9.85 | 9.86 | 11.45 | 8.7 | 6.56 | 4.29 | 5.17 | 4.77 | 4.67 | MIIP |
| gene1004 | -1.042959764 | 0.003468362 | 12.61 | 15.69 | 15.72 | 10.19 | 15.09 | 12.41 | 17.54 | 5.06 | 12.65 | 6.86 | 4.37 | 6.69 | LOC100739145 |
| gene10072 | 1.740214545 | 1.33E-07 | 14.25 | 6.74 | 7.45 | 4.11 | 4.62 | 5.54 | 6.71 | 9.7 | 5.77 | 10.19 | 20.41 | 14.78 | LOC100522873 |
| gene10086 | -2.228098967 | 5.08E-07 | 0.46 | 0.42 | 0.99 | 0.58 | 2.19 | 0.25 | 0.8 | 0.57 | 1 | 0.06 | 0.12 | 0.44 | PADI1 |
| gene10120 | 2.333425596 | 2.49E-05 | 0.23 | 0.14 | 0.41 | 0.09 | 0.04 | 0.5 | 0.34 | 1.93 | 0.81 | 1.69 | 0.97 | 0.33 | LOC100624917 |
| gene10121 | 1.551914792 | 2.01E-06 | 101.64 | 64.59 | 55.3 | 51.22 | 52.85 | 39.63 | 59.22 | 159.12 | 110.23 | 73.67 | 218.33 | 110.44 | CDA |
| gene10150 | -1.002767668 | 0.00524731 | 1.6 | 1.78 | 2.09 | 6.4 | 2 | 3.43 | 2.88 | 3.54 | 2.87 | 2.66 | 2.21 | 0.86 | LOC100739392 |
| gene10165 | 1.838490469 | 0.001229974 | 0.17 | 0.24 | 0.15 | 0.26 | 0.45 | 0.08 | 1.03 | 0.82 | 0.29 | 1.7 | 0.97 | 0.4 | LOC100621946 |
| gene10185 | 4.82215628 | 0.003168798 | 0.08 | 0.04 | 0.19 | 0 | 0 | 0 | 0 | 0 | 0 | 0.19 | 0.12 | 0.21 | PAQR7 |
| gene10257 | -2.907352352 | 3.08E-09 | 0.14 | 0.49 | 0.37 | 0.59 | 1.75 | 1.79 | 0.59 | 0.23 | 0.05 | 0.13 | 0.13 | 0.32 | MATN1 |
| gene10270 | 2.126211164 | 9.24E-05 | 0.16 | 2.42 | 0.22 | 0.25 | 0.09 | 0.12 | 0.15 | 0.32 | 0.38 | 0.18 | 0.33 | 1.38 | LOC100520618 |
| gene10274 | -2.389223679 | 3.49E-07 | 0.4 | 0.54 | 0.96 | 0.57 | 1.27 | 1.82 | 0.32 | 0.17 | 0 | 0.13 | 0.08 | 0.46 | LOC100737666 |
| gene10302 | -1.879197253 | 0.000475525 | 0.37 | 0.42 | 0.49 | 0.46 | 0.66 | 0.68 | 0.24 | 0.42 | 0.48 | 0 | 0.12 | 0.32 | LCK |
| gene10321 | 1.037076637 | 0.003720128 | 5.32 | 6.1 | 4.72 | 2.85 | 2.85 | 3.63 | 2.52 | 4.5 | 4.89 | 6.42 | 5.08 | 6.86 | LOC100739829 |
| gene10380 | -1.290709813 | 7.03E-05 | 22.42 | 15.41 | 26.3 | 15.59 | 71.04 | 20.06 | 27.03 | 6.52 | 13.67 | 12.2 | 11.98 | 18.79 | MFSD2A |
| gene10387 | 1.062137253 | 0.000987007 | 24.76 | 26.5 | 26.28 | 28.62 | 27.56 | 28.17 | 23.05 | 36.33 | 59.35 | 47.62 | 50.21 | 71.89 | CAP1 |
| gene10392 | 1.03253714 | 0.003062552 | 4.04 | 2.57 | 3.46 | 4.6 | 2.28 | 2.04 | 2.4 | 6.66 | 6.25 | 5.6 | 6.83 | 5.21 | LOC100739231 |
| gene10396 | -1.273583954 | 0.00044066 | 2.5 | 2.81 | 3.69 | 5.71 | 4.68 | 4.78 | 1.17 | 4.57 | 2.8 | 2.16 | 1.78 | 2.26 | LOC100622189 |
| gene10397 | -1.174529306 | 0.002012744 | 5.28 | 6.27 | 4.86 | 8.4 | 6.69 | 7.66 | 8.87 | 6.62 | 3.21 | 4.73 | 3.09 | 2.16 | LOC100739356 |
| gene10410 | 1.428313096 | 2.15E-05 | 15.27 | 8.98 | 8.99 | 6.4 | 10.56 | 7 | 11.26 | 18.72 | 16.3 | 8.64 | 32.15 | 20.78 | CIDEA |
| gene10413 | 2.399470081 | 2.72E-06 | 1.47 | 2.83 | 1.64 | 0.21 | 0.54 | 0.34 | 0.19 | 1.41 | 0.62 | 2.58 | 1.94 | 1.26 | LOC100623971 |
| gene10431 | 2.212057466 | 0.000643336 |  |  |  |  |  |  |  |  |  |  |  |  |  |
| gene1044 | -2.064212954 | 5.94E-10 | 31.99 | 15.79 | 34.61 | 28.99 | 55.49 | 27.83 | 13.02 | 6.29 | 11.88 | 10.41 | 6.42 | 9.59 | LOC100516053 |
| gene10472 | 1.034256308 | 0.001386811 | 5.62 | 7.24 | 8.72 | 4.53 | 3.93 | 3.43 | 11.17 | 6.6 | 7.27 | 8.69 | 6.92 | 8.03 | LOC100739192 |
| gene10473 | 1.216930756 | 0.000151109 | 81.94 | 102.56 | 102.46 | 111.89 | 73.73 | 59.44 | 94.89 | 113.28 | 141.8 | 116.36 | 202.79 | 227.28 | LAMA3 |
| gene10498 | -1.728688139 | 7.68E-05 | 0.04 | 0.18 | 0.17 | 0.86 | 0.3 | 0.88 | 0.49 | 0.17 | 0.17 | 0.47 | 0 | 0.14 | LOC100515669 |
| gene10499 | -1.297972783 | 0.000205239 | 0.18 | 0.58 | 0.91 | 1.95 | 2.24 | 2.56 | 0.98 | 0.42 | 1.29 | 0.91 | 0.8 | 0.93 | DSG1 |
| gene10504 | 1.820539214 | 0.008464725 | 0.08 | 0.1 | 0 | 0.1 | 0.42 | 0.39 | 0.05 | 0 | 0 | 3.07 | 0 | 0 | TTR |
| gene10511 | -1.832447817 | 5.14E-06 |  |  |  |  |  |  |  |  |  |  |  |  |  |
| gene10512 | -1.941800028 | 3.71E-09 | 17.54 | 17.31 | 29.49 | 24.69 | 31.72 | 141.43 | 88.83 | 21.06 | 15.34 | 17.7 | 23.26 | 7.6 | MEP1B |
| gene10517 | -1.206813728 | 0.002704294 | 0.15 | 0.17 | 0.28 | 0.39 | 0.3 | 0.3 | 0.12 | 0.11 | 0.1 | 0.19 | 0.12 | 0.13 | ASXL3 |
| gene10536 | -2.67775592 | 1.02E-08 | 0.76 | 0.89 | 0.3 | 0.87 | 0.5 | 0.89 | 0.29 | 0.22 | 0.2 | 0.26 | 0 | 0.1 | LOC100621611 |
| gene10543 | -1.140794594 | 0.001567653 | 0.92 | 1.98 | 1.61 | 3.79 | 3.07 | 2.37 | 1.13 | 3.67 | 1.07 | 1.14 | 0.98 | 1.97 | CELF4 |
| gene10552 | -1.263068253 | 0.000350727 | 5.01 | 3.25 | 3.09 | 4.45 | 6.29 | 6.36 | 4.03 | 5.48 | 2.78 | 2.55 | 2.69 | 1.73 | LPAR3 |
| gene10568 | -1.337249131 | 5.15E-05 | 6.72 | 6.75 | 5.38 | 35.46 | 6.4 | 14.2 | 6.69 | 11.56 | 7.56 | 11.68 | 4.67 | 5.39 | IFI44 |
| gene10577 | 1.006323903 | 0.001858695 | 28.23 | 26.14 | 29.3 | 49.91 | 26.33 | 59.6 | 29.3 | 100.85 | 43.16 | 70.14 | 95.95 | 93.28 | LOC100737098 |
| gene10616 | -1.299963867 | 8.42E-05 | 13.81 | 16.61 | 15.12 | 34.84 | 30.9 | 29.16 | 8.48 | 15.59 | 10.1 | 17.5 | 10.15 | 10.16 | DIRAS3 |
| gene10647 | -1.09121223 | 0.001962522 | 1.05 | 0.73 | 1.79 | 1.6 | 2.27 | 1.36 | 1.08 | 1.03 | 1.04 | 1.29 | 0.53 | 0.62 | KANK4 |
| gene10655 | -1.072105326 | 0.001401696 | 5.51 | 2.72 | 5.82 | 6.83 | 10.69 | 6.1 | 7.8 | 4.42 | 5.42 | 3.47 | 4.32 | 3.2 | CYP2J34 |
| gene10677 | 1.279669783 | 0.001732598 | 2.22 | 2.61 | 2.41 | 1.61 | 1.25 | 0.8 | 3.42 | 2.68 | 2.04 | 2.32 | 2.51 | 3.86 | TMEM61 |
| gene10689 | -3.650097775 | 3.95E-15 | 2.19 | 1.79 | 0.59 | 2.6 | 1.26 | 1.18 | 0.21 | 0.07 | 0.19 | 0.23 | 0.06 | 0.1 | LOC100622589 |
| gene10696 | -2.309518366 | 0.00264108 | 0.24 | 0.26 | 0.05 | 0.44 | 0.38 | 0.42 | 0.46 | 0.18 | 0.28 | 0.06 | 0.06 | 0.1 | LOC100515733 |
| gene10702 | -1.033787142 | 0.009457992 | 0.4 | 0.89 | 0.54 | 1.87 | 0.65 | 1.25 | 1.37 | 0.84 | 0.2 | 0.44 | 0.64 | 0.65 | SLC1A7 |
| gene10705 | -1.042333047 | 0.001449442 | 14.04 | 14 | 21.67 | 23.87 | 37.56 | 27.03 | 22.89 | 12.96 | 15.04 | 17.06 | 13.23 | 11.75 | LOC100519847 |
| gene10739 | -2.916102472 | 0.001815837 | 0.21 | 0.87 | 0.77 | 0.72 | 0.59 | 1.06 | 0.4 | 0.47 | 0.82 | 0.02 | 0.32 | 0.1 | LOC100626175 |
| gene1075 | -1.630561565 | 4.80E-06 | 1.96 | 1.88 | 1.81 | 3.72 | 12.44 | 2.48 | 6.09 | 0.49 | 0.87 | 2.32 | 2.5 | 1.28 | SERPINB7 |
| gene10753 | -1.120028628 | 0.000872037 | 4.3 | 6.55 | 2.76 | 10.91 | 6.15 | 5.99 | 1.72 | 2.81 | 1.82 | 5.15 | 3.13 | 2.12 | LOC100523909 |
| gene1076 | -1.990461166 | 3.46E-09 | 8.14 | 9.74 | 11.71 | 15.59 | 32.41 | 31.99 | 33.84 | 2.03 | 5.43 | 6.58 | 6.64 | 6.31 | LOC100153783 |
| gene10766 | -1.685209353 | 0.001084532 | 0.68 | 0.26 | 0.76 | 1.55 | 1.31 | 1.06 | 1.12 | 0.99 | 0.18 | 0.17 | 0.58 | 0.52 | LOC100525230 |
| gene10791 | 1.202939383 | 0.000253118 | 17.42 | 7.42 | 6.44 | 3.99 | 4.16 | 8.3 | 7.83 | 8.3 | 8.62 | 6.06 | 14.09 | 15.5 | PLK3 |
| gene10792 | -2.798179176 | 0.000481506 | 0.4 | 0.49 | 0.61 | 0.81 | 0.33 | 1.1 | 0.83 | 0.84 | 0.2 | 0.19 | 0.09 | 0 | TCTEX1D4 |
| gene10796 | -1.506263578 | 0.000159531 | 4.44 | 1.98 | 4.28 | 3.63 | 7.68 | 4.62 | 2.3 | 2.09 | 1.25 | 2.08 | 2.64 | 0.84 | LOC100739546 |
| gene10810 | -1.788701252 | 0.001326536 | 1.17 | 3.39 | 5.81 | 2.74 | 2.35 | 3.35 | 2.46 | 0 | 1.02 | 1.9 | 0 | 0.42 | LOC100736914 |
| gene10835 | -1.761302913 | 1.31E-05 |  |  |  |  |  |  |  |  |  |  |  |  |  |
| gene10856 | -1.182524988 | 0.004302869 | 0.52 | 0.39 | 0.53 | 0.53 | 2.06 | 1.09 | 0.36 | 0.43 | 0.68 | 0.51 | 0.42 | 0.69 | LOC100526058 |
| gene10871 | -1.946959753 | 5.88E-07 | 1.31 | 2.55 | 1.74 | 3.88 | 3.42 | 2.06 | 4.52 | 0.59 | 2.03 | 0.45 | 0.72 | 1.22 | LOC100152843 |
| gene10873 | -1.020992205 | 0.001509669 | 52.66 | 58.99 | 57.25 | 66.35 | 79.7 | 88.25 | 85.58 | 41.9 | 38.77 | 38.5 | 33.47 | 39.64 | GMDS |
| gene10893 | -3.393566229 | 1.75E-07 | 0.25 | 0.66 | 0.33 | 1.05 | 0.7 | 0.91 | 0.55 | 0.07 | 0.63 | 0.17 | 0.11 | 0.05 | LOC100522260 |
| gene1091 | -1.316243453 | 0.004012828 | 0.1 | 0.13 | 0.29 | 0.34 | 0.51 | 0.37 | 0.17 | 0.41 | 0.25 | 0.19 | 0.12 | 0.15 | CDH20 |
| gene10911 | -1.576278428 | 4.03E-05 | 3.17 | 3.49 | 3.44 | 4.14 | 4.06 | 3.93 | 2.26 | 0.98 | 0.47 | 0.87 | 2.25 | 0.77 | LOC100627410 |
| gene10915 | 3.278718066 | 1.57E-14 | 0.09 | 0.1 | 0.1 | 0.08 | 0.26 | 0.12 | 0.59 | 2.37 | 0.88 | 1.54 | 2.19 | 0.84 | LOC100627687 |
| gene10923 | 1.143169201 | 0.006266658 | 0.33 | 0.39 | 0.22 | 0.25 | 0.27 | 0.81 | 0.69 | 0.94 | 0.27 | 0.54 | 0.93 | 1.19 | MAK |
| gene10925 | -1.654044022 | 3.87E-07 | 47.5 | 23.12 | 61.3 | 82.03 | 162.5 | 85.4 | 82.37 | 26.85 | 56.67 | 33.74 | 35.63 | 32.96 | LOC100153368 |
| gene10929 | -1.832081459 | 5.81E-05 | 0.98 | 1.3 | 1.74 | 1.11 | 1.95 | 2.67 | 0.72 | 2.14 | 0.73 | 0.63 | 0.3 | 0.61 | LOC100621321 |
| gene10937 | 2.932193552 | 0.003292388 | 0.19 | 0.12 | 0.36 | 0.11 | 0 | 0.2 | 0.3 | 0.49 | 0.49 | 0.44 | 1.62 | 0.28 | LOC100621914 |
| gene10942 | -1.369625051 | 0.000573555 | 4.14 | 5.99 | 3.84 | 5.33 | 8.57 | 5.97 | 3.49 | 1.73 | 2.53 | 3.7 | 0.81 | 2.96 | SIRT5 |
| gene10951 | -1.976889008 | 1.82E-09 | 104.76 | 52.68 | 95.62 | 147.11 | 330.39 | 170.8 | 177.96 | 76.55 | 85.64 | 44.38 | 65.84 | 49.76 | MYLIP |
| gene10957 | -2.99364939 | 1.58E-08 | 1.57 | 0.97 | 1.31 | 2.09 | 14.19 | 7.06 | 1.26 | 0.83 | 0.35 | 0 | 1.87 | 0.85 | LOC100522879 |
| gene10969 | -1.189810478 | 0.000233689 | 113.51 | 89.4 | 89.99 | 66.59 | 142.45 | 82.8 | 66.95 | 36.9 | 37.4 | 45.67 | 43.67 | 35.71 | TPMT |
| gene10978 | 1.191051983 | 0.000210292 | 219.85 | 348.38 | 209.75 | 104.84 | 21.17 | 191.31 | 28.68 | 338.72 | 179.7 | 199.1 | 214.69 | 268.43 | LOC100512542 |
| gene10995 | 1.413954995 | 0.000104592 | 1.02 | 0.72 | 1.62 | 0.44 | 0.49 | 0.72 | 0.45 | 0.88 | 0.62 | 2.05 | 0.85 | 1.31 | LOC100154661 |
| gene110 | 1.29062491 | 0.000131253 | 6.82 | 6.89 | 10.62 | 5.06 | 3.98 | 11.07 | 2.27 | 9.03 | 9.06 | 17.47 | 11.48 | 17.98 | LOC100737874 |
| gene1104 | 1.0560514 | 0.001134183 | 3.64 | 3.75 | 5.01 | 5.44 | 5.7 | 4.73 | 6.91 | 7.38 | 12.46 | 5.08 | 10.91 | 15.56 | MALT1 |
| gene11058 | 1.314435691 | 0.003354152 | 2.74 | 1.77 | 1.49 | 1.31 | 1.24 | 1.97 | 2.8 | 9.31 | 1.72 | 1.44 | 8.41 | 0.69 | LOC100621915 |
| gene11069 | 3.732970602 | 2.46E-05 | 0 | 0 | 0 | 0 | 0 | 0.12 | 0.06 | 0.89 | 0.49 | 0.98 | 0.25 | 0.35 | LOC100514657 |
| gene11113 | -3.504935313 | 0.000481426 | 0 | 0.05 | 0.11 | 0.66 | 0.1 | 0.31 | 0 | 0.05 | 0.15 | 0.07 | 0 | 0 | GPX5 |
| gene11124 | 1.423638645 | 1.17E-05 | 141.95 | 34.85 | 35.28 | 68.99 | 44.31 | 74.73 | 55.75 | 110.74 | 25.15 | 306.76 | 127.99 | 60.26 |  |
| gene11127 | -2.161331854 | 6.69E-11 | 64.78 | 126.71 | 222.93 | 269.92 | 212.56 | 152.33 | 39.12 | 59.46 | 16.32 | 75.7 | 35.79 | 28.5 | LOC100513601 |
| gene1113 | -1.368779269 | 0.000485769 | 0.3 | 0.13 | 0.23 | 0.99 | 0.5 | 0.66 | 0.19 | 0.19 | 0.39 | 0.14 | 0.2 | 0.44 | CILP |
| gene11130 | -1.110545112 | 0.00054743 | 74.5 | 94.14 | 111.49 | 165.89 | 99.83 | 211.47 | 78.14 | 92.09 | 29.47 | 103.78 | 60.95 | 49.18 | SLA-1 |
| gene11138 | 2.233613812 | 6.20E-11 | 11.01 | 3.99 | 5.06 | 4.65 | 5.72 | 3.67 | 2.91 | 13.56 | 13.9 | 8.58 | 21.12 | 33.32 | RNF39 |
| gene11140 | 1.302999782 | 0.002167406 | 2.04 | 0.19 | 0.41 | 0.36 | 3.99 | 0.59 | 1.56 | 4.52 | 8.37 | 0.66 | 5.27 | 6.27 | LOC100155727 |
| gene11144 | 1.300213682 | 6.04E-05 | 27.1 | 17.35 | 10.36 | 10.95 | 9.57 | 13.64 | 40 | 24.46 | 36.72 | 9.63 | 34.45 | 35.37 |  |
| gene11157 | 1.737317713 | 4.16E-07 | 4.85 | 6.59 | 5.96 | 9.88 | 5.87 | 5.8 | 4.55 | 19.02 | 7.97 | 21.11 | 21.66 | 26.2 | UBD |
| gene1116 | -1.089770241 | 0.003157895 | 0.54 | 0.52 | 0.76 | 0.84 | 0.88 | 1.19 | 0.54 | 0.26 | 0.46 | 0.6 | 0.27 | 0.46 | LOC100513900 |
| gene11237 | -1.266041086 | 0.000111368 |  |  |  |  |  |  |  |  |  |  |  |  |  |
| gene11255 | 1.792013378 | 4.19E-08 | 162.88 | 571.14 | 355.48 | 176.26 | 78.32 | 110.85 | 160.69 | 375.75 | 385.05 | 297.79 | 465.03 | 445.5 | LOC100154770 |
| gene11265 | -1.673505051 | 3.63E-05 | 0.89 | 2.12 | 0.66 | 1.18 | 1.59 | 4.56 | 1.54 | 0.32 | 2.34 | 0.71 | 0.52 | 0.91 | TCF19 |
| gene11273 | 1.116757962 | 0.001685879 | 7.06 | 2.9 | 1.64 | 6.53 | 4.1 | 4.71 | 3.11 | 8.18 | 5.71 | 16.07 | 8.15 | 8.11 | LOC100521803 |
| gene11296 | -1.113818747 | 0.000658664 | 3.84 | 2.72 | 2.94 | 6.53 | 2.28 | 7.91 | 3.55 | 2 | 1.15 | 3.84 | 1.58 | 2.06 | C4 |
| gene11335 | 3.205605023 | 0.000179505 | 0.19 | 0.06 | 0.7 | 0 | 0.11 | 0 | 0 | 0.4 | 0.08 | 0.91 | 0.53 | 0 | LOC100519552 |
| gene11338 | -1.906521844 | 1.66E-08 | 5.91 | 2.54 | 3.56 | 41.08 | 40.14 | 7.59 | 10.76 | 20.76 | 4.29 | 7.77 | 5.52 | 10.07 | SLA-DRB1 |
| gene11343 | 5.102927563 | 0.000978692 | 0.08 | 0.03 | 0.05 | 0 | 0 | 0 | 0.1 | 0.22 | 0.07 | 0.07 | 0.26 | 0.35 |  |
| gene11377 | 1.696121151 | 0.006540354 | 0.06 | 0.21 | 0.16 | 0.1 | 0.1 | 0.25 | 0.19 | 0.62 | 0.2 | 0.52 | 0.23 | 0.67 | MLIP |
| gene11386 | 1.492665354 | 6.55E-06 | 63.15 | 23.34 | 48.95 | 14.9 | 14.17 | 11.7 | 71.62 | 31.5 | 32.61 | 35.67 | 42.77 | 32.42 | LOC100522554 |
| gene11417 | 1.030580925 | 0.001284664 | 1371.1 | 1213.6 | 692.49 | 532.34 | 733 | 617.85 | 893.88 | 981.8 | 2113.6 | 546.15 | 1536.3 | 1589.8 | HMGA1 |
| gene11424 | -1.892297591 | 9.85E-05 | 0.4 | 0.23 | 0.54 | 0.48 | 1.62 | 2.12 | 1.52 | 0.24 | 0.07 | 0.31 | 0.75 | 0.05 | SPDEF |
| gene11425 | -1.653251748 | 3.44E-05 | 1.5 | 0.88 | 1.18 | 1.95 | 1.34 | 3.19 | 3.98 | 0.65 | 0.1 | 0.97 | 0.72 | 0.34 | LOC100157037 |
| gene1144 | -1.427079419 | 0.002086585 | 0.51 | 0.4 | 0.13 | 0.74 | 0.38 | 0.32 | 0.18 | 0.13 | 0.31 | 0.22 | 0.24 | 0.08 | SKOR1 |
| gene11469 | -1.05031049 | 0.001491926 | 4.74 | 3.96 | 11.11 | 13.2 | 12.94 | 9.49 | 6.6 | 1.98 | 3.77 | 4.73 | 4.66 | 7.32 | PI16 |
| gene11496 | 1.287449611 | 0.000231697 | 3.87 | 7.54 | 5.64 | 1.57 | 0.92 | 3.01 | 1.27 | 1.6 | 2.32 | 3.27 | 3.09 | 6.32 |  |
| gene11497 | 1.220198173 | 0.00044638 | 9.12 | 12.26 | 17.48 | 3.64 | 3.88 | 5.91 | 5.58 | 3.8 | 4.08 | 10.27 | 9.1 | 10.63 | LOC100155551 |
| gene11503 | -1.218114178 | 0.004128876 | 0.3 | 1.64 | 0.95 | 1.96 | 0.76 | 5.41 | 1 | 1.94 | 1.15 | 1.16 | 0.85 | 1.19 | LOC100737974 |
| gene11505 | 4.672554734 | 0.005776466 | 0.04 | 0.09 | 0.09 | 0 | 0 | 0 | 0.09 | 0.08 | 0 | 0.12 | 0.42 | 0 | LOC100738047 |
| gene11506 | 1.849738463 | 0.002149704 | 0.6 | 0.35 | 0.35 | 0.25 | 0.06 | 0.47 | 0.42 | 0.89 | 0.18 | 0.87 | 1 | 0.8 | TREM1 |
| gene11526 | -1.720729544 | 0.001567695 | 0.52 | 0.08 | 0.43 | 0.92 | 1.66 | 1.61 | 0.42 | 0.25 | 1.07 | 0.53 | 0.1 | 0.66 | LOC100739200 |
| gene11570 | -1.22066575 | 0.001360245 | 0.92 | 1.78 | 1.16 | 2.2 | 1.81 | 1.18 | 1.36 | 0.77 | 1.38 | 0.81 | 0.5 | 0.87 | LOC100514413 |
| gene11582 | 1.056837772 | 0.000955391 | 1340.1 | 1450 | 1382.8 | 911.81 | 859.79 | 802.66 | 852.7 | 1651.7 | 1733.4 | 1729 | 1920.4 | 1525.2 | HSPCB |
| gene11592 | 1.3501731 | 0.000361129 | 2.35 | 1.4 | 1.13 | 0.94 | 0.8 | 1.58 | 2.22 | 5.07 | 2.36 | 1.92 | 4.44 | 1.62 | LOC100737965 |
| gene116 | 1.006830326 | 0.00166804 | 33.97 | 18.15 | 23.87 | 18.59 | 21.51 | 30.7 | 23.43 | 31.01 | 18.93 | 34.15 | 54.89 | 46.67 | AKAP12 |
| gene11619 | 2.095926813 | 0.000297848 | 0.27 | 0.31 | 0.38 | 0.2 | 0.14 | 0.08 | 0.33 | 1.06 | 0.41 | 0.78 | 0.68 | 0.17 | LOC100516620 |
| gene11620 | 3.36438863 | 6.82E-05 | 0.15 | 0.13 | 0.41 | 0.12 | 0.06 | 0 | 0 | 0.49 | 0.18 | 0.59 | 0.81 | 0.28 | CRISP2 |
| gene11624 | 2.650639606 | 2.42E-07 | 1.02 | 1.44 | 0.2 | 0 | 0.77 | 0.07 | 0.76 | 0.98 | 1.43 | 0.67 | 0.41 | 4.36 | CRISP3 |
| gene11644 | -1.00258032 | 0.003352348 | 4.12 | 5.21 | 5.18 | 9.45 | 7.66 | 6.11 | 1.29 | 10.91 | 6.16 | 7.51 | 3.08 | 1.02 | PAQR8 |
| gene11690 | 1.655304495 | 1.73E-06 | 1.19 | 0.4 | 0.81 | 0.43 | 0.63 | 0.76 | 3.33 | 1.71 | 3.31 | 1.18 | 3.23 | 1.06 | ADAMTSL3 |
| gene11693 | 2.067959935 | 3.08E-06 | 0.29 | 0.14 | 0.17 | 0.19 | 0.12 | 0.14 | 0.23 | 0.33 | 0.07 | 0.46 | 0.71 | 0.6 | LOC100156444 |
| gene11705 | 1.128958723 | 0.005508926 | 0.44 | 0.48 | 0.27 | 0.31 | 0.22 | 0.23 | 0.78 | 1.18 | 0.46 | 0.72 | 0.61 | 0.33 |  |
| gene11743 | 1.591019376 | 0.006540354 | 0.01 | 0 | 0.01 | 0.01 | 0.05 | 0.02 | 0.06 | 0.02 | 0.01 | 0.11 | 0.04 | 0.1 | ACAN |
| gene11744 | 1.941130446 | 3.03E-08 | 7.06 | 2.47 | 1.85 | 1.44 | 3.5 | 1.79 | 3.69 | 9.16 | 4.08 | 4.86 | 14.62 | 5.32 | LOC100157078 |
| gene11746 | 1.101957924 | 0.000595398 | 135.81 | 114.11 | 161.4 | 91.53 | 73.82 | 76.96 | 43.96 | 193.22 | 166.34 | 150.71 | 211.53 | 137.71 | ABHD2 |
| gene1176 | 1.216395043 | 0.0004274 | 3.8 | 0.9 | 0.88 | 1.35 | 1.56 | 2.11 | 4.38 | 3.33 | 1.25 | 2.39 | 5.05 | 3.62 | LOC100627025 |
| gene11765 | -1.450522771 | 0.002827583 | 0.8 | 1.13 | 0.93 | 1.58 | 1.67 | 0.94 | 0.92 | 1.07 | 1.49 | 0.41 | 0.63 | 0.43 | LOC100625251 |
| gene11768 | -2.16109112 | 8.47E-05 |  |  |  |  |  |  |  |  |  |  |  |  |  |
| gene11818 | -2.078788218 | 3.21E-09 | 1.94 | 2.56 | 1.5 | 1.4 | 11.07 | 2.37 | 1.16 | 0.37 | 1.97 | 1.43 | 0.58 | 1.51 | ISLR2 |
| gene11822 | -1.710155078 | 2.19E-06 | 0.76 | 1.77 | 3.72 | 4.3 | 2.37 | 3.95 | 1.56 | 0.56 | 1.61 | 1.25 | 0.57 | 1.35 | LOXL1 |
| gene11872 | 1.530249166 | 2.34E-06 | 32.7 | 43.94 | 36.19 | 26.11 | 17.64 | 29.43 | 21.4 | 75.06 | 55.83 | 40.67 | 89.82 | 69.89 | BAZ1A |
| gene11878 | 1.259591069 | 9.80E-05 | 53.98 | 81.18 | 38.58 | 28.61 | 86.25 | 23.86 | 60.38 | 57.23 | 90.87 | 53.69 | 84.98 | 185.15 | LOC100152368 |
| gene11880 | 1.924546643 | 9.01E-06 | 0.23 | 0.7 | 0.99 | 0.48 | 0.92 | 0.91 | 1.19 | 1.49 | 1.6 | 3.23 | 3.06 | 2.45 | NPAS3 |
| gene11950 | -1.761142667 | 0.002364822 | 0.34 | 0.33 | 0.16 | 0.33 | 0.37 | 0.44 | 0.16 | 0.1 | 0 | 0.07 | 0.06 | 0.17 | JPH4 |
| gene12 | -1.009590493 | 0.006108881 | 1.69 | 1.41 | 1.19 | 0.59 | 3.42 | 2.9 | 1.42 | 0.79 | 1.7 | 1.45 | 0.99 | 0.95 | LOC100621157 |
| gene120 | 1.024371872 | 0.002323458 | 7.8 | 2.76 | 5.11 | 4.65 | 3.85 | 1.47 | 1.17 | 8.14 | 3.77 | 5.58 | 11.41 | 2.71 |  |
| gene12022 | 1.602972887 | 8.53E-07 | 179.16 | 96.88 | 74.71 | 41.41 | 40.04 | 71.89 | 77.49 | 147.63 | 93.52 | 90.06 | 197.08 | 153.48 | PNP |
| gene12182 | -1.355899683 | 0.009920114 | 0.17 | 0.16 | 0.31 | 0.59 | 0.42 | 1.07 | 0.32 | 0.46 | 0.15 | 0.33 | 0.27 | 0.19 | ST8SIA2 |
| gene12184 | -1.731201865 | 4.74E-05 | 0.59 | 0.52 | 0.6 | 0.81 | 1.49 | 0.75 | 1.19 | 0.1 | 0.35 | 0.15 | 0.38 | 0.37 | SV2B |
| gene12185 | 1.151962922 | 0.002950621 | 0.13 | 0.12 | 0.4 | 0.57 | 1.28 | 0.22 | 0.8 | 1.6 | 1.16 | 1.65 | 1.24 | 1.67 |  |
| gene1221 | 1.177327432 | 0.000275812 | 96.13 | 52.1 | 56.51 | 51.98 | 45.06 | 41.69 | 110.18 | 66.47 | 86.03 | 76.37 | 94.17 | 131.31 | ARF6 |
| gene12213 | 1.048381747 | 0.001147866 | 41.46 | 39.93 | 34.29 | 37.87 | 24.32 | 21.16 | 45.06 | 51.02 | 59.77 | 39.47 | 67.03 | 58.94 | PLEK2 |
| gene12224 | 1.409361962 | 1.26E-05 | 453.18 | 498.32 | 405.06 | 258.69 | 272.91 | 249.3 | 571.34 | 652.69 | 612.64 | 715.16 | 762.57 | 529.94 | ZFP36L1 |
| gene12226 | 1.08018653 | 0.000761898 | 45.4 | 95.93 | 105.58 | 66.58 | 58 | 73.21 | 83.65 | 105.38 | 99.97 | 118.87 | 130.01 | 152.9 | ACTN1 |
| gene12237 | -2.351983649 | 1.25E-11 | 1.18 | 4.17 | 4.24 | 10.48 | 7.38 | 8.68 | 3.54 | 0.96 | 4.08 | 1.84 | 1.19 | 2.02 | SMOC1 |
| gene12241 | 2.200552496 | 0.003333236 | 0.03 | 0.13 | 0.12 | 0.05 | 0.02 | 0.05 | 0.08 | 0.2 | 0.12 | 0.28 | 0.25 | 0 | LOC100514528 |
| gene12248 | 1.686686898 | 4.18E-07 | 4.76 | 3.89 | 2.7 | 3.15 | 2.05 | 3.84 | 6.9 | 10.99 | 7.36 | 6.36 | 10.31 | 10.95 |  |
| gene12249 | 1.513518926 | 4.48E-06 | 18.56 | 14.79 | 10.56 | 9.43 | 5.43 | 12.12 | 16.59 | 18.88 | 21.92 | 17.18 | 24.31 | 31.63 |  |
| gene12255 | -1.386301707 | 4.78E-05 | 5.96 | 5.26 | 4.63 | 13.04 | 7.17 | 8.22 | 2.55 | 10.37 | 6.49 | 5.86 | 3.05 | 1.82 |  |
| gene12286 | -1.033599085 | 0.001408624 | 4.14 | 4 | 7.07 | 4.2 | 11.13 | 8.68 | 2.05 | 0.82 | 6.19 | 5.58 | 2.97 | 3 | LOC100514300 |
| gene12300 | 2.077016718 | 2.94E-10 | 162.82 | 207.56 | 197.92 | 52.67 | 138.1 | 76.88 | 316.59 | 251.38 | 128.23 | 251.07 | 277.55 | 568.05 | FOS |
| gene12301 | -1.077825773 | 0.000813561 | 29.4 | 25.77 | 33.18 | 53.79 | 76.11 | 43.85 | 58.47 | 48.58 | 25.76 | 26.07 | 29.22 | 24.81 | FLVCR2 |
| gene12312 | -1.600557649 | 5.86E-05 | 0.75 | 0.46 | 0.88 | 1.45 | 3.16 | 1.16 | 1.18 | 0.35 | 0.49 | 0.68 | 0.64 | 0.6 | ESRRB |
| gene12323 | 1.548263121 | 0.003298513 | 1.63 | 0.18 | 0.06 | 0.26 | 0.17 | 0.43 | 0.24 | 0.46 | 0.13 | 1.99 | 0.41 | 0.21 | LOC100626607 |
| gene12333 | -1.663873865 | 4.04E-05 | 1.66 | 1.65 | 1.78 | 2.52 | 2.19 | 2.28 | 1.45 | 0.23 | 1.57 | 0.51 | 0.29 | 1.34 | AHSA1 |
| gene12340 | -1.198001792 | 0.000414832 | 7.7 | 5.88 | 4.64 | 3.47 | 5.7 | 30.22 | 1.76 | 3.62 | 2.81 | 8.25 | 4.72 | 3.44 | DIO2 |
| gene12360 | -1.15275952 | 0.000844709 | 3.85 | 2.48 | 2.46 | 7.3 | 2.52 | 3.84 | 5.22 | 4.09 | 3.39 | 1.8 | 2.57 | 1.54 | KCNK10 |
| gene12388 | 2.509794897 | 4.20E-12 | 0.78 | 1.19 | 1.76 | 0.21 | 0.25 | 1.42 | 4.87 | 7.42 | 2.78 | 2.09 | 4.04 | 3.73 | LOC100512175 |
| gene12410 | -1.376393669 | 0.000543239 | 0.47 | 0.34 | 0.29 | 0.91 | 0.35 | 0.23 | 0.13 | 1.03 | 0.47 | 0.25 | 0.25 | 0.06 |  |
| gene12414 | -1.852941269 | 0.000144845 | 1.99 | 1.21 | 0.51 | 0.8 | 1.25 | 0.93 | 1.25 | 0.71 | 0.21 | 0.53 | 0.19 | 0.08 | LOC100627413 |
| gene12420 | -1.863635841 | 1.27E-08 | 487.73 | 338.68 | 493.58 | 833.15 | 609.85 | 351.54 | 200.4 | 211.07 | 161.91 | 166.08 | 209.61 | 102.92 | ISG12(A) |
| gene12421 | -1.194958703 | 0.000233176 | 93.82 | 104.63 | 134.51 | 100.82 | 150.69 | 103.1 | 56.21 | 67.62 | 63.69 | 28.37 | 63.49 | 57.15 | LOC100152306 |
| gene12429 | -3.139036747 | 6.27E-20 | 183.69 | 965.76 | 2014 | 1365.4 | 2375 | 4515.9 | 2505.8 | 138.43 | 226 | 225.45 | 486.07 | 174.87 | UFBP |
| gene12430 | -1.38823796 | 0.000116343 | 2.18 | 1.48 | 3.62 | 2.04 | 3.03 | 8.52 | 13.71 | 0.53 | 0.99 | 0.73 | 2.97 | 1.24 | LOC100155953 |
| gene12431 | -1.913736375 | 5.08E-09 | 1053.2 | 967.41 | 962.04 | 1293.7 | 1280.4 | 2335.6 | 2779.8 | 243.3 | 215.25 | 222.56 | 750.27 | 253.22 | UABP-2 |
| gene12435 | 1.093107801 | 0.005512045 | 7.59 | 0.05 | 10.34 | 0.22 | 1.73 | 0.96 | 4.26 | 1.6 | 1.23 | 4.27 | 0.99 | 0.9 | SERPINA3-1 |
| gene12436 | 1.077958925 | 0.001865294 | 0.12 | 0.02 | 0.08 | 2.28 | 3.53 | 1.16 | 1.05 | 4.16 | 0.47 | 10.56 | 2.63 | 1.66 | LOC100153899 |
| gene12437 | 3.475455551 | 1.43E-19 | 0.93 | 0.07 | 1.55 | 0.9 | 0.33 | 1.1 | 7.42 | 4.36 | 2.08 | 19.5 | 1.55 | 4.58 | SERPINA3-3 |
| gene12439 | 2.687849606 | 5.39E-14 | 0.89 | 0.04 | 1.48 | 1.48 | 2.34 | 1.47 | 18.12 | 3.53 | 2.95 | 17.14 | 3.41 | 13.3 | LOC100156325 |
| gene12440 | 2.030059007 | 7.22E-10 | 112.78 | 183.88 | 162.31 | 50.09 | 53.83 | 9.89 | 192.07 | 82.21 | 80.6 | 216.34 | 99.74 | 146.15 | SERPINA3-2 |
| gene12449 | -1.004035894 | 0.002161229 | 64.82 | 38.05 | 54.58 | 58.41 | 42.56 | 44.15 | 32.65 | 49.08 | 25.62 | 30.38 | 16.94 | 23.3 | LOC100154744 |
| gene12466 | -1.058063101 | 0.001044836 | 38.64 | 32.5 | 65.48 | 30.1 | 70.81 | 32.1 | 14.95 | 15.67 | 15.31 | 28.26 | 15.98 | 18.94 | LOC100625046 |
| gene12474 | 1.561284385 | 2.32E-06 | 24.43 | 17.44 | 20.35 | 7.31 | 11.8 | 5.35 | 5.45 | 4.07 | 27.05 | 19.61 | 16.48 | 34.31 | LOC100513744 |
| gene12480 | -1.18325696 | 0.000493093 | 8.8 | 16.24 | 10.7 | 10.07 | 11.17 | 8.6 | 9.12 | 4.24 | 6.13 | 4.12 | 2.94 | 5.69 | LOC100739812 |
| gene1250 | 1.487682123 | 0.0009321 | 0.99 | 1.5 | 0.41 | 0.8 | 0.93 | 0.98 | 0.7 | 0.84 | 0.78 | 0.81 | 2.83 | 3.25 |  |
| gene12512 | -1.031296364 | 0.001995445 | 28.81 | 39.99 | 42.03 | 52.27 | 55.08 | 59.05 | 36.85 | 28.1 | 36.15 | 21.69 | 32.36 | 24.29 | LOC100521669 |
| gene1253 | 1.079862637 | 0.001468173 | 2.52 | 2.84 | 3.52 | 3 | 4.08 | 3.52 | 5.12 | 5.38 | 4.85 | 5.64 | 5.78 | 10.27 | BMP4 |
| gene12538 | -2.934760401 | 1.88E-09 | 0.37 | 0.35 | 1.34 | 1.93 | 0.53 | 2.37 | 0.94 | 0.09 | 0 | 0.27 | 0.14 | 0.25 | LOC100624633 |
| gene12625 | -1.090198114 | 0.003457232 | 4.24 | 1.11 | 2.24 | 3.21 | 2.17 | 1.93 | 0.57 | 2.97 | 0.99 | 2.62 | 0.47 | 0.4 | GCM1 |
| gene12669 | -1.214170505 | 0.00040247 | 1.81 | 3.05 | 3.12 | 1.96 | 3.88 | 3.89 | 2.17 | 1.44 | 1.16 | 1.93 | 0.97 | 1.22 | LOC100620576 |
| gene12670 | 2.849963093 | 0.000339779 | 0.16 | 0.01 | 0.05 | 0.04 | 0 | 0.02 | 0.1 | 0.18 | 0.15 | 0.23 | 0.11 | 0.17 | CRMP1 |
| gene12687 | 1.139638176 | 0.000499189 | 7.52 | 11.54 | 12.26 | 10.08 | 6.25 | 10.24 | 16.89 | 12.1 | 21.99 | 13.16 | 16.52 | 26.14 | AFAP1 |
| gene12689 | 1.043449357 | 0.002072664 | 1.17 | 1.9 | 1.34 | 1.19 | 2.15 | 1.56 | 3.22 | 2.42 | 2.43 | 3.36 | 2.92 | 3.53 | LOC100522624 |
| gene12691 | -1.054302076 | 0.001366292 | 2.46 | 2.95 | 3.45 | 4.55 | 5.57 | 4.61 | 6.15 | 1.97 | 4.2 | 2.12 | 2.22 | 2.54 | LOC100737944 |
| gene12716 | -1.202128906 | 0.000746201 | 4.32 | 1.98 | 2.12 | 3.04 | 10.74 | 3.75 | 9.54 | 4.17 | 2.79 | 3.39 | 1.43 | 2.84 | C1QTNF7 |
| gene12720 | -1.278745621 | 0.000139087 | 14.09 | 12.65 | 14.38 | 11.95 | 8.45 | 22.21 | 9.99 | 5.54 | 12.89 | 11.09 | 3.6 | 2.52 | LOC100625942 |
| gene12727 | -1.115348759 | 0.000699068 | 19.26 | 9.34 | 20.48 | 30.21 | 29.71 | 23.64 | 10.97 | 35.11 | 8.39 | 18 | 8.94 | 10.97 | LOC100513100 |
| gene12728 | -1.37765842 | 9.88E-05 | 16.98 | 9.22 | 22.59 | 36.3 | 29.55 | 30.54 | 10.25 | 38.38 | 10.65 | 15.28 | 12.04 | 8.92 | LOC100627218 |
| gene12730 | -1.013330368 | 0.002229761 | 21.77 | 16.87 | 21.74 | 21.33 | 19.56 | 25.44 | 18.18 | 15.62 | 9.05 | 13.98 | 9.47 | 8.42 | QDPR |
| gene12736 | -1.705010123 | 0.0003009 | 0.46 | 0.14 | 0.71 | 0.47 | 0.29 | 0.32 | 0.05 | 0.08 | 0.04 | 0.19 | 0.1 | 0.04 |  |
| gene12738 | -1.609148962 | 0.000397114 | 2.35 | 5.38 | 5.27 | 4.31 | 5.59 | 7.42 | 3.31 | 2 | 1.54 | 0.96 | 3.82 | 0.63 | LOC100513483 |
| gene12739 | -1.289378768 | 0.000150257 | 2.36 | 3.97 | 2.16 | 2.73 | 2.75 | 5.55 | 1.8 | 2.68 | 3.74 | 1.91 | 1.56 | 0.87 | NCAPG |
| gene12756 | -1.239946552 | 0.00011807 | 48.59 | 68.56 | 111.15 | 77.69 | 95.31 | 112.03 | 90.01 | 21.63 | 36.16 | 38.58 | 34.99 | 42.86 | LOC100621948 |
| gene12768 | 1.513839016 | 0.003298513 | 0.24 | 0.18 | 0.47 | 0.31 | 0.31 | 0.09 | 0.52 | 0.31 | 0.31 | 1.15 | 0.19 | 0.67 | LOC100623128 |
| gene1279 | 1.443698522 | 0.000138121 | 3.22 | 3.42 | 3.19 | 2.18 | 0.92 | 0.79 | 1.32 | 4.84 | 3.36 | 3.68 | 2.09 | 4.3 | LOC100513515 |
| gene12799 | -2.216695058 | 5.75E-07 | 0.76 | 0.69 | 1.07 | 2.31 | 0.83 | 3.48 | 1 | 1.39 | 0.63 | 0.92 | 0.19 | 0.23 | LOC100525349 |
| gene12830 | -1.512492866 | 0.000344835 | 4.55 | 4.6 | 6.65 | 6.42 | 7.29 | 6.37 | 1.25 | 6.36 | 3.63 | 3.18 | 2.45 | 1.49 | LOC100737157 |
| gene12831 | -1.002261774 | 0.007294474 | 6.09 | 3.48 | 4.48 | 6.92 | 4.7 | 2.05 | 2.59 | 5.37 | 2.91 | 3.44 | 1.09 | 2.29 | LOC100621918 |
| gene12832 | -1.103272672 | 0.000639301 | 29.4 | 20.4 | 19.73 | 50.77 | 24.25 | 19.18 | 6.01 | 27.92 | 22.57 | 21.07 | 11.34 | 10.62 | ATP8A1 |
| gene12850 | 1.317799113 | 0.000330417 | 0.72 | 0.75 | 0.66 | 0.45 | 0.56 | 0.51 | 0.43 | 0.67 | 0.66 | 1.43 | 0.75 | 1.48 | LOC100738048 |
| gene12856 | 2.003966725 | 1.65E-07 |  |  |  |  |  |  |  |  |  |  |  |  |  |
| gene12901 | -2.974761244 | 0.001195451 | 0.28 | 0.51 | 0.51 | 0.39 | 0.64 | 0.56 | 0.18 | 0.23 | 0.49 | 0.09 | 0.09 | 0 | LOC100736721 |
| gene12917 | -1.101364779 | 0.000707334 | 8.56 | 8.29 | 10.76 | 9.3 | 33.81 | 11.96 | 11.55 | 8.49 | 5.58 | 8.89 | 6.51 | 9.97 | GUCY1A3 |
| gene12928 | -1.586056107 | 0.000651928 | 2.48 | 1.68 | 0.41 | 4.52 | 9.21 | 3.48 | 2.82 | 5.5 | 1.75 | 1.54 | 1.97 | 2.33 | LOC100516145 |
| gene12932 | -1.031742783 | 0.0021555 | 2.02 | 2.28 | 2.96 | 3 | 8.01 | 2.87 | 3.35 | 3.96 | 2.04 | 2.89 | 1.26 | 2.61 | LOC100623130 |
| gene12939 | 4.655276992 | 0.005776466 |  |  |  |  |  |  |  |  |  |  |  |  |  |
| gene12959 | -1.353909359 | 0.000342052 | 7.23 | 9.53 | 13.11 | 19.28 | 15.11 | 13.43 | 10.17 | 9.64 | 7.46 | 9.41 | 5.91 | 3.42 | HOPX |
| gene13005 | -1.184284339 | 0.001017073 | 2.03 | 3.02 | 3.58 | 3.45 | 3.55 | 4.17 | 2.88 | 0.9 | 1.41 | 2.8 | 0.71 | 1.38 | TMPRSS11A |
| gene13007 | -1.669779742 | 2.64E-06 | 13.81 | 6.74 | 1.72 | 8.37 | 3.41 | 5.5 | 3.92 | 1.42 | 10.16 | 0.41 | 1.14 | 3.56 | LOC100738056 |
| gene13011 | -1.057886887 | 0.001102272 | 6.19 | 21.66 | 25.99 | 37.58 | 33.72 | 29.66 | 27.62 | 6.9 | 11.33 | 13.87 | 12.28 | 20.79 | LOC100513671 |
| gene13021 | 2.533923911 | 0.000328194 |  |  |  |  |  |  |  |  |  |  |  |  |  |
| gene13027 | 2.063411014 | 7.23E-07 | 1.04 | 0.61 | 0.16 | 0.39 | 0.28 | 0.48 | 0.53 | 0.92 | 1.98 | 2.43 | 0.98 | 1.26 | UGT2A1 |
| gene13041 | 1.819717973 | 1.37E-06 | 9.5 | 17.03 | 11.97 | 2.49 | 3.31 | 6.85 | 9.44 | 7.99 | 15.32 | 18.31 | 11.25 | 12.33 | LOC100739482 |
| gene13042 | 1.572019375 | 3.75E-06 | 3.21 | 5.5 | 3.43 | 0.79 | 1.34 | 2.41 | 2.4 | 3.93 | 2.43 | 6.57 | 3.05 | 3.41 | SLC4A4 |
| gene13043 | -5.906170542 | 4.28E-20 | 0.18 | 0.2 | 0.05 | 2.53 | 2.08 | 3.51 | 0.69 | 0.04 | 0 | 0 | 0 | 0.1 | LOC100739516 |
| gene13050 | 1.348810762 | 3.57E-05 | 0.1 | 0.23 | 0.25 | 0.88 | 6.12 | 26.56 | 0.27 | 0.07 | 0.11 | 84.56 | 0.13 | 0.27 | ALB |
| gene13056 | 1.42398173 | 8.19E-05 | 2.57 | 14.79 | 3.7 | 2.52 | 6.54 | 6.03 | 4.73 | 8.64 | 8.05 | 16.02 | 10.81 | 12.42 | AMCF-II |
| gene13061 | 1.764764722 | 8.38E-07 | 14.09 | 24.59 | 14.96 | 5.46 | 7.6 | 5.07 | 11.26 | 29.8 | 10.03 | 14.39 | 25.5 | 19.44 | CXCL2 |
| gene13064 | 1.229475441 | 0.000393533 | 12.44 | 8.77 | 14.03 | 4.1 | 3.83 | 4.01 | 6.35 | 5.28 | 8.29 | 9.21 | 7.31 | 10.53 | AREG |
| gene1308 | 1.108846092 | 0.000634556 | 23.79 | 20.04 | 22.99 | 25.68 | 21.7 | 22.88 | 22.87 | 42.31 | 50.52 | 32.53 | 51.94 | 60.62 | LOC100738515 |
| gene1309 | -8.381820577 | 1.99E-19 | 6.18 | 6.51 | 0.15 | 0 | 15.44 | 3.97 | 4.14 | 0 | 0.2 | 0 | 0 | 0 | MNAT1 |
| gene13111 | 2.110347164 | 4.20E-05 | 0.09 | 0.21 | 0.27 | 0.1 | 0.39 | 0 | 1.89 | 0 | 0 | 2.06 | 0.05 | 0.12 | FGG |
| gene13112 | 1.16400593 | 0.000799308 | 0.21 | 0.32 | 0.85 | 2.1 | 1.03 | 2.8 | 5.59 | 0.04 | 0.08 | 12.99 | 0.11 | 0.24 | LOC100626178 |
| gene13122 | 2.416585356 | 0.005219397 |  |  |  |  |  |  |  |  |  |  |  |  |  |
| gene13148 | -1.058899313 | 0.007677925 | 4.4 | 4 | 2.91 | 5.85 | 4.32 | 4.64 | 9.83 | 2.3 | 3.14 | 1.27 | 2.82 | 2.81 | NR3C2 |
| gene13174 | -1.352695966 | 0.000219265 | 1.56 | 0.82 | 3.52 | 2.8 | 0.85 | 3.84 | 3.13 | 1.06 | 2.99 | 0.86 | 1.1 | 0.82 | HHIP |
| gene1318 | 1.357929829 | 2.77E-05 | 44.17 | 47.04 | 42.92 | 37.71 | 25.72 | 32.8 | 33 | 67.94 | 62.39 | 54.05 | 84.83 | 96.73 | LOC100521309 |
| gene13193 | -1.921943729 | 0.000433916 | 0.33 | 0.47 | 0.19 | 0.22 | 0.25 | 2.22 | 0.15 | 0.08 | 0.38 | 0.24 | 0.12 | 0.26 | LOC100512242 |
| gene13265 | 2.503045 | 0.000211552 | 0.28 | 0.46 | 0.69 | 0.11 | 0.05 | 0.21 | 0 | 0.55 | 0.08 | 0.31 | 1.19 | 0.54 | LOC100738704 |
| gene13275 | -5.352372547 | 0.000178599 | 0.46 | 0.2 | 0.5 | 1.57 | 0.33 | 0.18 | 0.17 | 2.19 | 0.39 | 0.11 | 0.11 | 0 | FABP2 |
| gene13277 | 1.096973726 | 0.000629758 | 37.69 | 38.1 | 46.3 | 36.56 | 23.64 | 34.89 | 35.79 | 50.71 | 57.9 | 39.29 | 86.08 | 67.78 | USP53 |
| gene13303 | -1.436720846 | 0.000798635 | 0.22 | 0.53 | 0.23 | 1.73 | 2.99 | 0.94 | 1.56 | 2.13 | 0.61 | 1.13 | 0.34 | 0.56 | LOC100513102 |
| gene13312 | -1.078941191 | 0.000854169 | 17.08 | 29.24 | 43.69 | 43.56 | 33.28 | 78.59 | 34.97 | 20.71 | 51.26 | 30.3 | 20.19 | 20.29 | PITX2 |
| gene13313 | -1.421454601 | 0.00015432 | 2.76 | 3.84 | 3.6 | 8.93 | 3.6 | 3.79 | 3.55 | 2.82 | 4.09 | 1.85 | 2.13 | 2.02 | LOC100737472 |
| gene13321 | -1.419288068 | 1.38E-05 | 23.26 | 18.86 | 8.8 | 13.58 | 16.43 | 52.05 | 6.7 | 11.29 | 5.71 | 11.85 | 9.54 | 7.89 | LOC100516921 |
| gene13327 | -3.807804025 | 7.03E-27 | 67.96 | 11.13 | 82.67 | 222.25 | 554.77 | 178.11 | 374.38 | 44.68 | 29.8 | 15.03 | 24.01 | 27.48 | LOC100519324 |
| gene1335 | -4.104290196 | 3.66E-26 | 1.96 | 0.63 | 1.94 | 7.21 | 26.26 | 5.59 | 12.12 | 2.63 | 1.33 | 0.62 | 1.05 | 0.58 | ESR2 |
| gene13367 | 4.521496033 | 2.05E-13 | 0.09 | 0 | 0.12 | 0.2 | 0.11 | 0.27 | 1.63 | 0.1 | 1.29 | 5.7 | 1.6 | 4.09 | UBE2D2 |
| gene13371 | -1.636679628 | 4.95E-07 | 83.12 | 45.02 | 52.58 | 131.45 | 118.39 | 209.71 | 188.97 | 50.35 | 19.57 | 51.25 | 52.39 | 38.17 | LOC100524695 |
| gene13376 | -1.516305201 | 7.41E-05 | 4.94 | 5.85 | 3.47 | 5.05 | 18.18 | 13.86 | 10.16 | 3.88 | 1.92 | 5.8 | 2.6 | 4.33 | LOC100628129 |
| gene13379 | -3.773115179 | 1.16E-19 | 15.08 | 18.59 | 9.91 | 8.78 | 35.55 | 118.16 | 17.31 | 4.11 | 1.38 | 4.27 | 5.4 | 1.88 | LOC100620154 |
| gene13392 | -1.093111024 | 0.006441643 | 0.86 | 1.57 | 0.75 | 1.46 | 4.83 | 1.57 | 1.55 | 1.98 | 0.41 | 2.04 | 0.67 | 1.06 | ADH1C |
| gene13412 | -1.596778527 | 6.63E-06 |  |  |  |  |  |  |  |  |  |  |  |  |  |
| gene13418 | -4.028695229 | 3.12E-22 | 0.17 | 5.68 | 4.1 | 0.94 | 1.72 | 5.16 | 2.3 | 0.12 | 0.33 | 0.3 | 0.08 | 0.1 | MMRN1 |
| gene1342 | 3.335714174 | 0.00611413 | 0.19 | 0.47 | 0.13 | 0 | 0 | 0.14 | 0.16 | 0.1 | 0 | 0.31 | 0.15 | 1.12 | LOC100526029 |
| gene13424 | 1.32505447 | 3.95E-05 | 123.12 | 135.04 | 121.87 | 54.26 | 84.44 | 58.91 | 64.13 | 70.53 | 145.9 | 121.79 | 129 | 228.49 | LOC100517722 |
| gene13428 | -1.542198053 | 5.87E-06 | 13.47 | 17.7 | 12.08 | 30.37 | 15.41 | 33.05 | 12.09 | 9.81 | 7.72 | 12.24 | 9.07 | 5 | LOC100518083 |
| gene13435 | -1.353850335 | 2.62E-05 | 48621 | 33779 | 50794 | 31893 | 66258 | 67376 | 49481 | 13274 | 16602 | 18696 | 22008 | 21711 | SPP1 |
| gene13443 | 1.090717163 | 0.004002967 | 0.8 | 0.79 | 1.11 | 1.09 | 1.64 | 2.01 | 0.67 | 1.63 | 4.17 | 4.21 | 1.65 | 3.95 | HSD17B13 |
| gene13487 | 1.162270864 | 0.000394689 | 2.7 | 5.01 | 4.51 | 4.55 | 5.13 | 4.54 | 4.57 | 5.75 | 8.71 | 8.75 | 9.27 | 12.76 | PRKG2 |
| gene13488 | -1.999229123 | 1.70E-06 | 0.45 | 1.02 | 0.74 | 2.63 | 0.64 | 2.18 | 0.64 | 0.48 | 0.69 | 0.75 | 0.42 | 0.19 | BMP3 |
| gene1353 | -2.387084908 | 1.14E-05 | 1.43 | 1.58 | 1 | 1.53 | 1.44 | 2.37 | 1.15 | 0.37 | 0.32 | 0.2 | 0.39 | 0.48 | LOC100513000 |
| gene13531 | -1.067973297 | 0.001347575 | 22.64 | 16.3 | 23.54 | 31.67 | 26.18 | 26.61 | 27.72 | 14.23 | 15.07 | 12.84 | 11.14 | 15.05 | LMO1 |
| gene13533 | -1.279082979 | 0.003826756 | 0.25 | 0.55 | 0.46 | 0.92 | 1.26 | 0.61 | 0.34 | 0.13 | 0.26 | 0.16 | 0.31 | 0.64 | TUB |
| gene13565 | -1.6982226 | 0.008591092 | 0.42 | 0.25 | 0.89 | 1.53 | 1.59 | 2.41 | 1.15 | 0.93 | 0.73 | 0 | 1.31 | 0.25 | LOC100622619 |
| gene13573 | -2.766550584 | 0.000103929 | 0.06 | 0.17 | 0.28 | 0.41 | 0.35 | 0.6 | 0.09 | 0.09 | 0.06 | 0.05 | 0.05 | 0.08 |  |
| gene13605 | -1.621568481 | 7.34E-07 | 18.52 | 8 | 26.91 | 24.21 | 44.04 | 21.56 | 11.84 | 8.4 | 9.76 | 9.31 | 8.16 | 11.1 | LOC100519163 |
| gene13613 | 2.412804053 | 0.001815837 | 0.06 | 0.11 | 0.15 | 0.14 | 0.14 | 0 | 0.38 | 0.74 | 0.72 | 0.76 | 0.46 | 0.5 | LOC100625698 |
| gene1362 | 1.353598637 | 0.002450308 | 1.99 | 4.54 | 2.14 | 3.58 | 2.79 | 1.18 | 3.34 | 9.24 | 8.24 | 5.26 | 9.09 | 4.53 | P14ARF |
| gene13657 | 1.653299704 | 0.002450843 | 0.79 | 0.22 | 1.05 | 0.56 | 0.1 | 0.17 | 0.58 | 1.06 | 0 | 1.49 | 0.77 | 0.22 | LOC100525239 |
| gene13839 | -2.379789495 | 7.33E-12 | 8.16 | 2.56 | 18.75 | 10.26 | 36.5 | 17.7 | 4.79 | 3.27 | 3.37 | 6.96 | 2.28 | 3.15 | LOC100513029 |
| gene13840 | -3.797718823 | 4.89E-25 | 12.68 | 2.37 | 29.43 | 24.58 | 58.41 | 33.24 | 5.4 | 4.8 | 2.96 | 3.84 | 1.26 | 3.25 | P2RY6 |
| gene13869 | -1.224474326 | 0.000980315 | 2.51 | 1.44 | 1.83 | 4.12 | 3.07 | 2 | 2.13 | 1.75 | 3.72 | 1.1 | 1.76 | 1.03 | LOC100739223 |
| gene13894 | -1.270166753 | 0.00040177 |  |  |  |  |  |  |  |  |  |  |  |  |  |
| gene13935 | 1.933082048 | 0.000163583 | 0.12 | 0.21 | 0.12 | 0.1 | 0.24 | 0.3 | 0.83 | 1.4 | 0.99 | 0.9 | 0.75 | 0.76 | LOC100517667 |
| gene13983 | 1.405277662 | 5.87E-05 | 0.77 | 1.67 | 1.64 | 0.82 | 1.35 | 1.5 | 2.63 | 2.16 | 1.63 | 2.15 | 3.9 | 3.15 | FOLH1 |
| gene13986 | 1.098545039 | 0.000621159 | 131.08 | 76.32 | 94.75 | 70.3 | 71.22 | 56.57 | 46.05 | 84.92 | 108.05 | 244.65 | 86.54 | 88.64 | LOC100737718 |
| gene14009 | -1.287816372 | 0.000187933 | 8.58 | 6 | 6.15 | 11 | 21.5 | 8.96 | 11.38 | 4.82 | 7.84 | 7.28 | 5.02 | 4.57 | LOC100516505 |
| gene14016 | -1.269725415 | 0.000157405 | 70.31 | 65.19 | 105.7 | 35.05 | 42.95 | 26.31 | 26.22 | 12.74 | 23.52 | 12.31 | 12.7 | 17.04 | LOC100517177 |
| gene14017 | -1.096856765 | 0.001471352 | 2.89 | 3.74 | 4.84 | 5.84 | 3.29 | 2.73 | 2.7 | 1.41 | 1.99 | 1.2 | 2.33 | 1.84 | LOC100513419 |
| gene14029 | -1.406463378 | 5.57E-05 | 6.8 | 3.02 | 3.69 | 4.09 | 10.06 | 8.51 | 3.8 | 4.68 | 2.07 | 2.78 | 3.97 | 1.59 | SESN3 |
| gene1404 | 3.099355943 | 0.000481426 | 0.13 | 0 | 0.36 | 0 | 0.16 | 0 | 1.34 | 0 | 0.34 | 0.74 | 0.2 | 1.01 | IFN-OMEGA-2 |
| gene14040 | 1.689999029 | 0.006337787 | 1.24 | 1.03 | 0.86 | 0.52 | 0.41 | 0.13 | 1.62 | 1.51 | 0.77 | 1.59 | 0.46 | 1.61 |  |
| gene14045 | 1.15362201 | 0.000380708 | 7.96 | 5.39 | 5.99 | 5.93 | 4.89 | 10.02 | 6.18 | 19.71 | 7.91 | 3.26 | 28.2 | 11.74 | TRPC6 |
| gene14046 | -1.846885531 | 4.16E-08 | 47.75 | 6.37 | 18.3 | 30.86 | 6.66 | 27.11 | 27.87 | 10.92 | 11.53 | 4.62 | 9.07 | 3.39 | ANGPTL5 |
| gene14055 | 1.324183022 | 4.63E-05 | 80.42 | 110.76 | 182.76 | 125.77 | 5.25 | 39.71 | 64.66 | 497.41 | 107.98 | 187.66 | 154.47 | 70.24 | MMP7 |
| gene14059 | 1.829300342 | 3.20E-08 | 13.13 | 92.52 | 25.83 | 16.66 | 6.5 | 7.42 | 4.69 | 24.53 | 15.24 | 48.25 | 29.89 | 27.74 | MMP1 |
| gene14060 | 1.167419062 | 0.000288217 | 48.2 | 75.28 | 41.63 | 35.95 | 42.9 | 35.13 | 39.73 | 29.39 | 48.83 | 94.49 | 81.3 | 73.16 | MMP3 |
| gene14061 | 2.089786152 | 8.07E-06 | 0.24 | 0.88 | 0.25 | 0.1 | 0.44 | 0.27 | 0.17 | 1.18 | 0.14 | 1.23 | 0.63 | 1.28 | MMP12 |
| gene14075 | -1.286659967 | 0.0007249 | 1.35 | 1.12 | 1.32 | 4.18 | 1.11 | 2.97 | 0.94 | 1.86 | 0.74 | 1.23 | 1.2 | 0.81 | CASP1 |
| gene14110 | -1.100258163 | 0.003043516 | 12.69 | 12.2 | 22.78 | 24.92 | 6.89 | 29.37 | 5.07 | 17.36 | 27.94 | 15.32 | 8.39 | 4.29 | LOC100620611 |
| gene14134 | 1.48792097 | 4.97E-06 | 40.45 | 55.6 | 82.12 | 48.08 | 28.43 | 47.87 | 60.76 | 110.85 | 141.01 | 62.24 | 110.24 | 158.77 | IL18 |
| gene14153 | -3.087494222 | 5.86E-07 | 0.21 | 0.19 | 0.69 | 2.4 | 0.12 | 5.54 | 0.17 | 0.11 | 0.55 | 0.34 | 0.49 | 0.15 | NNMT |
| gene14166 | 1.83506786 | 0.000355893 | 0 | 0 | 0.45 | 0.43 | 0.85 | 2.24 | 0.07 | 0.54 | 0 | 10.19 | 0 | 0.75 | APOC3 |
| gene14169 | 1.23661755 | 0.006737982 | 0.52 | 0.37 | 0.61 | 0.38 | 0.45 | 0.63 | 0.84 | 1.7 | 0.69 | 1.09 | 1.21 | 0.96 |  |
| gene14180 | -1.648894094 | 0.005097461 | 0.39 | 2.66 | 2.71 | 1.64 | 5.28 | 3.24 | 1.85 | 0.14 | 0.33 | 2.18 | 0.88 | 0.55 | FXYD2 |
| gene14181 | -1.045523931 | 0.006306612 | 2.37 | 1.12 | 2.99 | 2.75 | 1.69 | 1.21 | 1.11 | 2.42 | 1.09 | 1.18 | 0.93 | 0.58 | LOC100511523 |
| gene14191 | -1.891697586 | 0.002841191 | 1.25 | 0.93 | 0.55 | 1.43 | 0.7 | 1.43 | 1.21 | 1.2 | 0.76 | 0.22 | 0.33 | 0.32 | CD3G |
| gene14221 | -1.124221419 | 0.001612117 | 5.79 | 5.23 | 7.13 | 9.76 | 6.56 | 8.48 | 1.59 | 7.58 | 6.6 | 4.13 | 4.21 | 2.79 | LOC100522389 |
| gene14227 | -1.122310459 | 0.000771225 | 5.04 | 2.56 | 5.47 | 5.63 | 11.94 | 3.2 | 5.01 | 2.12 | 1.52 | 2.05 | 3.3 | 4 | LOC100520041 |
| gene14244 | 1.821328452 | 1.41E-05 | 0.16 | 0.48 | 0.17 | 0.32 | 0.48 | 0.93 | 1.25 | 3.64 | 1.63 | 1.85 | 2.48 | 1.3 | LOC100521388 |
| gene14323 | -2.755275471 | 0.001317472 | 0 | 0.19 | 0 | 0.58 | 0.37 | 0.03 | 0.07 | 0.45 | 0.35 | 0.16 | 0 | 0 | LOC100523913 |
| gene14354 | -1.680874878 | 0.001297567 | 1.11 | 2.43 | 4.94 | 4.8 | 2.57 | 5.37 | 1.92 | 1.95 | 2.6 | 0.91 | 1.47 | 1.33 | LOC100736666 |
| gene14369 | -2.124476857 | 8.79E-10 | 1.74 | 0.36 | 2.24 | 7.58 | 9.72 | 3.32 | 6.12 | 0.99 | 2.05 | 2.23 | 1.2 | 1.31 | KIRREL3 |
| gene14370 | 1.171789755 | 0.000277815 | 64.68 | 42.99 | 49.77 | 37.04 | 34.18 | 34.7 | 56.69 | 83.59 | 45.75 | 62.73 | 86.53 | 80 | ETS1 |
| gene14373 | 3.473972396 | 2.70E-22 | 15.97 | 38.16 | 5.4 | 3.86 | 1.43 | 6.72 | 2.31 | 44.68 | 22.04 | 43.52 | 26.55 | 56.23 | KCNJ1 |
| gene14378 | 1.34616722 | 6.70E-05 | 8.89 | 14.12 | 6.55 | 3.78 | 7.5 | 5.38 | 12.19 | 8.31 | 6.15 | 13.72 | 8.7 | 18.87 | LOC100516284 |
| gene14388 | -1.000242839 | 0.007220231 | 0.7 | 0.69 | 1.52 | 1.28 | 0.39 | 1.77 | 0.57 | 0.6 | 1.4 | 0.81 | 0.62 | 0.21 | LOC100518855 |
| gene14398 | -3.774031111 | 0.000110367 | 0.12 | 0.08 | 0 | 0.21 | 0.26 | 0.08 | 0.05 | 0.04 | 0.31 | 0 | 0 | 0.03 | LOC100626754 |
| gene14409 | 1.385856558 | 0.002069172 | 0.52 | 0.22 | 0.26 | 0.28 | 0.21 | 0.34 | 0.52 | 0.66 | 0 | 0.68 | 1.12 | 0.25 | GLB1L2 |
| gene14423 | -1.031921215 | 0.001753255 | 6.9 | 5.79 | 7.44 | 7.02 | 17.57 | 8.45 | 6.49 | 8.59 | 3.43 | 6.05 | 3.66 | 6.24 | FMO1 |
| gene14428 | 1.933650873 | 7.92E-09 | 10.81 | 8.16 | 8.63 | 4.39 | 8.23 | 9.09 | 5.45 | 6.3 | 8.14 | 27.19 | 21.2 | 31.66 | LOC100511032 |
| gene14430 | -1.189618991 | 0.001493219 | 1.67 | 1.2 | 1.26 | 1.73 | 1 | 5 | 0.84 | 1.61 | 0.9 | 0.77 | 1.36 | 1.06 | LOC100511526 |
| gene14439 | -1.425558028 | 0.000307065 | 1.02 | 1.22 | 1.69 | 2.35 | 2.14 | 1.38 | 1.72 | 0.29 | 0.91 | 0.48 | 0.56 | 1.1 | LOC100524822 |
| gene14485 | 2.138955926 | 0.009755884 | 0.19 | 0.04 | 0.25 | 0.15 | 0 | 0.18 | 0 | 0.13 | 0.33 | 0.5 | 0.29 | 0.77 | LOC100522695 |
| gene14487 | -2.113189214 | 0.000403113 | 0.18 | 0.12 | 0.11 | 0.05 | 0.18 | 0.98 | 0.35 | 0.04 | 0 | 0.16 | 0.06 | 0.03 | PIGR |
| gene14488 | -2.490697726 | 0.000620677 | 0.25 | 0.42 | 0.33 | 1.31 | 0.43 | 0.71 | 0.3 | 0.06 | 0 | 0 | 0.29 | 0.18 | LOC100739302 |
| gene14489 | -2.829273585 | 1.00E-05 | 0.1 | 0.08 | 0.24 | 0.13 | 0.13 | 1.16 | 0.6 | 0.17 | 0.15 | 0.07 | 0.01 | 0.11 | LOC100522888 |
| gene14490 | -1.509673149 | 6.56E-06 | 3.22 | 1.62 | 2.29 | 2.26 | 1.47 | 11.7 | 5.83 | 1.21 | 0.93 | 1.8 | 2.24 | 1.06 |  |
| gene14493 | 2.128978198 | 5.34E-10 | 26.83 | 7.8 | 8.6 | 2.13 | 3.21 | 8.53 | 9.42 | 10.05 | 11.54 | 8.08 | 22.5 | 25.83 | YOD1 |
| gene14500 | 1.015122608 | 0.001514236 | 161.17 | 203.72 | 198.93 | 168.14 | 158.98 | 165.42 | 192.5 | 285.19 | 232.8 | 196 | 516.12 | 235.65 | CD55 |
| gene14501 | 1.828019519 | 9.98E-08 | 0.46 | 0.7 | 0.68 | 1.28 | 0.73 | 1.59 | 5.47 | 5.28 | 1.8 | 3.24 | 5.16 | 3.68 | CR2 |
| gene14507 | -1.502922844 | 8.31E-06 | 6.57 | 18.66 | 9.49 | 6.05 | 12.33 | 11.54 | 3.41 | 4.25 | 1.67 | 5.99 | 2.29 | 2.17 | STEAP4 |
| gene14515 | 1.924453668 | 7.76E-08 | 11.43 | 27.93 | 12.88 | 3.38 | 3.92 | 4.3 | 2.48 | 44.91 | 13.77 | 25.22 | 5.3 | 13.1 | LOC100518208 |
| gene14559 | 1.77247146 | 0.00010805 | 0.76 | 0.31 | 0.29 | 0.76 | 0.31 | 0.68 | 0.04 | 1.39 | 1.23 | 2 | 1.43 | 2.39 | PON3 |
| gene14571 | 1.898242848 | 7.19E-05 | 2.06 | 2.83 | 2.52 | 1.47 | 0.44 | 0.7 | 0.7 | 0.1 | 1.18 | 4.5 | 1.98 | 3.37 | LOC100525179 |
| gene14580 | -1.07313509 | 0.007092972 | 2.24 | 1.87 | 1.98 | 3.3 | 1.72 | 2.03 | 0.73 | 1.22 | 1.96 | 1.21 | 0.98 | 1.07 | LOC100511893 |
| gene14588 | 1.171007395 | 0.000308842 | 43.81 | 38.62 | 35.02 | 36.87 | 23.76 | 35.78 | 30.98 | 56.43 | 55.34 | 44.32 | 66.53 | 96.05 | LOC595121 |
| gene14599 | -1.090190008 | 0.009901884 | 0.97 | 1.52 | 1.34 | 1.98 | 1.58 | 1.86 | 0.83 | 2.88 | 1.2 | 0.85 | 0.95 | 0.69 | LOC100623666 |
| gene14629 | 1.207754816 | 0.000250601 | 1.42 | 1.37 | 1.59 | 0.6 | 0.55 | 1.03 | 0.32 | 0.91 | 1.11 | 1.32 | 1.23 | 2.25 |  |
| gene14660 | -1.157514345 | 0.000500986 | 4.22 | 3.18 | 4.58 | 5.75 | 3.69 | 9.51 | 2.57 | 2.17 | 2.07 | 2.32 | 3.99 | 1.76 | LOC100523199 |
| gene14661 | -2.086394715 | 5.05E-06 | 0.13 | 0.29 | 0.9 | 0.75 | 0.78 | 1.27 | 1.14 | 0.49 | 0.2 | 0.27 | 0.18 | 0.21 | GRM3 |
| gene14667 | 1.154735783 | 0.002566132 | 1.03 | 3.25 | 1.53 | 1.34 | 0.77 | 2.73 | 2.05 | 2.48 | 2.74 | 3.28 | 5.15 | 1.67 | LOC100739844 |
| gene14691 | -1.536204698 | 3.25E-05 | 1.41 | 1.14 | 4.23 | 4.82 | 3.25 | 9.57 | 2.05 | 2.85 | 2.59 | 2.23 | 2.32 | 1.4 | LOC100737774 |
| gene1470 | 1.102219803 | 0.00171551 | 2.38 | 0.53 | 1.05 | 0.77 | 0.57 | 1.46 | 1.97 | 3.07 | 0.56 | 2.38 | 3.02 | 0.32 | FREM1 |
| gene14720 | 1.33321404 | 7.23E-05 | 30.3 | 28.2 | 23.91 | 27.7 | 37.42 | 19.08 | 58.17 | 67.78 | 68.07 | 54.18 | 84.13 | 66.9 | LOC100517670 |
| gene14725 | -1.703787581 | 0.000737041 | 1.43 | 1.42 | 1.51 | 1.44 | 1.9 | 0.84 | 0.95 | 0.49 | 1.65 | 0.27 | 0.33 | 0.66 | LOC100518922 |
| gene14736 | -1.647183222 | 7.96E-05 | 1.65 | 0.55 | 0.51 | 2.86 | 0.15 | 0.43 | 0.8 | 0.63 | 0.2 | 0.31 | 0.39 | 0.38 | SLC26A3 |
| gene14743 | -1.370677031 | 9.16E-05 | 1.44 | 1.02 | 0.98 | 0.65 | 1 | 3.96 | 1.76 | 0.09 | 0.3 | 0.85 | 0.82 | 0.39 | NRCAM |
| gene14745 | 1.05286221 | 0.001112929 | 68.75 | 74 | 69.99 | 65.34 | 60.78 | 47.91 | 64.52 | 125.47 | 100.3 | 93.35 | 139.25 | 115.11 | LOC100623945 |
| gene14749 | -1.323542416 | 8.98E-05 | 25.53 | 22.37 | 20.35 | 11.93 | 10.06 | 70.55 | 29.68 | 13.44 | 16.3 | 22.33 | 6.71 | 6.63 | LOC100521143 |
| gene14751 | -2.204540068 | 7.51E-11 | 3.17 | 15.18 | 11.35 | 32.15 | 6.72 | 19.47 | 2.73 | 14.69 | 3.59 | 4.39 | 5.44 | 2.31 | AMPH |
| gene14752 | -1.82781612 | 2.47E-07 |  |  |  |  |  |  |  |  |  |  |  |  |  |
| gene14821 | -1.305746095 | 0.009435547 | 1.14 | 0.61 | 0.51 | 0.7 | 0.72 | 0.8 | 0.55 | 0.14 | 0.81 | 0.31 | 0.21 | 0.38 | LOC100625292 |
| gene14830 | -2.49762097 | 1.58E-13 | 4.26 | 13.93 | 8.27 | 13.64 | 33.14 | 17.04 | 12.93 | 0.97 | 3.45 | 4.32 | 2.71 | 4.1 | TNN |
| gene14842 | 1.259083805 | 0.000402254 | 0.97 | 1.68 | 0.84 | 0.81 | 1.22 | 0.97 | 1.86 | 0.28 | 1.48 | 3.22 | 1.36 | 2.42 | LOC100522028 |
| gene14849 | 1.676341748 | 8.87E-07 | 1.78 | 2.35 | 2.6 | 1.62 | 1.41 | 1.69 | 3.21 | 2.7 | 5.46 | 5.32 | 2.6 | 6.73 | LOC100737809 |
| gene14851 | 1.9561321 | 3.29E-08 | 2.91 | 4.7 | 6.47 | 2.21 | 2.3 | 2.82 | 4.33 | 5.08 | 11.59 | 8.41 | 7.89 | 10.67 | RALGPS2 |
| gene14858 | 1.153604468 | 0.002168858 | 0.1 | 0.79 | 0.42 | 0.76 | 1.36 | 1.76 | 0.88 | 1.39 | 0.92 | 7.58 | 0.52 | 0.43 | LOC100523986 |
| gene14880 | 2.76910994 | 2.54E-07 | 0.67 | 0.1 | 0 | 1.78 | 0.57 | 0 | 0 | 0.36 | 0 | 16.12 | 0 | 0.48 | LOC100512122 |
| gene149 | -1.282418038 | 0.00031812 | 5.59 | 3.72 | 3.1 | 3.8 | 3.94 | 4.72 | 3.28 | 0.93 | 2.72 | 1.48 | 1.56 | 1.91 | LOC100155054 |
| gene1490 | 1.298419486 | 0.009674716 | 0.23 | 0.6 | 0.29 | 0.1 | 0.15 | 0.17 | 0.05 | 0.4 | 0.26 | 0.7 | 0.21 | 0.17 | LOC100739045 |
| gene14921 | 1.274727459 | 7.53E-05 | 187.21 | 338.97 | 253.29 | 85.49 | 54.84 | 58.97 | 58.29 | 181.77 | 145.75 | 216.51 | 131.18 | 122.54 | PGHS-2 |
| gene14946 | -1.530668334 | 7.48E-05 | 5.15 | 4.03 | 4.52 | 9.2 | 10.32 | 7.44 | 5.8 | 3.24 | 3.6 | 3.35 | 2.11 | 3.54 | LOC100524332 |
| gene14958 | -1.039148267 | 0.004051043 | 1.7 | 3.9 | 0.76 | 0.97 | 3.56 | 5.11 | 2.98 | 1.02 | 4.5 | 1.71 | 1.83 | 0.97 | NEK2 |
| gene14990 | -4.169210095 | 6.60E-24 | 1.46 | 3.33 | 4.05 | 2.76 | 4.75 | 27.59 | 4.12 | 0.41 | 0.2 | 0.32 | 1.03 | 0.55 | LOC100515158 |
| gene14991 | -3.543279161 | 2.70E-08 | 0.5 | 0.81 | 0.43 | 0.8 | 0.95 | 1.25 | 0.75 | 0.79 | 0.19 | 0.06 | 0.17 | 0 | ZPBP |
| gene1500 | 1.346614878 | 0.000232452 | 6 | 7.21 | 1.26 | 1.78 | 2.5 | 1.08 | 1.7 | 2.77 | 6.31 | 4.85 | 3.99 | 4.44 | CD274 |
| gene15024 | 1.343321565 | 3.12E-05 | 683.87 | 154.43 | 294.81 | 123.03 | 219.93 | 193.89 | 348.76 | 379.47 | 216.1 | 481.51 | 425.6 | 415.63 | RGS2 |
| gene15025 | 2.602950736 | 2.07E-13 | 9.9 | 8.19 | 14.59 | 3.01 | 3.25 | 6.76 | 5.45 | 6.79 | 12.86 | 21.36 | 15.33 | 37.68 | RGS1 |
| gene15051 | 1.233858355 | 0.008167542 |  |  |  |  |  |  |  |  |  |  |  |  |  |
| gene15064 | -1.638024454 | 1.01E-06 | 80.45 | 100.67 | 84.8 | 93.03 | 107.77 | 118.98 | 24.18 | 73.87 | 42.3 | 44.02 | 47.32 | 8.76 | LOC100517050 |
| gene15075 | -1.477475458 | 9.07E-06 | 8.07 | 19.46 | 12.98 | 25 | 69.83 | 45.69 | 143.14 | 11.94 | 8.58 | 13.62 | 20.61 | 14.76 | LOC100518506 |
| gene15085 | -1.160607205 | 0.005986377 | 0.63 | 0.95 | 0.97 | 0.9 | 0.63 | 1.04 | 0.65 | 0.64 | 0.73 | 0.33 | 0.53 | 0.24 | LOC100521858 |
| gene15110 | 2.51688676 | 1.92E-10 | 2.19 | 0.74 | 0.8 | 0.42 | 0.86 | 0.39 | 0.48 | 1.89 | 0.83 | 2.78 | 4.19 | 2.2 | LOC100522753 |
| gene1513 | 1.518656745 | 2.99E-06 | 36.88 | 85.47 | 52.61 | 23.24 | 32.9 | 16.95 | 30.12 | 29.02 | 40.31 | 78.72 | 48.29 | 78.52 | SLC1A1 |
| gene15149 | -1.563766894 | 4.17E-06 | 1.67 | 1.81 | 5.68 | 14.57 | 6.63 | 7.85 | 2.52 | 11.61 | 5.07 | 3.66 | 3.38 | 2.55 | LOC100516995 |
| gene15157 | -1.883747489 | 7.04E-06 |  |  |  |  |  |  |  |  |  |  |  |  |  |
| gene15160 | 2.2514667 | 1.38E-08 |  |  |  |  |  |  |  |  |  |  |  |  |  |
| gene15194 | -1.299676089 | 0.000420208 | 0.52 | 1.45 | 0.29 | 0.22 | 0.99 | 1.22 | 0.88 | 0.32 | 1.32 | 0.37 | 0.32 | 0.28 | KIF14 |
| gene15200 | 1.964444916 | 5.18E-05 | 0.04 | 1.04 | 0.94 | 0.72 | 0.14 | 0.3 | 1.03 | 0.45 | 1.58 | 1.5 | 0.07 | 2.58 | LOC100622450 |
| gene15205 | -2.757527287 | 3.23E-12 | 0.74 | 0.12 | 0.79 | 0.96 | 4.71 | 0.82 | 1.81 | 0.14 | 0.39 | 0.36 | 0.35 | 0.24 |  |
| gene15210 | 1.700717752 | 3.20E-06 | 6.17 | 0.36 | 1.42 | 0.67 | 0.67 | 1.26 | 0.51 | 2.28 | 0.91 | 4.6 | 2.67 | 0.82 | LOC100524949 |
| gene15217 | -1.026049134 | 0.002052072 | 12.41 | 12.79 | 18.63 | 27.62 | 16.84 | 28.75 | 12.95 | 7.68 | 9.4 | 11.96 | 6.24 | 16.44 | LOC100739704 |
| gene15232 | 1.107467205 | 0.003477567 |  |  |  |  |  |  |  |  |  |  |  |  |  |
| gene15250 | -1.223014793 | 0.000220099 | 6.79 | 8.64 | 10.47 | 11.36 | 10.28 | 17.1 | 12.21 | 4.24 | 4.75 | 7.69 | 3.13 | 5.28 | LOC100516459 |
| gene15262 | -3.243578926 | 1.72E-16 | 1.7 | 2.23 | 1.82 | 2.89 | 4.25 | 5.24 | 5.96 | 0.59 | 0.1 | 0.37 | 0.41 | 0.51 | SLC28A3 |
| gene15275 | -6.166032942 | 1.27E-16 | 0.21 | 3.03 | 2.82 | 2.55 | 3.18 | 8.95 | 2.63 | 0 | 0.65 | 0.19 | 0 | 0 | CCL21 |
| gene15290 | 1.859361485 | 4.88E-07 | 0.22 | 0.42 | 0.18 | 0.36 | 0.34 | 0.39 | 1.06 | 1.96 | 1.33 | 1.31 | 1.49 | 1.02 | KIF24 |
| gene15313 | -1.237707205 | 0.000186699 | 3.85 | 3.25 | 2.26 | 9.03 | 4.05 | 7.59 | 3.33 | 2.46 | 2.72 | 4.16 | 1.48 | 2.91 | DDX58 |
| gene15318 | 1.551749298 | 0.000316429 |  |  |  |  |  |  |  |  |  |  |  |  |  |
| gene15325 | -1.034453236 | 0.004908966 |  |  |  |  |  |  |  |  |  |  |  |  |  |
| gene15340 | -1.546328837 | 3.32E-06 | 28.81 | 65.39 | 78.45 | 169.24 | 50.24 | 82.03 | 31.23 | 79.98 | 57.84 | 26.7 | 41.87 | 30.27 | LOC100517417 |
| gene15402 | -1.41726934 | 0.002438944 |  |  |  |  |  |  |  |  |  |  |  |  |  |
| gene15407 | -1.220999767 | 0.001053792 | 0.66 | 1.2 | 0.42 | 0.72 | 1.18 | 2.2 | 1.08 | 0.65 | 1.81 | 0.56 | 0.51 | 0.62 | MASTL |
| gene15414 | -1.227345825 | 0.002771855 | 0.06 | 0.79 | 0.23 | 0.12 | 0.92 | 0.14 | 0.22 | 1.85 | 0.35 | 0.14 | 0.21 | 0.15 | MYO3A |
| gene15416 | -1.521983288 | 2.48E-05 |  |  |  |  |  |  |  |  |  |  |  |  |  |
| gene15424 | 2.809300227 | 7.98E-07 | 5.52 | 2.23 | 1.69 | 0.17 | 1.01 | 0.26 | 5.25 | 3.02 | 4.86 | 2.81 | 5.34 | 1.15 | LOC100519970 |
| gene15432 | -4.421430976 | 7.98E-12 | 2.42 | 10.85 | 3.34 | 14.18 | 3.53 | 6.52 | 1.35 | 2.48 | 2.13 | 0.69 | 0.33 | 0 | LOC100520629 |
| gene15480 | 2.242546568 | 1.36E-11 | 96.94 | 93.89 | 63.33 | 55.13 | 48.9 | 40.8 | 68.58 | 124.3 | 187.3 | 108.9 | 195.69 | 352.03 | LOC100516289 |
| gene15493 | -2.874956077 | 7.42E-15 | 14.56 | 14.89 | 17.17 | 24.41 | 30.34 | 11.32 | 4.53 | 1.44 | 2.66 | 1.73 | 1.92 | 5.01 | LOC100737810 |
| gene15495 | -6.630471079 | 4.06E-09 | 0.09 | 0.04 | 0.33 | 0.24 | 0.96 | 0.04 | 0.16 | 0 | 0.04 | 0 | 0 | 0 | LOC100517730 |
| gene15500 | 5.151711691 | 1.64E-40 | 3.47 | 19.82 | 2.29 | 2.12 | 3.28 | 2.74 | 6.86 | 109.9 | 11.17 | 162.25 | 114.77 | 6.52 | LOC733634 |
| gene15501 | -4.992181245 | 0.000978692 |  |  |  |  |  |  |  |  |  |  |  |  |  |
| gene15515 | 1.584442897 | 8.42E-06 |  |  |  |  |  |  |  |  |  |  |  |  |  |
| gene15519 | 1.094487306 | 0.000675306 | 36.25 | 58.88 | 49.28 | 20.23 | 28.25 | 27.01 | 36.25 | 33.02 | 44.27 | 43.93 | 50.84 | 60.53 | PFKP |
| gene15544 | 1.102618684 | 0.001707184 | 15.54 | 23.06 | 27.56 | 15.62 | 10.72 | 17.66 | 26.87 | 28.87 | 27.32 | 31.8 | 28.77 | 30.13 | LOC100525248 |
| gene15546 | 1.070272152 | 0.001641231 | 17.1 | 8.43 | 8.71 | 5.81 | 6.87 | 9.44 | 10.61 | 10.35 | 11.56 | 9.94 | 19.58 | 14.62 | LOC100627052 |
| gene15582 | 5.241005801 | 0.000550739 |  |  |  |  |  |  |  |  |  |  |  |  |  |
| gene15619 | -1.237593033 | 0.000161605 | 4.73 | 17.72 | 28.37 | 30.68 | 32.43 | 27.01 | 24.56 | 4.74 | 15.13 | 7.54 | 6.8 | 22.5 | LOC100739731 |
| gene1562 | -2.116020565 | 3.01E-10 | 32.92 | 37.65 | 8.26 | 7.34 | 10.03 | 125.16 | 13.29 | 9.41 | 6.17 | 16.05 | 7.47 | 7.64 | LOC100517137 |
| gene15620 | 1.211093872 | 0.00030961 | 0.62 | 0.92 | 1.42 | 1.37 | 1.86 | 1.53 | 0.91 | 1.94 | 6.43 | 7.8 | 2.53 | 0.63 | ATP8A2 |
| gene15627 | -1.561331033 | 0.000574175 | 0.72 | 0.67 | 0.55 | 1.02 | 0.61 | 2.83 | 0.58 | 0.71 | 0.73 | 0.61 | 0.48 | 0.32 | GPR12 |
| gene15664 | 1.238560596 | 0.000120298 | 1412.4 | 407.81 | 817.21 | 305.6 | 385.18 | 209.5 | 141.01 | 295.67 | 378.4 | 1455.4 | 330.77 | 346.32 | HSPH1 |
| gene15665 | 1.451204309 | 7.06E-06 | 561.04 | 164.04 | 306.27 | 115.24 | 153.41 | 94.26 | 53.73 | 119.14 | 205.97 | 627.51 | 178.96 | 183.99 | HSPH1 |
| gene15682 | 2.030581396 | 0.005457892 | 0.15 | 0.17 | 0.1 | 0.09 | 0.06 | 0.04 | 0.25 | 0.08 | 0.13 | 0.42 | 0.35 | 0.07 | LOC100522696 |
| gene15685 | -1.186758185 | 0.003344001 | 3.24 | 2.53 | 3.04 | 4.6 | 3.81 | 4.16 | 3.37 | 4.64 | 3.35 | 3.83 | 1.16 | 0.63 | LOC100624094 |
| gene15725 | -1.561232766 | 0.00010603 | 0.76 | 1.07 | 0.84 | 0.83 | 1.71 | 2.58 | 0.81 | 0.82 | 0.26 | 0.83 | 0.62 | 0.28 | LOC100152644 |
| gene15737 | -1.352664032 | 0.001147924 | 4.02 | 5.93 | 9.26 | 11.38 | 9.44 | 9.76 | 1.95 | 4.44 | 2.48 | 2.32 | 2.19 | 7.15 | LOC100738038 |
| gene15761 | -1.635284851 | 1.45E-06 | 4.74 | 6.23 | 8.63 | 10.5 | 9.89 | 17.77 | 25.77 | 0.94 | 2.08 | 4.6 | 5.41 | 1.79 | CPB2 |
| gene15782 | 1.835069353 | 0.006141164 | 0.36 | 0.37 | 0.21 | 0.09 | 0.19 | 0.12 | 0.29 | 0.13 | 0.21 | 0.46 | 0.74 | 0.29 |  |
| gene15804 | 1.679158727 | 0.002450843 |  |  |  |  |  |  |  |  |  |  |  |  |  |
| gene15829 | 3.357664287 | 0.00611413 |  |  |  |  |  |  |  |  |  |  |  |  |  |
| gene15852 | 1.237582616 | 0.000423143 |  |  |  |  |  |  |  |  |  |  |  |  |  |
| gene15875 | 1.066502197 | 0.001125948 | 14.99 | 22.9 | 20.91 | 8.92 | 16.17 | 11.13 | 12.18 | 17.22 | 13.08 | 25.94 | 19.23 | 28.7 | LOC100157266 |
| gene15879 | 2.888381066 | 4.19E-13 | 3.24 | 5.74 | 2.58 | 6.47 | 6.72 | 3.27 | 67.72 | 61.11 | 48.17 | 52.17 | 38.84 | 31.08 | LOC100737161 |
| gene15882 | -1.669375904 | 4.32E-05 | 0.04 | 0.12 | 0.62 | 0.56 | 0.83 | 1.5 | 0.19 | 0.2 | 0.29 | 0.22 | 0.19 | 0.45 | SLITRK6 |
| gene15891 | -1.175835697 | 0.000398958 | 3.75 | 4.37 | 10.52 | 16.2 | 5.47 | 8.48 | 1.66 | 20.07 | 4.88 | 4.54 | 7.16 | 1.12 | GPC5 |
| gene15900 | 1.311423771 | 0.000132125 | 3.04 | 2.31 | 1.89 | 1.56 | 1.72 | 2.53 | 2.57 | 4.41 | 2.21 | 4.83 | 4.88 | 4.08 | LOC100152536 |
| gene15905 | -1.577738885 | 0.001113274 |  |  |  |  |  |  |  |  |  |  |  |  |  |
| gene15906 | 1.619612687 | 0.00014898 | 0.09 | 0.63 | 0.36 | 0.35 | 0.17 | 0.22 | 0.38 | 0.73 | 0.82 | 0.59 | 0.78 | 0.76 | LOC100620159 |
| gene15931 | -1.820056402 | 0.001626139 | 0.15 | 0.28 | 0.08 | 0.27 | 0.26 | 0.6 | 0.48 | 0.15 | 0.11 | 0.1 | 0.03 | 0.15 | LOC100523016 |
| gene15941 | -4.525444319 | 5.02E-10 | 0.08 | 0.09 | 0.05 | 0.33 | 0.21 | 0.32 | 0.07 | 0.04 | 0.02 | 0.02 | 0 | 0.01 | LOC100524825 |
| gene15954 | -1.014239904 | 0.00852001 | 5.87 | 5.82 | 13.31 | 11.94 | 11.86 | 9.89 | 4.65 | 13.37 | 1.86 | 5.94 | 8.15 | 2.49 | LOC100512806 |
| gene1597 | 1.014468502 | 0.002542437 | 4.93 | 4.85 | 6.83 | 3.74 | 4.86 | 5.5 | 5.13 | 6.75 | 8.7 | 6.83 | 7.38 | 13.08 | DNAJB5 |
| gene15981 | -2.560670852 | 4.60E-13 | 85.18 | 70.14 | 44.57 | 98.17 | 52.85 | 52.48 | 23.45 | 15.47 | 15.79 | 12.97 | 6.35 | 14.22 | LOC100620225 |
| gene15982 | -1.039119407 | 0.004932163 | 1.67 | 1.55 | 2.08 | 2.04 | 3.23 | 3.99 | 4.02 | 1.66 | 4.03 | 1.62 | 0.67 | 2.08 | LOC100620837 |
| gene15988 | 1.583167426 | 1.84E-05 | 2.39 | 2.38 | 1.5 | 1.94 | 1.8 | 0.82 | 3.61 | 4.11 | 5.62 | 2.47 | 4.04 | 6.84 | ATP4B |
| gene16004 | -1.730474693 | 0.003056262 | 0.12 | 0.2 | 0.06 | 0.19 | 0.34 | 0.34 | 0.24 | 0.2 | 0.13 | 0.1 | 0.02 | 0.13 |  |
| gene16012 | -2.250922065 | 5.52E-07 | 0.41 | 0.52 | 0.92 | 2.01 | 1.88 | 2.47 | 1.97 | 0.43 | 0.57 | 0 | 0.64 | 0.58 | LOC100523990 |
| gene16044 | -1.230587694 | 0.001804024 | 0.39 | 0.61 | 1.36 | 0.76 | 0.6 | 0.93 | 0.85 | 0.2 | 0.36 | 0.12 | 0.32 | 0.48 | CARD14 |
| gene16047 | -1.083486012 | 0.000906057 | 7.83 | 10.28 | 7.65 | 9.14 | 11.14 | 9.43 | 7.68 | 3.92 | 4.3 | 4.91 | 4.09 | 4.63 | LOC100526132 |
| gene1606 | -1.573861994 | 7.96E-05 | 3.95 | 4.6 | 4.1 | 2.91 | 3.55 | 3.08 | 2.52 | 1 | 1.98 | 1.91 | 0.55 | 0.68 |  |
| gene1608 | -1.260823156 | 0.001148249 | 0.25 | 0.28 | 1.96 | 0.56 | 0.18 | 1.15 | 0.14 | 0.24 | 0.14 | 0.15 | 0.22 | 0.37 | LOC100156421 |
| gene16140 | -2.260245401 | 0.003796963 | 0.09 | 0.17 | 0.11 | 0.13 | 0.35 | 0.23 | 0.1 | 0.22 | 0.12 | 0.04 | 0.03 | 0.09 | LOC100515852 |
| gene16142 | -1.215507998 | 0.002010259 | 0.96 | 1.62 | 1.98 | 4.43 | 2.26 | 4.14 | 2.87 | 2.78 | 1.49 | 1.21 | 1.76 | 1.44 | CD300C |
| gene16147 | -1.210101078 | 0.004288186 | 0.26 | 0.19 | 0.43 | 0.36 | 0.24 | 1.42 | 0.35 | 0.32 | 0.11 | 0.26 | 0.15 | 0.4 | DNAI2 |
| gene1616 | 1.064339764 | 0.008311692 | 0.75 | 0.63 | 1.45 | 0.58 | 0.92 | 0.47 | 1.54 | 1.33 | 0.7 | 1.81 | 1.27 | 1.06 | CA9 |
| gene16183 | -2.841033183 | 7.02E-09 | 1.14 | 0.89 | 3.18 | 8.74 | 1.44 | 6.03 | 4.78 | 0.66 | 0 | 1.57 | 0.33 | 0.3 | LOC100522637 |
| gene162 | 1.067386464 | 0.000909885 | 8.48 | 8.53 | 7.11 | 4.95 | 6.18 | 5.88 | 6.43 | 5.04 | 8.6 | 7.98 | 10.55 | 15.7 | HIVEP2 |
| gene16235 | -1.330661552 | 0.007684297 | 0.5 | 0.38 | 1.1 | 0.81 | 1.96 | 1.54 | 0.39 | 0.79 | 0.44 | 1.4 | 0.2 | 0.09 | LOC100516998 |
| gene16241 | 2.163777918 | 0.00264108 | 0.02 | 0.07 | 0.05 | 0.09 | 0.04 | 0 | 0.06 | 0.29 | 0 | 0.33 | 0.18 | 0.13 | LOC100738284 |
| gene16277 | -1.370625011 | 0.002459931 | 0.59 | 0.57 | 1.09 | 1.95 | 1.13 | 1.13 | 0.37 | 0.45 | 0.14 | 0.45 | 0.93 | 0.27 | FZD2 |
| gene16283 | -1.015850139 | 0.005908685 | 1.23 | 1.7 | 2.26 | 3.4 | 1.25 | 3.17 | 2.6 | 1.3 | 1.51 | 0.91 | 0.97 | 1.79 | RUNDC3A |
| gene16330 | -1.36818965 | 0.002438944 | 0.45 | 0.42 | 1.33 | 1.75 | 0.67 | 1.13 | 0.78 | 0.47 | 0.79 | 0.51 | 0.27 | 0.5 | CCR10 |
| gene16360 | -1.131950254 | 0.00117945 | 2.74 | 2.07 | 3.06 | 1.53 | 4.01 | 4.68 | 2.17 | 1.48 | 1.56 | 1.92 | 1.34 | 1.27 | LOC100736934 |
| gene16362 | -1.035633295 | 0.001704194 | 6.36 | 8.98 | 12.16 | 10.32 | 15.02 | 16.42 | 13.46 | 8.17 | 5.49 | 8.01 | 6.25 | 5.56 | LEPREL4 |
| gene16363 | -1.033196044 | 0.003059249 | 4.91 | 5.71 | 6.02 | 3.61 | 8.22 | 9.25 | 6.71 | 2.87 | 3.26 | 3.37 | 3.91 | 2.73 | FKBP10 |
| gene16364 | 1.921571721 | 5.22E-06 |  |  |  |  |  |  |  |  |  |  |  |  |  |
| gene16366 | 3.368598444 | 6.82E-05 | 0 | 0.07 | 0.04 | 0.03 | 0.03 | 0 | 0.18 | 0.15 | 0.44 | 0.36 | 0.44 | 0.04 | LOC100737030 |
| gene16421 | -1.195748162 | 0.002012921 | 0.76 | 1.15 | 1.1 | 0.49 | 1.19 | 3.26 | 1.3 | 0.66 | 1.96 | 0.88 | 0.5 | 0.73 | CDC6 |
| gene16440 | -2.58367907 | 0.000100073 | 0.25 | 0.73 | 0.33 | 0.47 | 0.8 | 1.36 | 0.56 | 0.16 | 0.09 | 0 | 0.24 | 0.14 | ZPBP2 |
| gene16454 | -1.060528894 | 0.001913333 | 1.98 | 8.36 | 6.65 | 4.91 | 5.34 | 5.23 | 5.29 | 1.68 | 6.66 | 2.96 | 1.68 | 2.63 | LOC100517731 |
| gene16470 | -1.12409448 | 0.000735935 | 49.78 | 31.56 | 23 | 30.73 | 82.77 | 30.28 | 35.24 | 15.2 | 19.13 | 19.78 | 15.12 | 30 | LOC100626612 |
| gene16485 | -1.141213374 | 0.00117945 | 1.65 | 2.92 | 3.62 | 3.6 | 5.4 | 5.5 | 3.5 | 2.23 | 3.36 | 1.69 | 2.9 | 1.74 | HOXB3 |
| gene16494 | -1.053626125 | 0.002472866 | 7.05 | 6.95 | 4.97 | 5.28 | 9.27 | 8.31 | 4.1 | 3.71 | 2.13 | 3.34 | 2.66 | 4.69 | LOC100522700 |
| gene16500 | -1.536041102 | 6.78E-06 | 71.37 | 66.75 | 41.57 | 57.49 | 67.19 | 35.03 | 13.25 | 43.92 | 5.39 | 20.06 | 29.66 | 3.97 | LOC100739319 |
| gene16507 | 1.968119871 | 5.24E-08 | 5.46 | 6.98 | 6.73 | 1.78 | 2.07 | 1.83 | 0.63 | 5.09 | 4.85 | 5.34 | 6.43 | 9.23 | LOC100621753 |
| gene16513 | -1.583933642 | 0.001306042 | 2.7 | 1.22 | 0.8 | 0.94 | 1.55 | 1.88 | 1.69 | 1.05 | 0.79 | 0.49 | 0.32 | 0.57 | LOC100736722 |
| gene16528 | -2.252769309 | 8.14E-09 | 3.21 | 2.79 | 1.15 | 5.58 | 2.74 | 3.3 | 4.91 | 0.38 | 2.26 | 1.15 | 0.67 | 0.65 | SGCA |
| gene16547 | -5.438041805 | 0.000102878 | 0 | 0 | 0 | 0 | 0.41 | 0 | 0 | 0 | 0 | 0 | 0 | 0 | LOC100737860 |
| gene16553 | 1.20442941 | 0.000187073 | 18.54 | 26.52 | 21.45 | 12.12 | 8.12 | 12.47 | 12.98 | 15.91 | 18.64 | 29.62 | 18.44 | 24.88 | ABCC3 |
| gene16557 | -2.408981395 | 0.000384103 | 0.03 | 0.1 | 0.06 | 0.21 | 0.32 | 0.15 | 0.19 | 0.05 | 0.18 | 0.07 | 0.02 | 0.02 | LOC100512012 |
| gene16670 | 2.38376376 | 0.001040859 |  |  |  |  |  |  |  |  |  |  |  |  |  |
| gene16671 | -1.128016906 | 0.005163801 | 2.19 | 3.24 | 2.13 | 3.02 | 3.52 | 1.86 | 3.49 | 0.67 | 2.53 | 2.26 | 0.84 | 0.69 | LOC100736724 |
| gene16690 | -1.103919567 | 0.009894972 | 1.65 | 1.33 | 1.31 | 3.39 | 1.58 | 1.92 | 1.34 | 1.87 | 0.97 | 1.45 | 1.12 | 0.64 | LOC100737761 |
| gene16692 | 1.37645065 | 2.93E-05 | 39.65 | 28.9 | 26 | 16.04 | 16.63 | 17.59 | 16.1 | 23.25 | 26.68 | 39.28 | 45.47 | 40.94 | LOC100623524 |
| gene16713 | -1.788425582 | 0.000362426 | 0.52 | 0.5 | 0.51 | 0.73 | 0.66 | 0.41 | 0.16 | 0.25 | 0.14 | 0.2 | 0.25 | 0.06 | SLFN11 |
| gene16734 | 1.182027527 | 0.009714081 | 2.61 | 0.9 | 0.5 | 0.43 | 0.75 | 0.63 | 1.72 | 1.14 | 0.93 | 0.86 | 1.34 | 1.75 | CDK5R1 |
| gene16739 | 1.864666947 | 3.23E-05 |  |  |  |  |  |  |  |  |  |  |  |  |  |
| gene16759 | 1.780816891 | 2.71E-06 | 33.68 | 37.83 | 13.04 | 6.56 | 3.97 | 1.16 | 1.13 | 2.6 | 6.16 | 8.05 | 4.26 | 26.28 | LOC100511472 |
| gene16760 | 1.375133616 | 2.12E-05 | 85.06 | 98.27 | 62.24 | 58.39 | 63.86 | 31.85 | 43.09 | 75.61 | 95.66 | 108.06 | 89.18 | 191.88 | LGALS9 |
| gene16763 | 1.207317558 | 0.000824332 | 12.5 | 11.56 | 7.95 | 7.97 | 11.86 | 5.94 | 10.57 | 14.71 | 14.38 | 16.17 | 14.98 | 27.11 | LOC100737244 |
| gene16846 | -1.051439499 | 0.002327389 | 4.99 | 3.99 | 4.37 | 5.45 | 5.11 | 6.61 | 3.97 | 3.74 | 3.42 | 2.92 | 2.15 | 2.98 | LOC100525077 |
| gene16865 | -1.011569065 | 0.003929545 | 1.91 | 2.57 | 1.76 | 4.1 | 1.18 | 2.71 | 3.2 | 1.17 | 2.11 | 1.4 | 1.31 | 1.13 | LOC100624795 |
| gene16917 | -1.286028436 | 0.000120649 | 6.11 | 7.14 | 3.05 | 12.92 | 13.49 | 9.86 | 13.76 | 6.89 | 2.9 | 4.43 | 6.68 | 3.34 | LOC100523750 |
| gene16932 | -2.335356646 | 8.85E-06 |  |  |  |  |  |  |  |  |  |  |  |  |  |
| gene16933 | -1.038149969 | 0.006977695 | 3.39 | 2.89 | 4.46 | 6.99 | 4.08 | 6.92 | 4.72 | 6.22 | 3.56 | 3.97 | 2.77 | 1.87 | LOC100525536 |
| gene16940 | -1.508587982 | 0.000127248 | 1.36 | 1.92 | 1.25 | 2.41 | 2.33 | 1.86 | 1.53 | 0.74 | 1.09 | 0.76 | 0.6 | 0.96 | ENO3 |
| gene1695 | 1.625053868 | 2.43E-06 | 3.54 | 1.38 | 2.43 | 2.83 | 0.85 | 1.37 | 1.93 | 2.99 | 2.36 | 6.32 | 2.41 | 6.38 | NOR-1 |
| gene16951 | -1.40461966 | 0.000901672 | 0.51 | 0.39 | 0.48 | 0.39 | 0.9 | 1.3 | 0.83 | 0.26 | 0.38 | 0.14 | 0.24 | 0.54 | ZMYND15 |
| gene16972 | 1.012504623 | 0.001892603 | 6.32 | 7.54 | 8.58 | 8.75 | 4.81 | 7.35 | 7.32 | 7.1 | 9.96 | 7.08 | 9.59 | 23.5 | LOC100620614 |
| gene16989 | 1.214338776 | 0.000188577 | 32.53 | 41.47 | 36.08 | 25.05 | 28.03 | 18.08 | 32.78 | 49.61 | 76.39 | 33.04 | 50.81 | 75.24 | LOC100622059 |
| gene17034 | 2.598417515 | 5.62E-13 | 0.15 | 1.18 | 0.65 | 1.16 | 0.4 | 0.78 | 2.05 | 3.55 | 1.56 | 1.14 | 7.04 | 5.05 | ALOXE3 |
| gene17035 | 1.012181242 | 0.00943318 | 0.68 | 1.19 | 1.52 | 2.11 | 0.66 | 0.89 | 1.38 | 3.71 | 0.43 | 1.06 | 3.34 | 2.75 | LOC100620745 |
| gene1706 | -1.500337289 | 0.000582988 | 0.93 | 2.01 | 0.86 | 1.62 | 1.86 | 1.66 | 0.68 | 0.41 | 0.61 | 0.67 | 0.24 | 0.88 | LOC100154648 |
| gene17115 | -1.956470613 | 8.71E-09 | 5.2 | 9.55 | 11.44 | 9.79 | 8.29 | 18.64 | 20.66 | 4.18 | 1.37 | 2.95 | 4.32 | 1.8 | LOC100525483 |
| gene17116 | -2.535919292 | 1.99E-12 | 3.93 | 5.09 | 5.14 | 9.05 | 2.51 | 11.45 | 1.49 | 2.47 | 0.65 | 0.49 | 3.03 | 0.25 | ALDH3A1 |
| gene17120 | -2.446330663 | 2.57E-05 | 1.22 | 0.59 | 0.86 | 0.98 | 1.66 | 2.06 | 0.78 | 0.34 | 0.45 | 0.21 | 0.2 | 0.37 | LOC100621187 |
| gene17153 | 1.532463953 | 8.54E-05 | 0.23 | 1.12 | 0.56 | 0.82 | 0.5 | 0.36 | 1.49 | 2.05 | 1.73 | 1.68 | 1.96 | 1 | LOC100736917 |
| gene17160 | 1.572456651 | 1.30E-06 | 32.13 | 18.1 | 20.37 | 41.28 | 24.79 | 22.43 | 51.34 | 83.8 | 40.6 | 38.01 | 152.74 | 58.92 | GALNTL2 |
| gene17181 | -1.011520862 | 0.001919049 | 21.5 | 11.96 | 13.82 | 37.87 | 18.92 | 17.36 | 16.52 | 33.31 | 25.88 | 13.62 | 15.07 | 6.97 | ZNF385D |
| gene17191 | -1.46329116 | 7.32E-06 | 53.97 | 76.73 | 71.2 | 98.76 | 72.4 | 136.85 | 33.13 | 43.59 | 26.24 | 50.09 | 35.11 | 22.86 | LOC100620195 |
| gene17202 | -1.22069895 | 0.001471363 | 2.49 | 2.81 | 3.65 | 3.76 | 4.29 | 4.03 | 1.64 | 3.04 | 1.62 | 2.16 | 1.68 | 1.32 | LOC100523139 |
| gene17213 | -1.113538909 | 0.006076209 | 4.38 | 4.63 | 7.71 | 9.66 | 3.49 | 5.36 | 3.75 | 8.01 | 6.5 | 5.13 | 0.8 | 2.62 | LOC100739439 |
| gene17266 | 2.094132135 | 2.95E-08 | 0.2 | 0.24 | 0.17 | 0.09 | 0.19 | 0.28 | 0.62 | 0.9 | 0.28 | 1.14 | 0.72 | 0.46 | LOC100152567 |
| gene17274 | 1.097129672 | 0.000670814 | 47.07 | 18.96 | 21.32 | 16.34 | 16.61 | 23.85 | 26.08 | 35.37 | 19.86 | 20.84 | 44.81 | 49.7 | CSRNP1 |
| gene17275 | 2.551469143 | 8.35E-10 | 0.16 | 0.21 | 0.1 | 0.12 | 0.09 | 0.1 | 0.3 | 0.18 | 0.24 | 0.23 | 0.59 | 0.84 | XIRP1 |
| gene17276 | -1.449256772 | 0.000281492 | 0.78 | 0.86 | 0.99 | 1.12 | 2.13 | 3.71 | 1.36 | 1.84 | 0.68 | 0.81 | 0.67 | 1.06 | LOC100622126 |
| gene17289 | 1.250530158 | 0.000284996 | 2.93 | 2.24 | 3.58 | 2.08 | 1.48 | 2.82 | 2.88 | 3.57 | 1.73 | 7.07 | 4.96 | 2.6 | ENTPD3 |
| gene17316 | -1.240621541 | 0.002472901 | 0.27 | 0.53 | 0.94 | 1.34 | 0.77 | 1.28 | 1.83 | 0.44 | 0.22 | 0.57 | 0.38 | 0.48 | LOC100525890 |
| gene17334 | -1.03319837 | 0.006800476 | 1.72 | 2.98 | 2.6 | 2.85 | 2.19 | 4.89 | 1.71 | 1.46 | 1.15 | 2.32 | 0.77 | 1.61 | LOC100627100 |
| gene17354 | 1.886055588 | 2.56E-05 | 0.48 | 0.69 | 0.59 | 0.44 | 0.39 | 0.3 | 0.36 | 0.77 | 0.84 | 1.67 | 0.97 | 1.56 | CCR9 |
| gene17363 | -1.346218096 | 0.004884258 | 0.25 | 0.94 | 0.54 | 1.35 | 0.62 | 1.14 | 0.58 | 1.28 | 0.44 | 0.35 | 0.57 | 0.31 | CCRL2 |
| gene17364 | -2.111964307 | 7.63E-08 | 0.78 | 1.16 | 1.69 | 0.71 | 1.92 | 3.12 | 1.4 | 0.79 | 0.35 | 0.75 | 0.45 | 0.11 | LTF |
| gene17417 | -1.842480555 | 2.32E-05 | 0.51 | 1.02 | 1.07 | 1.09 | 1.52 | 3.34 | 0.35 | 0.96 | 0.98 | 0.78 | 0.13 | 0.69 | LOC100155669 |
| gene17427 | -1.173038926 | 0.00137939 | 2.11 | 1.84 | 1.79 | 2.84 | 2.7 | 2.64 | 2.33 | 1.52 | 0.84 | 1.44 | 1.2 | 0.87 | P4HTM |
| gene17437 | -1.358063902 | 0.000173484 | 3.3 | 2.55 | 3.19 | 2.92 | 5.8 | 5.79 | 2.4 | 2.2 | 1.15 | 1.85 | 2.37 | 1.25 | LOC100510980 |
| gene17455 | -1.610838514 | 1.08E-05 | 0.44 | 0.64 | 0.93 | 2.13 | 1.28 | 1.71 | 1.22 | 0.89 | 0.53 | 0.74 | 0.33 | 0.59 | UBA7 |
| gene17457 | 1.409603745 | 3.45E-05 | 2.84 | 4.13 | 3.8 | 1.6 | 3.08 | 3.44 | 1.05 | 1.85 | 1.91 | 8.57 | 3.25 | 9.02 | CAMKV |
| gene17484 | 1.147672999 | 0.00048831 | 13.28 | 7.65 | 12.44 | 9.22 | 16.08 | 21.51 | 16.52 | 35.18 | 8.73 | 5.56 | 46.7 | 45.14 |  |
| gene1753 | 3.255086645 | 1.60E-05 | 0.76 | 0.15 | 0 | 0 | 0.87 | 0 | 3.05 | 8.21 | 1.77 | 3.7 | 4.38 | 1.81 | LOC100736986 |
| gene17545 | -1.102258885 | 0.003277504 | 1.11 | 0.63 | 2.06 | 0.96 | 2.78 | 1.81 | 1.83 | 0.4 | 0.92 | 1.14 | 0.5 | 0.96 | CHDH |
| gene17550 | -2.04640638 | 9.03E-05 | 0.6 | 1.37 | 3.41 | 1.93 | 3.1 | 2.4 | 1.46 | 0.33 | 0.32 | 0.74 | 0.84 | 0.35 | LOC100737042 |
| gene17551 | -2.709371542 | 5.72E-12 | 0.54 | 0.67 | 1.72 | 2.41 | 5.42 | 2.25 | 0.51 | 0.56 | 0.54 | 0.53 | 0.69 | 0.34 | CACNA2D3 |
| gene17573 | 1.053856907 | 0.001228137 | 25.82 | 36.58 | 35.7 | 12.65 | 14.55 | 11.97 | 8.91 | 17.99 | 18.59 | 24.26 | 23.68 | 30.7 | ABHD6 |
| gene17592 | 1.334702692 | 0.002140236 | 0.15 | 0.36 | 0.46 | 0.3 | 0.12 | 0.19 | 0.25 | 0.19 | 0.2 | 0.65 | 0.2 | 0.62 | CADPS |
| gene17596 | -1.451856725 | 0.006253371 | 0.67 | 0.88 | 0.57 | 0.83 | 1.4 | 3.77 | 0.87 | 0.89 | 0 | 0.64 | 0.85 | 0.61 | LOC100519514 |
| gene17637 | 1.622722228 | 1.45E-05 | 0.48 | 0.37 | 0.42 | 0.41 | 0.24 | 0.42 | 1.47 | 0.94 | 0.58 | 1.78 | 0.41 | 0.98 | CHL1 |
| gene17643 | 1.093050361 | 0.000697839 | 71.11 | 75.13 | 64.15 | 61.58 | 52.64 | 54.43 | 66.78 | 90.26 | 88.96 | 78.93 | 116.3 | 149.47 | TRNT1 |
| gene17646 | 1.078792723 | 0.004504072 | 0.53 | 0.42 | 0.72 | 0.52 | 0.36 | 1.25 | 0.44 | 0.61 | 0.18 | 3.03 | 0.92 | 0.38 | LOC100627698 |
| gene17653 | 1.071355787 | 0.000820614 | 246.1 | 424.8 | 364.07 | 250.04 | 364.84 | 173.89 | 405.78 | 424.29 | 371.42 | 262.59 | 487.67 | 850.22 | LOC100514873 |
| gene17664 | 2.213097904 | 2.92E-10 | 11.28 | 18.7 | 8.86 | 1.36 | 1.58 | 7.4 | 1.49 | 18.36 | 11.07 | 21.41 | 6.81 | 16.68 | LOC100621522 |
| gene17665 | 3.352976923 | 0.00611413 | 0.27 | 0.25 | 0.14 | 0 | 0.12 | 0 | 0.16 | 0.11 | 0.18 | 1.17 | 0.49 | 0.29 | CAV3 |
| gene17666 | 1.946712141 | 3.43E-09 | 93.65 | 97.12 | 94.73 | 6.11 | 5.94 | 83.1 | 9.51 | 49.59 | 116.44 | 119.96 | 75.03 | 148.34 | OXTR |
| gene17695 | 3.138113832 | 6.86E-20 | 100.9 | 148.19 | 59.71 | 120.81 | 99.84 | 95.55 | 1067.5 | 1137.4 | 754.84 | 989.42 | 956.39 | 749.17 | LOC100739306 |
| gene17744 | -1.126265359 | 0.000702086 | 4.88 | 5.11 | 7.28 | 7.11 | 8.26 | 11.68 | 5.25 | 2.5 | 1.94 | 4.69 | 4.05 | 3.25 | LOC100621722 |
| gene17746 | -1.281166466 | 0.000108676 | 7.41 | 7.14 | 13.52 | 13.38 | 15.28 | 21.3 | 8.02 | 3.54 | 6.78 | 7.33 | 5.91 | 6.61 | LOC100738595 |
| gene17775 | -1.121606536 | 0.00093954 | 1.95 | 3.65 | 2.31 | 1.82 | 3.14 | 6.35 | 2.84 | 1.17 | 3.21 | 2.18 | 1.3 | 1.54 | MCM7 |
| gene17782 | -1.058713699 | 0.009472035 | 1.9 | 1.22 | 1.75 | 2.97 | 2.56 | 2.35 | 0.99 | 2.18 | 2.4 | 2.47 | 0.4 | 0.95 | ACPP |
| gene17791 | -1.427701219 | 6.55E-05 | 0.78 | 0.96 | 2.28 | 2.36 | 1.19 | 4.89 | 1.75 | 4.3 | 0.19 | 0.51 | 1.25 | 1.22 | LOC100521929 |
| gene17799 | -1.03741406 | 0.001299123 | 50.55 | 43.97 | 20.27 | 21.75 | 33.28 | 29.84 | 17.09 | 17.41 | 8.84 | 9.38 | 9.55 | 20.95 | ICA |
| gene17812 | 1.953507707 | 0.004164925 | 0.11 | 0.12 | 0.02 | 0.04 | 0.07 | 0.02 | 0.15 | 0.18 | 0.21 | 0.13 | 0.27 | 0.16 | LOC100524585 |
| gene17852 | 1.206053916 | 0.009457508 | 0.23 | 0.13 | 0.19 | 0.18 | 0.16 | 0.21 | 0.41 | 0.56 | 0.22 | 0.49 | 0.33 | 0.4 | CLSTN2 |
| gene17875 | -1.673658407 | 3.85E-07 | 12.94 | 4.96 | 16.48 | 20.08 | 53.03 | 17.4 | 20.12 | 8.74 | 8.43 | 6.33 | 9.91 | 11.48 | LOC100626514 |
| gene17895 | -1.20485911 | 0.00020191 | 10.35 | 9.3 | 12.7 | 14.06 | 15.05 | 26.78 | 15.28 | 8.85 | 8.69 | 8.68 | 7.11 | 7.51 | HPS3 |
| gene17896 | -2.306258997 | 3.82E-12 | 118.41 | 100.53 | 225.94 | 176.28 | 141.84 | 676.56 | 264.51 | 19.78 | 30.07 | 41.27 | 121.55 | 24.19 | CP |
| gene1792 | 1.668320278 | 3.13E-05 | 0.53 | 0.46 | 0.38 | 0.32 | 0.25 | 0.48 | 0.37 | 0.63 | 1.12 | 1.86 | 0.43 | 0.89 | LOC100620295 |
| gene1794 | 1.568928689 | 2.72E-06 | 7.32 | 10.93 | 6.57 | 3.01 | 2.99 | 2.82 | 3.11 | 5.75 | 8.42 | 10.88 | 6.06 | 8.57 | SUSD1 |
| gene17941 | -1.572881649 | 0.000267308 | 2.8 | 7.36 | 3.91 | 8.55 | 5.15 | 7.09 | 1.12 | 8.81 | 2.7 | 2.97 | 2.88 | 1.2 | LOC100625460 |
| gene1795 | 1.033481206 | 0.001338517 | 24.84 | 33.08 | 28.97 | 23.82 | 17.09 | 21.5 | 28 | 32.82 | 43.17 | 37.37 | 43.54 | 41.84 | ROD1 |
| gene17951 | 1.101402136 | 0.000606796 | 75.99 | 56.29 | 60.89 | 36.98 | 36.76 | 36.85 | 46.87 | 50.84 | 64.72 | 45.78 | 94.27 | 86.63 | TIPARP |
| gene17955 | -1.056661111 | 0.001064627 | 8.3 | 11.46 | 19.23 | 31.41 | 15.97 | 28.24 | 5.07 | 15.78 | 13.59 | 13.86 | 13.43 | 7.71 | VEPH1 |
| gene17988 | 1.660627095 | 0.001135088 | 0.54 | 0.05 | 0.18 | 0.22 | 0 | 0.54 | 0.98 | 1.72 | 0.78 | 0.2 | 1.81 | 0 | OTOL1 |
| gene17991 | -2.161106966 | 3.14E-05 | 3.49 | 3.82 | 7.53 | 18.6 | 4.73 | 8.45 | 1.09 | 2.91 | 4.53 | 3.74 | 3.11 | 1.21 | BST2 |
| gene18 | 1.177071197 | 0.000413568 | 8.48 | 10.46 | 8.85 | 3.25 | 1.58 | 4.26 | 2.53 | 2.5 | 4.6 | 7.88 | 7.4 | 4.36 | LOC100512933 |
| gene1802 | 2.492392209 | 6.10E-13 | 7.32 | 27.37 | 6.98 | 2.5 | 1.74 | 5.22 | 0.9 | 12.29 | 4.39 | 16.43 | 14.02 | 19.86 | LOC100157182 |
| gene18022 | 1.059456862 | 0.001132598 | 16.03 | 19.69 | 20.92 | 16.15 | 8.85 | 13.76 | 20.59 | 15.66 | 29.11 | 22.64 | 24 | 30.69 | SKIL |
| gene18023 | 1.119418332 | 0.000494094 | 75.6 | 82.86 | 95.24 | 96.22 | 59.9 | 92.57 | 92.64 | 118.51 | 186.17 | 106.05 | 174.19 | 234.01 | SLITRK3 |
| gene1803 | -1.113545451 | 0.002381968 | 5.23 | 5.04 | 11.39 | 10.61 | 10.23 | 13.57 | 0.85 | 10.82 | 4.07 | 5.82 | 4.67 | 5.11 | LOC100516718 |
| gene18035 | -1.517404464 | 3.10E-06 | 113.01 | 49.4 | 177.84 | 121.15 | 259.27 | 132.5 | 92.44 | 61.81 | 61.91 | 80.41 | 54.4 | 42.04 | TNFSF10 |
| gene18036 | -1.783481724 | 1.20E-07 | 14.49 | 6.1 | 22.14 | 22.55 | 40.88 | 18.53 | 17.75 | 12.92 | 9 | 10.04 | 7.65 | 5.87 | GHSR |
| gene18052 | 1.171323869 | 0.005307712 | 0.75 | 0.85 | 0.57 | 1.13 | 0.92 | 1.23 | 1.16 | 5.48 | 1.87 | 4.26 | 2.02 | 0.83 | KCNMB2 |
| gene18073 | 3.408165143 | 9.25E-13 | 5.82 | 4.97 | 5.53 | 0.52 | 0 | 0.21 | 0 | 2.17 | 0.66 | 1.07 | 2.16 | 3.97 | SOX2 |
| gene18083 | 1.66947684 | 3.31E-07 | 41.77 | 16.75 | 14.16 | 13.27 | 9.75 | 14.68 | 22.76 | 43.64 | 23.84 | 26.75 | 54.46 | 32.74 | LOC100624866 |
| gene18105 | -2.133197229 | 0.00014077 | 0.11 | 0.13 | 0.04 | 0.14 | 0.47 | 0.1 | 0.08 | 0.08 | 0 | 0 | 0.05 | 0.12 | VWA5B2 |
| gene1812 | 1.874790801 | 5.65E-06 | 1.48 | 1.03 | 1.56 | 0.89 | 0.41 | 1.53 | 2.67 | 6.61 | 2.47 | 3.99 | 3.8 | 1.76 | LOC100622036 |
| gene18124 | -1.047175403 | 0.00133522 | 19.66 | 14.99 | 19.77 | 26.26 | 40.07 | 22.45 | 25.67 | 16.09 | 17.54 | 14.04 | 12.08 | 15.93 |  |
| gene18129 | 1.029515351 | 0.001504307 | 12.02 | 16.41 | 19.36 | 20.34 | 13.26 | 12.01 | 21.66 | 24.18 | 35.61 | 26.45 | 35.73 | 27.47 | LOC100737989 |
| gene18135 | -4.63651994 | 0.005776466 | 0.23 | 0.85 | 1.2 | 1.65 | 0 | 2.02 | 0.75 | 1.96 | 0.82 | 0 | 0 | 0 | LOC100516696 |
| gene18141 | 2.36305959 | 6.21E-05 | 0.02 | 0.04 | 0.03 | 0 | 0.12 | 0.22 | 0 | 0.05 | 0 | 1.63 | 0.04 | 0 | HRG |
| gene18163 | -1.311932539 | 0.001756881 | 1.69 | 1.85 | 1.94 | 2.51 | 5.12 | 0.62 | 0.9 | 3.87 | 0.56 | 0.87 | 1.82 | 0.61 | CLDN16 |
| gene1819 | 1.452751189 | 8.68E-06 | 28.83 | 30.41 | 26.5 | 21.05 | 11.89 | 14.94 | 26.62 | 22.75 | 31.3 | 14.65 | 39.96 | 69.77 | LOC100739564 |
| gene18203 | -1.442934277 | 0.00411271 | 0.23 | 0.42 | 0.37 | 0.67 | 0.61 | 0.44 | 0.24 | 0.25 | 0.3 | 0 | 0.37 | 0.25 | LOC100524712 |
| gene18209 | 1.515654504 | 7.79E-05 | 1.31 | 1.05 | 0.98 | 1.67 | 0.81 | 0.64 | 2.35 | 2.71 | 1.42 | 3.97 | 2.43 | 2.27 | LOC100512372 |
| gene18231 | -2.032575285 | 0.000594484 | 0.7 | 0.95 | 0.9 | 3.05 | 2.73 | 1.23 | 3.23 | 1.32 | 0.84 | 0.38 | 0.56 | 0.68 | LOC100738833 |
| gene18277 | 2.328929777 | 5.81E-09 | 0.25 | 0.34 | 0.38 | 0.3 | 0.39 | 0.34 | 0.79 | 0.71 | 0.69 | 1.96 | 0.71 | 2.39 | LOC100620908 |
| gene18281 | -1.467563851 | 8.55E-05 | 3.4 | 4.49 | 6.27 | 6.25 | 0.55 | 18.9 | 4.23 | 2.11 | 0.85 | 3.25 | 3.43 | 2.2 | LOC100523211 |
| gene18301 | -1.213301129 | 0.002462015 |  |  |  |  |  |  |  |  |  |  |  |  |  |
| gene18303 | -1.16167484 | 0.000764618 | 4.08 | 5.05 | 4.66 | 10.12 | 9.38 | 9.16 | 4.75 | 6.86 | 4.95 | 5.59 | 3.02 | 3.99 | LSAMP |
| gene18328 | -1.59351956 | 3.16E-05 | 0.67 | 0.99 | 1.74 | 3.41 | 2.2 | 4.94 | 1.68 | 2.98 | 1.31 | 0.97 | 1.18 | 1.25 | CD200R1 |
| gene18329 | -2.691880936 | 9.58E-07 | 0.28 | 0 | 0.67 | 2.46 | 0.32 | 0.91 | 0.61 | 0.43 | 0.22 | 0.07 | 0.07 | 0.37 | CD200R1L |
| gene18347 | -2.24229903 | 0.001064093 |  |  |  |  |  |  |  |  |  |  |  |  |  |
| gene18369 | -1.71738489 | 7.68E-07 | 6.58 | 4.95 | 7.15 | 3.99 | 8.16 | 8.82 | 6.3 | 2.54 | 2.36 | 1.55 | 2.33 | 2.22 | LOC100153011 |
| gene18371 | 1.045719902 | 0.001118405 | 52.64 | 44.79 | 44.03 | 36.46 | 29.06 | 39.6 | 34.83 | 68.54 | 82.58 | 63.47 | 79.71 | 64.91 | LOC100520981 |
| gene1838 | -1.266634787 | 8.48E-05 | 12.41 | 54.21 | 92.89 | 130.84 | 111.94 | 98.95 | 112.35 | 23.42 | 67.77 | 36.15 | 36.15 | 64.75 | TNC |
| gene18381 | -1.279453387 | 0.000109595 | 1.19 | 3.53 | 2.98 | 9 | 6.81 | 8.93 | 3.32 | 1.54 | 1.58 | 3.9 | 1.94 | 4.05 | ABI3BP |
| gene18382 | -1.368886785 | 4.62E-05 | 6.9 | 12.76 | 11.08 | 33.01 | 20.4 | 31.01 | 11.74 | 7.86 | 11.77 | 12.29 | 8.75 | 10.63 | LOC100624376 |
| gene18405 | -1.350278727 | 0.007260216 | 0.31 | 0.49 | 0.41 | 0.52 | 0.75 | 0.83 | 0.63 | 0.82 | 0.34 | 0.18 | 0.27 | 0.34 | LOC100157828 |
| gene1841 | -1.101394969 | 0.000595268 | 5.81 | 27.42 | 39.94 | 57.54 | 52.94 | 46.15 | 56.06 | 6.19 | 46.42 | 15.64 | 19.82 | 34.75 | LOC100737237 |
| gene1846 | 1.080377701 | 0.00186231 | 0.45 | 0.48 | 0.46 | 0.76 | 0.63 | 1.17 | 2.17 | 2.56 | 1.68 | 2.14 | 1.87 | 1.19 | ASTN2 |
| gene18474 | 2.419729708 | 2.70E-05 | 0.24 | 0.14 | 0.11 | 0.1 | 0.04 | 0.05 | 0.54 | 0.69 | 0.15 | 0.42 | 0.3 | 0.32 | EPHA3 |
| gene18480 | 3.004259829 | 0.000139716 | 0.39 | 0.16 | 0.26 | 0 | 0.1 | 0.06 | 0.41 | 0.36 | 0.53 | 0.7 | 0.61 | 0.12 | POU1F1 |
| gene18518 | -4.625860294 | 0.005776466 |  |  |  |  |  |  |  |  |  |  |  |  |  |
| gene18523 | 1.827906639 | 0.001229974 | 0.14 | 0.15 | 0.15 | 0.11 | 0.09 | 0.02 | 0.19 | 0.25 | 0.19 | 0.28 | 0.23 | 0.32 | LOC100737392 |
| gene18538 | 1.091979052 | 0.000746892 | 11.65 | 24.74 | 20.23 | 3.98 | 6.37 | 14.54 | 5.08 | 5.81 | 10.92 | 11.86 | 12.6 | 25.78 | ADAMTS1 |
| gene18540 | -1.649283289 | 4.83E-07 | 5.76 | 9.96 | 13.77 | 26.84 | 21.75 | 17.57 | 9.44 | 2.87 | 10.19 | 5.71 | 4.41 | 10.31 | ADAMTS5 |
| gene18553 | -1.089694292 | 0.009439399 | 0.37 | 0.45 | 0.22 | 0.52 | 0.29 | 0.9 | 0.64 | 0.75 | 0.02 | 0.37 | 0.16 | 0.24 | GRIK1 |
| gene18557 | -4.67372732 | 5.78E-11 | 0.27 | 0.37 | 1.19 | 0.65 | 3.15 | 2.8 | 0.33 | 0.14 | 0 | 0 | 0.22 | 0 | LOC100624997 |
| gene18559 | -2.989950466 | 0.00611413 |  |  |  |  |  |  |  |  |  |  |  |  |  |
| gene18586 | -1.735958636 | 0.000478611 | 1.21 | 0.15 | 1.14 | 1.48 | 1.92 | 1.24 | 2.6 | 0.78 | 1 | 0.28 | 0.36 | 0.71 | LOC100156100 |
| gene18602 | -1.255149614 | 0.003590063 | 1.84 | 0.95 | 1.42 | 4.54 | 6.91 | 1.95 | 2.98 | 0.81 | 1.53 | 1.74 | 2.18 | 1.62 | LOC100621502 |
| gene18615 | 1.097523902 | 0.000635981 | 26.35 | 30.67 | 28.22 | 24.23 | 21.31 | 27.64 | 27.42 | 47.49 | 48.29 | 25.66 | 55.19 | 68.09 | MORC3 |
| gene18622 | -1.226513585 | 0.000434838 | 4.53 | 8.51 | 4.85 | 7.04 | 17.07 | 12.48 | 7.15 | 8.17 | 10.73 | 9.42 | 3.46 | 2.81 | LOC100153272 |
| gene1863 | -1.306633248 | 6.85E-05 | 2.6 | 3.24 | 5.11 | 2.64 | 7.22 | 10.82 | 16.27 | 1.3 | 1.42 | 3.14 | 3.35 | 1.57 | C5 |
| gene18634 | 1.81641048 | 0.001506099 | 1.68 | 1.22 | 0.7 | 0.12 | 0.85 | 0.61 | 1.19 | 1.1 | 1.84 | 2.74 | 1.66 | 0.75 |  |
| gene18641 | -2.746885254 | 8.10E-16 | 77.97 | 25.55 | 70.89 | 62.18 | 84.31 | 84.83 | 31.3 | 5.21 | 10.37 | 23.29 | 5.64 | 5.4 | LOC100517006 |
| gene18643 | 2.320552292 | 0.008284963 | 0.28 | 0.02 | 0.16 | 0.1 | 0.05 | 0 | 0.03 | 0.13 | 0 | 0.4 | 0.19 | 0.23 | LOC100516832 |
| gene18662 | -1.339902503 | 0.000119074 | 6.98 | 4.62 | 25.56 | 12.93 | 12.24 | 38.01 | 28.39 | 1.2 | 1.67 | 0 | 21.89 | 1.1 | TFF2 |
| gene18663 | -3.97006077 | 3.51E-09 | 0.99 | 1.86 | 5.66 | 1.07 | 8.71 | 2.64 | 1.35 | 0 | 0 | 0 | 0.72 | 0 | TFF1 |
| gene18664 | -4.181524613 | 3.09E-18 | 0.77 | 0.07 | 1 | 1.37 | 1.74 | 6.4 | 0.45 | 0 | 0 | 0 | 0.44 | 0.04 | LOC100627826 |
| gene18667 | 1.014165054 | 0.001890517 | 10.77 | 6.75 | 13.74 | 7.49 | 8.81 | 8.4 | 10.52 | 10.08 | 17.15 | 19.17 | 9.78 | 19.59 | LOC100127144 |
| gene18669 | -1.148542531 | 0.000407979 | 20.64 | 26.07 | 24.13 | 32.47 | 33.41 | 30.17 | 15.4 | 10.74 | 17.3 | 10.94 | 11.75 | 19.07 | LOC100620809 |
| gene18672 | 1.308080826 | 5.75E-05 | 44.49 | 27.42 | 35.54 | 21.48 | 17.84 | 17.99 | 40.85 | 26.07 | 31.28 | 39.07 | 37.2 | 60.51 | LOC100621265 |
| gene18675 | -1.099906424 | 0.001026361 | 11.55 | 12.03 | 9.19 | 12.86 | 14.53 | 19.07 | 11.12 | 7.69 | 5.08 | 8.27 | 5.78 | 6.99 | LOC100623793 |
| gene18682 | -1.203996022 | 0.00678038 | 0.11 | 0.19 | 0.26 | 0.33 | 0.24 | 0.32 | 0.14 | 0.15 | 0.21 | 0.15 | 0.13 | 0.12 | LOC100622032 |
| gene1871 | 1.32214618 | 4.03E-05 | 493.55 | 571.61 | 402.55 | 219.5 | 273.31 | 260.7 | 242.96 | 302.61 | 553.54 | 498.52 | 535.28 | 782.64 | GGTA1 |
| gene1872 | 1.210518934 | 0.001689376 | 0.76 | 0.92 | 1.92 | 0.76 | 0.95 | 1.06 | 0.8 | 1.02 | 1.53 | 2.4 | 1.81 | 2.07 |  |
| gene18722 | 3.890681164 | 3.93E-15 | 0.27 | 0.2 | 0.39 | 0.06 | 0.09 | 0.23 | 0.11 | 1.24 | 0.8 | 1.45 | 2.09 | 2.01 | LOC100520406 |
| gene1873 | 2.20694269 | 0.000643336 | 0.03 | 0 | 0 | 0 | 0.3 | 0 | 0.21 | 0.17 | 0.11 | 0.63 | 0.36 | 0.51 | LOC100515993 |
| gene18731 | -1.735499619 | 2.54E-07 | 35.48 | 18.37 | 33.33 | 45.75 | 45.69 | 40.68 | 58.82 | 11.39 | 9.68 | 11.41 | 10.36 | 16.73 | GADD45G |
| gene18756 | -1.41267352 | 0.000465718 | 1.15 | 3.25 | 3.39 | 3.03 | 3.03 | 5.39 | 5.06 | 1.74 | 1.63 | 1.7 | 2.19 | 0.32 | NPM2 |
| gene18761 | 1.030943094 | 0.001932004 | 0.62 | 1.31 | 2.76 | 2.25 | 1.78 | 1.42 | 1.82 | 2.38 | 3.17 | 1.2 | 4.1 | 5.32 | HR |
| gene18776 | 2.801739747 | 1.16E-07 | 1.68 | 1.52 | 0.72 | 0.17 | 0.08 | 0.31 | 0.46 | 0.57 | 0.88 | 1.17 | 0.57 | 1.79 | EGR3 |
| gene18781 | 1.68990638 | 0.000261371 | 3.91 | 3.94 | 4.43 | 1.02 | 1.57 | 1.07 | 3.44 | 2.47 | 1.5 | 5.81 | 3.71 | 2.82 | LOC100517193 |
| gene18788 | 1.840508172 | 0.000107579 | 0.38 | 0.34 | 0.62 | 0.13 | 0.23 | 0.26 | 0.46 | 0.42 | 0.35 | 1.1 | 0.42 | 0.67 | LOC100154839 |
| gene18805 | 1.775583783 | 2.57E-06 | 0.04 | 0.19 | 0.3 | 0.61 | 0.33 | 0.43 | 0.79 | 2.44 | 2.01 | 0.24 | 2.2 | 1.88 | LOC100625407 |
| gene18826 | 1.534194675 | 0.008565373 | 0.32 | 0.78 | 0.08 | 0.27 | 0.07 | 0.14 | 1.4 | 0.63 | 0.15 | 0.56 | 0.36 | 0.45 | GULO |
| gene18838 | 1.824368015 | 5.24E-08 | 4.68 | 7.07 | 6.8 | 6.38 | 10.14 | 6.9 | 39.85 | 36.01 | 25.42 | 33.45 | 25.71 | 21.88 | LOC100154295 |
| gene18868 | -2.24309847 | 0.003796963 | 0.03 | 0.13 | 0.25 | 0.2 | 0.2 | 0.15 | 0.17 | 0.07 | 0.03 | 0.03 | 0.08 | 0 | ADAM29 |
| gene18871 | -1.367992151 | 2.23E-05 | 683.87 | 323.31 | 497.17 | 516.98 | 1684.6 | 951.09 | 731.44 | 244.9 | 454.48 | 362.81 | 340.74 | 488.3 | HPGD |
| gene18892 | -1.075880679 | 0.001059469 | 26.16 | 20.83 | 23.46 | 27.62 | 34.83 | 31.09 | 23.65 | 14.89 | 14.11 | 15.97 | 12.54 | 14.73 | LOC100153608 |
| gene18897 | -1.890888248 | 9.87E-06 | 1.11 | 0.07 | 0.36 | 0.32 | 5.28 | 0.34 | 1.06 | 0.2 | 0.41 | 0.25 | 0.12 | 1.19 | LOC100627945 |
| gene18910 | -1.981250971 | 6.44E-06 | 2.78 | 2.16 | 0.87 | 1.33 | 3.08 | 1.42 | 1.1 | 0.04 | 0.26 | 0.77 | 0.23 | 0.49 | LOC100736581 |
| gene18919 | 2.82015575 | 5.27E-06 | 0.34 | 0.06 | 0.41 | 0.1 | 0.02 | 0.06 | 0.17 | 0.2 | 0.07 | 0.28 | 0.3 | 0.73 | MMP17 |
| gene18938 | 1.334334673 | 0.006253371 | 0.18 | 0.14 | 0.16 | 0.24 | 0.05 | 0.03 | 0.12 | 0.6 | 0.1 | 0.31 | 0.36 | 0.19 | TMEM132B |
| gene18942 | 1.073579188 | 0.0008003 | 426.61 | 210.89 | 173.2 | 194.81 | 297.06 | 156.52 | 312.69 | 265.43 | 408.86 | 575.8 | 408.32 | 356.94 | UBC |
| gene18975 | -3.210196139 | 0.002185737 | 0.22 | 0.11 | 0.53 | 0.09 | 0.04 | 0.66 | 0.45 | 0.12 | 0.29 | 0.06 | 0 | 0 | GPR81 |
| gene18994 | -2.269754653 | 4.32E-07 | 1.52 | 0.97 | 0.62 | 2.5 | 0.54 | 3.04 | 1.18 | 0.34 | 0.06 | 0.59 | 0.49 | 0.15 | HPD |
| gene19026 | -1.203264265 | 0.001833685 | 0.43 | 0.26 | 0.34 | 0.34 | 0.62 | 0.59 | 0.41 | 0.38 | 0.4 | 0.29 | 0.14 | 0.21 | LOC100153673 |
| gene19041 | 2.343242558 | 5.04E-05 | 0.19 | 0.87 | 0.17 | 0.4 | 0.22 | 0.09 | 0.58 | 1.02 | 1.58 | 0.84 | 1.12 | 1.67 | LOC100156825 |
| gene19049 | 1.938057283 | 5.55E-07 | 0.12 | 0.33 | 0.41 | 0.07 | 0.16 | 0.59 | 0.2 | 0.3 | 0.47 | 0.7 | 0.34 | 1.84 | NOS1 |
| gene19064 | -2.274874391 | 9.66E-10 | 3.34 | 4.15 | 6.37 | 5.05 | 9.63 | 5.94 | 4.31 | 0.71 | 1.45 | 1.04 | 1.78 | 1.4 | LOC100154365 |
| gene19072 | -1.094560623 | 0.000742561 | 6.82 | 11.93 | 16.63 | 42.91 | 6.64 | 25.06 | 11.11 | 11.38 | 7.91 | 18.31 | 8.71 | 6.96 | OAS2 |
| gene19113 | -1.111018921 | 0.009228251 | 0.78 | 1.04 | 0.73 | 0.52 | 0.73 | 1.44 | 0.56 | 0.71 | 0.28 | 0.52 | 0.43 | 0.23 | HNF1A |
| gene19115 | -1.437855491 | 0.002827583 | 0.18 | 0.33 | 0.1 | 0.13 | 0.33 | 2.72 | 0.23 | 0.45 | 0.32 | 0.41 | 0.17 | 0.51 | OASL |
| gene19133 | -1.118293484 | 0.001294394 | 4.99 | 5.48 | 6.44 | 6.8 | 9.73 | 8.03 | 7.01 | 5.86 | 3.46 | 5.06 | 2.97 | 3.13 | LOC100520333 |
| gene19136 | 1.353042317 | 0.007828157 | 0.58 | 1.26 | 0.53 | 0.43 | 0.29 | 0.16 | 0.53 | 0.91 | 0.57 | 1.35 | 0.68 | 0.36 | DAO1 |
| gene19175 | -1.280017888 | 0.000641714 | 1.71 | 2.59 | 2.21 | 2.53 | 3.68 | 2.68 | 2.4 | 1.09 | 1.07 | 1.22 | 1.03 | 1.38 | LOC100158098 |
| gene19191 | 2.338756186 | 1.36E-11 | 5.84 | 10.67 | 23.8 | 4.09 | 3.02 | 6 | 1.75 | 50.52 | 13.96 | 24.48 | 18.07 | 20.79 | LOC100154446 |
| gene19209 | 1.06219951 | 0.001344131 | 2.77 | 2.82 | 3.23 | 2.29 | 2.2 | 1.77 | 3.86 | 3.84 | 4.92 | 4.06 | 3.3 | 5.34 | LOC100515970 |
| gene19221 | -1.014108125 | 0.003844209 | 2.89 | 2.74 | 2.32 | 3.27 | 3.68 | 4.19 | 3.33 | 2.18 | 2.1 | 1.26 | 2.01 | 2.03 | PATZ1 |
| gene19243 | -4.490120468 | 6.90E-10 | 2.81 | 5.15 | 2.44 | 5.45 | 1.99 | 1.4 | 1.82 | 0.32 | 0.19 | 0 | 0.34 | 0 | LOC100152327 |
| gene19244 | -1.976264636 | 0.000506025 | 0.64 | 0.93 | 0.59 | 1.09 | 1.4 | 0.78 | 0.99 | 1.29 | 0.69 | 0.4 | 0.31 | 0.28 | LOC100738484 |
| gene19263 | -1.771356001 | 6.56E-05 | 1 | 0.87 | 1.81 | 1.48 | 2.71 | 3.18 | 0.55 | 1.92 | 0.83 | 0.6 | 0.74 | 0.68 | GSTT1 |
| gene19266 | -1.060057807 | 0.003377952 | 1.96 | 1.31 | 1.65 | 2.06 | 3.96 | 2.76 | 2 | 1.42 | 1.08 | 2.16 | 0.77 | 1.24 | SLC2A11 |
| gene19286 | -2.603803571 | 4.17E-09 | 3.04 | 3.1 | 1.19 | 4.03 | 2.25 | 2.31 | 0.66 | 1.13 | 0.68 | 0.38 | 0.8 | 0.17 | YDJC |
| gene19290 | -1.251696569 | 0.002210646 | 1.35 | 1.29 | 0.99 | 1.8 | 3.16 | 1.16 | 1.23 | 1.44 | 0.76 | 0.71 | 0.54 | 1.35 | LOC100152324 |
| gene19324 | -2.042682929 | 1.72E-07 | 1.72 | 2.39 | 2.1 | 2.76 | 7.33 | 7.37 | 3.67 | 1.62 | 1.19 | 1.14 | 0.74 | 2.17 | CLDN5 |
| gene19363 | 1.510039709 | 7.25E-05 |  |  |  |  |  |  |  |  |  |  |  |  |  |
| gene19375 | 1.52529765 | 2.98E-06 | 59.09 | 103.36 | 134.48 | 89.67 | 84.6 | 91.22 | 106.09 | 160.85 | 124.39 | 243.34 | 246.47 | 247.38 | LOC100736612 |
| gene19391 | -3.119121124 | 3.42E-05 | 0.03 | 0.11 | 0.24 | 0.17 | 0.46 | 0.07 | 0 | 0.08 | 0.06 | 0.05 | 0 | 0.02 | LOC100153049 |
| gene19418 | -1.573783195 | 5.47E-06 | 4 | 2.58 | 5.19 | 6.36 | 10.16 | 3.01 | 6.17 | 2.19 | 2.35 | 1.87 | 2.05 | 2.53 | RASGEF1A |
| gene19422 | -1.237887236 | 0.000899181 | 4.81 | 2.53 | 3.96 | 3.62 | 4.52 | 4.28 | 2.33 | 1.54 | 1.46 | 2.58 | 1.41 | 1.18 |  |
| gene19425 | 2.350158292 | 6.65E-12 | 11.62 | 19.67 | 7.92 | 2.9 | 6.61 | 5.87 | 4.03 | 20 | 15.93 | 39.01 | 16.12 | 21.68 | PHYHIPL |
| gene19426 | 1.348267101 | 3.34E-05 | 17.45 | 24.46 | 16.12 | 9.81 | 16.97 | 13.39 | 11.53 | 33.21 | 20.57 | 41.08 | 24.48 | 34.43 | LOC100525364 |
| gene19438 | 1.371754818 | 6.39E-05 | 4.4 | 4.38 | 2.81 | 2.35 | 1.82 | 2.81 | 5.73 | 3.67 | 5.79 | 3.98 | 4.82 | 8.4 | LOC100157770 |
| gene19440 | -2.067609255 | 0.001691652 | 0.03 | 0.02 | 0.37 | 0.65 | 0.54 | 0.09 | 0.15 | 0.45 | 0.33 | 0.1 | 0.05 | 0.17 | ZNF365 |
| gene19442 | -1.005193137 | 0.00616892 | 4.14 | 3.37 | 4.51 | 4.28 | 3.58 | 5.34 | 5.06 | 0.88 | 4.6 | 2.65 | 1.25 | 2.51 | LOC100153304 |
| gene19443 | 2.599837463 | 4.94E-09 | 1.58 | 0.79 | 0.75 | 0.31 | 0.28 | 0.19 | 0.93 | 0.48 | 0.98 | 0.51 | 0.68 | 3.32 | EGR2 |
| gene19479 | 1.1269145 | 0.000693931 | 3 | 2.48 | 2.52 | 1.7 | 3.08 | 2.43 | 2.23 | 3.49 | 3.02 | 4.76 | 2.9 | 7.66 | HKDC1 |
| gene19494 | -1.737966093 | 3.47E-06 |  |  |  |  |  |  |  |  |  |  |  |  |  |
| gene19545 | 1.976937796 | 2.74E-07 | 1.72 | 2.46 | 1.65 | 1.33 | 1.37 | 0.9 | 2.89 | 1.35 | 3.92 | 1.47 | 3.9 | 8.01 | DUSP13 |
| gene19549 | -5.19039413 | 0.000550739 | 0.11 | 0.42 | 0.92 | 0.67 | 0.93 | 0.5 | 0.83 | 0.6 | 0.64 | 0 | 0.1 | 0 | LOC100521521 |
| gene19554 | 1.564877391 | 0.00100097 | 1.42 | 2.46 | 2.67 | 1.46 | 1.82 | 0 | 1.71 | 7.49 | 1.44 | 4.04 | 3.12 | 2.88 | KCNMA1 |
| gene19568 | -1.499588644 | 0.000318043 | 0.48 | 0.57 | 0.34 | 1.19 | 2.58 | 1.6 | 1.13 | 0.68 | 0.56 | 0.57 | 0.65 | 0.65 | MAT1A |
| gene19569 | 1.681996467 | 0.000318323 | 1.07 | 1.4 | 0.47 | 0.59 | 1.07 | 0.41 | 0.71 | 2.99 | 1.75 | 3.72 | 0.94 | 2.23 | LOC100524960 |
| gene19573 | 1.572094576 | 0.000109157 | 0.95 | 0.78 | 0.75 | 0.7 | 0.54 | 0.87 | 1.05 | 1.11 | 1.48 | 2.85 | 1.28 | 1.97 | LOC100153283 |
| gene19576 | -1.025895242 | 0.005394376 | 2.84 | 1.16 | 0.65 | 3.76 | 2.07 | 3.02 | 0.79 | 10.19 | 4.85 | 2.63 | 0.93 | 0.7 | LOC100622354 |
| gene19591 | 1.462778283 | 0.000512559 | 0.43 | 0.17 | 0.14 | 0.27 | 0.53 | 0.59 | 0.78 | 1.87 | 0.89 | 1.57 | 1.49 | 0.66 | LOC100739538 |
| gene19595 | 2.07617099 | 0.000175311 | 0.21 | 0.58 | 0.21 | 0.37 | 0.04 | 0.05 | 0.33 | 1.3 | 0.28 | 0.88 | 0.77 | 0.37 | LOC100736629 |
| gene19598 | 1.377528533 | 0.00034013 | 0.68 | 0.88 | 0.98 | 0.37 | 0.67 | 0.51 | 0.95 | 0.61 | 0.77 | 1.69 | 0.81 | 1.49 | LDB3 |
| gene19600 | 2.577187357 | 0.000788254 | 1.12 | 0.85 | 0.73 | 0.33 | 0.41 | 0.02 | 1.11 | 1.72 | 0.98 | 0.51 | 3.25 | 1 | LOC100153113 |
| gene19603 | -1.125076959 | 0.000711874 | 9.06 | 5.37 | 4.14 | 1.96 | 9.12 | 4.69 | 1.23 | 0.84 | 1.11 | 4.89 | 0.75 | 1.69 | MMRN2 |
| gene19606 | 1.989087134 | 2.08E-08 | 0.16 | 0.37 | 1.24 | 1.08 | 0.47 | 0.68 | 1.3 | 1.65 | 1.27 | 4.22 | 2.13 | 2.25 | MMRN2 |
| gene19607 | 2.198675174 | 6.98E-06 | 0.8 | 0.18 | 1.17 | 0.77 | 0.57 | 0.91 | 0.24 | 2.47 | 0 | 5.96 | 0.78 | 3.28 | LOC100154755 |
| gene19609 | 3.220879404 | 5.29E-08 | 0 | 0.26 | 0.37 | 0.54 | 0.16 | 0.61 | 3.1 | 3.46 | 4.49 | 3.89 | 6.71 | 1.21 | LOC100155089 |
| gene19611 | -2.1654989 | 0.000156899 | 0.41 | 0.27 | 0.48 | 0.29 | 0.93 | 0.72 | 0.78 | 0.29 | 0.34 | 0.18 | 0.18 | 0.08 | GPRIN2 |
| gene19637 | 3.563777471 | 5.69E-19 | 0.42 | 0.61 | 0.15 | 0.44 | 0.37 | 0.4 | 3.52 | 6.36 | 4.94 | 3.39 | 5.63 | 4.88 |  |
| gene19645 | -1.03349992 | 0.002214089 | 12.5 | 17.4 | 24.78 | 27.03 | 23.69 | 35.58 | 12.29 | 21.54 | 15.19 | 19.45 | 11.74 | 10.01 | MSMB |
| gene19667 | 3.219400018 | 4.49E-11 | 0 | 0.02 | 0 | 0.3 | 0 | 0 | 1.01 | 1.18 | 0.58 | 1.04 | 1.28 | 0.55 |  |
| gene19676 | 2.272537613 | 0.001278726 |  |  |  |  |  |  |  |  |  |  |  |  |  |
| gene19689 | -1.718876066 | 6.40E-07 | 0.28 | 7.99 | 21.35 | 9.35 | 23.84 | 5.5 | 12.33 | 2.28 | 3.21 | 3.6 | 0.9 | 7.18 | DKK1 |
| gene19704 | -4.385871288 | 1.08E-20 | 1.77 | 1.29 | 0.13 | 8.05 | 1.09 | 4.07 | 1 | 0.46 | 0.77 | 0.41 | 0.2 | 0.05 | LOC100156277 |
| gene19714 | -1.882188617 | 2.20E-08 | 4.42 | 5.91 | 5.31 | 17.56 | 4.65 | 29.76 | 4.19 | 8.65 | 6.32 | 4.51 | 4.5 | 4.4 | IFIT3 |
| gene19717 | -2.236210032 | 5.41E-11 | 6.85 | 2.98 | 7.42 | 10.62 | 29.82 | 16.09 | 19.8 | 3 | 5.67 | 3.63 | 3.39 | 4.71 | SLC16A12 |
| gene1973 | 2.07153818 | 2.12E-05 | 0.52 | 1.32 | 1.38 | 0.81 | 0.5 | 0.62 | 0.97 | 2.09 | 0.45 | 2.67 | 2.87 | 2.48 | LOC100621926 |
| gene19752 | 1.657454278 | 5.44E-07 | 20.98 | 43.15 | 21.52 | 8.59 | 11.33 | 8.95 | 14.09 | 25.81 | 21.52 | 38.15 | 23.99 | 26.81 | O3FAR1 |
| gene19754 | -1.003693844 | 0.003353984 | 0.65 | 1.54 | 2.01 | 1.8 | 4.96 | 1.74 | 5.65 | 1.03 | 1.15 | 1.15 | 1.3 | 1.69 | LOC100512867 |
| gene19785 | -1.305605266 | 7.52E-05 | 7.56 | 6.77 | 9.21 | 14.82 | 22.46 | 17.77 | 7.82 | 8.88 | 7.8 | 7.9 | 6.61 | 7.2 | BLNK |
| gene19810 | 2.980713046 | 4.96E-17 | 13.25 | 17.49 | 21.13 | 3.76 | 2.23 | 3.97 | 1.28 | 14.17 | 4.17 | 34.32 | 13.31 | 27.99 | SFRP5 |
| gene19811 | 1.035419516 | 0.001277004 | 27.55 | 18.06 | 23.26 | 23.58 | 15.72 | 21.53 | 34.31 | 36.57 | 38.79 | 24.75 | 43.97 | 50.07 | LOC100151975 |
| gene19822 | -1.100389012 | 0.00103591 | 2.58 | 2.48 | 4.09 | 4.21 | 2.36 | 1.43 | 0.55 | 4.18 | 1.23 | 1.81 | 1.07 | 0.82 | ABCC2 |
| gene19825 | 1.277725984 | 0.001036543 | 0.33 | 1.26 | 0.68 | 0.69 | 0.24 | 1.36 | 0.04 | 0.35 | 0.54 | 3.48 | 1.01 | 0.8 | CYP2C33 |
| gene19840 | -1.280156174 | 0.008358088 |  |  |  |  |  |  |  |  |  |  |  |  |  |
| gene19850 | -1.840860368 | 0.000603592 |  |  |  |  |  |  |  |  |  |  |  |  |  |
| gene19888 | -1.492839239 | 8.69E-06 | 15.23 | 6.02 | 12.86 | 21.41 | 12.85 | 11.2 | 4.41 | 13.36 | 6.84 | 8.39 | 4.43 | 3.11 | AS3MT |
| gene1989 | -2.430917159 | 1.51E-08 |  |  |  |  |  |  |  |  |  |  |  |  |  |
| gene19894 | 1.199356874 | 0.000256788 | 42.49 | 18.29 | 24.41 | 30.34 | 22.63 | 24.72 | 42.49 | 84.99 | 28.13 | 35.28 | 95.37 | 39.22 | LOC100739626 |
| gene19897 | 1.270069248 | 7.85E-05 | 35.58 | 91.11 | 64.07 | 74.36 | 75.46 | 53.74 | 112.6 | 92.84 | 232 | 75.1 | 181.2 | 213.8 | COL17A1 |
| gene19914 | 1.474178369 | 6.26E-06 | 104.54 | 29.52 | 27.5 | 15.64 | 13.99 | 22.89 | 34.5 | 30.5 | 43.23 | 29.49 | 40.82 | 68.51 | DUSP5 |
| gene19922 | 1.731905879 | 6.52E-06 |  |  |  |  |  |  |  |  |  |  |  |  |  |
| gene19929 | 1.40806355 | 0.000103081 | 4.03 | 3.05 | 3.25 | 3.85 | 3.49 | 2.22 | 5.39 | 8.52 | 5.32 | 5.09 | 8.68 | 10.65 | LOC100157559 |
| gene19932 | -2.283000728 | 2.94E-06 | 0.18 | 0.16 | 0.88 | 1 | 0.21 | 0.88 | 0.13 | 0.06 | 0.1 | 0.24 | 0.03 | 0.13 | LOC100627133 |
| gene19940 | -1.305483473 | 0.002195538 | 1.07 | 0.73 | 1.27 | 1.3 | 1.31 | 1.31 | 0.33 | 0.48 | 0.64 | 0.83 | 0.29 | 0.45 | ADRB1 |
| gene19943 | 2.18497846 | 3.04E-06 | 0.02 | 0.09 | 0.09 | 0.19 | 0.13 | 0.17 | 0.22 | 0.15 | 1.34 | 0.11 | 0.36 | 1.5 | VWA2 |
| gene19983 | 1.411842712 | 1.23E-05 | 538.1 | 105.51 | 136.5 | 94.07 | 97.55 | 75.98 | 96.64 | 76.76 | 119.82 | 427.7 | 106.7 | 172.83 | BAG3 |
| gene19996 | 6.466810225 | 3.63E-37 | 0.17 | 0.17 | 0.22 | 0.25 | 0.13 | 0.18 | 6.82 | 9.02 | 9.7 | 9.62 | 12.74 | 21.29 | LOC100625949 |
| gene20008 | -1.582808526 | 0.00655525 | 0.15 | 0.2 | 0.22 | 0.32 | 0.27 | 0.32 | 0.24 | 0.09 | 0.11 | 0.08 | 0.1 | 0.09 | LOC100515802 |
| gene20041 | -1.852356971 | 9.23E-07 | 3.49 | 2 | 2.94 | 4.4 | 5.43 | 3.36 | 2.5 | 1.56 | 1.21 | 1.36 | 0.92 | 1.35 | LOC100153851 |
| gene20044 | -1.807912555 | 4.72E-07 | 1.71 | 1.95 | 1.83 | 5.08 | 3.17 | 1.75 | 0.67 | 2.87 | 1.75 | 0.93 | 1 | 0.87 | LOC100156286 |
| gene20046 | -1.151500074 | 0.001490023 | 1.11 | 1.71 | 2.34 | 4.26 | 2.6 | 2.25 | 1.02 | 4.28 | 2.45 | 2.27 | 0.88 | 0.91 | LOC100157876 |
| gene20064 | 1.723447249 | 7.04E-06 | 0.67 | 0.77 | 0.41 | 0.69 | 0.88 | 0.56 | 3.56 | 3.15 | 2.38 | 2.59 | 2.19 | 1.88 | LOC100737116 |
| gene20070 | -1.385157327 | 0.006010537 |  |  |  |  |  |  |  |  |  |  |  |  |  |
| gene20096 | -2.787644905 | 0.004204377 | 0.01 | 0.06 | 0.05 | 0.08 | 0.05 | 0.12 | 0.01 | 0.03 | 0.02 | 0 | 0.01 | 0.01 | LOC100156553 |
| gene201 | -3.067219884 | 0.00611413 |  |  |  |  |  |  |  |  |  |  |  |  |  |
| gene20117 | -1.300652374 | 6.51E-05 | 6.91 | 8.69 | 18.41 | 24.29 | 12.26 | 12.04 | 1.15 | 15.44 | 6.35 | 10.05 | 5.13 | 4.18 | KIF5C |
| gene20184 | 1.580047849 | 0.001267616 | 0.02 | 0.1 | 0.25 | 0.26 | 0.08 | 0.15 | 0.05 | 0.39 | 0.15 | 0.45 | 0.21 | 0.79 | DPP10 |
| gene20186 | 4.822014652 | 1.02E-13 | 0.24 | 0.06 | 0.23 | 0.12 | 0 | 0 | 0 | 0.32 | 0 | 1.8 | 1.18 | 1.12 | LOC100154782 |
| gene20189 | -1.866722025 | 6.85E-07 | 2.29 | 2.01 | 3.43 | 2.95 | 1.36 | 8.11 | 0.69 | 1.64 | 3.13 | 1.3 | 1.31 | 0.69 | LOC100516298 |
| gene20197 | -2.584893523 | 1.99E-12 | 3.2 | 3.2 | 5.66 | 5.66 | 5.99 | 10.21 | 9.57 | 0.46 | 1.68 | 1.6 | 1.04 | 0.98 | PROC |
| gene20210 | 1.116499062 | 0.007878254 | 0.38 | 0.14 | 0.17 | 0.29 | 0.21 | 0.3 | 0.45 | 0.23 | 0.37 | 0.97 | 0.29 | 0.44 | LOC100622103 |
| gene20237 | -1.269532854 | 0.000312598 | 2.51 | 2.52 | 1.97 | 2.31 | 2.67 | 4.28 | 1.94 | 1.13 | 1.54 | 1.46 | 1.04 | 1.23 | LOC100625139 |
| gene20238 | -1.590216034 | 8.97E-06 | 1.62 | 1.61 | 1.4 | 2.36 | 1.68 | 2.1 | 0.37 | 0.62 | 0.79 | 0.74 | 0.67 | 0.6 | MYOM2 |
| gene20253 | 1.019162094 | 0.002374422 | 19.82 | 13.18 | 22.47 | 30.99 | 26.43 | 24.7 | 20.57 | 26.89 | 40.46 | 28.14 | 49.67 | 81.56 | DEFB1 |
| gene20263 | -2.196782986 | 0.000297848 | 0.2 | 0.13 | 0.91 | 1.36 | 0.17 | 0.42 | 0.2 | 0.66 | 0.51 | 0.3 | 0 | 0.1 | LOC100511598 |
| gene20294 | -2.247306517 | 0.000132976 | 0.07 | 0.44 | 0 | 0.68 | 0 | 4.23 | 2.12 | 0.08 | 1.27 | 0.46 | 0.28 | 0.41 | LOC100623527 |
| gene20299 | 1.25364838 | 0.000190582 | 3.36 | 4.02 | 5.94 | 5.5 | 3.88 | 6.57 | 7.94 | 8.44 | 15.71 | 4.59 | 4.61 | 26.79 | LRP2BP |
| gene20302 | 1.107561069 | 0.00501487 | 0.61 | 0.17 | 0.54 | 0.46 | 0.29 | 0.55 | 0.23 | 1.01 | 0.64 | 0.99 | 0.6 | 1.07 | LOC100517861 |
| gene20305 | 1.079567845 | 0.000777287 | 27.05 | 20.82 | 21.42 | 13.2 | 13.44 | 21.57 | 7.54 | 27.52 | 18.92 | 31.27 | 26.03 | 40.53 | SORBS2 |
| gene20312 | -1.15731336 | 0.000313795 | 116.91 | 99.83 | 93.96 | 254.7 | 227.84 | 238.37 | 103.8 | 290.8 | 110.84 | 77.45 | 109.87 | 122.82 | LOC100049687 |
| gene20386 | -1.248962248 | 0.001657845 | 0.78 | 0.31 | 0.68 | 0.86 | 1.24 | 1.48 | 0.59 | 0.53 | 1.02 | 0.58 | 0.57 | 0.36 | OCA2 |
| gene204 | -1.209308935 | 0.000175562 | 54.69 | 18.89 | 59.36 | 55.3 | 89.97 | 66.56 | 30.66 | 45.64 | 28.9 | 36.18 | 26.08 | 27.24 | LOC100517381 |
| gene20418 | 2.491541175 | 1.20E-12 | 6.52 | 4.35 | 3.66 | 1.21 | 1.28 | 3.09 | 2.45 | 2.36 | 4.89 | 7.2 | 4.98 | 17.31 | NR4A2 |
| gene20429 | -1.325757966 | 0.004837742 | 0.25 | 0.8 | 0.21 | 0.83 | 1.41 | 0.55 | 0.13 | 0.64 | 0.28 | 0.31 | 0.5 | 0.32 | LOC100522709 |
| gene20439 | -1.192531696 | 0.000439822 | 2.42 | 1.84 | 2.38 | 2.45 | 6.26 | 1.75 | 1.77 | 0.94 | 1.55 | 1.67 | 1.22 | 1.67 | LY75 |
| gene20465 | -4.133773595 | 4.17E-06 | 0.08 | 0.1 | 0 | 0.6 | 0.78 | 1.83 | 0.62 | 0.44 | 0.52 | 0.14 | 0 | 0 | LOC100513690 |
| gene20491 | 2.39580151 | 3.51E-05 | 2.47 | 0.22 | 0.07 | 0.06 | 0.12 | 0.52 | 0.46 | 0.43 | 0.27 | 0.59 | 1.46 | 1.33 | LOC100519057 |
| gene20493 | 3.308127758 | 1.79E-19 | 6.52 | 5.67 | 3.85 | 1.4 | 2.12 | 2.66 | 4.27 | 10.18 | 14.77 | 7.27 | 13.65 | 36.63 | LOC100519223 |
| gene20501 | 1.110752814 | 0.000526319 | 489.29 | 560.4 | 572.98 | 525.6 | 332.51 | 591.61 | 456.27 | 1105.8 | 988.51 | 647.94 | 1220.7 | 1103.1 | SSB |
| gene20533 | 3.310539424 | 3.07E-21 | 7.83 | 9.64 | 3.01 | 7.02 | 6.98 | 5.99 | 47.98 | 71.55 | 54.51 | 53.54 | 64.44 | 73.15 |  |
| gene20583 | 2.210037274 | 5.83E-05 | 0.01 | 0.03 | 0.01 | 0.01 | 0.01 | 0 | 0.02 | 0.01 | 0.02 | 0.02 | 0.05 | 0.03 | LOC100620445 |
| gene20586 | 1.040904493 | 0.001151965 | 112.16 | 139.78 | 101.92 | 82.92 | 89.94 | 91.02 | 65.46 | 105.52 | 206.16 | 131.34 | 131.14 | 260.13 | SESTD1 |
| gene20606 | -1.303057961 | 0.000112545 | 0.48 | 0.43 | 0.45 | 1.07 | 0.46 | 0.32 | 0.09 | 0.98 | 0.4 | 0.35 | 0.27 | 0.11 |  |
| gene20616 | 1.02541691 | 0.001632595 | 16 | 12.3 | 13.67 | 12.76 | 9.41 | 12.42 | 15.26 | 26.81 | 23.42 | 22 | 22.38 | 23.35 | LOC100511723 |
| gene20617 | -1.011458458 | 0.001555569 | 337.11 | 339.24 | 357.68 | 524.95 | 682.31 | 682.44 | 332.23 | 217.21 | 196.72 | 400.1 | 270.13 | 243.36 | COL3A1 |
| gene20636 | -1.422647147 | 0.000409866 |  |  |  |  |  |  |  |  |  |  |  |  |  |
| gene20660 | -1.096060806 | 0.006739689 | 0.13 | 0.12 | 0.1 | 0.09 | 0.22 | 0.41 | 0.08 | 0.07 | 0.07 | 0.11 | 0.12 | 0.09 | DNAH7 |
| gene20706 | 1.060809672 | 0.001434644 | 6.77 | 7.27 | 7.97 | 5.87 | 6.18 | 5.67 | 9.61 | 8.1 | 9.22 | 10.98 | 10.28 | 14.46 | LOC100624904 |
| gene20742 | -1.072663656 | 0.002217741 | 2.8 | 1.75 | 2.07 | 3.93 | 5.29 | 4.34 | 6.32 | 2.88 | 2.38 | 2.3 | 2.32 | 1.65 |  |
| gene20745 | -1.314531806 | 0.000214238 | 2.36 | 3.21 | 2.68 | 4.9 | 8.97 | 4.08 | 3.56 | 2.07 | 1.76 | 3.42 | 2.81 | 0.92 | GPR1 |
| gene20759 | -1.160690066 | 0.000515761 | 5.48 | 4.7 | 7.92 | 3.38 | 12.77 | 6.15 | 5.1 | 2.22 | 3.71 | 3.08 | 3.21 | 3.47 | LOC100519058 |
| gene20772 | 1.366715922 | 3.38E-05 | 2.68 | 3.4 | 3.41 | 1.56 | 2.3 | 1.1 | 2.33 | 2.39 | 3.93 | 4.1 | 4.24 | 4.12 | MAP2 |
| gene20788 | 3.208066431 | 7.01E-16 | 0.24 | 0.92 | 0.15 | 0.26 | 0.08 | 0.08 | 0.87 | 0.01 | 0.35 | 1.79 | 0.39 | 1.62 | CPS1 |
| gene20821 | -1.196995091 | 0.006212279 | 0.15 | 0.53 | 0.41 | 0.95 | 1.49 | 0.19 | 6.02 | 0.23 | 0.35 | 0.57 | 0.35 | 0.24 | RUFY4 |
| gene2084 | -1.113142855 | 0.009027212 | 1.21 | 0.79 | 1 | 1.59 | 2.09 | 1.41 | 1.64 | 1 | 1.05 | 0.46 | 1.19 | 0.68 | LOC100521710 |
| gene20858 | 1.155101667 | 0.000647477 | 2.23 | 3.03 | 1.48 | 4.16 | 2.51 | 2.78 | 4.78 | 6.2 | 7.22 | 7.44 | 7.74 | 5.19 | TUBA1A |
| gene20870 | -1.452746438 | 0.006399476 | 0.09 | 0.2 | 0.1 | 0.28 | 0.62 | 0.24 | 0.37 | 0.21 | 0.2 | 0.09 | 0.15 | 0.19 | LOC100737498 |
| gene20872 | -1.677998114 | 5.17E-05 | 0.24 | 0.27 | 0.18 | 0.42 | 0.49 | 0.43 | 0.16 | 0.11 | 0.19 | 0.2 | 0.03 | 0.18 | SPEG |
| gene20887 | 4.287114845 | 1.00E-15 | 0.15 | 0.07 | 0.43 | 0.08 | 0.02 | 0.09 | 0.64 | 0.52 | 0.53 | 1.68 | 0.82 | 1.36 | LOC100625740 |
| gene20899 | -1.095813422 | 0.00066234 | 18.77 | 16.65 | 7.88 | 21.11 | 16.64 | 11.31 | 18.55 | 10.74 | 12.56 | 9.41 | 8.47 | 4.52 | DOCK10 |
| gene20916 | 2.899632596 | 1.63E-05 | 1.69 | 9.54 | 8.14 | 0.46 | 0 | 0.38 | 0.21 | 1.5 | 0.46 | 1.29 | 3.7 | 1.37 | CCL20 |
| gene20977 | -1.056078837 | 0.001947463 | 12.25 | 6.55 | 6.8 | 9.91 | 14.76 | 7.03 | 6.22 | 6.1 | 5.11 | 8.44 | 4.97 | 1.85 | SAG |
| gene20983 | -1.232910596 | 0.00319347 | 0.63 | 0.85 | 0.91 | 0.81 | 0.87 | 1.21 | 0.74 | 0.33 | 0.29 | 0.37 | 0.43 | 0.39 | LOC100512196 |
| gene21018 | 1.49045203 | 6.49E-05 | 0.2 | 0.28 | 0.25 | 0.54 | 0.35 | 0.38 | 2.14 | 2.02 | 0.57 | 2.21 | 0.72 | 0.54 | LOC100623173 |
| gene21033 | 2.494930635 | 9.08E-10 | 0.8 | 0.94 | 0.99 | 0.13 | 0.05 | 0.66 | 0.17 | 0.88 | 1.32 | 1.37 | 1.41 | 1.34 |  |
| gene21083 | 3.486684598 | 0.00364903 | 0.06 | 0.02 | 0.04 | 0 | 0.07 | 0 | 0.31 | 0.12 | 0 | 0.19 | 0.41 | 0 | LOC100513889 |
| gene21089 | 6.189543008 | 9.95E-33 | 0.08 | 0.45 | 7.96 | 0.19 | 0.19 | 0.24 | 9.56 | 15.22 | 0 | 7.87 | 13.33 | 24.55 | LOC100513317 |
| gene21095 | -2.360393258 | 7.62E-08 | 19.45 | 5.65 | 7.8 | 28.91 | 23.95 | 29.52 | 6.51 | 5.49 | 9.88 | 3.31 | 10.18 | 2.89 | OCA2 |
| gene21108 | -2.015134159 | 0.005856017 | 0 | 0.07 | 0.43 | 0.19 | 0.34 | 0.47 | 0.15 | 0.17 | 0.05 | 0.13 | 0.09 | 0 | CTNND2 |
| gene21125 | 1.248890885 | 0.000103027 | 260.78 | 390.82 | 531.78 | 321.17 | 214.89 | 307.32 | 399.05 | 646.41 | 929.4 | 396.65 | 797.52 | 712.27 | LOC100522108 |
| gene21130 | 3.428114223 | 1.82E-11 | 0.11 | 0.26 | 0.38 | 0.34 | 0.17 | 0.1 | 1.62 | 0.49 | 0.88 | 1.28 | 0.79 | 3.39 | LOC100523217 |
| gene21138 | -1.035486794 | 0.005895321 | 1.23 | 0.49 | 0.59 | 1.78 | 1.18 | 0.94 | 0.49 | 1.37 | 1.67 | 1.07 | 0.4 | 0.43 | CDH9 |
| gene21154 | -1.099537655 | 0.001375827 | 1.96 | 3.04 | 2.44 | 3.49 | 2.59 | 4.01 | 3.65 | 0.62 | 3.37 | 1.49 | 1.18 | 1.86 | ADAMTS12 |
| gene21168 | -1.156675255 | 0.000808726 | 2.01 | 2.29 | 2.52 | 2.7 | 5.46 | 3.72 | 6.99 | 1.31 | 1.54 | 0.98 | 1.99 | 2.19 | PRLR |
| gene21181 | -1.076318775 | 0.000991552 | 13.04 | 10.55 | 17.2 | 24.41 | 13.27 | 12.25 | 3.87 | 22.51 | 12.27 | 12.7 | 6.55 | 4.04 | SLC1A3 |
| gene21206 | -3.234441312 | 1.87E-20 | 98.98 | 269.75 | 248.58 | 387.66 | 604.81 | 2087.4 | 1454.4 | 11.79 | 32.85 | 182.44 | 103.87 | 30.31 | LOC100737864 |
| gene21207 | -2.723490585 | 1.91E-10 | 0.15 | 0.14 | 0.26 | 0.47 | 1.09 | 2.81 | 0.47 | 0.16 | 0.12 | 0.46 | 0.08 | 0.09 | C6 |
| gene21234 | 1.021708442 | 0.001873515 | 6.11 | 5.81 | 5.83 | 4.71 | 3.02 | 3.78 | 5.29 | 7.74 | 6.79 | 8.22 | 7.03 | 7.34 | PARP8 |
| gene21242 | 1.026344673 | 0.001361254 | 36.72 | 39.46 | 40.72 | 33.8 | 27.89 | 26.51 | 32.4 | 45.64 | 70.48 | 37.51 | 64.12 | 70.46 | ITGA2 |
| gene21244 | -1.221392843 | 0.001005802 | 2.64 | 4.47 | 18.35 | 28.12 | 7.92 | 6.81 | 5.78 | 6.33 | 11.77 | 2.44 | 4.93 | 10.23 | LOC100622982 |
| gene2126 | -1.333542153 | 0.000144921 | 3.26 | 4.42 | 5 | 4.76 | 5.31 | 7.79 | 4.86 | 2.94 | 5.43 | 3.01 | 1.46 | 2.47 | LOC100521241 |
| gene21276 | -2.958707425 | 1.67E-13 | 0.75 | 0.42 | 0.83 | 1.07 | 2.46 | 6.07 | 2.95 | 0.33 | 0.55 | 0.3 | 0.46 | 0.39 | ANKRD55 |
| gene21284 | 1.08625093 | 0.004410706 | 2.61 | 2.94 | 2.51 | 0.99 | 2.27 | 0.52 | 2.32 | 3.07 | 1.61 | 3.39 | 3.04 | 1.55 | LOC100737888 |
| gene21294 | 2.046737397 | 9.31E-06 | 8.73 | 9.4 | 5.79 | 3.02 | 1.78 | 3.35 | 4.23 | 4.13 | 4.81 | 9.58 | 10.31 | 11.91 | LOC100523871 |
| gene21296 | -5.279006782 | 0.000312409 | 0.52 | 0.55 | 0 | 1.06 | 1.04 | 0.56 | 0.31 | 0.27 | 0.45 | 0 | 0 | 0 | LOC100524224 |
| gene21308 | 1.262757029 | 0.004373299 | 0.94 | 0.84 | 1.11 | 1.34 | 0.54 | 2.06 | 4.19 | 4.38 | 1.46 | 5.19 | 2.83 | 1.01 | LOC100622319 |
| gene21313 | -1.958724103 | 1.71E-05 | 7.04 | 5.94 | 3.63 | 25.87 | 8.38 | 6.12 | 13.85 | 1.88 | 21.18 | 4.99 | 0.31 | 5.72 | LOC100739127 |
| gene21379 | 1.532792909 | 2.21E-06 | 393.5 | 290.72 | 266.6 | 193.64 | 224.92 | 161.48 | 450.28 | 576.54 | 200.94 | 380.82 | 618.72 | 616.62 | DUSP1 |
| gene21425 | 1.935315179 | 0.005856017 | 0.17 | 0.12 | 0.07 | 0.09 | 0.03 | 0.11 | 0.1 | 0.08 | 0.13 | 0.2 | 0.23 | 0.39 | GABRB2 |
| gene21433 | -1.921895139 | 2.46E-08 | 8.49 | 6.73 | 5.15 | 15.36 | 18.09 | 18.14 | 4.45 | 2.4 | 3.42 | 5 | 3.31 | 4.94 | C1QTNF2 |
| gene21435 | -1.453197143 | 0.006399476 | 1.67 | 1.17 | 1.48 | 1.49 | 2.35 | 1.03 | 0.26 | 1.08 | 0.14 | 0.52 | 0.38 | 0.8 | FABP6 |
| gene21446 | 1.344790954 | 0.000182546 |  |  |  |  |  |  |  |  |  |  |  |  |  |
| gene21463 | 3.876831618 | 5.88E-08 |  |  |  |  |  |  |  |  |  |  |  |  |  |
| gene21475 | -1.438287642 | 0.001080298 | 3.36 | 2.64 | 1.7 | 2.63 | 4.51 | 3.23 | 2.06 | 0.25 | 2.38 | 0.91 | 1.51 | 1.37 | LOC100738893 |
| gene21493 | -1.047292273 | 0.001408059 | 8.49 | 7.12 | 8.95 | 11.42 | 9.66 | 25.75 | 7.52 | 4.59 | 6.01 | 7.76 | 9.36 | 4.45 | SLC36A1 |
| gene21494 | -1.313694471 | 5.43E-05 | 15.37 | 11.72 | 21.1 | 27.51 | 53.81 | 41.58 | 40.83 | 13.1 | 15.73 | 15.78 | 15.08 | 17.19 | SLC36A2 |
| gene21495 | 1.026245479 | 0.007816616 | 2.58 | 0.95 | 1.56 | 1.16 | 1.73 | 1.78 | 0.9 | 4.38 | 1.99 | 2.83 | 5.04 | 1.12 | LOC100625371 |
| gene21515 | -1.000589609 | 0.007087585 | 4.51 | 3.47 | 9.19 | 10.4 | 3.86 | 6.3 | 3.81 | 3.51 | 3.25 | 4.43 | 3.19 | 2.57 | LOC100518586 |
| gene21520 | 2.130287424 | 0.001496317 | 1.6 | 1.96 | 0 | 0.63 | 1.85 | 2.31 | 4.29 | 13.99 | 1.87 | 6.08 | 10.1 | 4.59 |  |
| gene21531 | -2.377532339 | 0.000539044 | 0.11 | 0.23 | 0.28 | 0.16 | 0.19 | 0.15 | 0.09 | 0.06 | 0.18 | 0.02 | 0.02 | 0.05 | TERT |
| gene21553 | 3.586628585 | 0.002185737 | 0 | 0.11 | 0.14 | 0 | 0.02 | 0 | 0.07 | 0 | 0.18 | 0.03 | 0.17 | 0.15 | LOC100622160 |
| gene21556 | -1.220586959 | 0.001138133 | 15.22 | 9.09 | 12.39 | 33.24 | 15.24 | 37.63 | 15.24 | 11.66 | 11.04 | 12.2 | 13.72 | 10.3 | LOC100523337 |
| gene21561 | -1.03972131 | 0.003424763 | 2.69 | 6.1 | 3.65 | 7.93 | 6.68 | 3.01 | 8.4 | 2.85 | 5.74 | 3.76 | 1.91 | 2.82 | CLDN23 |
| gene21574 | 1.044834571 | 0.002071456 | 25.66 | 43.62 | 20.96 | 19.84 | 36.63 | 19.6 | 43.87 | 50.38 | 27.93 | 65.56 | 28.11 | 60.68 | LOC100511354 |
| gene21575 | -1.167696919 | 0.00027392 | 68.6 | 239.84 | 306.27 | 388.25 | 405.39 | 207.16 | 138.3 | 50.45 | 197.05 | 233.51 | 74.14 | 134.36 | LOC100739628 |
| gene216 | 2.91888358 | 1.49E-06 | 0.26 | 1.38 | 0.54 | 0 | 0.04 | 0.32 | 0.15 | 0.55 | 0.07 | 1.24 | 0.82 | 0.59 | LOC100157645 |
| gene21631 | 1.179078521 | 0.000254042 |  |  |  |  |  |  |  |  |  |  |  |  |  |
| gene21636 | -1.394578436 | 0.00497313 | 0.24 | 0.52 | 0.6 | 0.64 | 0.42 | 1.34 | 0.8 | 0.41 | 0.11 | 0.25 | 0.28 | 0.35 | AP3M2 |
| gene21637 | 1.061951284 | 0.000916791 | 106.4 | 171.81 | 154.22 | 139.12 | 74.12 | 113.02 | 119.11 | 284.88 | 184.51 | 106.19 | 214.32 | 326.43 | PLAT |
| gene21640 | 1.073440919 | 0.001015564 | 26.21 | 49.03 | 34.82 | 16.52 | 12.81 | 15.35 | 11.6 | 27.41 | 30.17 | 33.61 | 23.91 | 33.44 | LOC100511905 |
| gene21647 | 1.019284058 | 0.001449926 | 477.59 | 541.67 | 531.62 | 183.77 | 316.7 | 250.48 | 231.44 | 262.86 | 366.07 | 358.2 | 355.84 | 756.97 | PRNP |
| gene2166 | -1.310067286 | 0.003328246 | 0.72 | 0.62 | 0.27 | 0.82 | 0.67 | 0.72 | 1.06 | 0.15 | 0.42 | 0.36 | 0.13 | 0.37 | CEL |
| gene21665 | 1.454736152 | 0.004094153 | 0.02 | 0.07 | 0.04 | 0.08 | 0.12 | 0.07 | 0.08 | 0.59 | 0.02 | 0.16 | 0.27 | 0.24 | LOC100627135 |
| gene21667 | 1.432951992 | 9.82E-06 | 11.95 | 33.51 | 35.93 | 27.65 | 22.81 | 13.65 | 34.82 | 43.29 | 65.36 | 31.43 | 62.67 | 72.27 | FERMT1 |
| gene21670 | 1.021151958 | 0.003481516 | 2.91 | 4.44 | 3.23 | 3.91 | 1.35 | 2.07 | 3.79 | 4.79 | 5.25 | 3.51 | 4.81 | 5.88 | LOC100152318 |
| gene21677 | 3.491001946 | 6.54E-17 | 0.62 | 0.39 | 0.18 | 0.26 | 1.21 | 0.13 | 12.58 | 1.31 | 1.97 | 14.8 | 2.38 | 1.9 | LOC100154727 |
| gene21694 | -2.393326621 | 8.48E-09 | 0.81 | 0.35 | 0.54 | 0.78 | 2.55 | 1.11 | 0.52 | 0.08 | 0.32 | 0.42 | 0.12 | 0.29 | SEL1L2 |
| gene21697 | -2.000374102 | 8.95E-09 | 3.26 | 1.63 | 3.78 | 19.09 | 26.03 | 8.61 | 23.65 | 5.65 | 2.39 | 3.71 | 4.66 | 4.82 | LOC100738215 |
| gene21701 | 1.935433435 | 0.000391493 | 0.31 | 0.04 | 0.37 | 0.08 | 0.36 | 0.15 | 0.86 | 2.4 | 1.09 | 0.88 | 0.53 | 0.94 | LOC100738356 |
| gene21715 | -1.11223226 | 0.001101196 | 2.25 | 2.48 | 3.82 | 3.84 | 5.8 | 5.09 | 3.32 | 2.71 | 2.54 | 2.96 | 1.41 | 2.34 |  |
| gene21739 | -2.05418563 | 4.03E-07 | 0.42 | 1.34 | 1.76 | 2.33 | 0.73 | 4.15 | 2.06 | 0.74 | 0.38 | 0.66 | 0.77 | 0.22 | FOXA2 |
| gene21758 | -1.07800807 | 0.001550162 | 3.85 | 2.66 | 4.66 | 4.25 | 4.14 | 5.04 | 2.34 | 2.83 | 1.69 | 1.25 | 2.66 | 2.16 | LOC100154828 |
| gene21769 | 1.45593706 | 7.47E-06 | 75.26 | 36.67 | 44.59 | 15.2 | 12.55 | 49.29 | 21.84 | 56.4 | 43.03 | 24.25 | 91.67 | 80.51 | SMOX |
| gene21789 | -1.020865352 | 0.005097727 | 9.01 | 13.21 | 11.34 | 13.38 | 12.53 | 18.02 | 13.3 | 9.9 | 5.9 | 9.3 | 6.27 | 5.46 | LOC100739128 |
| gene21795 | -5.121354322 | 0.000550739 | 0.05 | 0.98 | 0 | 0.24 | 0.23 | 1.59 | 0 | 0 | 0 | 0 | 0 | 0 | OXT |
| gene21813 | 1.213361037 | 0.001041134 | 7.11 | 2.84 | 18.73 | 3.73 | 1.7 | 1.03 | 3.05 | 4.13 | 1.05 | 5.41 | 6.9 | 2.43 | LOC100739609 |
| gene2182 | -2.161976465 | 8.07E-06 | 0.25 | 0.23 | 0.22 | 0.39 | 0.54 | 0.69 | 0.94 | 0.33 | 0.1 | 0.09 | 0.11 | 0.15 | SARDH |
| gene21821 | -1.389839276 | 0.00497313 | 0.33 | 0.28 | 0.28 | 0.64 | 0.29 | 0.71 | 0.55 | 0.86 | 0.42 | 0.4 | 0.2 | 0.03 | LOC100514740 |
| gene21827 | 1.145682777 | 0.000371145 | 113.76 | 89.46 | 112.56 | 58.91 | 67.08 | 65.65 | 69.94 | 95.94 | 122.1 | 119.71 | 132.2 | 156.86 | SDCBP2 |
| gene21830 | 2.739470093 | 3.62E-05 | 0.03 | 0.13 | 0.18 | 0.06 | 0.12 | 0 | 0.11 | 0.38 | 0 | 1 | 0.26 | 0.19 | RAD21L1 |
| gene21850 | -1.723436378 | 2.07E-05 | 1.22 | 2.27 | 3.64 | 2.78 | 3.28 | 4.19 | 1.24 | 0.42 | 1.88 | 2.27 | 0.63 | 0.24 | LOC100520156 |
| gene21859 | 3.492662607 | 6.84E-23 | 143.06 | 426.65 | 136.55 | 40.79 | 4.85 | 19.83 | 6.47 | 145.6 | 105.48 | 234.58 | 185.58 | 285.06 | LOC100738325 |
| gene2187 | -1.128381741 | 0.002724963 | 1.52 | 1.44 | 2.33 | 2.12 | 2.06 | 2.04 | 0.89 | 1 | 0.9 | 0.96 | 0.76 | 1.04 | LOC100737434 |
| gene2188 | -1.066009087 | 0.000883906 | 35.21 | 38.65 | 45.44 | 37.73 | 55.22 | 56.41 | 24.06 | 11.49 | 29.51 | 25.22 | 17.85 | 26.15 | COL5A1 |
| gene21886 | -1.347218225 | 0.005927748 | 0.56 | 0.53 | 0.27 | 0.57 | 0.56 | 0.88 | 0.61 | 0.37 | 0.27 | 0.29 | 0.24 | 0.22 | LOC100624021 |
| gene2189 | -1.971661295 | 2.06E-05 | 0.16 | 0.34 | 1.45 | 1.7 | 1.22 | 1.57 | 0.25 | 0.16 | 0.27 | 0.7 | 0 | 0.39 | FCN2 |
| gene2190 | -1.953221957 | 7.50E-06 | 1.26 | 2.31 | 2.14 | 3.55 | 1.18 | 1.35 | 0.74 | 1.35 | 1.26 | 0.37 | 0.81 | 0.39 | FCN1 |
| gene21931 | 1.173321733 | 0.000835315 | 10.13 | 8.78 | 9.2 | 1.96 | 4.86 | 5.19 | 4.75 | 5.25 | 5.45 | 7.87 | 7.36 | 10.86 | LOC100152247 |
| gene2196 | -3.648945985 | 1.69E-07 | 0.42 | 0.2 | 0 | 0.38 | 0.98 | 1.51 | 0.33 | 0.13 | 0.09 | 0.15 | 0 | 0.06 | LOC100519464 |
| gene2197 | -3.225683294 | 8.64E-21 | 521.21 | 729.17 | 868.8 | 997.46 | 1328.7 | 4101.8 | 1860.2 | 76.82 | 82.44 | 71.05 | 504.43 | 59.48 | UCAL-P19 |
| gene21990 | 1.013700838 | 0.001814598 | 7.51 | 5.4 | 6.24 | 4.49 | 4.79 | 4.97 | 5.66 | 3.7 | 10.19 | 6.54 | 6.45 | 14.66 | TTI1 |
| gene21991 | 1.014993121 | 0.001632162 | 58.46 | 46.08 | 45.03 | 26.16 | 33.56 | 40.37 | 37.07 | 25.87 | 71.2 | 41.03 | 51.13 | 101.32 | RPRD1B |
| gene21995 | 1.334701219 | 3.53E-05 | 24.61 | 91.01 | 87.99 | 28.74 | 120.89 | 14.15 | 33.08 | 21.12 | 112.07 | 135.77 | 106.6 | 168.87 | LBP |
| gene22004 | -4.625860294 | 0.005776466 | 0.14 | 0.06 | 0.03 | 0.11 | 0.11 | 0.07 | 0.02 | 0.05 | 0.13 | 0 | 0 | 0 | LOC100517013 |
| gene22024 | -1.277206233 | 0.000225565 | 0.56 | 0.96 | 6.54 | 12.88 | 2.24 | 4.05 | 3.43 | 3.75 | 1.74 | 4.06 | 2.28 | 1.43 | LOC100157757 |
| gene22034 | 4.269929352 | 2.64E-05 | 0.16 | 0.37 | 0.18 | 0 | 0 | 0.04 | 0.37 | 0.08 | 0.09 | 0.39 | 0.44 | 0.11 | HNF4A |
| gene22051 | -2.014352267 | 8.65E-10 | 8660.5 | 11596 | 13852 | 12094 | 15506 | 34721 | 37698 | 1562 | 2567.6 | 3024 | 8792.3 | 2672.4 | SLPI |
| gene22052 | -1.273738257 | 0.000118776 | 27.31 | 27.39 | 37.98 | 96.78 | 22.21 | 23.29 | 43.29 | 25.49 | 10.36 | 7.9 | 31.81 | 16.22 | LOC100512873 |
| gene22053 | -1.48764494 | 0.000176502 | 0.21 | 0.79 | 0.12 | 1.87 | 1.22 | 1.62 | 8.36 | 0.16 | 0.15 | 0.49 | 0.53 | 0.59 | MATN4 |
| gene22056 | -4.633458217 | 0.005776466 | 0 | 0.11 | 0.08 | 0.28 | 0.2 | 0.42 | 0.38 | 0 | 0.1 | 0 | 0 | 0 | LOC100623174 |
| gene22059 | 1.291365814 | 0.00249021 |  |  |  |  |  |  |  |  |  |  |  |  |  |
| gene2206 | 2.027095071 | 0.007254728 |  |  |  |  |  |  |  |  |  |  |  |  |  |
| gene22068 | -1.948177823 | 0.000433916 | 0.97 | 1.25 | 0.14 | 1.11 | 1.83 | 0.55 | 0.25 | 0.59 | 0 | 0.66 | 0 | 0.29 | LOC100738201 |
| gene22073 | 2.276393722 | 0.002259848 | 0.35 | 0.32 | 0.46 | 0.41 | 0.6 | 0.25 | 1.87 | 7.25 | 1.51 | 1.69 | 3 | 0.74 | LOC100517866 |
| gene2208 | 1.615049335 | 0.001135088 | 0.55 | 0.15 | 0.27 | 0.11 | 0.14 | 0.13 | 0.08 | 0.08 | 0.08 | 0.32 | 0.26 | 0.54 | LOC100154175 |
| gene22101 | -1.975184196 | 2.42E-05 | 0.78 | 0.78 | 0.58 | 1.23 | 0.89 | 0.79 | 0.07 | 1.72 | 0.72 | 0.33 | 0.25 | 0.13 | LOC100157990 |
| gene22108 | -1.14250303 | 0.005163801 | 0.75 | 0.81 | 1.2 | 2.09 | 0.78 | 1.19 | 0.08 | 1.15 | 0.92 | 0.49 | 0.51 | 0.82 |  |
| gene22113 | -1.407012218 | 0.006329004 | 0.78 | 0.64 | 0.48 | 1.09 | 0.62 | 1.27 | 0.66 | 0.22 | 0.38 | 0.36 | 0.14 | 0.55 | LOC100739059 |
| gene22134 | -1.100106983 | 0.000632552 | 113.14 | 70.32 | 102.82 | 83.62 | 154.59 | 56.57 | 109.3 | 53.3 | 46.94 | 55.73 | 41.58 | 38.37 | LOC100739855 |
| gene22136 | 1.189291757 | 0.000219935 | 143.1 | 117.73 | 92.11 | 53.28 | 54.61 | 62.81 | 67.21 | 71.45 | 92.03 | 103.36 | 120.54 | 150.08 | PTPN1 |
| gene22144 | -1.175955326 | 0.002639605 |  |  |  |  |  |  |  |  |  |  |  |  |  |
| gene22150 | -1.136253588 | 0.000640032 | 12.56 | 4.34 | 7.08 | 20.06 | 8.39 | 7.48 | 3.36 | 12.08 | 6.35 | 5.81 | 3.57 | 6.54 | LOC100153963 |
| gene22158 | 1.99085607 | 0.000266974 | 6.38 | 3.58 | 1.21 | 1.07 | 0.75 | 0.19 | 2.39 | 0.96 | 3.19 | 3.6 | 2.47 | 2.24 | LOC100514080 |
| gene2217 | -1.001898365 | 0.004321572 | 4.46 | 5.01 | 6.76 | 5.02 | 5.05 | 6.24 | 6.03 | 2.84 | 2.54 | 2.54 | 3.3 | 2.08 | LOC100157017 |
| gene22175 | 1.589019787 | 0.001226441 |  |  |  |  |  |  |  |  |  |  |  |  |  |
| gene22181 | 3.393416679 | 9.97E-22 | 25.53 | 38.21 | 0.86 | 1.02 | 5.71 | 1.64 | 2.28 | 1.87 | 13.84 | 31.03 | 20.02 | 35.88 | PCK1 |
| gene22182 | -1.160451595 | 0.002179401 | 0.66 | 1.26 | 0.65 | 2.71 | 1.85 | 2.29 | 1.13 | 0.83 | 1.03 | 1.31 | 0.93 | 0.78 | ZBP1 |
| gene22202 | -1.568739101 | 5.81E-05 | 2.93 | 2.14 | 5.4 | 5.14 | 4.78 | 4.74 | 3.57 | 1.35 | 2.8 | 1.18 | 1.81 | 1.85 | EDN3 |
| gene22219 | -1.360507845 | 0.000946579 | 0.63 | 0.61 | 0.29 | 1.23 | 0.52 | 0.91 | 0.44 | 0.26 | 0.21 | 0.39 | 0.19 | 0.43 | LOC100524409 |
| gene22229 | -1.186069317 | 0.001417154 | 0.65 | 0.99 | 1.68 | 1.39 | 1.7 | 1.85 | 0.73 | 2.13 | 0.87 | 0.93 | 0.44 | 0.8 | LOC100627920 |
| gene22234 | 1.448038192 | 0.00011358 | 0 | 0 | 0 | 0 | 1.84 | 0.95 | 1.97 | 0.93 | 2.42 | 3.25 | 1.12 | 3.23 | LOC100512191 |
| gene22237 | 1.618940982 | 0.002588055 | 0.28 | 0.16 | 0.45 | 0.2 | 0.2 | 0.21 | 0.77 | 0.3 | 0.3 | 0.56 | 0.29 | 1.02 | VIPR2 |
| gene22273 | -4.515423832 | 1.29E-07 | 0.22 | 0.2 | 0.37 | 0.83 | 0.57 | 1.43 | 0.44 | 0.06 | 0.48 | 0 | 0 | 0.08 | LOC100624234 |
| gene22274 | -2.245532071 | 8.92E-07 | 0.57 | 0.75 | 0.94 | 1.52 | 1.28 | 2.6 | 0.93 | 0.6 | 0.5 | 0.58 | 0.23 | 0.31 | LOC100739295 |
| gene22287 | 1.099378429 | 0.003362656 | 1.78 | 0.45 | 0.68 | 0.28 | 0.8 | 0.52 | 0.48 | 0.98 | 0.95 | 0.85 | 1.29 | 1.16 | ATG9B |
| gene22289 | -1.344365048 | 0.00036663 | 3.84 | 3.09 | 6.62 | 5.08 | 6.75 | 8 | 2.64 | 6.7 | 2.54 | 2.31 | 3.2 | 2.02 | LOC100518161 |
| gene22292 | -1.166333591 | 0.000877482 | 7.71 | 7.19 | 6.06 | 9.6 | 10.12 | 7.5 | 2.87 | 5.51 | 5.55 | 3.57 | 3.73 | 4.39 | LOC100523103 |
| gene22293 | -1.114055611 | 0.001184841 | 9.16 | 7.99 | 8.18 | 8.23 | 8.04 | 7.85 | 4.11 | 8.38 | 6.05 | 4.66 | 3.34 | 2.91 | LOC100517619 |
| gene22299 | -1.064735331 | 0.001692144 | 5.16 | 8.72 | 8.83 | 8.17 | 8.16 | 14.08 | 6.38 | 6.93 | 4.91 | 3.48 | 3.98 | 6.48 | LOC100519177 |
| gene22317 | -2.102302204 | 2.39E-10 | 216.53 | 58.82 | 170.15 | 66.43 | 122.57 | 83.58 | 26.23 | 20.98 | 48.57 | 1.73 | 56.28 | 1.69 | LOC100622162 |
| gene22319 | 1.067338304 | 0.001530419 | 3.14 | 5.1 | 5.81 | 3.37 | 1.51 | 1.72 | 2.28 | 3.51 | 2.38 | 4.38 | 3.27 | 5.68 | KEL |
| gene22322 | 1.095188054 | 0.001973778 | 0.97 | 1.76 | 0.92 | 0.62 | 1.23 | 0.62 | 0.96 | 1.38 | 1.76 | 1.55 | 1.68 | 1.97 | EPHB6 |
| gene22388 | -1.001426641 | 0.001794752 | 68.34 | 85.15 | 143.97 | 340.72 | 192.56 | 321.07 | 173.72 | 80.11 | 57.41 | 198.89 | 87.88 | 128.42 | PTF-BETA |
| gene22465 | 1.575877934 | 0.000579088 | 0.22 | 0.61 | 0.63 | 0.43 | 0.54 | 0.37 | 0.53 | 0.61 | 0.71 | 1.92 | 0.69 | 1.26 | LOC100519990 |
| gene22466 | 1.514737781 | 0.002726773 | 0.38 | 0.41 | 0.34 | 0.15 | 0.73 | 0.18 | 0.68 | 0.71 | 0.59 | 1.1 | 0.3 | 1.7 | LOC100520159 |
| gene22467 | 1.782384142 | 1.84E-07 | 23.44 | 26.82 | 37.96 | 23 | 21.95 | 13.75 | 19.14 | 51.81 | 33.37 | 61.75 | 35.75 | 97.81 | HIG2 |
| gene22471 | 1.579405218 | 0.000507968 | 0.83 | 0.46 | 0.9 | 1.06 | 0.95 | 0.76 | 0.48 | 1.1 | 0.53 | 2.2 | 1.42 | 4.41 | LEP |
| gene22473 | -4.353519538 | 1.67E-26 | 15.83 | 12.9 | 9.53 | 19.59 | 10.08 | 9.75 | 3.06 | 1.16 | 1.36 | 0.18 | 0.63 | 1.02 | LOC100523579 |
| gene22518 | 1.115056344 | 0.000542094 | 10.04 | 18.6 | 18.3 | 5 | 7.84 | 7.75 | 5.4 | 13.67 | 7.8 | 15.25 | 10.75 | 17.28 | LOC100517505 |
| gene22519 | 2.2032376 | 3.59E-11 | 23.39 | 36.02 | 24.2 | 5.34 | 2.01 | 6.78 | 0.86 | 22.68 | 12.91 | 23.65 | 15.84 | 22.7 | CFTR |
| gene22596 | 2.32796236 | 1.32E-06 | 0.48 | 0.3 | 0.86 | 0.1 | 0.65 | 1.49 | 1.77 | 0.85 | 3.9 | 2.13 | 0.89 | 6.57 | LOC100511608 |
| gene22612 | -1.21272452 | 0.000433577 | 3.49 | 3.52 | 4.17 | 9.62 | 5.92 | 4.57 | 3.86 | 4.28 | 3.22 | 4.13 | 2.09 | 2.34 | LOC100513510 |
| gene22628 | -1.490795271 | 0.002347718 | 0.74 | 1.19 | 0.81 | 2.26 | 2.59 | 0.88 | 1.6 | 0.42 | 1.07 | 0.66 | 0.53 | 0.77 | HOXA13 |
| gene22630 | -1.092018915 | 0.002462522 | 7.67 | 12.11 | 9.46 | 10.87 | 15.26 | 21.45 | 22.52 | 9.12 | 9.5 | 6.68 | 6.67 | 8.36 | LOC100620451 |
| gene22663 | -3.114634383 | 1.89E-08 | 0.75 | 0.69 | 1.47 | 1.96 | 1.12 | 2.07 | 3.55 | 0 | 0.14 | 0 | 0.08 | 0.43 | PGAM2 |
| gene22671 | 2.295340476 | 1.11E-11 | 63.94 | 8.5 | 18.75 | 12.92 | 7.02 | 13.96 | 13.86 | 80.73 | 25.96 | 8.39 | 107.14 | 38.96 | IGFBP1 |
| gene22673 | 1.448180596 | 0.000817148 | 3.15 | 0.23 | 0.22 | 0.1 | 0.18 | 0.36 | 0.31 | 0.66 | 0.61 | 0.96 | 0.58 | 0.05 | LOC100624934 |
| gene22691 | 2.413479811 | 9.21E-08 | 0.13 | 0.09 | 0.09 | 0.09 | 0.1 | 0.07 | 0.39 | 0.66 | 0.22 | 0.71 | 0.43 | 0.18 | NPC1L1 |
| gene22700 | -2.750847415 | 0.000103929 |  |  |  |  |  |  |  |  |  |  |  |  |  |
| gene22702 | -1.049919777 | 0.00277955 | 11.59 | 12.38 | 13.47 | 11.91 | 10.76 | 12.67 | 13.97 | 5.82 | 10.98 | 7.59 | 4.06 | 5.07 | LOC100516709 |
| gene22712 | -1.777991277 | 6.61E-05 | 1.77 | 1.04 | 3.06 | 2.59 | 1.2 | 5.35 | 1.58 | 1.67 | 0.54 | 1.59 | 0.87 | 0.18 | LOC100621301 |
| gene22714 | 1.236585404 | 0.000146678 | 26.98 | 19.13 | 20.55 | 10.85 | 11.24 | 16.75 | 18.02 | 17.24 | 68.82 | 12.34 | 31.05 | 43.22 | LOC100517068 |
| gene22737 | -1.132049781 | 0.001068651 | 2.43 | 1.51 | 2.12 | 4.39 | 6.25 | 2.5 | 5.19 | 2.07 | 2.5 | 2.6 | 1.75 | 1.62 | STS |
| gene22738 | -1.328353855 | 0.001025327 | 2.51 | 1.25 | 1.97 | 2.74 | 3.74 | 2.87 | 2.82 | 1.96 | 1.96 | 2.15 | 0.76 | 0.9 | PNPLA4 |
| gene22755 | -1.734880225 | 0.001914305 | 0.32 | 0.97 | 0.88 | 1.55 | 0.37 | 2.34 | 0.53 | 2.23 | 0.23 | 0.32 | 0.31 | 0.48 | AMEL |
| gene22767 | 1.229947011 | 0.000288125 | 1.28 | 0.51 | 1.97 | 1.12 | 2.04 | 1.69 | 0.73 | 7.01 | 1.75 | 8.03 | 1.87 | 1.45 | EGFL6 |
| gene22774 | 1.44569764 | 0.000537407 |  |  |  |  |  |  |  |  |  |  |  |  |  |
| gene22782 | -1.609525964 | 8.42E-06 | 1.1 | 3.38 | 4.71 | 5.01 | 2.83 | 9.14 | 3.26 | 2.13 | 1.99 | 1.75 | 2.02 | 1.58 | FIGF |
| gene22810 | -1.298517056 | 8.18E-05 | 5.1 | 2.88 | 4.82 | 9.71 | 16.09 | 12.25 | 11.45 | 6.8 | 4.97 | 4.23 | 4.12 | 6.67 | PPEF1 |
| gene22833 | 1.530404307 | 2.26E-06 | 2729.4 | 1674.9 | 1679.2 | 894.05 | 989.92 | 1573.1 | 1715.7 | 3702.5 | 3053.9 | 3089.6 | 3989.5 | 2476.1 | SAT1 |
| gene22886 | -1.006341151 | 0.002496812 | 9.9 | 10.88 | 7.84 | 26.59 | 6.94 | 9.1 | 3.23 | 13.45 | 12.98 | 8.68 | 6.34 | 5.59 | LOC100738608 |
| gene22888 | -1.275793515 | 0.00011367 | 3.1 | 2.86 | 2.47 | 9.97 | 3.05 | 4.37 | 1.14 | 5.86 | 6.03 | 2.95 | 2.41 | 1.62 | DMD |
| gene22897 | 3.586628585 | 0.002185737 |  |  |  |  |  |  |  |  |  |  |  |  |  |
| gene22912 | 1.295472918 | 7.58E-05 | 30.01 | 45.27 | 27.56 | 15.3 | 13.97 | 34.9 | 14.31 | 32.46 | 28.37 | 43.51 | 45.15 | 60.75 | LOC100623926 |
| gene22915 | 2.012306459 | 0.002960981 | 0.06 | 0.02 | 0.05 | 0.04 | 0.04 | 0.08 | 0.21 | 0.41 | 0.39 | 0.27 | 0.29 | 0.11 | LOC100625302 |
| gene22928 | 1.144266372 | 0.002350262 | 0.68 | 0.66 | 0.66 | 0.52 | 0.39 | 0.72 | 0.83 | 1.19 | 1.18 | 0.86 | 1.07 | 1.53 | LOC100511730 |
| gene22932 | -1.454793897 | 7.00E-06 | 283.4 | 142.13 | 312.06 | 338.64 | 644.86 | 265.48 | 290.96 | 118.62 | 177.21 | 141.04 | 159.07 | 145.38 | LOC100736958 |
| gene22986 | -1.83117956 | 9.49E-07 | 4.51 | 1.83 | 9.11 | 9.02 | 4.1 | 3.82 | 0.98 | 3.38 | 0.44 | 1.23 | 1.85 | 1.57 | LOC100523295 |
| gene22998 | -2.464851319 | 9.34E-09 | 0.45 | 0.16 | 0.32 | 0.74 | 1.77 | 1.06 | 0.45 | 0.24 | 0.42 | 0.31 | 0.13 | 0.25 |  |
| gene23026 | -1.052750588 | 0.003067011 | 4.11 | 3.88 | 3.53 | 5.25 | 5.9 | 4.42 | 4.2 | 4.8 | 3.24 | 2.65 | 2.73 | 2.01 | SYP |
| gene23037 | -1.22033918 | 0.000900722 | 0.3 | 1.37 | 0.55 | 0.48 | 1.05 | 1.36 | 0.66 | 0.43 | 1.67 | 0.54 | 0.48 | 0.2 | LOC100515638 |
| gene23056 | -4.875466293 | 0.001753584 |  |  |  |  |  |  |  |  |  |  |  |  |  |
| gene23080 | 1.030226111 | 0.0019556 | 16.44 | 2.72 | 7.05 | 4.1 | 3.59 | 4.19 | 1.8 | 2.38 | 3.23 | 8.67 | 2.31 | 12.54 | LOC100518817 |
| gene23093 | -1.234186738 | 0.000233348 | 33.65 | 34.88 | 40.93 | 74.64 | 104.76 | 65.87 | 88.15 | 63.04 | 35.64 | 37.75 | 30.74 | 33.88 | LOC100152387 |
| gene23125 | 1.251494313 | 0.001492931 | 1.06 | 1.2 | 0.85 | 0.36 | 0.87 | 0.99 | 1.04 | 0.8 | 1.26 | 1.87 | 1.54 | 1.66 | LOC100518049 |
| gene23217 | -2.326363612 | 0.000757105 | 0.35 | 0.42 | 0.13 | 0.41 | 0.31 | 0.32 | 0.42 | 0.05 | 0.13 | 0 | 0.16 | 0.04 |  |
| gene23242 | -1.74141589 | 0.005041377 | 1.34 | 0.58 | 1.61 | 1.55 | 2.01 | 3.47 | 0.32 | 0.28 | 0.94 | 1.31 | 0.21 | 0.38 | LOC100620814 |
| gene23249 | -1.605146901 | 2.26E-05 | 0.88 | 0.93 | 1.38 | 1.34 | 2.39 | 1.58 | 0.91 | 0.3 | 0.45 | 0.72 | 0.71 | 0.32 | RPS6KA6 |
| gene23254 | 1.040280815 | 0.002159185 | 1.59 | 2.27 | 2.25 | 1.29 | 1.4 | 3.51 | 2.36 | 3.89 | 2.16 | 4.7 | 3.59 | 3.84 | ZNF711 |
| gene23255 | 2.479697076 | 9.11E-13 | 2.88 | 3.48 | 1.16 | 2.07 | 2.87 | 1.55 | 8.68 | 6.16 | 20.24 | 12.86 | 6.52 | 15.97 | POF1B |
| gene23278 | -1.050904036 | 0.001749214 | 5.18 | 5.04 | 4.64 | 9.65 | 6.29 | 4.77 | 4.05 | 5.21 | 4.62 | 3.4 | 3.61 | 2.73 | LOC100739034 |
| gene23290 | 1.505565998 | 0.000377026 | 0.23 | 0.88 | 0.47 | 0.26 | 0.2 | 0.35 | 0.4 | 0.61 | 0.73 | 1 | 0.69 | 0.48 | PCDH19 |
| gene23297 | 1.298393095 | 0.006010537 | 0.68 | 0.6 | 1.39 | 0.75 | 0.33 | 0.32 | 0.44 | 0.98 | 0.89 | 0.62 | 1.5 | 1.12 | LOC100739747 |
| gene23304 | 2.810220479 | 0.002185737 | 0.04 | 0.05 | 0.02 | 0.02 | 0.02 | 0 | 0.04 | 0.11 | 0.07 | 0.02 | 0.06 | 0.17 | DRP2 |
| gene23319 | -1.512994402 | 0.003377167 | 0.73 | 0.42 | 0.96 | 1.17 | 0.99 | 1.24 | 0.33 | 0.66 | 0.16 | 0.29 | 0.36 | 0.5 | LOC100621834 |
| gene23326 | -1.434514221 | 1.09E-05 | 15.36 | 18.57 | 29.06 | 40.55 | 16.56 | 18.05 | 3.82 | 12.96 | 9.22 | 11.06 | 7.9 | 8.09 | LOC100151894 |
| gene23328 | 2.456484035 | 1.11E-05 | 0 | 0.38 | 0.17 | 0.51 | 0.14 | 0.18 | 0.66 | 3.36 | 0.88 | 2.04 | 1.49 | 0.81 | LOC100621480 |
| gene23329 | 2.913942761 | 2.03E-06 |  |  |  |  |  |  |  |  |  |  |  |  |  |
| gene23336 | 2.176369052 | 3.29E-07 | 5.91 | 6.21 | 7.29 | 4.18 | 3.78 | 3.2 | 2.21 | 30.69 | 8.64 | 20.05 | 15.93 | 12.16 | LOC100522845 |
| gene23338 | -1.117508627 | 0.003056471 | 0.44 | 0.61 | 0.92 | 0.63 | 1.11 | 0.66 | 0.26 | 0.4 | 0.38 | 0.21 | 0.46 | 0.43 | LOC100523039 |
| gene23346 | -2.971278324 | 0.00611413 | 0 | 0.08 | 0 | 0.59 | 1.15 | 0.18 | 0.2 | 0.66 | 0.44 | 0.2 | 0 | 0 | LOC100524125 |
| gene23362 | -3.505563223 | 3.04E-05 | 4.94 | 7.2 | 2.92 | 3.55 | 1.58 | 6.74 | 1.55 | 2.01 | 0.96 | 0.45 | 0.43 | 0 | LOC100525848 |
| gene23384 | -2.241298713 | 0.001496317 | 0.31 | 0.49 | 0.65 | 0.47 | 1.02 | 0.32 | 0.39 | 0.05 | 0.31 | 0.29 | 0 | 0.12 | LOC100627548 |
| gene2339 | -1.009950205 | 0.002349551 | 2.46 | 3.09 | 3.15 | 4.04 | 4.6 | 4.2 | 2.72 | 2.27 | 2.04 | 1.53 | 1.93 | 2.68 | MAMDC4 |
| gene23405 | -1.043398572 | 0.001442492 | 7.92 | 11.62 | 9.42 | 8.69 | 9.04 | 37.38 | 8.41 | 8.23 | 6.11 | 7.07 | 12.58 | 5.51 | CHRDL1 |
| gene2341 | -2.074081846 | 0.000234558 | 1.07 | 1.25 | 1.05 | 1.14 | 1.38 | 1.4 | 0.69 | 0.12 | 0.6 | 0.65 | 0.18 | 0.24 | LCN8 |
| gene23417 | -1.029069055 | 0.008362968 | 2.05 | 2.7 | 1.33 | 2.03 | 2.23 | 2.55 | 1.66 | 0.94 | 1.22 | 0.87 | 1.31 | 1.04 | LOC100523160 |
| gene23436 | -1.673853747 | 1.99E-06 |  |  |  |  |  |  |  |  |  |  |  |  |  |
| gene23463 | 1.544803274 | 2.26E-06 | 21.79 | 33.46 | 24.09 | 9.38 | 8.3 | 9.74 | 7.4 | 11.9 | 22.33 | 20.07 | 20.2 | 36.52 | LONRF3 |
| gene23473 | 1.40542543 | 0.000379967 | 0.47 | 0.48 | 1.05 | 0.91 | 0.71 | 0.68 | 0.72 | 0.92 | 0.71 | 2.59 | 1.62 | 1.65 |  |
| gene23497 | 2.093210854 | 0.007254728 | 0.16 | 0.71 | 0.14 | 0 | 0.06 | 0.31 | 0.09 | 0.11 | 0.19 | 1.2 | 0 | 0.38 | RAB33A |
| gene23525 | -1.027071764 | 0.001483722 | 11.55 | 11.94 | 19.28 | 29.08 | 26.25 | 38.61 | 13.36 | 20.65 | 11.6 | 18.85 | 13.49 | 12.29 | GPC3 |
| gene23526 | 1.473995271 | 0.000255692 | 3.08 | 5.67 | 3.64 | 0.78 | 0.8 | 1.77 | 0.95 | 2.53 | 2.54 | 2.4 | 1.81 | 4.34 | LOC100516533 |
| gene23528 | 1.019227559 | 0.002953767 | 8.96 | 10.73 | 11.29 | 7.6 | 9.17 | 9.74 | 7.27 | 7.1 | 12.47 | 10.44 | 16.67 | 24.24 | PHF6 |
| gene23538 | -1.085545603 | 0.002827998 | 30.54 | 32.45 | 32.9 | 32.94 | 27.38 | 33.58 | 11.22 | 11.43 | 14.26 | 14.57 | 15.46 | 12.64 | LOC100521310 |
| gene23541 | -1.32846512 | 0.000689573 | 2.91 | 4.13 | 2.92 | 3.18 | 3.31 | 4.43 | 1.81 | 1.13 | 1.56 | 1.67 | 1.2 | 1.41 | LOC100521652 |
| gene23578 | 2.399581643 | 5.19E-08 | 2.25 | 2.32 | 0.1 | 0.07 | 0.27 | 0.13 | 2.98 | 1.14 | 0.53 | 1.33 | 0.7 | 0.46 | LOC100525800 |
| gene23594 | -1.707363505 | 2.22E-05 | 1.62 | 1.54 | 2.06 | 3.06 | 2.4 | 2.11 | 1.88 | 1.13 | 1.12 | 1.16 | 0.71 | 0.51 | LOC100513063 |
| gene23616 | -1.54550968 | 2.76E-06 | 11.1 | 8.75 | 11.62 | 4.99 | 59.11 | 8.73 | 31.45 | 3.29 | 5.57 | 8.92 | 6.85 | 9.18 | GABRE |
| gene23671 | 2.525391794 | 9.58E-08 |  |  |  |  |  |  |  |  |  |  |  |  |  |
| gene23733 | 1.801327055 | 1.09E-06 |  |  |  |  |  |  |  |  |  |  |  |  |  |
| gene23747 | 1.417503796 | 8.69E-05 | 1.7 | 9.35 | 4.2 | 1.57 | 3.33 | 2.2 | 2.33 | 6.06 | 8.24 | 4.75 | 9.47 | 3.93 | SLC7A11 |
| gene23748 | 1.742675514 | 9.04E-08 | 882.06 | 825.64 | 933.99 | 383.69 | 383.06 | 276.92 | 590.66 | 895.37 | 787.52 | 991.75 | 1130.9 | 1256.9 | LOC100628052 |
| gene23757 | -1.595723608 | 0.000140122 | 1.77 | 3.86 | 3.89 | 2.44 | 4.26 | 6.78 | 4.15 | 1.29 | 1.21 | 1.88 | 1.46 | 0.99 | LOC100620951 |
| gene23763 | 1.50325863 | 0.000353419 | 1.23 | 1.03 | 1.21 | 0.44 | 0.43 | 0.66 | 1.39 | 0.36 | 0.65 | 1.98 | 1.46 | 0.62 | IRK1 |
| gene238 | 1.677971876 | 0.000737041 | 1.81 | 0.43 | 1.09 | 0.32 | 0.36 | 0.28 | 0.28 | 0.45 | 2.63 | 1.95 | 0.31 | 0.9 | ARG1 |
| gene23894 | -1.216560158 | 0.000304168 | 5.43 | 5.01 | 18.66 | 20.08 | 6.98 | 7.82 | 1.79 | 12.42 | 4.8 | 5.5 | 3.94 | 5.12 | LOC100627175 |
| gene23930 | 1.600779088 | 8.60E-05 | 0.35 | 0.5 | 0.48 | 0.25 | 0.19 | 0.34 | 0.38 | 0.67 | 0.69 | 0.97 | 0.63 | 0.62 | LOC100623020 |
| gene24003 | -1.315354239 | 0.000709613 | 1.52 | 0.7 | 0.94 | 0.75 | 3.48 | 1.06 | 1.16 | 0.48 | 0.31 | 0.91 | 0.56 | 0.69 | LOC100622675 |
| gene24019 | -1.215215595 | 0.000166629 | 41.4 | 45.35 | 31.69 | 69.48 | 90.44 | 96.76 | 74.2 | 27.97 | 39.54 | 29.55 | 30.07 | 46.88 | LOC100624383 |
| gene24032 | -1.45504455 | 0.002827583 | 0.43 | 1.83 | 1.52 | 2.69 | 3.25 | 6.61 | 2.3 | 2.25 | 1.5 | 0.93 | 1.56 | 1.84 | LOC780407 |
| gene24046 | -1.040591869 | 0.00195818 | 5.09 | 5.75 | 4.08 | 6.22 | 4.75 | 5.16 | 2.87 | 2.66 | 1.72 | 3.27 | 1.83 | 2.56 | LOC100627176 |
| gene24063 | 2.486899504 | 1.43E-10 | 0.29 | 0.39 | 0.13 | 0.32 | 0.16 | 0.24 | 1.62 | 2.05 | 0.81 | 1.77 | 1.13 | 1.1 | LOC100620661 |
| gene24077 | 2.817486525 | 0.008284963 | 0.99 | 1.47 | 1.13 | 0 | 0.33 | 0 | 2.16 | 0.44 | 0.25 | 3.23 | 0 | 0 | LOC100622016 |
| gene24081 | -4.876892528 | 0.001753584 | 0.09 | 0.07 | 0.11 | 0.1 | 0.08 | 0.06 | 0.02 | 0.03 | 0.05 | 0 | 0 | 0 | LOC100737765 |
| gene2410 | -1.043596242 | 0.001256694 | 32.56 | 29.26 | 35.58 | 30.75 | 38.34 | 52.45 | 30.34 | 18.29 | 17.55 | 24.92 | 17.07 | 15.24 | CHID1 |
| gene2414 | -1.215829977 | 0.00034951 | 3.24 | 3.01 | 3.81 | 7.43 | 9.72 | 7.91 | 6.99 | 3.33 | 3.24 | 3.57 | 3.25 | 3.68 | EFCAB4A |
| gene24140 | 1.47022363 | 0.000476648 | 1.44 | 2.29 | 1.65 | 0.53 | 0.15 | 0.56 | 0.24 | 0.16 | 0.34 | 1.15 | 0.92 | 1.18 | UNC5CL |
| gene24151 | 1.327198371 | 5.30E-05 | 9.92 | 15.95 | 12.9 | 9.2 | 6.99 | 10.14 | 6.11 | 16.26 | 11.47 | 18.16 | 19.55 | 25.55 | LOC100621279 |
| gene24157 | -3.154870359 | 1.13E-13 | 0.92 | 1.22 | 2.07 | 5.11 | 5.03 | 1.4 | 0.57 | 1.39 | 0.62 | 0.2 | 0.48 | 0.6 | LOC100621966 |
| gene24188 | -1.694981866 | 0.001829243 | 0.76 | 0.69 | 2.01 | 3.41 | 0.87 | 3.28 | 0.91 | 0.4 | 2.21 | 0 | 0.4 | 1.81 | LOC100625477 |
| gene24217 | -3.082855188 | 0.00364903 | 0.48 | 0.21 | 0.05 | 0.28 | 0.12 | 0.2 | 0.06 | 0.22 | 0.12 | 0.06 | 0 | 0 | LOC100628216 |
| gene2422 | -1.041339474 | 0.001910715 | 16.12 | 25.66 | 23.67 | 24.11 | 31.66 | 25.34 | 22.23 | 18.01 | 15.03 | 14.66 | 11.75 | 12.1 | TMEM80 |
| gene24268 | -2.517508247 | 1.04E-12 | 67.68 | 44.48 | 77.76 | 45.38 | 57.96 | 23.75 | 9.48 | 16.58 | 11.5 | 6.44 | 10.67 | 4.75 | HSD17B2 |
| gene24280 | 2.317312484 | 3.33E-06 |  |  |  |  |  |  |  |  |  |  |  |  |  |
| gene24293 | -2.459294112 | 0.008284963 | 0.02 | 0.03 | 0.01 | 0.06 | 0.01 | 0.03 | 0 | 0.01 | 0 | 0.01 | 0 | 0.01 |  |
| gene24296 | -2.62641264 | 0.00153302 | 0.58 | 0.75 | 0.55 | 0.93 | 0.89 | 0.62 | 0.17 | 0.3 | 0.47 | 0.23 | 0 | 0.21 | LOC100517709 |
| gene2431 | -1.409016736 | 0.006329004 | 0.14 | 0.21 | 0.32 | 0.47 | 0.25 | 0.66 | 0.24 | 0.57 | 0.18 | 0.07 | 0.19 | 0.26 | LOC100514751 |
| gene24316 | 1.521767927 | 0.00655525 | 0.13 | 0.05 | 0.33 | 0.22 | 0.09 | 0.08 | 0.41 | 0.12 | 0.3 | 0.4 | 0.36 | 0.24 | LOC100155978 |
| gene24328 | -1.456416236 | 0.001776499 | 0.25 | 0.38 | 0.46 | 0.51 | 0.44 | 0.43 | 0.19 | 0.14 | 0.22 | 0.17 | 0.14 | 0.2 |  |
| gene24332 | -3.433599153 | 4.53E-05 | 0.11 | 0.14 | 2.12 | 1.6 | 0 | 0.66 | 0 | 0.12 | 0.2 | 0.19 | 0 | 0 | LOC100623371 |
| gene2434 | 1.115406318 | 0.000575363 | 229.1 | 129.67 | 123.34 | 87.61 | 56.37 | 77 | 120.3 | 129.67 | 182.51 | 88.45 | 137.96 | 230.16 | PHLDA2 |
| gene24340 | -1.209951455 | 0.000261001 | 11.59 | 12.63 | 9.65 | 14.24 | 14.09 | 22.53 | 8.72 | 8.03 | 7.7 | 8.63 | 6.96 | 5.69 |  |
| gene24394 | -1.946425887 | 7.86E-08 | 2.21 | 1.99 | 2.99 | 4.72 | 2.02 | 2.95 | 0.78 | 3.07 | 2.66 | 1.33 | 0.56 | 0.63 | LOC100621442 |
| gene24400 | -1.723064041 | 2.38E-06 | 0.81 | 0.68 | 1.74 | 0.78 | 3.25 | 1.1 | 0.93 | 0.32 | 0.4 | 0.51 | 0.3 | 0.75 | CCDC33 |
| gene24409 | 1.414420484 | 1.22E-05 | 53.16 | 60.14 | 65.16 | 45.03 | 32.09 | 46.63 | 70.73 | 87.61 | 110.59 | 66.7 | 145.27 | 101.43 | PHLDB2 |
| gene24423 | 1.206296635 | 0.000217898 | 11.16 | 12.19 | 12.61 | 7.8 | 8.9 | 14.1 | 7.92 | 15.31 | 16.45 | 17.69 | 18.43 | 31.77 | LOC100624385 |
| gene24433 | -1.856028661 | 4.68E-05 | 0.8 | 0.57 | 0.96 | 1.05 | 1.27 | 0.88 | 0.91 | 0.15 | 0.41 | 0.27 | 0.22 | 0.34 | LOC100625479 |
| gene24434 | 1.638606497 | 0.004836139 | 0.08 | 0.04 | 0.08 | 0.05 | 0.16 | 0.06 | 0.03 | 0.18 | 0.1 | 0.29 | 0.52 | 0.03 | LOC100625383 |
| gene24455 | -2.029435552 | 0.004164925 | 0.11 | 0.21 | 0.55 | 0.13 | 0.59 | 0.6 | 0.06 | 0.04 | 0.2 | 0 | 0 | 0.27 | LOC100627551 |
| gene24465 | 3.617662848 | 1.04E-05 | 0.06 | 0 | 0.08 | 0.07 | 0.07 | 0 | 1.02 | 0.53 | 0.45 | 1.04 | 0.4 | 0.91 | LOC100620598 |
| gene24467 | -5.411604205 | 0.000102878 | 0 | 0 | 0 | 0.03 | 0 | 0.62 | 0 | 0 | 0.04 | 0 | 0 | 0 | LOC100620789 |
| gene24484 | 3.65270951 | 5.17E-23 | 344.17 | 9.88 | 40.06 | 1.82 | 8.5 | 10.46 | 10.92 | 94.21 | 3.62 | 135.09 | 111 | 7.78 | HSP70 |
| gene24485 | 2.26465922 | 7.62E-10 | 2.5 | 0.56 | 0.72 | 0.4 | 0.57 | 0.91 | 2.37 | 5.19 | 3.32 | 2.46 | 4.21 | 1.88 | LOC100623207 |
| gene2449 | -2.284328827 | 0.000338567 | 0.97 | 1.91 | 1.43 | 2.02 | 1.24 | 2.17 | 0.17 | 1.47 | 3.01 | 0.53 | 0.34 | 0.15 | FADD |
| gene24507 | 1.777651902 | 0.004451698 | 0.01 | 0.01 | 0.01 | 0.01 | 0 | 0 | 0 | 0.01 | 0.01 | 0 | 0.02 | 0.03 |  |
| gene24509 | 1.880797169 | 0.008227848 | 0.51 | 0.28 | 0 | 0.13 | 0.07 | 0.08 | 0.37 | 0.3 | 0.2 | 0.28 | 0.4 | 0.36 | LOC100625723 |
| gene24532 | 1.281084223 | 0.008167542 | 1.12 | 0.97 | 1.93 | 0.85 | 0.46 | 0.95 | 0.69 | 1.45 | 1.27 | 3.54 | 0.94 | 1.04 | CYP19A1 |
| gene24558 | 3.364395123 | 1.05E-20 | 419.59 | 9.42 | 52.85 | 5.69 | 10.45 | 13.1 | 10.29 | 105.48 | 6.52 | 163.73 | 124.28 | 5.87 | HSP70 |
| gene2456 | 1.221368105 | 0.000348108 | 1.36 | 3.24 | 4.1 | 2.01 | 1.68 | 2.98 | 2.37 | 3.85 | 3.38 | 3.78 | 5.66 | 5.28 | LOC100738137 |
| gene24566 | -1.336666835 | 0.004695089 | 0.41 | 0.41 | 0.63 | 0.84 | 1.3 | 1.08 | 0.14 | 0.89 | 0.24 | 0.76 | 0.32 | 0.24 | LOC100623996 |
| gene2457 | 1.223906368 | 0.00039701 | 1.67 | 2.55 | 3.04 | 1.65 | 2.14 | 2.98 | 2.28 | 2.61 | 3.62 | 2.72 | 6.04 | 6.2 | ANO1 |
| gene24664 | 2.141482512 | 3.04E-07 | 0.37 | 0.34 | 0.41 | 0.41 | 0.19 | 0.35 | 0.82 | 1.24 | 0.93 | 1.83 | 0.81 | 1.35 |  |
| gene24673 | -5.43840883 | 0.000102878 | 0 | 0 | 0.04 | 0.51 | 0 | 0.12 | 0 | 0 | 0 | 0 | 0 | 0 | LOC100627396 |
| gene24674 | 1.550625839 | 2.78E-05 | 0.37 | 0.14 | 0.2 | 0.75 | 0.35 | 4.45 | 9.01 | 0.04 | 0 | 15.34 | 0.06 | 0.05 | FGA |
| gene24681 | 1.033143878 | 0.002841409 | 26.06 | 16.21 | 23.58 | 19.44 | 14.71 | 15.71 | 18.43 | 20.9 | 33.24 | 20.87 | 37.18 | 39.24 | CHMP4C |
| gene24696 | 3.805094716 | 0.000481426 | 0 | 0.15 | 0.32 | 0 | 0.14 | 0 | 0 | 0.38 | 0.42 | 0.78 | 2.09 | 0 | LOC100621670 |
| gene24737 | -1.624438267 | 0.002056503 | 1.12 | 1.54 | 0.62 | 0.76 | 2.44 | 0.51 | 0.33 | 0.99 | 0.41 | 0.38 | 0.56 | 0.25 | LOC100625748 |
| gene24767 | -1.203959116 | 0.000601969 | 0.87 | 1.2 | 3.26 | 2.34 | 2.99 | 5.39 | 0.65 | 1.66 | 0.55 | 2.22 | 1.09 | 1.18 | LOC100736941 |
| gene24784 | 1.298275139 | 0.001360776 | 2.71 | 0.53 | 0.98 | 1.45 | 0.33 | 0.77 | 2.31 | 1.5 | 1.51 | 1.67 | 3.63 | 0.77 | LOC100737314 |
| gene24791 | 1.312652952 | 0.002877512 |  |  |  |  |  |  |  |  |  |  |  |  |  |
| gene24797 | 2.10966481 | 4.77E-05 | 0.08 | 0.59 | 0.7 | 0 | 0.83 | 0.81 | 2.01 | 1.65 | 1.83 | 1.05 | 2.92 | 2.42 | LOC100623805 |
| gene24799 | 2.796229263 | 2.95E-14 | 0.35 | 1.13 | 2.26 | 0.62 | 0.43 | 0.24 | 0.98 | 1.67 | 2.85 | 3.45 | 3.29 | 2.08 | LOC100624264 |
| gene24802 | -3.20053437 | 3.42E-10 | 1.31 | 1.69 | 2.24 | 1.5 | 2.62 | 1.07 | 1.19 | 0.44 | 0.37 | 0.26 | 0.1 | 0.3 | LOC100624526 |
| gene24844 | -1.516586219 | 0.000487055 | 0.52 | 0.5 | 1.03 | 0.54 | 1.14 | 0.81 | 0.23 | 0.16 | 0.41 | 0.28 | 0.24 | 0.31 | LOC100514803 |
| gene24855 | 1.21773622 | 0.000498679 | 7.2 | 6.67 | 6.9 | 6.61 | 5.71 | 5.09 | 8.21 | 12.61 | 15.08 | 11.02 | 11.39 | 16.45 | LOC100621868 |
| gene2486 | -1.49620271 | 0.003390801 | 0.05 | 0.49 | 0.68 | 0.46 | 0.85 | 1.34 | 0.16 | 0.21 | 0.34 | 0.48 | 0.17 | 0.23 | LOC100525027 |
| gene24876 | -1.313093645 | 0.003935584 | 0.76 | 0.42 | 0.64 | 0.35 | 0.53 | 1.52 | 0.11 | 0.26 | 0.44 | 0.34 | 0.32 | 0.23 | COCH |
| gene24890 | 1.999845924 | 0.004164925 |  |  |  |  |  |  |  |  |  |  |  |  |  |
| gene24905 | 3.178411911 | 1.42E-16 | 0.32 | 0.44 | 0.45 | 0.66 | 0.47 | 0.72 | 2.7 | 11.53 | 7.48 | 5.13 | 6.07 | 4.56 | LOC100627397 |
| gene24919 | 4.658532251 | 0.005776466 | 0 | 0.02 | 0.04 | 0 | 0 | 0 | 0 | 0.07 | 0 | 0.11 | 0.1 | 0.19 | LOC100156578 |
| gene24929 | 1.000680423 | 0.002591581 | 2.31 | 2.45 | 0.77 | 2.26 | 2.76 | 2.44 | 6.28 | 6.02 | 3.66 | 5.88 | 4.72 | 3.95 | LOC100622045 |
| gene24948 | -1.228739483 | 0.001083645 |  |  |  |  |  |  |  |  |  |  |  |  |  |
| gene2495 | -1.119489587 | 0.001042793 | 10.1 | 9.74 | 8.07 | 14.85 | 14.56 | 16.02 | 12.75 | 8.32 | 11.35 | 6.34 | 6.91 | 7.03 | LOC100511242 |
| gene24952 | 2.152983887 | 0.000350506 | 0.17 | 0.42 | 0.08 | 0.2 | 0.13 | 0.41 | 0.78 | 0.9 | 0.2 | 1.4 | 1.09 | 0.74 | LOC100738419 |
| gene24953 | -1.284391392 | 0.000630049 | 2.78 | 2.99 | 3.16 | 4.74 | 7.77 | 6.4 | 5.89 | 4.37 | 3.82 | 2.15 | 3.13 | 2.18 | LOC100624445 |
| gene24961 | -6.123768761 | 6.02E-07 | 2.8 | 0.58 | 1.5 | 0.56 | 0.96 | 0.6 | 0 | 0.12 | 0 | 0 | 0 | 0 | LOC100523970 |
| gene25039 | -3.880319073 | 7.48E-25 | 67.16 | 53.29 | 30.58 | 71.12 | 38.01 | 38.39 | 10.78 | 2.03 | 6.51 | 1.94 | 2.47 | 5.12 | DAPK2 |
| gene25051 | -2.446716436 | 1.14E-06 | 0.03 | 0.39 | 0.89 | 0.49 | 2.02 | 0.97 | 0.53 | 0.38 | 0.22 | 0.21 | 0.25 | 0.14 | KATNAL2 |
| gene25061 | -1.107723462 | 0.000785148 | 3.02 | 2.87 | 3.84 | 4.41 | 4.51 | 4.47 | 1.28 | 1.59 | 1.85 | 1.39 | 2.2 | 2.39 | LOC100628058 |
| gene25069 | -1.442992361 | 0.000798635 | 0.04 | 0.1 | 0.31 | 0.29 | 0.36 | 0.31 | 0.14 | 0.28 | 0.11 | 0.18 | 0.07 | 0.09 |  |
| gene25072 | -1.201619403 | 0.003916844 | 0.61 | 1.2 | 0.87 | 1.43 | 1.86 | 1.08 | 1.77 | 1.24 | 0.81 | 0.61 | 0.8 | 0.49 | LOC100620765 |
| gene25075 | -2.509851134 | 3.10E-10 | 3.11 | 2.15 | 3.14 | 3.59 | 2.87 | 3.34 | 0.76 | 1.03 | 0.27 | 0.88 | 0.77 | 0.07 | LOC100621444 |
| gene25088 | 1.135808664 | 0.000542401 | 2.89 | 4.1 | 4.33 | 2.69 | 2.88 | 4.07 | 3.38 | 4.79 | 6.92 | 4.15 | 7.34 | 8.69 | LOC100622995 |
| gene25097 | -1.75847142 | 1.38E-05 | 3.07 | 0.36 | 0.49 | 0.65 | 1.6 | 2.6 | 1.48 | 0.45 | 1.25 | 0.5 | 0.78 | 0.12 | SCG2 |
| gene25106 | -1.880518057 | 9.72E-06 | 0.83 | 0.38 | 0.81 | 0.65 | 1.35 | 1.82 | 0.74 | 0.11 | 0.75 | 0.52 | 0.24 | 0.27 | LOC100624689 |
| gene25112 | 4.665769643 | 0.005776466 | 0.06 | 0 | 0 | 0 | 0 | 0 | 0.23 | 0.12 | 0 | 0 | 0.44 | 0.24 | LOC100738141 |
| gene25121 | -1.179717654 | 0.007348063 | 1.04 | 1.83 | 1.27 | 0.81 | 1.39 | 3.09 | 1.15 | 1.08 | 2.21 | 0.59 | 0.79 | 0.85 | LOC100626221 |
| gene25143 | -3.260555789 | 0.002185737 |  |  |  |  |  |  |  |  |  |  |  |  |  |
| gene25190 | 1.994914903 | 1.65E-09 | 20.72 | 32.74 | 13.78 | 11.38 | 13.57 | 8.64 | 23.37 | 27.49 | 18.35 | 30.68 | 40.18 | 58.46 | LOC100625277 |
| gene25195 | -2.047440542 | 9.41E-05 | 0.68 | 0.82 | 0.78 | 0.56 | 0.44 | 0.74 | 0.21 | 0.18 | 0.1 | 0.25 | 0.15 | 0 | LOC100516478 |
| gene25200 | -1.544098205 | 6.25E-06 | 26.2 | 18.46 | 28.62 | 29.7 | 43.74 | 27.49 | 39.61 | 7.57 | 12.16 | 16.79 | 10.03 | 7.57 | LOC100626400 |
| gene2521 | -1.54044953 | 5.01E-05 | 1.12 | 1.17 | 2.09 | 1.15 | 1.76 | 2.27 | 1.89 | 0.87 | 0.23 | 0.64 | 0.65 | 0.45 | PC |
| gene25232 | -1.435206968 | 0.000583023 | 4.1 | 3.13 | 5.64 | 6.19 | 5.06 | 12.89 | 2.47 | 1.53 | 2.82 | 3.81 | 2.31 | 2.31 | LOC100621994 |
| gene25242 | -1.756529372 | 0.006337787 | 0.08 | 0.12 | 0.33 | 1.05 | 0 | 0.04 | 0.09 | 0.18 | 0 | 0.05 | 0.13 | 0.12 | LOC100623147 |
| gene25250 | 1.55121377 | 0.002588055 | 0.22 | 0.11 | 0.21 | 0.34 | 0.1 | 0.03 | 0.47 | 0.82 | 0.62 | 0.22 | 0.77 | 0.42 | LOC100624083 |
| gene25253 | 1.203995157 | 0.000176004 | 5293.1 | 6803.3 | 3921.6 | 3856.1 | 3216.6 | 3847.9 | 5684.8 | 9897.9 | 7930.4 | 6708.7 | 9176.1 | 8240.9 | PTMA |
| gene25260 | 1.972607266 | 5.54E-08 | 0.45 | 1.47 | 0.9 | 0.67 | 0.83 | 0.8 | 2.5 | 5.72 | 1.06 | 0.73 | 6.11 | 1.59 | LOC100037974 |
| gene25264 | 1.697531139 | 0.006540354 | 0.29 | 0.36 | 0.51 | 0.06 | 0.28 | 0.35 | 0.54 | 0.76 | 0.34 | 0.55 | 0.53 | 1.11 | LOC100625687 |
| gene25267 | 3.301549135 | 3.43E-19 | 0.25 | 3.63 | 0.13 | 0.09 | 2.66 | 0.18 | 5.43 | 10.87 | 8.99 | 5.11 | 12.82 | 11.02 | LOC100625877 |
| gene25284 | 1.601481333 | 5.87E-05 | 0.15 | 0.51 | 0.1 | 0.4 | 0.42 | 0.41 | 1.97 | 1.71 | 0.93 | 1.42 | 1.41 | 0.82 | F13B |
| gene2530 | -1.984647449 | 0.000839906 | 0.09 | 0.15 | 0.22 | 0.29 | 0.32 | 0.15 | 0.35 | 0.12 | 0.05 | 0.09 | 0.02 | 0.08 | LOC100737994 |
| gene2531 | -1.068384088 | 0.003154575 | 7.86 | 4.45 | 5.11 | 10.28 | 9.58 | 8.45 | 6.3 | 5.48 | 5.06 | 5.39 | 4.98 | 2.91 | CCS |
| gene25338 | -1.082863729 | 0.001356308 | 36.06 | 38.92 | 34.27 | 37.81 | 41.53 | 45.85 | 37.3 | 25.54 | 25.59 | 29.24 | 15.19 | 13.52 | LOC100624982 |
| gene25350 | -1.937860245 | 5.44E-05 | 0.33 | 1.24 | 0.62 | 1.6 | 0.73 | 1.25 | 0.55 | 0.14 | 0.23 | 0.38 | 0.37 | 0.14 | LOC100525254 |
| gene25360 | 1.151260688 | 0.000388007 | 25.3 | 21.82 | 26.23 | 14.75 | 22.06 | 24.48 | 22.43 | 23.6 | 31.03 | 32.48 | 36.33 | 61.98 | LOC100627866 |
| gene25397 | 2.443854925 | 7.78E-11 | 4.07 | 9.64 | 4.47 | 1.36 | 2.04 | 1.31 | 1.79 | 6.37 | 6.95 | 7.76 | 12.23 | 4.53 | LOC100623148 |
| gene25401 | -1.391409992 | 0.000246559 | 2.78 | 2.79 | 2.39 | 2.88 | 3.27 | 2.42 | 3.09 | 1.97 | 1.96 | 1.22 | 0.73 | 1.23 | LOC100623655 |
| gene25420 | -2.295406676 | 1.43E-05 | 0.48 | 1.09 | 1.17 | 2.96 | 1.26 | 2.06 | 1.65 | 0.43 | 0.36 | 0.78 | 0.22 | 0.2 | LOC100736973 |
| gene25424 | -1.896732206 | 0.000258826 | 0.75 | 1.06 | 0.89 | 0.85 | 0.48 | 0.89 | 0.25 | 0.12 | 0.05 | 0.32 | 0.18 | 0.08 | LOC100519980 |
| gene25444 | 4.408338244 | 1.56E-17 | 0.3 | 0.07 | 0.25 | 0.04 | 0.22 | 0.22 | 0.18 | 4.09 | 0.07 | 3.37 | 4.83 | 0.16 |  |
| gene25465 | 1.303459791 | 0.000659685 | 5.31 | 4.67 | 2.21 | 2.5 | 1.86 | 3.07 | 2.42 | 3.05 | 5.14 | 4.57 | 8.44 | 4 | LOC100622233 |
| gene25512 | 3.004552091 | 4.76E-18 | 286.21 | 10.38 | 37.17 | 5.38 | 9.12 | 7.31 | 8.76 | 43.41 | 5.45 | 101.19 | 66.23 | 4.87 | HSP70 |
| gene2553 | -5.209871088 | 0.000312409 | 0.17 | 0.19 | 0.43 | 0.07 | 0.17 | 0.38 | 0.21 | 0.09 | 0.15 | 0 | 0 | 0 | LOC100522179 |
| gene25546 | -5.375039368 | 0.000178599 |  |  |  |  |  |  |  |  |  |  |  |  |  |
| gene25563 | -1.546968447 | 0.001582149 |  |  |  |  |  |  |  |  |  |  |  |  |  |
| gene25591 | 1.042152898 | 0.001287193 | 61.83 | 53.84 | 80.98 | 42.07 | 40.99 | 35.34 | 42.97 | 41.68 | 76.75 | 131.15 | 43.2 | 66.34 | DDIT3 |
| gene256 | -1.193237134 | 0.005456779 | 0.32 | 0.47 | 0.72 | 1.43 | 0.85 | 1.79 | 0.28 | 0.8 | 0.98 | 0.68 | 0.76 | 0.28 | LOC100622458 |
| gene25600 | 1.453990871 | 0.000541481 | 0.7 | 1.19 | 0.58 | 0.03 | 0.45 | 0.87 | 0.73 | 0.55 | 0.79 | 0.94 | 1.36 | 1.14 | LOC100620398 |
| gene25603 | 1.389218518 | 7.91E-05 | 0.32 | 0.96 | 0.51 | 0.59 | 1.96 | 0.53 | 0.5 | 0.35 | 0.32 | 6.96 | 0.81 | 0.5 | PC1/3 |
| gene25621 | 1.144478957 | 0.003822106 | 0.82 | 0.5 | 0.67 | 0.64 | 0.5 | 0.49 | 0.83 | 1.62 | 0.88 | 0.67 | 1.61 | 1.21 | LOC100622580 |
| gene25627 | -1.688111325 | 0.008591092 | 0.13 | 0.22 | 0.45 | 0.13 | 0.15 | 1.1 | 0.13 | 0.11 | 0.29 | 0.17 | 0.18 | 0.05 | LOC100623149 |
| gene2565 | 2.872821357 | 8.21E-17 | 30.51 | 30.27 | 23.75 | 7.96 | 5.89 | 19.66 | 25.58 | 42.63 | 29.79 | 28.75 | 63.67 | 136.55 | LOC100525205 |
| gene25673 | 1.121489964 | 0.000512351 | 257.95 | 36.24 | 36.91 | 38.81 | 34.44 | 33.64 | 28.23 | 29.85 | 30.82 | 183.14 | 28.25 | 22.05 | LOC100627777 |
| gene25697 | -1.056428614 | 0.006521216 | 1.01 | 0.6 | 0.85 | 3 | 2.32 | 1.47 | 1.41 | 2.71 | 1 | 1.26 | 1.38 | 0.58 | LOC100622048 |
| gene25704 | 2.127172204 | 8.46E-10 | 23.31 | 11.26 | 7.93 | 2.68 | 5.26 | 48.18 | 3.94 | 113.37 | 138.79 | 40.96 | 131.33 | 51.47 | NMB |
| gene25712 | 1.211510633 | 0.001525387 | 0.71 | 1.03 | 1.3 | 0.93 | 0.47 | 0.62 | 1.32 | 0.71 | 1.55 | 0.61 | 1.56 | 2.29 | LOC100623775 |
| gene25713 | -1.437138015 | 1.61E-05 | 11.77 | 14.33 | 9.2 | 64.49 | 4.74 | 51.01 | 9.18 | 21.03 | 9.21 | 17.95 | 12.21 | 12.3 | ISG15 |
| gene2572 | 1.058074835 | 0.0035758 | 0.53 | 0.8 | 1.03 | 1.54 | 1.62 | 1.17 | 3.12 | 4.62 | 2.77 | 4.67 | 2.86 | 1.3 | SNX32 |
| gene25736 | 4.57327761 | 5.24E-31 | 0.86 | 1.77 | 0.5 | 1.33 | 0.78 | 0.76 | 9.7 | 45.87 | 13.81 | 21.9 | 32.68 | 11.9 | LOC100626102 |
| gene25771 | 1.06426808 | 0.001096317 | 20 | 28.95 | 30.24 | 12.76 | 9.49 | 17.96 | 12.85 | 27.19 | 19.75 | 22.36 | 32.54 | 25.07 | LOC100621245 |
| gene25801 | 1.460343396 | 6.33E-06 | 1036.4 | 439.52 | 464.64 | 452.55 | 441.91 | 328.42 | 770.41 | 1180.8 | 666.45 | 1026.1 | 1301.9 | 926.8 | LOC100624472 |
| gene25820 | -1.644644156 | 4.69E-07 | 245.25 | 139.78 | 242.32 | 247.11 | 528.49 | 251.09 | 181.22 | 79.36 | 95.93 | 110.87 | 77.33 | 134.15 | CBR1 |
| gene25826 | 1.55187394 | 1.79E-06 | 79.43 | 130.22 | 106.39 | 75.24 | 48.14 | 121.5 | 55.19 | 193.97 | 163.2 | 133.96 | 264.58 | 278.62 | LOC100626719 |
| gene25839 | -1.873815879 | 0.000475525 | 12.73 | 0.35 | 0.91 | 7.28 | 0.79 | 0 | 5.03 | 1.56 | 2.81 | 0.56 | 0.36 | 1.15 | CYP3A46 |
| gene25843 | 4.841437391 | 0.003168798 | 0 | 0.58 | 0 | 0 | 0 | 0 | 0.06 | 0 | 0.27 | 0 | 1.11 | 0 | CYP2C36 |
| gene2588 | -1.020746179 | 0.007368676 | 3.1 | 5.25 | 4.14 | 2.46 | 3.76 | 3.45 | 2.63 | 2.28 | 1.86 | 0.98 | 1.47 | 2.26 | LOC100736704 |
| gene25910 | -1.794641736 | 1.28E-07 | 5.7 | 4.34 | 5.25 | 7.18 | 17.19 | 13.92 | 7.18 | 3.11 | 3.22 | 2.71 | 3.81 | 4.16 | ENOSF1 |
| gene25911 | -1.06885034 | 0.002296192 | 9 | 14.19 | 11.44 | 11.68 | 11.04 | 14.21 | 8.26 | 5.82 | 7.28 | 8.74 | 5.34 | 3.23 | TYMS |
| gene2594 | 1.67325053 | 0.004836139 | 0.21 | 0.25 | 0.64 | 0.16 | 0.15 | 0.04 | 0.45 | 0.48 | 0.79 | 0.57 | 0.3 | 0.34 | LOC100737751 |
| gene25951 | -1.909724736 | 7.02E-09 | 24.61 | 12.22 | 24.23 | 33.2 | 72.15 | 31.74 | 27.95 | 10.5 | 13.71 | 13.23 | 12.01 | 10.59 |  |
| gene25995 | -2.454816497 | 5.43E-06 | 1.09 | 1.31 | 1.26 | 1.92 | 1.36 | 2.62 | 1.14 | 0.72 | 0.75 | 0.35 | 0.1 | 0.63 | LOC100627210 |
| gene25996 | -1.947963701 | 6.58E-06 | 0.9 | 0.89 | 1.76 | 1.14 | 1.64 | 2.24 | 0.34 | 0.81 | 1.02 | 0.11 | 0.31 | 0.79 | LOC100517370 |
| gene26010 | -1.182181619 | 0.007815782 | 3.5 | 4.87 | 2.84 | 7.31 | 5.6 | 7.58 | 5.63 | 0.81 | 3.4 | 1.9 | 2.15 | 4.45 | LOC100620631 |
| gene26011 | 3.330656345 | 5.81E-13 | 1.11 | 1.02 | 0.99 | 0.65 | 2.35 | 0.8 | 1.34 | 41.83 | 0.98 | 3.93 | 32.8 | 2.12 | LOC404703 |
| gene26012 | 2.176522275 | 1.69E-05 | 0.73 | 0.45 | 0 | 0.84 | 1.16 | 1.49 | 0.94 | 6.41 | 2.79 | 0.24 | 8.4 | 5.19 | SPAG11 |
| gene26014 | -5.097942464 | 0.000550739 | 0.27 | 0.75 | 1.08 | 0.94 | 0.78 | 0.78 | 0.98 | 1.91 | 0.48 | 0 | 0 | 0 | LOC692191 |
| gene26026 | -2.682228468 | 1.74E-15 | 297.89 | 520.63 | 827.55 | 721.23 | 992.63 | 2118.1 | 1497.5 | 54.32 | 83.89 | 147.27 | 320.02 | 96.51 | LOC100738609 |
| gene26047 | -1.565163721 | 0.000203374 | 3.75 | 3.33 | 8.39 | 9.69 | 9.46 | 11.06 | 3.29 | 3.15 | 4.27 | 2.43 | 4.44 | 2.96 | LOC100624307 |
| gene26071 | -2.086306455 | 0.002960981 | 1.27 | 0.15 | 0 | 3.72 | 0.15 | 0.55 | 0.41 | 0.13 | 0.67 | 1.03 | 0 | 0 | CYP3A39 |
| gene26091 | 1.706625698 | 0.002841191 | 0.18 | 0.06 | 0.12 | 0.43 | 0.27 | 0 | 0.55 | 1.06 | 0.4 | 1.27 | 0.65 | 0.53 | MCT7 |
| gene2610 | -1.521107414 | 7.01E-05 | 0.99 | 1.23 | 1.7 | 1.51 | 1.67 | 1.61 | 0.92 | 1.13 | 1.18 | 0.99 | 0.37 | 0.32 | NAALADL1 |
| gene26102 | -2.759713585 | 1.16E-09 | 12.15 | 0.62 | 1.49 | 17.4 | 1.53 | 1.03 | 3.45 | 1.71 | 1.61 | 0.66 | 0.64 | 1.46 | CYP3A39 |
| gene26106 | -1.914857734 | 0.002533349 | 0.1 | 0.3 | 0.17 | 0.38 | 0.62 | 0.46 | 0.21 | 0.07 | 0.11 | 0.26 | 0.1 | 0 | CKM |
| gene26111 | -1.192269826 | 0.00021127 | 1209.3 | 1222.4 | 829.91 | 983.92 | 1133.5 | 1056.7 | 1483.2 | 330.19 | 908.41 | 461.63 | 413.32 | 470.75 | LOC100622549 |
| gene26118 | 1.130254583 | 0.000695585 | 8.02 | 10.42 | 16.56 | 6.17 | 5.37 | 17.69 | 17.83 | 9.03 | 7.97 | 8.29 | 11.72 | 39.67 | LOC100623310 |
| gene26128 | 1.122240894 | 0.000507003 | 39.57 | 63.09 | 35.3 | 17.14 | 21.56 | 21.08 | 27.11 | 23.61 | 42.56 | 33.08 | 38.62 | 53.54 | LDLR |
| gene26134 | -1.063351903 | 0.001555672 | 4.3 | 3.52 | 2.99 | 3.84 | 4.37 | 4.27 | 3.41 | 2.21 | 2.95 | 1.93 | 2.25 | 1.62 | LOC100625123 |
| gene2614 | 1.161791309 | 0.003324172 | 1.4 | 3.38 | 1.69 | 1.84 | 0.89 | 0.91 | 1.72 | 2 | 2.58 | 3.98 | 0.72 | 3.2 | LOC100515875 |
| gene26140 | -1.786028697 | 3.42E-05 | 10.6 | 6.83 | 7.96 | 5.5 | 4.01 | 3.04 | 6.05 | 2.21 | 3.83 | 1.45 | 0.38 | 1.62 | LOC100625729 |
| gene2616 | 4.371893681 | 7.56E-09 | 0.85 | 5.49 | 0.39 | 0.18 | 0.19 | 0 | 0 | 1.06 | 0 | 5.2 | 0.02 | 0.99 | LOC100516058 |
| gene26178 | 1.038815513 | 0.00136383 | 139.36 | 184.31 | 90.64 | 50.16 | 60.7 | 62.8 | 71.13 | 99.77 | 133.78 | 103.68 | 120.07 | 119.94 | LDLR |
| gene26188 | 1.567567234 | 1.30E-06 | 13133 | 1662 | 2459.9 | 1399.5 | 2034.1 | 1447.8 | 1196.5 | 2906 | 1800.5 | 8226.3 | 4202.7 | 1884.1 | HSP70.2 |
| gene26189 | 1.24652161 | 0.000371107 | 5.15 | 1.08 | 1.7 | 1.27 | 1.32 | 1.42 | 1.85 | 1.51 | 1.91 | 5.96 | 1.59 | 1.92 | HSPA1L |
| gene26190 | -1.368853456 | 7.16E-05 | 2.43 | 2.06 | 3.18 | 3.07 | 5.22 | 4.22 | 3.79 | 1.09 | 2.17 | 1.24 | 1.47 | 1.95 | ALDH1L1 |
| gene26201 | 1.240675016 | 0.000135651 | 30.82 | 26.01 | 15.88 | 20.56 | 14.77 | 24.34 | 14.3 | 59.04 | 47.05 | 37.13 | 51.68 | 45.71 | LOC100623249 |
| gene26204 | -1.310229172 | 0.005682717 | 1.08 | 1.11 | 1.33 | 1.95 | 1.55 | 1.13 | 0.19 | 0.92 | 0.64 | 1.18 | 0.33 | 0.37 | ITGB1BP2 |
| gene26207 | 1.200194244 | 0.008762792 | 1.09 | 1.15 | 2.1 | 0.59 | 0.73 | 0.84 | 0.78 | 1.13 | 1.06 | 2.3 | 1.45 | 1.15 | LOC100624064 |
| gene26223 | 1.163194322 | 0.000285965 | 1039.5 | 1301.6 | 1354.6 | 1409.2 | 1183.7 | 1604.2 | 1308.7 | 3509.2 | 3103.9 | 2131.1 | 3578.6 | 3258.5 |  |
| gene26226 | 1.705402227 | 1.57E-07 | 10296 | 11372 | 13168 | 10469 | 9951.9 | 12755 | 12935 | 24966 | 30323 | 23224 | 35128 | 45016 |  |
| gene26227 | 1.367462576 | 2.19E-05 | 3067.6 | 3866.7 | 3644.2 | 3752.2 | 3462.3 | 3892.3 | 3632.4 | 9957.2 | 7618.4 | 7635.4 | 10539 | 9327.5 |  |
| gene26230 | 1.087623777 | 0.000689344 | 429.55 | 616.73 | 765.83 | 643.91 | 612.16 | 860.32 | 357.18 | 1187.9 | 879.98 | 1144.9 | 1230.7 | 1934.1 |  |
| gene26231 | 1.07643976 | 0.000769902 | 1103.3 | 1526 | 1649.3 | 1631.3 | 1451.9 | 1865.2 | 1393.5 | 3851.4 | 3080.2 | 2728 | 4016.4 | 3243.6 |  |
| gene264 | -1.094383732 | 0.001152055 | 6.37 | 4.96 | 8.16 | 15.48 | 10.73 | 11.53 | 2.67 | 12.19 | 10.11 | 9.8 | 3.49 | 4.23 | LOC100623327 |
| gene2660 | -1.215329896 | 0.000818784 | 1.36 | 1.69 | 0.88 | 2.23 | 1.84 | 2.48 | 1.45 | 0.41 | 0.82 | 1 | 0.88 | 0.84 | LOC100511737 |
| gene2667 | 3.486552417 | 1.16E-19 | 3.98 | 13.7 | 6.4 | 0.61 | 0.19 | 1.23 | 0.09 | 1.11 | 2.1 | 4.8 | 5.55 | 10.27 | LOC100737802 |
| gene2669 | -4.530640976 | 2.91E-23 | 0.66 | 1.3 | 0.24 | 0.11 | 0.16 | 12.45 | 0.52 | 0.12 | 0.16 | 0.07 | 0.23 | 0.2 | LOC100737875 |
| gene2705 | -2.507504066 | 4.12E-08 | 0 | 0.25 | 2.6 | 5.32 | 0.74 | 16.16 | 0.11 | 0.28 | 0.16 | 1.13 | 0 | 2.38 | PHEROC |
| gene2724 | 1.060538711 | 0.002448663 | 5.14 | 6.8 | 9.06 | 6.47 | 6.59 | 4.53 | 3.79 | 12.47 | 4.27 | 13.48 | 14.99 | 7.31 | LOC100519467 |
| gene2734 | -1.158108041 | 0.000499578 | 9.7 | 5.08 | 13.8 | 24.02 | 10.24 | 4.73 | 2.97 | 16.86 | 8.35 | 4.76 | 6.39 | 5.79 | LOC100524300 |
| gene2735 | -1.178829095 | 0.008400008 | 0.59 | 0.4 | 2.78 | 2.47 | 0.71 | 0.53 | 0.08 | 2.43 | 1.61 | 0.7 | 0.63 | 0.3 | LOC100524479 |
| gene2769 | 1.476395901 | 0.001235689 | 0.35 | 0.33 | 0.24 | 0.79 | 0.54 | 0.6 | 1.08 | 1.28 | 0.45 | 2 | 1.35 | 2.05 | MS4A2 |
| gene2774 | -1.539802583 | 0.000126239 | 16.22 | 7 | 14.06 | 22.48 | 10.11 | 11.22 | 20.16 | 4.76 | 0.76 | 4.3 | 3.93 | 6.29 | LOC100621891 |
| gene2813 | -2.721613999 | 1.10E-09 | 1.14 | 2.57 | 2.97 | 3.04 | 2.6 | 3.49 | 0.98 | 0.79 | 0.18 | 0.62 | 0.53 | 0.28 | LOC100627035 |
| gene2818 | -1.372615955 | 0.002459931 | 0.51 | 0.73 | 1.3 | 0.7 | 1.72 | 2.27 | 0.17 | 0.22 | 0.15 | 0.88 | 0.81 | 0.06 | LOC100517803 |
| gene2849 | 4.962390789 | 0.001753584 | 0 | 0.02 | 0.05 | 0 | 0 | 0 | 0.23 | 0 | 0.18 | 0.23 | 0 | 0.3 | LOC100513457 |
| gene2871 | 1.074088363 | 0.002792988 |  |  |  |  |  |  |  |  |  |  |  |  |  |
| gene2894 | -3.845362941 | 5.94E-10 |  |  |  |  |  |  |  |  |  |  |  |  |  |
| gene2927 | 3.198466807 | 0.000179505 |  |  |  |  |  |  |  |  |  |  |  |  |  |
| gene2968 | 2.523632667 | 6.64E-10 | 2.92 | 3.71 | 4.84 | 3.71 | 4.2 | 2.81 | 7.02 | 25.45 | 9.35 | 30.09 | 25.76 | 5.91 | LOC100525679 |
| gene3003 | -1.47295661 | 6.63E-06 | 8.49 | 12.62 | 13.84 | 16.93 | 49.08 | 29.27 | 35.6 | 8.64 | 9.41 | 9.07 | 12.21 | 12.08 | CREB3L1 |
| gene3012 | -1.787021267 | 5.61E-05 | 0.9 | 0.7 | 0.67 | 1.31 | 2.08 | 1.47 | 0.51 | 0.83 | 0.15 | 0.4 | 0.57 | 0.43 | LOC100623533 |
| gene3035 | 1.037485293 | 0.009646915 | 0.29 | 0.89 | 0.05 | 0.26 | 0.39 | 0.31 | 0.17 | 0.39 | 0.4 | 0.75 | 0.38 | 0.8 | RAG-1 |
| gene3039 | -1.055201654 | 0.004334833 | 3 | 0.95 | 0.99 | 4.15 | 2.9 | 2.27 | 1.15 | 3.23 | 2.47 | 1.66 | 1.64 | 1.12 | LOC100524421 |
| gene3044 | -1.993080024 | 2.99E-06 | 4.59 | 3.27 | 9.07 | 14.02 | 9.82 | 8.24 | 3.75 | 1.73 | 5.38 | 0.34 | 1.07 | 6.35 | LOC100626862 |
| gene3045 | -1.460065678 | 8.89E-06 | 6.91 | 6.67 | 10.92 | 19.86 | 16.17 | 12.19 | 8.09 | 4.22 | 4.29 | 6.14 | 3.32 | 7.61 | PAMR1 |
| gene3046 | -1.449792921 | 0.008110732 | 0.56 | 0.35 | 0.33 | 0.46 | 0.36 | 0.55 | 0.56 | 0.4 | 0.31 | 0.25 | 0.11 | 0.1 | LOC100627068 |
| gene3051 | 1.01330121 | 0.001610758 | 104.08 | 134.71 | 123.61 | 45.81 | 44.11 | 77.21 | 37.48 | 89.98 | 80.93 | 99.23 | 94.63 | 129.06 | LOC100526094 |
| gene3092 | -1.270698228 | 0.000997374 | 1.34 | 1.46 | 3.5 | 6.34 | 2.07 | 2.83 | 2.12 | 2.46 | 2.18 | 1.28 | 1.5 | 1.78 | BDNF |
| gene3095 | -1.067209162 | 0.001390809 | 34.47 | 38.69 | 36.09 | 55.8 | 61.93 | 43.53 | 45.53 | 47.06 | 37.87 | 28.61 | 22.56 | 24.24 | LGR4 |
| gene3101 | -1.046669405 | 0.002844124 | 1.43 | 1.12 | 1.93 | 1.83 | 1.64 | 2.61 | 0.61 | 2.25 | 1.04 | 1.51 | 0.72 | 0.65 | ANO3 |
| gene3102 | -1.22819177 | 0.003494548 | 4.69 | 1.55 | 2.59 | 4.38 | 3.26 | 1.91 | 0.61 | 0.2 | 1.76 | 1.95 | 0.8 | 1.27 | LOC100520176 |
| gene3110 | 1.644455594 | 7.66E-05 | 1.61 | 0.7 | 0.63 | 0.8 | 0.93 | 0.55 | 1.07 | 2.19 | 1.91 | 1.85 | 2.1 | 3.12 | LOC100520718 |
| gene3126 | 1.68486831 | 0.002364822 | 0.51 | 0.13 | 0.93 | 0.08 | 0.22 | 0.14 | 0.09 | 0.44 | 0.2 | 0.15 | 0.55 | 0.64 | PTPN5 |
| gene3128 | -1.320394045 | 4.30E-05 | 146.61 | 102.52 | 164.99 | 140.51 | 442.6 | 175.66 | 103.88 | 66.27 | 89.45 | 80.18 | 103.17 | 113.45 | LOC100523718 |
| gene3130 | -1.371269583 | 0.004884258 | 2.42 | 4.59 | 5.4 | 5.97 | 9.18 | 2.42 | 3.64 | 4.92 | 1.47 | 1.03 | 2.79 | 2.89 | LOC100523888 |
| gene3138 | -1.582645579 | 0.002588055 | 0.1 | 0.12 | 1.02 | 3.94 | 0 | 2.21 | 4.16 | 0 | 0 | 1.25 | 0.6 | 0.14 | LOC100525856 |
| gene3145 | 3.350659571 | 0.000339779 | 0.03 | 0.07 | 0.04 | 0.03 | 0 | 0.04 | 0.07 | 0.33 | 0.25 | 0.61 | 0.14 | 0.12 | LOC100513908 |
| gene3149 | 1.184654392 | 0.009977259 | 0.14 | 0.1 | 0.1 | 0.12 | 0.12 | 0.1 | 0.22 | 0.32 | 0.31 | 0.12 | 0.29 | 0.32 | ABCC8 |
| gene3197 | 1.338087477 | 9.67E-05 | 7.51 | 5.24 | 6.15 | 4.15 | 5.74 | 5.79 | 5.57 | 9.33 | 8.23 | 12.55 | 20.04 | 5.34 | LOC100525978 |
| gene3205 | -1.174497156 | 0.003734191 |  |  |  |  |  |  |  |  |  |  |  |  |  |
| gene3244 | 3.180335428 | 9.15E-15 | 0.17 | 2.13 | 0.3 | 0.49 | 0.16 | 0.17 | 0.84 | 0.18 | 2.07 | 1.94 | 0.98 | 4.05 | LOC100525099 |
| gene328 | -1.403930751 | 0.002468 | 0.45 | 0.13 | 0.18 | 0.58 | 0.67 | 0.22 | 0.14 | 0.7 | 0.02 | 0.2 | 0.32 | 0.02 | COL9A1 |
| gene3304 | 2.623731229 | 0.000520691 |  |  |  |  |  |  |  |  |  |  |  |  |  |
| gene3310 | 5.631198141 | 5.97E-05 |  |  |  |  |  |  |  |  |  |  |  |  |  |
| gene3313 | -1.762697233 | 0.000555632 | 0.44 | 0.57 | 0.5 | 2.6 | 0.87 | 1.62 | 0.73 | 1.64 | 0.6 | 0 | 0.6 | 0.76 | LOC100515190 |
| gene3321 | 1.612679554 | 0.00646564 | 0.56 | 0.33 | 0.25 | 0.18 | 0.23 | 0.29 | 0.16 | 0.37 | 0.27 | 0.82 | 0.72 | 0.58 | LOC100514276 |
| gene3323 | -1.064890551 | 0.002975583 | 1.51 | 2.76 | 2.36 | 3.55 | 4.49 | 2.55 | 3.01 | 4.16 | 2.21 | 2.63 | 1.22 | 1.23 | LOC100514093 |
| gene3336 | -1.912556168 | 1.10E-07 | 0.73 | 0.85 | 1.04 | 5.5 | 2.31 | 1.21 | 2.05 | 0.47 | 2.08 | 0.46 | 0.4 | 1.44 | COMP |
| gene3340 | -1.193005759 | 0.001611949 | 1.13 | 3.61 | 2.89 | 3.86 | 2.08 | 3.67 | 3.15 | 1.09 | 1.68 | 1.48 | 1.6 | 0.96 | TMEM59L |
| gene3347 | -1.166843582 | 0.000352599 | 6.08 | 23.9 | 21 | 40.19 | 20.17 | 26.12 | 29.8 | 9.53 | 9.68 | 11.34 | 12.89 | 12.8 | CRLF1 |
| gene3362 | -1.617614965 | 0.003202536 | 0.16 | 0.18 | 0.1 | 0.42 | 0.7 | 0 | 0.25 | 0.58 | 0.24 | 0.06 | 0.12 | 0.17 | SLC5A5 |
| gene3385 | -2.736323745 | 1.39E-06 | 0.93 | 1.03 | 0.44 | 1.17 | 0.99 | 0.77 | 0.08 | 0.1 | 0.29 | 0.38 | 0 | 0.05 | LOC100514949 |
| gene3401 | -3.126202214 | 7.82E-10 | 0.21 | 0.79 | 0.84 | 3.12 | 0.4 | 1.6 | 0.2 | 0.34 | 0.12 | 0.11 | 0.22 | 0.2 | HSH2D |
| gene3429 | -1.041397262 | 0.00412229 | 2.75 | 2.04 | 1.76 | 4.64 | 3.2 | 3.67 | 3.38 | 1.89 | 1.66 | 1.81 | 1.38 | 2.29 | LOC100625866 |
| gene3438 | 2.782317919 | 0.000532308 | 0 | 0.11 | 0 | 0.08 | 0 | 0 | 0.18 | 0.28 | 0 | 0.44 | 0.11 | 0.1 | LOC100518296 |
| gene35 | -1.266617899 | 0.003826756 | 0.49 | 0.36 | 0.52 | 0.74 | 0.64 | 1.1 | 0.49 | 0.36 | 0.07 | 0.59 | 0.2 | 0.18 |  |
| gene3553 | 1.094432925 | 0.000731484 | 138.75 | 22.39 | 30.52 | 20.18 | 20.51 | 17.55 | 16.16 | 15.34 | 16.77 | 91.73 | 20.39 | 12.4 | DNAJB1 |
| gene3557 | -2.102398516 | 1.78E-05 | 1.38 | 0.9 | 1.86 | 2.7 | 3.34 | 3.21 | 1.85 | 0.6 | 1.73 | 0.8 | 0.52 | 0.77 | LOC100624148 |
| gene3559 | -1.825107127 | 2.00E-05 | 0.81 | 2.43 | 1.08 | 1.24 | 2.04 | 6.82 | 2.82 | 1.5 | 3.79 | 1.32 | 0.79 | 0.53 | ASF1B |
| gene3564 | 1.253411046 | 0.000171976 | 6.24 | 2.79 | 2.62 | 3.89 | 1.92 | 3.48 | 6.54 | 22.33 | 4.29 | 2.57 | 14.52 | 3.67 | PALM3 |
| gene3603 | 1.61633414 | 6.57E-07 | 54.75 | 78.43 | 82.58 | 45.99 | 49.27 | 51.18 | 105.82 | 75.45 | 88.28 | 42.61 | 94.7 | 290.41 |  |
| gene3665 | -2.474092753 | 0.005219397 | 0.17 | 0.15 | 0.22 | 0.29 | 0.14 | 0.42 | 0.37 | 0 | 0.07 | 0 | 0 | 0.12 | LOC100524668 |
| gene3679 | -4.627347118 | 0.005776466 | 0.15 | 0 | 0 | 0.18 | 0.52 | 0.22 | 0 | 0.24 | 0 | 0 | 0 | 0 | LOC100739456 |
| gene37 | -1.014490127 | 0.00397551 | 3.39 | 3.08 | 2.92 | 14.89 | 6.98 | 6.32 | 6.97 | 9.3 | 11.17 | 2.96 | 7.41 | 3.14 | PARK2 |
| gene3700 | -1.670049366 | 0.005097461 | 0.49 | 0.21 | 0.61 | 0.58 | 0.28 | 1.11 | 0.54 | 0.44 | 0.25 | 0.11 | 0.22 | 0.2 | LOC100519130 |
| gene3767 | 1.355024523 | 3.48E-05 | 39.36 | 56.41 | 32.86 | 17.54 | 22.34 | 22.47 | 31.08 | 30.41 | 53.46 | 36.23 | 52.75 | 64.01 | LDLR |
| gene3785 | -2.044784175 | 5.06E-09 | 12.49 | 21.81 | 28.22 | 24.66 | 42.97 | 80.72 | 79.05 | 2.99 | 4.24 | 6.49 | 22.38 | 5.1 | UF |
| gene3789 | -1.334985212 | 0.006092971 | 0.31 | 0.38 | 0.28 | 0.44 | 0.66 | 0.65 | 0.38 | 0.15 | 0.12 | 0.19 | 0.07 | 0.4 | ZNF709 |
| gene3803 | 1.095508816 | 0.000724169 | 18.89 | 33.57 | 29.84 | 15.34 | 14.75 | 18.66 | 14.07 | 31.06 | 21.95 | 22.89 | 32.86 | 43.77 | ANGPTL4 |
| gene3804 | -1.006771672 | 0.002668557 | 2.03 | 3.19 | 4.81 | 4.02 | 5.08 | 4.94 | 1.93 | 2.45 | 1.76 | 2.77 | 1.67 | 2.35 | KANK3 |
| gene3807 | -1.13354326 | 0.000575989 | 13.85 | 12.1 | 18.77 | 16.93 | 22.06 | 16.7 | 14.86 | 6.04 | 11.54 | 7.4 | 7.62 | 9.62 | LASS4 |
| gene3815 | -1.918008385 | 0.008227848 | 0.75 | 0.53 | 0.4 | 1.07 | 1.35 | 2.64 | 0.49 | 0.76 | 0.72 | 0.98 | 0 | 0.44 | LOC100520948 |
| gene382 | 1.439204703 | 0.002320483 | 0.44 | 0.08 | 0.07 | 0.03 | 0.72 | 0.08 | 0.21 | 0.54 | 0.19 | 1.37 | 0.6 | 0.43 | GABRR1 |
| gene3864 | 1.172369166 | 0.002679514 | 4.64 | 2.17 | 1.17 | 1.63 | 1.21 | 1.28 | 0.84 | 1.95 | 4.52 | 2.83 | 2.29 | 3.86 | LOC100736831 |
| gene3872 | -1.377800686 | 0.000373518 | 5.85 | 6.89 | 7.8 | 8.41 | 10.2 | 14.74 | 5.83 | 1.91 | 4.06 | 3.27 | 3.49 | 5.48 | LOC100518058 |
| gene3877 | -2.296455773 | 0.00011998 | 0.62 | 1.08 | 0.83 | 1.96 | 2.33 | 0.77 | 0.86 | 1.4 | 0.47 | 0.44 | 0 | 0.51 | LOC100513645 |
| gene3880 | -1.620813996 | 0.006576661 | 1.03 | 0.79 | 1.58 | 1.22 | 0.7 | 2.08 | 1.94 | 0.15 | 0.5 | 0.48 | 0.7 | 0 | LOC100514213 |
| gene3901 | 1.290602517 | 7.10E-05 | 4.58 | 4.29 | 4.22 | 2.24 | 4.7 | 4.67 | 2.93 | 2.78 | 3.95 | 9.12 | 5.72 | 12.7 | PTPRS |
| gene3923 | 1.768242762 | 0.00010805 | 0.35 | 0.31 | 0.36 | 0.48 | 0.16 | 0.3 | 0.4 | 1.36 | 0.28 | 1.91 | 0.7 | 0.46 | LOC100522979 |
| gene3941 | 2.608591462 | 3.70E-05 | 0.59 | 0.87 | 0.94 | 0.42 | 0.14 | 0.51 | 0.95 | 3.71 | 0 | 0.38 | 3.35 | 2.54 | LOC100736989 |
| gene3942 | 2.487684181 | 0.000481506 | 0 | 0.77 | 0.38 | 0.23 | 0.06 | 0 | 1.71 | 0.25 | 0.17 | 0.16 | 1.37 | 0.28 | LOC100737370 |
| gene3955 | 1.106779626 | 0.001470516 | 3.45 | 4.55 | 3.33 | 1.49 | 1.85 | 2.75 | 2.32 | 3.33 | 2.88 | 4.25 | 4.35 | 3.89 | LOC100516355 |
| gene3982 | 1.414112329 | 1.48E-05 | 5.24 | 6.26 | 5.84 | 4.25 | 4 | 3.35 | 6.04 | 3.88 | 9.44 | 5.26 | 10.45 | 13.85 |  |
| gene3992 | 3.750681553 | 1.74E-25 | 5.22 | 6.37 | 2.26 | 4.46 | 4.3 | 4.39 | 52.47 | 87.97 | 44.26 | 57.75 | 71.47 | 41.8 | LOC100512947 |
| gene4006 | -1.231754685 | 0.00029302 | 0.91 | 0.84 | 1.48 | 1.3 | 2.27 | 1.36 | 0.85 | 0.86 | 0.87 | 0.79 | 0.69 | 0.59 | ABCA7 |
| gene4019 | -1.682578876 | 0.003753698 | 0.75 | 0.68 | 1.25 | 1 | 1.61 | 0.64 | 0.71 | 0.37 | 0.98 | 0.45 | 0 | 0.5 | LOC100626712 |
| gene402 | -1.256082809 | 0.000899181 | 0.67 | 0.9 | 0.71 | 1.06 | 1.27 | 1.18 | 0.54 | 2.89 | 0.44 | 0.64 | 0.69 | 0.14 | EPHA7 |
| gene4055 | -1.242743278 | 0.000892275 | 0.36 | 0.46 | 1.06 | 0.47 | 0.92 | 1.12 | 0.64 | 0.33 | 0.17 | 0.54 | 0.24 | 0.27 | LOC100518179 |
| gene410 | -1.524327013 | 4.45E-05 | 3.25 | 0.76 | 1.82 | 2.48 | 2.22 | 4.86 | 3.17 | 0.86 | 1.34 | 1.07 | 0.31 | 1.83 | GPR63 |
| gene4119 | -1.662933172 | 6.73E-06 | 4.43 | 6.47 | 5.11 | 6.33 | 5.12 | 8.09 | 4.14 | 2.64 | 2.7 | 2.02 | 1.95 | 2.06 | LOC100624464 |
| gene4164 | -1.768823232 | 0.001494881 | 0.3 | 0.69 | 0.47 | 1.6 | 0.82 | 1.26 | 0.78 | 0.56 | 0.62 | 0.19 | 0.37 | 0.57 | LOC100521776 |
| gene4166 | 1.089565687 | 0.006791563 |  |  |  |  |  |  |  |  |  |  |  |  |  |
| gene418 | -1.781514843 | 0.000261371 | 0.64 | 0.68 | 0.43 | 1.04 | 1.22 | 1.17 | 0.71 | 1.07 | 1.16 | 0.45 | 0.38 | 0.2 | LOC100156103 |
| gene4181 | 1.987517583 | 1.26E-08 | 8.63 | 10.54 | 7.45 | 3.24 | 4.28 | 4.34 | 2.15 | 8.17 | 9.43 | 17.16 | 10.67 | 17.76 | HOMER1 |
| gene4182 | 2.007908949 | 8.24E-05 | 3 | 4 | 2.66 | 0.12 | 0.73 | 1.51 | 0.82 | 1.36 | 2.4 | 3.95 | 0.64 | 3.99 | HOMER1 |
| gene4187 | -2.053741728 | 3.90E-05 | 0.07 | 0.42 | 0.41 | 0.67 | 0.27 | 0.38 | 0.42 | 0.09 | 0.2 | 0.16 | 0.04 | 0.12 | LOC100513005 |
| gene4189 | -1.577579708 | 0.000243898 | 0.04 | 0.43 | 0.53 | 0.66 | 0.54 | 0.68 | 0.34 | 0.04 | 0.19 | 0.26 | 0.13 | 0.23 |  |
| gene4190 | 1.177826947 | 0.00272952 | 2.36 | 5.3 | 3.27 | 1.55 | 1.63 | 0.85 | 1.87 | 1.51 | 1.87 | 3.83 | 2.7 | 2.38 | LOC100737687 |
| gene4201 | 4.619030592 | 9.10E-08 | 0.13 | 0.08 | 0.34 | 0.07 | 0 | 0 | 0.18 | 0.44 | 0.06 | 0.84 | 0.2 | 0.37 | ACOT12 |
| gene426 | 4.974503363 | 0.001753584 |  |  |  |  |  |  |  |  |  |  |  |  |  |
| gene4273 | -1.140370472 | 0.000472869 | 14.41 | 6.75 | 8.78 | 24.08 | 10.37 | 11.14 | 5.03 | 17.3 | 13.43 | 10.73 | 5.37 | 4.22 | LOC100737557 |
| gene4274 | -1.41695937 | 0.000390018 | 1.37 | 0.41 | 0.25 | 1.52 | 1.18 | 0.66 | 0.19 | 0.13 | 0.69 | 0.98 | 0.2 | 0.1 | SLCO6A1 |
| gene4326 | -1.179075809 | 0.00032889 | 21.61 | 22.95 | 34.12 | 17.43 | 12.79 | 100.21 | 13.63 | 6.63 | 9.06 | 18.11 | 17.98 | 17.98 | CDO1 |
| gene433 | -1.153071274 | 0.009229164 | 0.34 | 0.32 | 0.39 | 0.67 | 0.58 | 0.91 | 0.36 | 0.42 | 0.3 | 0.34 | 0.37 | 0.21 | GRIK5 |
| gene4350 | -2.24721002 | 2.39E-10 | 2.36 | 1.52 | 6.14 | 5.52 | 2.11 | 14.69 | 1.41 | 1.77 | 0.93 | 1.51 | 0.71 | 2.26 | SNCAIP |
| gene4381 | 1.165363791 | 0.000327267 | 6.26 | 15.5 | 7.34 | 5.19 | 4.17 | 8.18 | 7.46 | 8.74 | 10.75 | 12.69 | 12.99 | 11.93 | SLC12A2 |
| gene4384 | -1.380592955 | 1.97E-05 | 23.1 | 13.6 | 43.53 | 30.42 | 90.37 | 58.5 | 24.14 | 16.25 | 21.16 | 12.78 | 19.53 | 34.2 |  |
| gene4413 | -1.107262105 | 0.005247107 | 0.59 | 0.44 | 1.02 | 0.76 | 0.68 | 1.13 | 0.44 | 0.41 | 0.61 | 0.57 | 0.25 | 0.36 | SHROOM1 |
| gene4432 | -2.318113801 | 1.12E-05 | 2.96 | 2.37 | 1.59 | 7.34 | 2.24 | 2.51 | 1.56 | 0.66 | 0.7 | 0.35 | 1.23 | 1 | LOC100737935 |
| gene4443 | 1.5967238 | 9.05E-07 | 462.77 | 1168 | 525.78 | 191.87 | 230.21 | 178.53 | 235.65 | 725.24 | 866.07 | 835.63 | 460.03 | 486.47 | CXCL14 |
| gene4444 | -1.965131031 | 4.38E-08 | 3.31 | 1.27 | 1.5 | 5.12 | 12.56 | 6.66 | 1.34 | 5.1 | 3.45 | 2.1 | 1.4 | 2.72 | LOC100518721 |
| gene4455 | 2.904743991 | 1.60E-17 | 10 | 10.05 | 4.92 | 16.62 | 14.58 | 12.66 | 147.27 | 157.97 | 73.22 | 186.63 | 91.94 | 44.97 | KLHL3 |
| gene4463 | 2.576729503 | 0.000224061 | 0 | 0.09 | 0.05 | 0 | 0.12 | 0.1 | 0.12 | 0.3 | 0 | 0.41 | 0.51 | 0.46 | WNT8A |
| gene4467 | -1.046979207 | 0.001937551 | 1.99 | 5.43 | 1.73 | 0.63 | 4.7 | 5.74 | 3.85 | 2.02 | 8.17 | 2.22 | 1.9 | 1.07 | KIF20A |
| gene4477 | 2.367399048 | 1.25E-12 | 60.33 | 88.45 | 78.22 | 24.6 | 36.37 | 24.07 | 79.67 | 76.67 | 94.25 | 81.23 | 79.79 | 262.87 | EGR1 |
| gene449 | -1.802108369 | 0.002958695 | 0.89 | 1.11 | 0.85 | 2.72 | 2.97 | 0.82 | 1.06 | 3.04 | 2.19 | 0 | 1.21 | 0.69 | PDSS2 |
| gene4493 | -1.413290521 | 0.00014407 | 2.54 | 2.48 | 2.7 | 3.34 | 5.17 | 2.09 | 2.55 | 2.24 | 1.05 | 2.15 | 0.62 | 1.24 | TMEM173 |
| gene4510 | 1.797709525 | 5.49E-08 | 21.68 | 39.3 | 44.02 | 23.42 | 23.68 | 16.96 | 30.21 | 39.11 | 50.3 | 37.28 | 60.3 | 116.62 | HBEGF |
| gene4527 | -3.785591171 | 6.82E-05 |  |  |  |  |  |  |  |  |  |  |  |  |  |
| gene4536 | -1.095749244 | 0.006636563 | 0.66 | 0.83 | 0.3 | 0.34 | 1.39 | 0.42 | 0.22 | 0.36 | 0.28 | 0.39 | 0.27 | 0.32 |  |
| gene4545 | 1.365884661 | 0.000370951 | 1.28 | 2.13 | 1.73 | 0.37 | 0.78 | 1.04 | 0.55 | 1.29 | 0.98 | 2.61 | 0.57 | 2.16 | LOC100739340 |
| gene4549 | -1.408228381 | 0.005085543 |  |  |  |  |  |  |  |  |  |  |  |  |  |
| gene4557 | 2.086680684 | 4.17E-05 | 0.18 | 0.16 | 0.27 | 0.04 | 0.17 | 0.13 | 0.05 | 0.02 | 0.08 | 0.99 | 0.14 | 0.31 | LOC100523539 |
| gene4609 | -1.131118799 | 0.000632964 | 2.84 | 5.88 | 3.92 | 28.86 | 10.27 | 4.45 | 36.19 | 7.48 | 6.08 | 2.63 | 9.19 | 7.26 | PPP2R2B |
| gene4629 | -4.233829654 | 3.49E-28 | 146.73 | 94.46 | 188.18 | 112.11 | 78.46 | 139.07 | 12.51 | 11.55 | 7.18 | 8.04 | 4.67 | 4.49 | LOC100513074 |
| gene465 | -1.198886327 | 0.000303964 | 4.34 | 3.12 | 9.12 | 14.33 | 28.51 | 12.83 | 12.14 | 6.52 | 3.66 | 9.91 | 6.52 | 7.56 | PPIL6 |
| gene4679 | -2.24437091 | 8.26E-05 | 0.25 | 0.47 | 0.76 | 0.34 | 0.94 | 1.35 | 2.04 | 0 | 0.57 | 0 | 0.06 | 0.41 | LOC100520296 |
| gene475 | -1.879304264 | 5.16E-07 | 2.13 | 0.5 | 1.24 | 3.46 | 7.31 | 2.56 | 3.57 | 2.55 | 1.74 | 1.57 | 1.41 | 0.73 | LOC100736631 |
| gene476 | -1.604612637 | 2.02E-06 | 9.29 | 7.85 | 8.69 | 10.84 | 16.1 | 24.03 | 8.16 | 6.59 | 4.56 | 3.23 | 6.73 | 6.05 | DDO |
| gene478 | -1.484131 | 0.000674149 | 0.75 | 0.37 | 0.4 | 0.62 | 1.78 | 0.6 | 1.03 | 0.1 | 0.08 | 0.3 | 0.22 | 0.53 | LOC100525793 |
| gene4815 | -2.137190902 | 6.08E-05 | 2.6 | 0.84 | 1.2 | 4.7 | 2.06 | 1.3 | 0.08 | 0.79 | 0 | 0.76 | 1.11 | 0 | LOC100626247 |
| gene4816 | -1.554246948 | 2.51E-06 | 82.51 | 59.62 | 87.87 | 102.72 | 76.06 | 106.29 | 46.17 | 50.16 | 31.94 | 28.91 | 46.98 | 17.33 | LOC100519082 |
| gene4819 | -1.668961056 | 0.000701075 | 0.46 | 0.24 | 0.57 | 0.31 | 0.66 | 0.52 | 0.2 | 0.14 | 0.11 | 0.16 | 0.16 | 0.12 | ATHL1 |
| gene4820 | -1.577692996 | 0.000604062 | 0.24 | 0.43 | 0.43 | 0.54 | 0.61 | 0.28 | 0.36 | 0.07 | 0.22 | 0.07 | 0.07 | 0.32 | NLRP6 |
| gene4834 | 1.148871859 | 0.002012354 | 0.39 | 5.46 | 2.25 | 6.35 | 1.61 | 3.75 | 5.3 | 9.59 | 3.5 | 9.3 | 7.79 | 7.46 | LOC100627004 |
| gene4836 | -1.011862523 | 0.001961603 | 8.69 | 10.91 | 12.89 | 13.33 | 18.75 | 19.85 | 9.17 | 6.03 | 7.27 | 8.02 | 7.61 | 9.26 | LOC100737907 |
| gene4853 | -2.227258023 | 1.10E-10 | 8.36 | 4.38 | 3.87 | 20.89 | 9.83 | 13.87 | 12.22 | 3.96 | 3.13 | 3.66 | 3.7 | 1.91 | LOC100522669 |
| gene4855 | -1.449807983 | 0.001262131 | 0.84 | 0.62 | 1.84 | 1.57 | 1.4 | 1.71 | 0.49 | 0.68 | 1.03 | 0.57 | 0.35 | 0.69 | LOC100738733 |
| gene4886 | -3.819405616 | 4.23E-05 | 0.29 | 0.09 | 0.19 | 0.11 | 0.28 | 1.23 | 0.28 | 0.05 | 0.09 | 0.08 | 0 | 0 | LOC100624843 |
| gene4897 | -1.810804218 | 2.57E-06 | 0.44 | 1 | 2.52 | 0.96 | 2.67 | 3.51 | 0.28 | 0.47 | 0.33 | 0.76 | 0.77 | 0.5 | LOC100625519 |
| gene4911 | 2.717881807 | 1.55E-14 | 7.64 | 8.55 | 5.28 | 1.58 | 1.75 | 1.8 | 2.87 | 0.71 | 4.21 | 10.21 | 13.55 | 8.51 | LOC100521613 |
| gene494 | -1.92931173 | 4.25E-06 | 0.68 | 1.51 | 1.88 | 1.75 | 1.48 | 3.75 | 0.89 | 0.89 | 0.32 | 0.65 | 0.56 | 0.56 | WISP3 |
| gene4969 | -1.119719536 | 0.001698269 | 0.63 | 1.2 | 1.61 | 2.61 | 1.56 | 1.94 | 1.74 | 1.2 | 0.84 | 0.78 | 1.25 | 0.69 | LOC100514396 |
| gene5043 | -1.143937301 | 0.001666456 | 5.72 | 4.64 | 5.77 | 4.84 | 6.82 | 6.8 | 5.31 | 5.17 | 2.92 | 2.17 | 3.21 | 2.77 | LOC100525511 |
| gene5052 | -1.126843872 | 0.00076268 | 15.09 | 11.68 | 17.31 | 16.76 | 23.36 | 26.88 | 13.02 | 5.28 | 6.52 | 12.1 | 7.01 | 10.75 | LOC100628220 |
| gene5053 | -1.340232025 | 3.95E-05 | 13.88 | 13.78 | 17.12 | 18.19 | 26.91 | 37.25 | 10.34 | 7.07 | 5.16 | 12.16 | 9.51 | 9.74 | LOC100628118 |
| gene5081 | 1.33552295 | 6.19E-05 | 17.31 | 23.16 | 25.88 | 15.18 | 17.55 | 18.25 | 24.01 | 21.59 | 29.4 | 29.02 | 32.75 | 61.53 | LOC100738951 |
| gene5098 | -3.023844781 | 1.12E-13 | 1.25 | 1.43 | 0.92 | 2.09 | 7.28 | 2.65 | 1.62 | 1.34 | 0.94 | 0.95 | 0.14 | 0.42 | 14-Sep |
| gene5103 | -1.11013563 | 0.001247568 | 21.83 | 23.06 | 24.69 | 28.46 | 21.64 | 32.1 | 14.06 | 20.43 | 16.63 | 17.67 | 8.51 | 10.99 | LOC100519594 |
| gene5107 | 4.825585383 | 0.003168798 | 0.11 | 0.07 | 0.44 | 0 | 0 | 0 | 0.52 | 0 | 0 | 0.54 | 0.52 | 0.47 | LOC100738907 |
| gene5118 | 1.598655324 | 1.44E-06 | 7.9 | 8.22 | 2.37 | 4.52 | 7.05 | 3.98 | 22.18 | 18.52 | 7.78 | 25.19 | 13.76 | 7.69 | PRSS53 |
| gene5172 | -1.397659621 | 0.000229788 | 3.75 | 3.33 | 3.3 | 5.77 | 4.72 | 4.39 | 2.78 | 2.33 | 1.61 | 1.93 | 1.7 | 1.86 | LOC100517816 |
| gene5186 | -2.987216801 | 4.54E-17 | 8.24 | 0.99 | 9.87 | 15.02 | 52.1 | 13.34 | 32.4 | 1.39 | 0.78 | 2.94 | 5.59 | 1.43 | LOC100739420 |
| gene5192 | -2.102815635 | 2.70E-09 | 14.37 | 22.57 | 30.25 | 29.9 | 29.97 | 52.06 | 29.42 | 15.37 | 14.44 | 6.23 | 9.27 | 9.62 | LOC100739592 |
| gene5231 | 2.119475654 | 0.000350506 | 0.02 | 4.14 | 0 | 0 | 0.03 | 0.3 | 0 | 0.32 | 0.08 | 0.39 | 0.83 | 0 | ACSM2B |
| gene5244 | 1.127494964 | 0.000886856 | 1.4 | 1.77 | 1.95 | 1.64 | 1.01 | 1.55 | 1.38 | 2.2 | 1.68 | 1.37 | 2.38 | 4.92 | ABCC1 |
| gene5251 | 1.333686313 | 0.00212893 | 7.79 | 5.24 | 4.64 | 3.99 | 2.8 | 4.9 | 10.24 | 16.5 | 3.81 | 4.34 | 14.92 | 8.21 | LOC100625965 |
| gene526 | -1.443583797 | 8.61E-06 | 273.22 | 125.26 | 296.21 | 266.76 | 516.71 | 314.1 | 181.95 | 114.84 | 181.54 | 109.06 | 134.81 | 147.69 | LOC100626351 |
| gene5270 | -2.227724895 | 1.27E-06 | 2.15 | 3.53 | 3.04 | 6.32 | 5.42 | 7.57 | 4.75 | 1.55 | 4 | 1.53 | 0.42 | 1.92 | PRM2 |
| gene5319 | -1.732689244 | 0.003056262 | 0.91 | 0.56 | 2.57 | 0.68 | 2.11 | 2.96 | 1.9 | 0.64 | 1.6 | 0.66 | 0.8 | 0.29 | LOC100738369 |
| gene5344 | -1.84035094 | 0.003753698 | 0.51 | 0.4 | 0.83 | 0.57 | 0.89 | 0.58 | 1 | 0.42 | 0.57 | 0.21 | 0.33 | 0.06 | LOC100517149 |
| gene5359 | 1.115471303 | 0.000541239 | 144.71 | 192.87 | 226.87 | 111.48 | 69.98 | 91.35 | 116.02 | 184.96 | 175.85 | 170.7 | 194.38 | 202.32 | TNFRSF12A |
| gene5364 | -2.2970368 | 0.00011998 | 0.02 | 0 | 0.12 | 0.04 | 0.74 | 0.17 | 0.37 | 0.05 | 0.06 | 0.17 | 0.03 | 0 | ZSCAN10 |
| gene5381 | 1.679119215 | 2.41E-07 |  |  |  |  |  |  |  |  |  |  |  |  |  |
| gene5394 | -2.393141528 | 0.004917768 | 0.2 | 0.14 | 0.03 | 0.36 | 0.15 | 0.21 | 0.1 | 0.13 | 0.26 | 0.04 | 0 | 0.07 | LOC100739103 |
| gene5400 | -1.292976981 | 0.001298713 | 0.28 | 0.29 | 0.27 | 0.21 | 0.47 | 0.5 | 0.33 | 0.17 | 0.35 | 0.17 | 0.1 | 0.21 |  |
| gene5421 | -1.541346253 | 4.62E-05 | 0.77 | 0.86 | 1.14 | 1.23 | 2.22 | 2.56 | 1.16 | 0.63 | 0.09 | 0.78 | 0.43 | 0.86 | IGFALS |
| gene5424 | -1.260705758 | 0.003799439 | 0.43 | 0.85 | 0.47 | 0.76 | 0.97 | 1.69 | 0.89 | 0.15 | 0.62 | 0.57 | 0.37 | 0.42 | LOC100521548 |
| gene546 | 1.11326804 | 0.000531106 | 179.34 | 72.76 | 62.8 | 50.45 | 46.06 | 66.92 | 70.63 | 69.49 | 89.88 | 100.57 | 124.65 | 113.44 | LOC100156873 |
| gene5475 | -1.483341977 | 0.005134042 | 1.13 | 2.45 | 1.57 | 0.67 | 1.31 | 0.76 | 1.47 | 0.09 | 0.53 | 0.71 | 0.14 | 0.12 |  |
| gene550 | -1.145825817 | 0.002329837 | 1.62 | 1.35 | 1.53 | 3.58 | 2.75 | 1.61 | 4.52 | 1.34 | 2.61 | 1.67 | 0.99 | 0.98 | LOC100511670 |
| gene5510 | 2.003571027 | 0.002335903 | 0.5 | 0.61 | 0.52 | 0.12 | 0.46 | 0.43 | 2.1 | 2.42 | 2.44 | 2.94 | 1.11 | 0.14 | LOC100512957 |
| gene5511 | -1.223191361 | 0.000399758 | 1.05 | 1.12 | 1.84 | 4.93 | 3.02 | 2.22 | 1.39 | 3.87 | 2.01 | 1.66 | 1.29 | 1.34 | LOC100513144 |
| gene5524 | 1.231963693 | 0.002472901 | 1.53 | 6.22 | 3.18 | 1.31 | 0.32 | 1.69 | 0.3 | 2.68 | 1.79 | 2.38 | 3.16 | 1.86 | IL1A |
| gene5563 | -3.866874982 | 4.49E-10 | 0.25 | 0.15 | 0.44 | 0.77 | 2.7 | 1.15 | 0.99 | 0.39 | 0.25 | 0.15 | 0.15 | 0 | LOC100524797 |
| gene557 | -1.732857965 | 0.001030482 | 1.33 | 0.41 | 1.59 | 0.62 | 5.95 | 2.88 | 0.75 | 2.22 | 1.16 | 1.08 | 0.42 | 1.14 | LOC100511295 |
| gene5585 | -3.42626365 | 1.27E-07 | 0.09 | 0.62 | 0.67 | 1.25 | 0.03 | 0.37 | 0.11 | 0.02 | 0.38 | 0.11 | 0 | 0.03 | SLC5A7 |
| gene5603 | 1.292520368 | 6.21E-05 | 36.28 | 37.69 | 40.37 | 12.05 | 28.12 | 39.86 | 20.13 | 44.42 | 57.13 | 60.97 | 39.94 | 87.83 | SLC9A4 |
| gene5607 | 1.104019501 | 0.001261036 | 1.66 | 2.51 | 4.36 | 2.64 | 1.41 | 3.02 | 2.86 | 8.79 | 4.99 | 4.94 | 4.87 | 4.64 | IL1RL1 |
| gene5646 | -1.270701281 | 0.001148249 | 0.1 | 0.31 | 0.51 | 0.42 | 0.57 | 1.97 | 1 | 0.13 | 0.07 | 0.36 | 0.52 | 0.28 | VWA3B |
| gene5648 | -2.406792137 | 1.92E-05 | 0.12 | 0.15 | 0.33 | 0.27 | 0.83 | 0.5 | 0.47 | 0.3 | 0.25 | 0.13 | 0.13 | 0.06 | LOC100739325 |
| gene5707 | -1.221861195 | 0.002704294 | 2.6 | 5.24 | 4.42 | 4.78 | 3.99 | 6.75 | 7.03 | 7.78 | 2.72 | 0.99 | 3.69 | 1.61 | NKL |
| gene5742 | -2.199009628 | 3.47E-09 | 11.18 | 2.83 | 69.63 | 9.46 | 7.17 | 20.65 | 504.54 | 6.28 | 11.44 | 0 | 4.54 | 3.01 | LOC100520832 |
| gene5745 | -1.947113694 | 4.45E-06 | 2.49 | 1.63 | 39.44 | 4.31 | 2.57 | 6.18 | 247.11 | 2.05 | 4.19 | 0 | 0.94 | 2.19 | REG3G |
| gene5753 | 1.176493966 | 0.000305452 | 7.92 | 8.64 | 8.19 | 4.71 | 7.44 | 4.42 | 4.92 | 14.93 | 7.62 | 8.4 | 20.29 | 7.36 | HK2 |
| gene5805 | -1.210175758 | 0.000180183 | 52.97 | 14.75 | 55.69 | 13.02 | 103.61 | 36.37 | 23.45 | 5.63 | 13.78 | 13.76 | 17.29 | 33.66 | LOC100521659 |
| gene5807 | -1.98522198 | 0.000144858 |  |  |  |  |  |  |  |  |  |  |  |  |  |
| gene5816 | -1.39299537 | 0.000331345 |  |  |  |  |  |  |  |  |  |  |  |  |  |
| gene5830 | 1.049319212 | 0.001108401 | 139.51 | 48.31 | 55.46 | 47.02 | 59.12 | 99.15 | 121.83 | 67.74 | 94.23 | 44.3 | 138.81 | 217.52 | MXD1 |
| gene5898 | 1.652193201 | 0.008622279 | 0.75 | 0.79 | 0.27 | 0.08 | 0.31 | 0.59 | 0.27 | 1.28 | 0.71 | 2.1 | 0.43 | 0.49 | LOC100522433 |
| gene5941 | -1.8057584 | 1.24E-07 | 1.83 | 3.08 | 4.26 | 8.2 | 4.57 | 9.17 | 3.87 | 0.66 | 6.08 | 3.17 | 0.7 | 2.25 | GPR115 |
| gene5956 | -1.618107211 | 2.01E-05 | 1.41 | 0.47 | 0.57 | 2.39 | 0.83 | 0.85 | 1 | 0.99 | 0.89 | 0.67 | 0.43 | 0.23 | LOC100519087 |
| gene5983 | 1.965921548 | 3.66E-08 | 3.64 | 3.44 | 3.23 | 2.16 | 3.11 | 1.56 | 4.2 | 6.38 | 4.11 | 3.05 | 9.41 | 13.04 |  |
| gene5998 | 1.004783827 | 0.001675103 | 322.32 | 366.06 | 352.86 | 142.21 | 192.46 | 207.69 | 111.49 | 200.17 | 222.16 | 345.39 | 237.68 | 470.09 | EPAS1 |
| gene6019 | -2.082405567 | 0.000126665 |  |  |  |  |  |  |  |  |  |  |  |  |  |
| gene6026 | -2.318330646 | 4.20E-05 | 0.5 | 0.39 | 0.24 | 1.29 | 1.42 | 0.66 | 0.73 | 0.05 | 0.72 | 0.3 | 0.29 | 0.07 | LOC100738491 |
| gene6028 | -2.173978181 | 0.002104777 |  |  |  |  |  |  |  |  |  |  |  |  |  |
| gene603 | -1.04351864 | 0.00151228 | 11.5 | 9.84 | 13.99 | 15.26 | 16.44 | 18.19 | 13.17 | 11.12 | 9.23 | 10.7 | 6.44 | 6.48 | ACAA2 |
| gene6090 | 1.081779981 | 0.002697716 | 0.53 | 0.46 | 0.34 | 0.45 | 0.43 | 0.33 | 0.75 | 0.48 | 0.7 | 0.98 | 0.57 | 0.96 | XDH |
| gene6102 | 2.20844039 | 4.24E-08 | 0.63 | 0.18 | 0.2 | 0.19 | 0.2 | 0.08 | 0.58 | 0.9 | 0.32 | 0.67 | 1.01 | 0.51 | ALK |
| gene6103 | 2.255374777 | 8.12E-10 | 0.5 | 1.35 | 0.48 | 1.24 | 0.39 | 0.31 | 1.55 | 2.21 | 1.18 | 1.32 | 4.59 | 2.8 |  |
| gene6133 | -1.674961914 | 6.63E-05 | 0.58 | 1.38 | 1.3 | 5.55 | 2.73 | 2.15 | 2.37 | 0.48 | 1.32 | 1.35 | 1.18 | 0.74 | LOC100625689 |
| gene6136 | -1.369350679 | 2.30E-05 | 39.98 | 26.48 | 50.42 | 55.38 | 121.96 | 49.51 | 55.26 | 22.02 | 26.52 | 29.96 | 25.67 | 30.59 | LOC100522014 |
| gene6148 | 2.059264077 | 2.65E-05 | 0.22 | 0.13 | 0.03 | 0.13 | 0.23 | 0.16 | 0.45 | 0.47 | 0.66 | 1.1 | 0.35 | 0.64 | DPYSL5 |
| gene6151 | 1.630200562 | 0.00646564 | 0.28 | 0.17 | 0.19 | 0.18 | 0.22 | 0.03 | 0.07 | 0.55 | 0.26 | 0.52 | 0.44 | 0.28 |  |
| gene6165 | -1.144075389 | 0.008793287 | 3.39 | 2.96 | 3.25 | 5.18 | 4.25 | 3.57 | 2.7 | 1.51 | 1.87 | 2.77 | 1.5 | 1.52 | LOC100622021 |
| gene6169 | 2.243231857 | 3.35E-10 | 2.42 | 3.58 | 3.25 | 0.78 | 0.66 | 1.82 | 0.6 | 1.52 | 4.1 | 6.08 | 3.37 | 5.29 |  |
| gene617 | 1.042550214 | 0.001190626 | 27.27 | 34.78 | 37.59 | 28.22 | 21.79 | 24.51 | 24.51 | 41.04 | 40.02 | 44.96 | 46.61 | 56.21 | ME2 |
| gene6178 | 1.750810867 | 0.001167194 | 0.44 | 0.3 | 0.49 | 0.24 | 0.32 | 0.29 | 0.69 | 0.43 | 0.28 | 1.25 | 0.95 | 0.46 | LOC100738869 |
| gene6195 | -1.108218397 | 0.000865132 | 8.25 | 8.07 | 8.04 | 22.88 | 12.23 | 9.74 | 6.86 | 14.23 | 7.67 | 6.32 | 5.97 | 7.93 | LOC100625316 |
| gene6216 | -1.252268129 | 0.001196707 | 0.73 | 1.08 | 1.04 | 0.72 | 1.29 | 1.13 | 0.68 | 0.48 | 1.26 | 0.51 | 0.32 | 0.48 | GEN1 |
| gene6220 | 1.473855902 | 7.62E-06 | 9.26 | 15.8 | 10.8 | 12.54 | 10.76 | 11.84 | 30.15 | 43.55 | 22.64 | 33.73 | 32.95 | 27.63 | LOC100518011 |
| gene6221 | 1.417590288 | 0.000112119 | 1.08 | 1.43 | 2.69 | 1.11 | 0.93 | 1.22 | 8.57 | 1.65 | 2.94 | 2.96 | 2.04 | 3.32 | LOC100518190 |
| gene6230 | -2.527603833 | 0.002259848 | 0.2 | 0.12 | 0.43 | 0.39 | 0.32 | 0.52 | 0.59 | 0.13 | 0.28 | 0 | 0.06 | 0.12 |  |
| gene6245 | -1.006689221 | 0.001689886 | 75.49 | 58.94 | 122.58 | 89.71 | 196.08 | 105.88 | 131.97 | 78.73 | 66.6 | 49.25 | 64.83 | 75.24 | ATP6V1C2 |
| gene6264 | -1.129156707 | 0.000710624 | 5 | 2.79 | 1.72 | 19.21 | 3.25 | 15.53 | 2.32 | 7.29 | 6.72 | 8.71 | 3.64 | 4.52 | IRG6 |
| gene6279 | 1.493967044 | 7.21E-05 | 1.4 | 1.71 | 1.62 | 1.58 | 0.73 | 1.36 | 1.98 | 2.75 | 3.55 | 1.72 | 3.7 | 4.36 | LOC100519387 |
| gene63 | -1.078288009 | 0.00089742 | 2.18 | 3.41 | 2.48 | 1.91 | 7.47 | 8.74 | 3.79 | 0.93 | 0.99 | 3.14 | 2.86 | 2.3 |  |
| gene6312 | -1.199889174 | 0.000200113 | 42.98 | 67.36 | 62.79 | 73.17 | 36.73 | 151.51 | 48.48 | 23.5 | 18.67 | 28.91 | 48.93 | 29.27 | LOC100518964 |
| gene6337 | -1.020092202 | 0.00315988 | 2.65 | 3 | 2.4 | 4.25 | 2.59 | 6 | 3.69 | 1.84 | 2.28 | 2.4 | 1.29 | 2.45 | LRRC24 |
| gene6359 | -1.398625996 | 0.000309729 | 1.86 | 2.21 | 2.12 | 1.74 | 2.4 | 1.73 | 2.27 | 1.13 | 1.56 | 0.99 | 0.49 | 0.73 | DGAT |
| gene6382 | -1.449471003 | 0.001534482 | 0.42 | 0.74 | 0.28 | 0.58 | 0.48 | 1.32 | 0.57 | 0.68 | 0.98 | 0.22 | 0.29 | 0.33 | LOC100737318 |
| gene6385 | -1.589725083 | 0.000370553 | 0.21 | 0.17 | 0.36 | 0.7 | 0.14 | 0.59 | 0.09 | 0.6 | 0.14 | 0.07 | 0.2 | 0.16 | LOC100628093 |
| gene6386 | -1.366047601 | 0.001626105 | 0.62 | 0.31 | 0.69 | 0.76 | 0.42 | 0.54 | 0.27 | 0.21 | 0.23 | 0.18 | 0.24 | 0.24 | RHPN1 |
| gene6387 | -1.093444167 | 0.007092972 |  |  |  |  |  |  |  |  |  |  |  |  |  |
| gene6408 | -1.096020091 | 0.007881788 | 0.78 | 0.26 | 0.14 | 0.34 | 0.4 | 0.55 | 0.16 | 0.15 | 0.13 | 0.3 | 0.06 | 0.22 | LOC100155271 |
| gene6519 | 2.312631987 | 7.59E-07 | 1.47 | 0.46 | 0.33 | 1.16 | 1.28 | 1.03 | 1.48 | 8.96 | 3.42 | 6.85 | 6.06 | 3.19 | ENY2 |
| gene6535 | -1.22854112 | 0.002003914 | 0.39 | 0.43 | 0.94 | 0.62 | 0.9 | 0.93 | 0.32 | 0.26 | 0.5 | 0.48 | 0.22 | 0.35 | ZFPM2 |
| gene6543 | -2.281223223 | 0.004204377 |  |  |  |  |  |  |  |  |  |  |  |  |  |
| gene6565 | 1.714603875 | 0.001829243 |  |  |  |  |  |  |  |  |  |  |  |  |  |
| gene6608 | -1.251449765 | 0.000463093 |  |  |  |  |  |  |  |  |  |  |  |  |  |
| gene6657 | 1.390952491 | 3.14E-05 | 27.79 | 10.4 | 9.09 | 5.4 | 3.75 | 9.79 | 9.25 | 9.7 | 7.78 | 11.07 | 13.3 | 22.52 | ATP6V0D2 |
| gene6658 | 1.485477719 | 4.72E-06 | 191 | 340.52 | 199.72 | 51.67 | 64.9 | 50.72 | 60.66 | 84.46 | 50.53 | 201.08 | 153.16 | 103.49 | CA2 |
| gene6659 | -3.33152117 | 4.74E-16 | 7.67 | 1.66 | 6.35 | 4.43 | 12.72 | 6.18 | 3 | 1.32 | 1.1 | 1.41 | 0.54 | 0.42 | CA3 |
| gene6684 | -2.108843978 | 1.83E-07 | 2.26 | 2.09 | 2.15 | 4.53 | 7.77 | 5.41 | 4.87 | 1.66 | 2.2 | 0.07 | 1.77 | 2.15 | IL7 |
| gene6699 | 1.768240616 | 1.04E-06 | 2.69 | 1.24 | 1.68 | 1.14 | 3.26 | 2.46 | 5.75 | 2.6 | 3.5 | 5.22 | 9.03 | 7.87 | LOC100158209 |
| gene6710 | -1.437670711 | 0.00242563 | 4.93 | 8.36 | 9.29 | 12.81 | 5.12 | 10.48 | 4.03 | 4.41 | 7.17 | 1.37 | 7.09 | 1.21 | LOC100737458 |
| gene6711 | -1.604399904 | 0.000111318 | 0.81 | 0.63 | 2.01 | 0.7 | 5.25 | 1.33 | 1.55 | 0.56 | 0.54 | 0.59 | 1.05 | 0.78 | LOC100517890 |
| gene6713 | -2.168774507 | 0.002104777 | 0.12 | 0.16 | 0.22 | 0.06 | 0.33 | 0.24 | 0.12 | 0.05 | 0.09 | 0.08 | 0 | 0.05 | LOC100518069 |
| gene6729 | 1.42993856 | 1.03E-05 | 19.85 | 23.15 | 21.59 | 13.2 | 12.58 | 14.51 | 8.65 | 22.19 | 25.93 | 51.55 | 25.79 | 28.69 | LOC100152427 |
| gene6735 | -1.45233728 | 2.68E-05 | 2.43 | 2.17 | 2.95 | 4.24 | 4.95 | 5.62 | 2.42 | 2.81 | 1.36 | 2.45 | 1.23 | 1.64 | LOC100524083 |
| gene6736 | -1.577227463 | 0.000222094 | 0.53 | 0.44 | 0.75 | 1.08 | 1.17 | 0.76 | 0.58 | 0.98 | 0.38 | 0.7 | 0.15 | 0.17 |  |
| gene6751 | 1.213298729 | 0.008355599 | 0.25 | 0.87 | 1.5 | 0.51 | 0.5 | 1.8 | 1.11 | 0.19 | 1.41 | 3.6 | 1.96 | 0.58 | LOC100525161 |
| gene6803 | -1.012885852 | 0.004184194 | 3.08 | 4.03 | 4.13 | 2.62 | 3.33 | 7.24 | 1.55 | 2.17 | 1.04 | 2.35 | 2.6 | 1.29 | LOC100154087 |
| gene6807 | 1.466215435 | 0.000247061 | 2.7 | 2.45 | 2.42 | 1.16 | 1.08 | 1.27 | 1.67 | 1.9 | 0.64 | 4.99 | 2.38 | 2.1 | LOC100156509 |
| gene6809 | 1.964613883 | 0.000532562 | 0.3 | 0.38 | 0.35 | 0.04 | 0.38 | 0.1 | 0.16 | 0.03 | 0.06 | 0.11 | 0.46 | 1.35 | OPRK1 |
| gene6810 | -2.434247263 | 1.48E-05 | 1.1 | 0.39 | 0.56 | 0.36 | 1.89 | 0.8 | 0.36 | 0 | 0 | 0.06 | 0.12 | 0.33 | LOC100628123 |
| gene6839 | 1.002558865 | 0.002272368 | 7.2 | 9.91 | 9.5 | 6.63 | 4.08 | 9.1 | 8.97 | 14.8 | 10.8 | 12.89 | 13.67 | 11.31 | C4H1orf114 |
| gene6853 | 1.421017042 | 0.001262131 | 0.29 | 0.1 | 0.2 | 0.15 | 0.1 | 0.07 | 0.07 | 0.24 | 0.08 | 0.31 | 0.41 | 0.11 | ADCY10 |
| gene6865 | -2.055385197 | 0.004164925 | 0.12 | 0.21 | 0.35 | 0.17 | 0.47 | 0.21 | 0.17 | 0.35 | 0.21 | 0.12 | 0.04 | 0.03 | LOC100737481 |
| gene6892 | 1.071962605 | 0.001927657 |  |  |  |  |  |  |  |  |  |  |  |  |  |
| gene6893 | 2.992517057 | 0.000139716 | 0 | 0.37 | 0.27 | 0.24 | 0 | 0.14 | 0.08 | 0.42 | 0.35 | 0.98 | 0.95 | 1.42 | LOC100620669 |
| gene6935 | 2.094300947 | 0.003796963 | 0.08 | 0 | 0 | 0.18 | 0.04 | 0.06 | 0.13 | 0.16 | 0.14 | 0.25 | 0.6 | 0.45 | ITLN2 |
| gene6962 | 1.744455339 | 0.001494881 | 0.37 | 0.38 | 0 | 0.18 | 0.34 | 0.16 | 1.02 | 1.06 | 0.46 | 0.79 | 0.71 | 0.8 | LOC100739124 |
| gene6964 | -1.101551694 | 0.009439399 | 3.56 | 5.93 | 5.28 | 1.97 | 5.57 | 3.14 | 5.42 | 3.6 | 3.51 | 2.57 | 1.06 | 1.35 | LOC100620368 |
| gene6966 | -1.879569582 | 2.04E-08 | 40.84 | 16.58 | 50.44 | 41.69 | 30.66 | 84.39 | 241.49 | 9.7 | 11.17 | 5.97 | 29.63 | 4.28 | CRP |
| gene6974 | 2.023929604 | 0.000839906 | 0.11 | 0.23 | 0.07 | 0.12 | 0.12 | 0.46 | 0.47 | 1.21 | 0.18 | 1.45 | 0.83 | 0.53 | LOC100152827 |
| gene6981 | -1.238557325 | 0.003565542 | 0.68 | 0.46 | 0.81 | 1.41 | 1.59 | 0.92 | 1.19 | 1.36 | 1.17 | 0.59 | 0.64 | 0.4 | CADM3 |
| gene6984 | 1.416039302 | 1.29E-05 | 31.01 | 38.75 | 32.54 | 26.66 | 21.37 | 32.44 | 45.72 | 56.35 | 70.45 | 56.45 | 58.94 | 90.18 | LOC100521317 |
| gene6988 | 1.705326255 | 0.003614803 | 0.12 | 0.44 | 0.11 | 0.27 | 0.14 | 0.26 | 0.22 | 0.37 | 0.14 | 0.45 | 0.5 | 0.85 | LOC100157689 |
| gene6996 | 1.87552126 | 0.001864797 |  |  |  |  |  |  |  |  |  |  |  |  |  |
| gene700 | 1.249300523 | 0.000132231 | 18.83 | 37.56 | 7.91 | 8.82 | 8 | 15.76 | 14.76 | 23.79 | 19.42 | 22.46 | 24.92 | 26.56 | LOC100522112 |
| gene7032 | -2.406062619 | 0.000384103 | 0.04 | 0.15 | 0.05 | 0.43 | 0.17 | 0.24 | 0.17 | 0 | 0.13 | 0.09 | 0.06 | 0 | FCRL3 |
| gene7042 | -1.253290394 | 0.001995588 | 0.65 | 0.43 | 0.83 | 1.19 | 1.25 | 1.63 | 1.45 | 0.71 | 0.51 | 0.57 | 0.57 | 0.54 | LRRC71 |
| gene7052 | -1.388340962 | 0.000232547 | 7.35 | 2.52 | 4.53 | 3.91 | 6.81 | 15.8 | 12.4 | 6.36 | 2.39 | 4.43 | 2.82 | 2.68 | CRABP2 |
| gene7057 | -2.515297114 | 0.002259848 | 0.07 | 0.11 | 0.1 | 0.12 | 0.76 | 0.32 | 0.19 | 0.08 | 0.06 | 0.12 | 0.06 | 0 | HAPLN2 |
| gene7058 | -1.112902267 | 0.001303182 | 0.64 | 1.25 | 0.73 | 0.56 | 1.82 | 2.18 | 1.58 | 0.81 | 2.31 | 0.76 | 0.76 | 0.52 | IQGAP3 |
| gene7070 | -1.376571443 | 0.000204234 | 19.69 | 17.03 | 22.35 | 18.55 | 34.5 | 18.19 | 10.49 | 16.89 | 7.31 | 9.36 | 9.07 | 8.99 | RHBG |
| gene7165 | -1.129573972 | 0.00059322 | 40.18 | 55.21 | 54.02 | 90.61 | 34.01 | 86.12 | 124.96 | 19.75 | 54.12 | 46.73 | 18.32 | 28.7 | LOC100156358 |
| gene7172 | 1.007078172 | 0.001633905 | 2134.6 | 2065 | 1536.2 | 2553.4 | 1436.8 | 1667.6 | 4959.6 | 2613.3 | 3421.5 | 4800 | 1768.2 | 4523.6 | S100A12 |
| gene7179 | 1.159454488 | 0.000475387 | 3.61 | 21.58 | 3.72 | 5.35 | 6.99 | 13.25 | 11.33 | 8.27 | 17.87 | 9.45 | 22.18 | 22.12 |  |
| gene7186 | 1.097980073 | 0.000607178 | 710.82 | 728.63 | 647.78 | 814.61 | 566.86 | 831.9 | 1052.7 | 786.29 | 1124.5 | 553.16 | 1248.1 | 2692.8 | IVL |
| gene7198 | -2.763774692 | 8.78E-16 | 16.19 | 5.38 | 11.44 | 14.01 | 12.4 | 85.26 | 41.8 | 8.3 | 5.83 | 1.53 | 7.54 | 6.13 | CRNN |
| gene7254 | 1.204014375 | 0.00020929 | 12.35 | 8.01 | 5.07 | 5.48 | 6.99 | 5.06 | 10.24 | 7.2 | 10.14 | 7.59 | 13.88 | 17.35 | ADAMTSL4 |
| gene7255 | 1.536297995 | 2.10E-06 | 510.57 | 271.72 | 139.51 | 141.58 | 184.76 | 156.52 | 244.54 | 234.73 | 239.16 | 348.13 | 397.57 | 605.31 | ECM1 |
| gene7271 | 2.064616394 | 0.009755884 | 0.17 | 0 | 0.13 | 0.34 | 0.11 | 0 | 0.16 | 0.2 | 0 | 1.1 | 1 | 0.28 | LOC100154181 |
| gene7281 | 2.204835189 | 7.10E-08 | 17.01 | 1.02 | 1.34 | 1.15 | 2.29 | 1.09 | 6.38 | 6.35 | 1.33 | 6.09 | 10.82 | 3.39 | LOC100155404 |
| gene7285 | 1.304664673 | 0.000107899 | 33.79 | 7.72 | 11.91 | 8.92 | 9.21 | 22.27 | 22.86 | 42.64 | 6.92 | 25.66 | 56.07 | 11.85 | LOC100624086 |
| gene7288 | 6.845172888 | 7.59E-10 | 0.04 | 0.04 | 0.17 | 0 | 0 | 0 | 0.19 | 0.18 | 0.04 | 0.17 | 0.24 | 0.8 | LOC100738895 |
| gene7291 | 1.045677654 | 0.001152049 | 108.91 | 77.25 | 65.37 | 65.55 | 62.67 | 65.07 | 137.12 | 173.76 | 152.11 | 94.47 | 150.92 | 137.13 | POLR3GL |
| gene7292 | -4.188394608 | 7.40E-28 |  |  |  |  |  |  |  |  |  |  |  |  |  |
| gene7347 | -2.08021134 | 0.000348499 |  |  |  |  |  |  |  |  |  |  |  |  |  |
| gene7366 | 1.636486129 | 0.001567695 |  |  |  |  |  |  |  |  |  |  |  |  |  |
| gene7376 | 1.875541813 | 1.02E-05 | 0.32 | 0.29 | 0.38 | 0.17 | 0.34 | 0.22 | 0.12 | 0.43 | 0.15 | 1.16 | 1.14 | 0.34 | AMPD1 |
| gene7386 | 1.222609765 | 0.001196707 | 1.32 | 1.54 | 1.27 | 0.97 | 0.71 | 0.58 | 1.44 | 1.97 | 1.95 | 1.44 | 2.4 | 1.32 | LOC100516672 |
| gene7388 | 1.370685085 | 6.67E-05 | 12.5 | 9.67 | 10.4 | 6.93 | 4.98 | 9.55 | 13.06 | 25.3 | 16.41 | 14.33 | 30.4 | 7.62 | LOC100517031 |
| gene7405 | 1.172695755 | 0.000268631 | 45.07 | 48 | 40.94 | 34.03 | 32.48 | 34.4 | 47.82 | 36.72 | 66.19 | 45.6 | 74.08 | 97.95 | CTTNBP2NL |
| gene7406 | -1.212187222 | 0.002211939 | 0.57 | 0.55 | 0.79 | 1.24 | 0.69 | 1.63 | 1.1 | 0.75 | 0.54 | 0.63 | 0.24 | 0.6 | KCND3 |
| gene7408 | -1.447551685 | 0.000213012 | 1.62 | 2.86 | 1.73 | 3.38 | 1.97 | 5.39 | 1.05 | 3.16 | 1.1 | 1.1 | 1.49 | 1.16 | LOC100153198 |
| gene7411 | 1.131710985 | 0.003875252 | 0.36 | 0.53 | 0.73 | 0.87 | 0.66 | 0.59 | 0.54 | 1.18 | 0.59 | 1.51 | 1.36 | 1.55 | ADORA3 |
| gene7419 | 2.323199635 | 0.002761731 | 0.02 | 0.03 | 0.06 | 0 | 0.08 | 0.03 | 0.2 | 0.1 | 0.16 | 0.22 | 0.18 | 0.23 | DENND2D |
| gene7448 | 2.13294093 | 2.49E-08 | 0.73 | 0.89 | 0.91 | 0.87 | 0.39 | 0.48 | 0.6 | 3.46 | 0.37 | 1.15 | 5.07 | 0.92 | EPS8L3 |
| gene7467 | -1.749609825 | 1.88E-07 | 4.49 | 3.78 | 5.76 | 4.34 | 9.77 | 9.9 | 7.74 | 1.32 | 1.03 | 2.96 | 2.52 | 1.49 | LOC100154257 |
| gene7485 | -1.027736598 | 0.00534567 | 1.76 | 0.55 | 0.91 | 1.48 | 1.77 | 3.12 | 3.84 | 1.66 | 2.13 | 1.01 | 0.99 | 0.98 | NTNG2 |
| gene7500 | -2.443860533 | 2.72E-06 | 0.23 | 1.1 | 2.09 | 3.2 | 1.61 | 0.84 | 0.75 | 0.73 | 0.41 | 0.94 | 0.09 | 0.16 | LOC100737939 |
| gene7501 | -2.197099131 | 9.86E-10 | 0.45 | 0.61 | 1.54 | 3.8 | 0.85 | 1.05 | 0.68 | 0.43 | 0.5 | 0.62 | 0.27 | 0.33 | COLL11A1 |
| gene7503 | -1.00243001 | 0.004182722 | 2.85 | 1.61 | 2.66 | 6.4 | 2.51 | 3.46 | 1.02 | 5.36 | 3.46 | 2.44 | 2.03 | 1.55 | OLFM3 |
| gene7504 | -1.353150794 | 0.000415797 |  |  |  |  |  |  |  |  |  |  |  |  |  |
| gene752 | 1.382074099 | 2.83E-05 | 50.28 | 29.84 | 15.41 | 5.77 | 6.05 | 12.38 | 11.57 | 14.17 | 12.52 | 15.93 | 14.77 | 29.33 | LOC100517385 |
| gene7522 | 2.384355216 | 1.94E-09 | 0.17 | 0.2 | 0.17 | 0.25 | 0.35 | 0.2 | 0.52 | 0.09 | 0.9 | 2.09 | 1.37 | 0.6 | LOC100158146 |
| gene753 | 1.411625358 | 1.30E-05 | 16.18 | 57.23 | 32.04 | 16.93 | 12.23 | 20.3 | 13.85 | 46.7 | 29.78 | 45.42 | 31.75 | 49.51 | LOC100153621 |
| gene7530 | -1.867088156 | 1.93E-07 | 3.02 | 1.19 | 2.11 | 4.97 | 6.62 | 6.2 | 2.39 | 0.96 | 2.72 | 1.58 | 1.3 | 1.89 | LOC100515728 |
| gene7534 | -2.787687304 | 0.000481506 |  |  |  |  |  |  |  |  |  |  |  |  |  |
| gene7548 | 1.157276915 | 0.000324538 | 85.42 | 117.78 | 120.84 | 89.95 | 67.29 | 88.82 | 70.46 | 117.68 | 153.06 | 134.24 | 189.68 | 201.17 | TMED5 |
| gene756 | 1.790545668 | 4.13E-08 | 62.78 | 77.89 | 78.08 | 17.09 | 28.73 | 37.62 | 15.72 | 33.28 | 66.06 | 79.4 | 69.8 | 128.23 | LOC100155557 |
| gene7580 | 4.976071015 | 0.001753584 | 0.08 | 0.06 | 0 | 0 | 0 | 0 | 0.08 | 0.08 | 0.13 | 0.08 | 0.2 | 0.11 | LOC100523668 |
| gene7590 | -2.35068952 | 4.30E-11 | 15.61 | 6.02 | 9.84 | 5.75 | 45.51 | 7.35 | 2.71 | 0.95 | 0.69 | 11.05 | 0.52 | 0.47 | LOC100738492 |
| gene7606 | 1.761485781 | 6.60E-08 | 425.33 | 136.35 | 253.24 | 159.71 | 118 | 118.49 | 311.96 | 605.46 | 131.27 | 146.97 | 670.7 | 454.19 | CYR61 |
| gene7643 | -2.026925601 | 0.000455095 | 0.44 | 0.76 | 1.41 | 0.99 | 0.31 | 1.54 | 0.18 | 0.19 | 0.39 | 0.14 | 0.49 | 0 | LOC100523243 |
| gene765 | -1.521280774 | 0.000325134 | 0.4 | 1.45 | 1.93 | 0.97 | 1.13 | 2.27 | 0.53 | 0.51 | 0.38 | 0.62 | 0.61 | 0.23 | SCG3 |
| gene7667 | -1.72573757 | 2.32E-05 | 1.16 | 1.33 | 2.33 | 1.49 | 2.81 | 2.05 | 0.9 | 0.66 | 0.43 | 0.91 | 0.51 | 0.5 | NAGA |
| gene7669 | 1.532024113 | 0.000202223 | 5.79 | 4.93 | 1.55 | 1.92 | 3.46 | 0.51 | 6.07 | 8.57 | 5.32 | 0.38 | 8.2 | 8.18 | LOC100737924 |
| gene7692 | -1.189718754 | 0.000698561 | 2.55 | 3.59 | 3.3 | 4.36 | 3.94 | 2.3 | 2.88 | 1.82 | 3.34 | 1.69 | 1.54 | 1.34 | LOC100155438 |
| gene7694 | 2.464241045 | 3.04E-11 | 0.27 | 0.47 | 0.77 | 0.8 | 0.88 | 0.41 | 5.71 | 7.24 | 2.25 | 7.05 | 2.95 | 1.51 | ENTHD1 |
| gene7715 | -1.53102969 | 0.000203983 | 1.03 | 0.27 | 3.15 | 1.94 | 1.46 | 3.31 | 0.89 | 0.23 | 1.23 | 1.69 | 0.29 | 0.32 | DMC1 |
| gene7718 | 1.956872852 | 0.007841465 | 0.11 | 0.1 | 0.07 | 0.1 | 0.06 | 0.04 | 0.16 | 0.09 | 0.1 | 0.28 | 0.4 | 0.16 | LOC100523060 |
| gene7722 | -1.059420437 | 0.001704843 | 4.18 | 3.22 | 4.84 | 3.28 | 4.94 | 5.14 | 2.97 | 1.37 | 2.54 | 2.72 | 1.55 | 2.01 | LOC100514711 |
| gene7723 | -1.122226974 | 0.000696956 | 16.78 | 9.61 | 14.62 | 21.5 | 39.82 | 21.29 | 16.17 | 4.53 | 12.69 | 10.56 | 9.47 | 17.11 | LOC100154063 |
| gene7725 | -1.075911807 | 0.001049731 | 10.57 | 6.41 | 7.66 | 13.7 | 24.56 | 11.26 | 13.72 | 4.81 | 9.06 | 9.93 | 5.57 | 7.68 | LOC100515261 |
| gene7729 | -1.391966583 | 6.24E-05 | 5.13 | 6.72 | 15.67 | 16.56 | 29.45 | 16.63 | 14.3 | 14.44 | 19.75 | 6.71 | 11.83 | 4.93 | LOC100737214 |
| gene7756 | -1.167474713 | 0.007720244 | 0.93 | 1.08 | 0.78 | 2.79 | 3.14 | 2.25 | 1.51 | 2.18 | 0.8 | 0.49 | 2.18 | 0.84 | LOC100621675 |
| gene7780 | -3.925388187 | 4.52E-17 | 0.48 | 0.14 | 0.94 | 1.09 | 7.63 | 1.35 | 1.83 | 0.2 | 0.5 | 0.36 | 0.2 | 0.09 | LOC100518305 |
| gene7794 | -1.236108119 | 0.00108142 | 0.94 | 1 | 0.4 | 0.95 | 1.8 | 0.89 | 1.46 | 0.84 | 0.69 | 0.56 | 0.34 | 0.62 | LOC100520194 |
| gene78 | 1.699002681 | 8.12E-07 | 1.71 | 0.72 | 0.6 | 0.63 | 0.91 | 0.29 | 1.82 | 1.65 | 1.62 | 1.74 | 2.52 | 1.57 | SYNJ2 |
| gene7817 | 2.005431071 | 2.75E-07 | 8.91 | 3.89 | 2.7 | 1.19 | 0.98 | 1.7 | 1.72 | 3.99 | 1.49 | 3.57 | 4.28 | 6.49 | LOC100737739 |
| gene782 | -1.353012288 | 0.002474679 | 2.13 | 0.78 | 2.38 | 1.83 | 6.17 | 2.96 | 1.42 | 0.62 | 0.77 | 0.8 | 1.53 | 1.96 | LOC100739494 |
| gene7832 | -3.791882432 | 1.37E-25 | 12.94 | 18.18 | 13.83 | 37.2 | 31.92 | 73.38 | 95.3 | 4 | 2.82 | 2.05 | 6.41 | 1.27 | PRPH |
| gene7836 | -1.70893128 | 4.74E-05 | 0.75 | 1.16 | 1.57 | 1.26 | 1.19 | 3.68 | 0.89 | 0.87 | 0.44 | 0.06 | 1.13 | 0.51 | LOC100623474 |
| gene7847 | -1.268589412 | 0.00079195 | 2.75 | 4.82 | 6.73 | 5.9 | 5.1 | 1.18 | 0.71 | 8.55 | 0.69 | 3.9 | 0.28 | 1.05 | AQP5 |
| gene7873 | -1.178924733 | 0.002663321 | 0.78 | 1.18 | 0.92 | 1.1 | 1.75 | 1.29 | 1.06 | 0.55 | 1.29 | 0.8 | 0.47 | 0.58 | FIGNL2 |
| gene7886 | -1.085406139 | 0.008373283 | 0.41 | 1.05 | 0.33 | 0.52 | 0.81 | 1.84 | 3.36 | 0.16 | 0.34 | 0.16 | 0.15 | 1.08 | LOC100737440 |
| gene7891 | -2.853655698 | 7.20E-10 | 0.07 | 1.92 | 1.11 | 1.02 | 1.03 | 3.57 | 3.4 | 0.18 | 0.5 | 0.23 | 0.37 | 0.15 | LOC100621351 |
| gene7892 | -2.097411762 | 9.05E-09 | 2.28 | 5.42 | 3.03 | 4.19 | 3.5 | 5.5 | 6.62 | 0.81 | 0.69 | 1.18 | 1.02 | 0.79 | LOC100525745 |
| gene7895 | 1.109741966 | 0.000989813 | 1.67 | 2.96 | 1.3 | 2.5 | 2.39 | 3.63 | 31.51 | 1.76 | 2.63 | 9.04 | 2.84 | 6.06 | KRT5 |
| gene7902 | -1.323550007 | 0.008764111 | 0.42 | 0.59 | 0.55 | 0.5 | 0.61 | 0.31 | 1.33 | 0.08 | 0.13 | 0.32 | 0.19 | 0.1 | LOC100157304 |
| gene7903 | -1.330994409 | 4.75E-05 | 5.14 | 2.35 | 9.43 | 12.02 | 12.1 | 31.58 | 15.94 | 0.78 | 3.39 | 4.29 | 0.45 | 16.31 | KRT4 |
| gene791 | -1.364392342 | 0.000221234 | 5.17 | 8.56 | 16.43 | 4.83 | 10.69 | 30.2 | 5.73 | 2.98 | 6.42 | 4.01 | 6.96 | 5.82 | LOC100736750 |
| gene7910 | -1.669256291 | 3.59E-07 | 32.58 | 58.39 | 48.83 | 102.71 | 91.36 | 86.28 | 107.02 | 21.89 | 48.27 | 26.68 | 34.75 | 23.65 | IGFBP6 |
| gene792 | -1.239742977 | 0.005163794 | 1.92 | 1.18 | 3.49 | 5.23 | 2.63 | 5.19 | 3 | 2.4 | 1.05 | 1.74 | 1.9 | 1.72 | LOC100736658 |
| gene7921 | -1.048559912 | 0.008923152 | 0.13 | 0.39 | 0.21 | 0.17 | 0.31 | 0.5 | 0.29 | 0.19 | 0.5 | 0.22 | 0.17 | 0.06 | ESPL1 |
| gene7962 | -1.574947649 | 0.000166168 | 3.42 | 3.09 | 5.51 | 5.37 | 3.64 | 4.05 | 1.39 | 3.63 | 1.25 | 2.54 | 1.07 | 0.84 | LOC100521495 |
| gene7966 | -2.517255204 | 1.35E-06 | 1.03 | 1 | 0.12 | 2.03 | 5.87 | 0.86 | 1.44 | 4.26 | 0.6 | 0.7 | 0.38 | 0.5 | LOC100523787 |
| gene8 | -1.294234245 | 0.000408521 | 2.05 | 2.01 | 1.24 | 2.32 | 2.83 | 2.24 | 2.24 | 1.07 | 1.13 | 0.85 | 0.49 | 1.54 | LOC100736968 |
| gene8102 | 1.080570077 | 0.003336994 | 2.14 | 2.12 | 3.89 | 1.54 | 1 | 1.86 | 0.65 | 2.73 | 2.76 | 2.48 | 2.65 | 3.68 | GDF11 |
| gene8149 | 3.104123154 | 8.46E-12 | 0 | 0.11 | 0.54 | 0.59 | 0.16 | 0.39 | 0.51 | 2.66 | 0.48 | 0.37 | 8.57 | 0.39 |  |
| gene8164 | 1.80157538 | 0.003753698 | 0.14 | 0.03 | 0.19 | 0.01 | 0.05 | 0.1 | 0.07 | 0.04 | 0 | 0.52 | 0.07 | 0 |  |
| gene817 | 3.168115417 | 1.60E-18 | 16.31 | 5.15 | 4.52 | 4.45 | 0.49 | 3.35 | 4.36 | 19.97 | 17.36 | 25.71 | 30.52 | 14.05 | SQRDL |
| gene8176 | 4.335814206 | 1.65E-05 | 0.01 | 0.01 | 0.08 | 0.02 | 0 | 0 | 0.16 | 0.06 | 0.16 | 0.22 | 0.19 | 0.11 | STAT6 |
| gene8200 | -1.075359999 | 0.001555029 | 8.8 | 10.39 | 12.09 | 12.23 | 15.1 | 9.68 | 8.38 | 7.58 | 5.82 | 8.23 | 4.23 | 4.98 | LOC100737094 |
| gene8202 | -1.4609055 | 8.68E-06 | 4.31 | 6.52 | 10.25 | 7.73 | 7.43 | 19.51 | 3.84 | 5.69 | 2.74 | 4.62 | 2.58 | 4.89 | LRIG3 |
| gene8250 | 2.43673672 | 2.87E-13 | 919.05 | 599.54 | 753.16 | 187.87 | 169.67 | 199.08 | 346.33 | 363.47 | 850.44 | 1085 | 947.27 | 881.6 | CPSF6 |
| gene828 | -1.298106298 | 0.002854961 | 0.59 | 0.46 | 0.86 | 0.77 | 0.69 | 0.63 | 0.68 | 1.04 | 0.68 | 0.41 | 0.29 | 0.14 | LOC100522231 |
| gene8292 | 1.884788967 | 9.75E-09 | 158.39 | 36.53 | 30.15 | 30.03 | 23.45 | 26.28 | 72.82 | 80.52 | 92.05 | 44.45 | 144.78 | 90.03 | LOC100523904 |
| gene8325 | -1.007066694 | 0.005403634 | 2.2 | 1.78 | 1.93 | 5.54 | 3.93 | 3.02 | 1.93 | 2.28 | 3.44 | 3.03 | 1.85 | 1.3 | LOC100516012 |
| gene8333 | 1.225731902 | 0.000153608 | 17.5 | 14.42 | 17.02 | 13.92 | 10.34 | 11.39 | 17.02 | 27.01 | 44.05 | 23.3 | 30.22 | 26.55 | LOC100625791 |
| gene834 | -1.31972458 | 0.000138128 | 5.23 | 7.18 | 4.93 | 4.03 | 7.82 | 11.16 | 4.71 | 6.04 | 2.3 | 2.53 | 3.33 | 2.96 | SORD |
| gene835 | -3.916827648 | 2.64E-05 | 0.4 | 0.35 | 0.45 | 0.4 | 0.52 | 0.9 | 0.5 | 0.18 | 0.5 | 0 | 0 | 0.08 | LOC100516583 |
| gene8356 | 1.184582322 | 0.000245822 | 8.82 | 9.4 | 11.63 | 5.08 | 8.89 | 9.69 | 7.37 | 15.91 | 9.21 | 20.31 | 12.01 | 19.9 | ABCC9 |
| gene8376 | 2.14555596 | 0.009755884 | 0.6 | 0.78 | 0.86 | 0 | 0.55 | 0.18 | 0.93 | 0.51 | 0 | 1.95 | 0.38 | 0.77 | LOC100623001 |
| gene8383 | -1.160489312 | 0.000608202 | 19.24 | 11.23 | 5.26 | 6.77 | 43.6 | 15.46 | 15.13 | 10.36 | 18.34 | 13.45 | 2.97 | 13.1 | LMO3 |
| gene839 | -1.020068166 | 0.001464116 | 190.6 | 223.47 | 183.34 | 398.41 | 194.42 | 301.94 | 167.15 | 263.18 | 153 | 114.33 | 176.39 | 130.55 |  |
| gene8390 | -2.097435173 | 2.06E-09 | 5.82 | 3.7 | 12.35 | 8.9 | 16.6 | 6.01 | 19.54 | 2.97 | 2.28 | 5.19 | 1.33 | 0.94 | PTPRO |
| gene8392 | -1.194543029 | 0.000415304 | 2.91 | 4.1 | 5.25 | 4.49 | 10.64 | 3.36 | 8.85 | 3.02 | 1.49 | 4.37 | 2.64 | 1.09 | LOC100738699 |
| gene8393 | 1.417466954 | 1.22E-05 | 209.17 | 152.74 | 211.17 | 69.55 | 78.51 | 72.73 | 41.77 | 147.43 | 153.38 | 184.25 | 176.09 | 210.1 | RERG |
| gene8410 | -2.082053177 | 2.60E-06 | 0.78 | 0.75 | 1.8 | 0.49 | 0.32 | 0.56 | 0.07 | 0.12 | 0.07 | 0.1 | 0.15 | 0.06 | GRIN2B |
| gene8411 | 1.780678393 | 2.80E-05 | 2.6 | 0.06 | 2.05 | 1.93 | 0.11 | 2.65 | 3.05 | 7.58 | 2.67 | 2.5 | 8.67 | 3.87 | LOC100739189 |
| gene8433 | 1.200556239 | 0.000184987 | 361.04 | 458.37 | 388.1 | 288.24 | 230.58 | 258.43 | 359.84 | 508.94 | 506.65 | 553.57 | 582.8 | 585.08 | LOC100518125 |
| gene8457 | -1.524756209 | 1.82E-05 | 8.95 | 3.16 | 6.2 | 7.43 | 9.25 | 7.67 | 1.8 | 5.06 | 2.76 | 2.86 | 1.76 | 3.64 | LOC100520135 |
| gene8458 | -1.54909749 | 0.000138815 | 2.3 | 3.53 | 3.9 | 6.27 | 3.25 | 4.72 | 1.55 | 3.81 | 1.55 | 2.66 | 1.64 | 0.53 | LOC100520308 |
| gene8461 | 1.766729035 | 0.000475525 | 0.58 | 0.36 | 0.14 | 0.44 | 0.6 | 0.21 | 0.09 | 1.13 | 0.66 | 2.64 | 0.26 | 1.08 | KLRF1 |
| gene8549 | 1.080389179 | 0.003418231 | 1.72 | 4.65 | 4.62 | 3.89 | 3.32 | 2.3 | 5.8 | 5.5 | 2.38 | 6.17 | 7.79 | 5.56 | CCND2 |
| gene8603 | -1.249849179 | 0.000107293 | 95.03 | 62.74 | 64.76 | 223.76 | 115.19 | 110.99 | 67.1 | 165.83 | 97.29 | 72.57 | 62.48 | 48.61 | SLC2A13 |
| gene8606 | 1.511359781 | 9.63E-05 | 0.97 | 0.44 | 0.41 | 0.12 | 3.11 | 0.73 | 5.24 | 2.93 | 4.9 | 4.55 | 1.77 | 5.03 | LOC100737691 |
| gene8613 | -1.033498455 | 0.002375082 | 1.56 | 2.33 | 2.65 | 3.63 | 3.71 | 1.86 | 0.98 | 3.03 | 1.45 | 1.36 | 2.4 | 0.63 | PDZRN4 |
| gene8636 | -1.329389092 | 0.001680849 | 0.79 | 0.64 | 0.82 | 1.05 | 1.51 | 0.56 | 0.72 | 1.14 | 0.77 | 0.41 | 0.11 | 0.73 | SLC38A4 |
| gene864 | -2.846324545 | 0.000328194 | 0.8 | 0.77 | 0.71 | 1.41 | 1.69 | 1.95 | 0.32 | 0.56 | 0.47 | 0 | 0 | 0.59 | LOC100736659 |
| gene8645 | 1.981287925 | 3.29E-08 | 0.37 | 3.43 | 0.9 | 1.01 | 3.34 | 5.52 | 9.04 | 4.78 | 14.09 | 26.41 | 2.72 | 9.23 | LOC100739479 |
| gene8705 | -2.264512302 | 3.09E-05 | 1.05 | 1.29 | 2.1 | 1.06 | 1.65 | 3.37 | 1.94 | 1.5 | 0.53 | 0.49 | 0.48 | 0.22 | LOC100738422 |
| gene8707 | -1.517284516 | 0.000188948 | 2.1 | 2.22 | 2.47 | 1.94 | 3.11 | 2.86 | 1.99 | 2.51 | 0.65 | 0.6 | 0.72 | 1.36 | LOC100521900 |
| gene8722 | -2.706741248 | 2.31E-13 | 2.7 | 2.39 | 2.47 | 2.7 | 4.82 | 10.34 | 8.92 | 1.51 | 0.57 | 1.12 | 1.3 | 0.21 | SLC5A8 |
| gene8724 | -1.597050953 | 1.09E-05 | 2.5 | 1.95 | 3.04 | 7.85 | 4.18 | 3.04 | 1.73 | 3.68 | 2.45 | 1.39 | 1.18 | 2.3 | LOC100739152 |
| gene875 | 1.58568353 | 0.00104058 | 0.4 | 0.23 | 0.24 | 0.18 | 0.14 | 0.24 | 0.31 | 0.89 | 0.24 | 0.28 | 0.58 | 0.69 | LOC100152957 |
| gene8750 | -1.299062363 | 0.002493083 | 0.73 | 0.35 | 0.44 | 1.07 | 0.6 | 0.9 | 1 | 0.61 | 0.4 | 0.49 | 0.36 | 0.19 | LOC100154617 |
| gene8761 | 1.857328205 | 1.80E-05 | 0.19 | 0.19 | 0.24 | 0.14 | 0.12 | 0.23 | 0.18 | 0.34 | 0.24 | 0.35 | 0.62 | 0.67 | LOC100515833 |
| gene8762 | 1.717722752 | 4.28E-07 | 6.17 | 6.09 | 11.4 | 5.31 | 5.24 | 5.38 | 7.06 | 7.47 | 9.88 | 9.21 | 14.74 | 26.1 | LOC100737604 |
| gene8777 | -1.221354263 | 0.00090676 | 10.48 | 10.43 | 18.51 | 9.26 | 17.17 | 26.97 | 12.79 | 6.83 | 8.84 | 10.38 | 5.23 | 6.83 | LOC100738154 |
| gene8784 | 1.05014046 | 0.00578984 | 1.12 | 0.62 | 1.66 | 1.01 | 1.33 | 1.34 | 1.09 | 1.67 | 0.81 | 2.23 | 1.07 | 4.07 | LOC100157843 |
| gene8788 | 3.085061079 | 1.38E-05 |  |  |  |  |  |  |  |  |  |  |  |  |  |
| gene8789 | -1.157242746 | 0.001552707 |  |  |  |  |  |  |  |  |  |  |  |  |  |
| gene8792 | -1.819443373 | 0.004671314 | 0.55 | 0.38 | 0.16 | 0.41 | 0.26 | 0.19 | 0.4 | 0.1 | 0.04 | 0.08 | 0.12 | 0.06 | GALNT4 |
| gene8803 | -1.352678235 | 0.009920114 | 0.18 | 0.08 | 0.05 | 0.2 | 0.07 | 0.67 | 0.05 | 0.03 | 0.03 | 0.15 | 0.08 | 0.11 | LOC100526053 |
| gene8806 | -1.928172904 | 6.74E-08 | 0.77 | 0.41 | 0.67 | 1.06 | 0.59 | 4.25 | 0.3 | 0.63 | 0.25 | 0.47 | 0.48 | 0.5 | LRRIQ1 |
| gene8829 | -1.152403038 | 0.000987585 | 1.37 | 1.65 | 2.62 | 7.43 | 3.69 | 3.58 | 1.78 | 6.83 | 2 | 3.67 | 1.79 | 1.11 | SYT1 |
| gene8834 | -1.436930847 | 0.000145279 | 0.45 | 0.76 | 0.53 | 0.31 | 2.55 | 0.88 | 1.01 | 0.29 | 0.85 | 0.41 | 0.53 | 0.45 | E2F7 |
| gene8851 | -1.554375936 | 0.008368548 |  |  |  |  |  |  |  |  |  |  |  |  |  |
| gene8859 | -1.393195607 | 0.003393968 | 0.14 | 0.17 | 0.13 | 0.21 | 0.69 | 0.37 | 1.26 | 0.07 | 0.14 | 0.22 | 0.17 | 0.08 | LOC100520675 |
| gene8875 | -1.110658978 | 0.001200715 | 8.87 | 8.77 | 9.99 | 11.39 | 9.5 | 11.82 | 11.03 | 7.06 | 5.97 | 6.56 | 4.26 | 4.02 | LOC100739038 |
| gene8880 | 1.377822216 | 2.38E-05 | 6.81 | 8.77 | 6.62 | 8.42 | 10.03 | 8.16 | 25.41 | 25.4 | 19.45 | 21.77 | 24.41 | 20.82 | LOC100627090 |
| gene8884 | 3.111028549 | 3.99E-19 | 7.27 | 9.02 | 3.11 | 7.57 | 6.04 | 6.2 | 46.08 | 88.22 | 44.42 | 53.33 | 67.95 | 43.6 | DPEP1 |
| gene8931 | -2.604178256 | 1.33E-13 | 2.43 | 2.11 | 2.38 | 4.46 | 12.17 | 5.87 | 10.62 | 1.44 | 1.8 | 1.74 | 1.38 | 0.55 | ATP2C2 |
| gene8946 | -2.307212068 | 7.21E-09 | 20.37 | 13.52 | 26.52 | 15.88 | 19.65 | 11.63 | 2.18 | 5.18 | 5.06 | 3.83 | 4.82 | 1.01 | HSD17B2 |
| gene8953 | -1.285112308 | 0.003270322 | 1.5 | 2.16 | 0.82 | 2.98 | 2.21 | 4.52 | 1.6 | 5.31 | 2.29 | 1.24 | 1.94 | 0.77 | LOC100519844 |
| gene8956 | -1.246490283 | 0.006173266 | 4.79 | 5.73 | 7.3 | 11.25 | 6.38 | 8.33 | 3.95 | 11.89 | 3.98 | 4.13 | 3.76 | 2.88 | LOC100518839 |
| gene8959 | 2.698833649 | 0.00364903 | 0 | 3.64 | 4.16 | 0.41 | 0.4 | 0 | 0 | 1.83 | 1.81 | 2.26 | 1.11 | 3.98 | LOC100738925 |
| gene8978 | -1.148088535 | 0.000997678 | 2.93 | 4.54 | 4.91 | 3.13 | 6.67 | 5.84 | 2.91 | 2.35 | 1.07 | 2.55 | 1.91 | 2.43 | LDHD |
| gene8986 | 1.032187482 | 0.001442226 | 17.63 | 26.87 | 11.81 | 18.95 | 15.52 | 15.74 | 34.35 | 23.55 | 32.85 | 20.88 | 38.94 | 38.36 | FA2H |
| gene8989 | 1.033003673 | 0.005773234 | 2.27 | 5.37 | 6.02 | 2.49 | 3.99 | 2.92 | 4.89 | 4.28 | 4.12 | 3.59 | 8.27 | 6.44 | LOC100736836 |
| gene8994 | -2.101017132 | 9.70E-07 | 0.33 | 0.6 | 1.11 | 1.37 | 1.32 | 2.9 | 1.33 | 0.79 | 0.44 | 0.5 | 0.44 | 0.4 | CLEC18A |
| gene9002 | -1.365682272 | 0.001897322 | 1.11 | 0.95 | 1.44 | 4.16 | 3.91 | 1.39 | 3.24 | 1.01 | 2.44 | 0.84 | 1.4 | 1.37 | LOC100737468 |
| gene9019 | 1.018254589 | 0.004881211 | 1.34 | 1.5 | 1.86 | 0.86 | 0.96 | 0.98 | 0.88 | 0.55 | 1.7 | 1.67 | 1.44 | 2.31 | LOC100514525 |
| gene9025 | -2.751887914 | 3.77E-16 | 999.68 | 1737.9 | 2063 | 2301.5 | 4479.4 | 4640.7 | 7479.1 | 36.14 | 213.1 | 389.77 | 970.58 | 256.38 | HP |
| gene9130 | 1.403404467 | 3.80E-05 | 63.14 | 11.64 | 12.39 | 6.88 | 21.79 | 33.86 | 176.34 | 94 | 132.38 | 29.19 | 115.23 | 10.49 | MT-III |
| gene9133 | -1.230690335 | 0.000136036 | 1265.3 | 131.65 | 555.1 | 343.49 | 250.25 | 1553.3 | 854.94 | 473.52 | 1113.6 | 220.41 | 461.89 | 170.71 | MT-2B |
| gene9142 | 1.58531512 | 0.001747473 | 0.31 | 0.31 | 0.05 | 0.6 | 0.13 | 0.22 | 0.97 | 0.64 | 0.81 | 0.88 | 0.37 | 1.68 | CES1 |
| gene9143 | 2.394056223 | 1.93E-06 | 0.07 | 0.19 | 0.16 | 0.31 | 0.11 | 0.05 | 0.97 | 0.43 | 0.3 | 1.08 | 0.27 | 0.91 | LOC100736962 |
| gene9144 | 1.559911086 | 0.00031222 | 0.25 | 0.88 | 0.27 | 0.76 | 0.98 | 0.5 | 2.6 | 1.32 | 1.14 | 1.63 | 0.87 | 4.05 | LOC100737013 |
| gene9151 | 1.020299589 | 0.006637023 | 3.06 | 4.96 | 2.92 | 1.25 | 2.52 | 4.61 | 3.8 | 4.58 | 4.06 | 6.16 | 4.51 | 5.43 | LOC100624004 |
| gene9155 | 1.170809997 | 0.000371195 | 8.05 | 3.49 | 3.12 | 7.15 | 5.57 | 4.37 | 7.61 | 12.82 | 13.89 | 8.93 | 15.43 | 12.58 | TOX3 |
| gene9169 | 1.663954679 | 4.21E-05 | 1.01 | 0.94 | 1.02 | 0.39 | 0.33 | 0.51 | 0.28 | 2.09 | 0.78 | 0.54 | 2.27 | 0.74 | LOC100626670 |
| gene9171 | -1.029515888 | 0.001379978 | 46.53 | 31.59 | 42.13 | 24.76 | 83.86 | 51.1 | 26.13 | 8.99 | 14.8 | 22.69 | 30.43 | 22.91 | GPT2 |
| gene9199 | -1.462069708 | 3.84E-05 | 1.23 | 1.07 | 1.35 | 3.74 | 1.59 | 3.24 | 1.99 | 1.25 | 1.06 | 1.57 | 0.7 | 0.77 | LRP3 |
| gene9201 | -1.315082943 | 7.48E-05 | 13.22 | 22.08 | 20.03 | 23.82 | 11.55 | 20.25 | 9.58 | 12.26 | 3.67 | 7.96 | 8.07 | 5.54 | CEBPA |
| gene9220 | -1.064523014 | 0.003384196 | 3.26 | 6.31 | 7.27 | 4.95 | 9.69 | 7.29 | 7.45 | 4.65 | 3.21 | 4.57 | 2.28 | 3.58 | LOC100737014 |
| gene9225 | 1.232073991 | 0.000165785 | 19.32 | 30.08 | 31.62 | 25.35 | 18.13 | 13.4 | 29.94 | 37.84 | 25.16 | 39.15 | 52.03 | 37.84 | LOC100624274 |
| gene9230 | 5.441248245 | 0.000178599 | 0.15 | 0.37 | 0 | 0 | 0 | 0 | 0 | 1.03 | 0.53 | 2.7 | 0.25 | 0.72 | HAMP |
| gene9231 | -1.897267311 | 7.38E-09 | 108.66 | 61.73 | 69.72 | 214.62 | 163.92 | 94.91 | 107.74 | 46.19 | 24.47 | 62.94 | 40.85 | 21.31 | LOC100516913 |
| gene9240 | -1.325356588 | 3.84E-05 | 893.18 | 550.34 | 450.37 | 1658.6 | 1303.2 | 669.05 | 875.31 | 406.49 | 210.93 | 502.48 | 584.41 | 322.37 | LOC100517283 |
| gene9251 | -5.924497155 | 2.63E-06 | 0.03 | 0.11 | 0.14 | 0.25 | 0.21 | 0.41 | 0.36 | 0.11 | 0.14 | 0 | 0 | 0 | ZBTB32 |
| gene9282 | -1.310418277 | 0.000463133 | 1.16 | 1.25 | 1.85 | 1.6 | 3.2 | 2.93 | 1.31 | 1.33 | 1.01 | 0.78 | 1.43 | 0.86 | ZNF461 |
| gene9300 | -1.857847333 | 0.006141164 |  |  |  |  |  |  |  |  |  |  |  |  |  |
| gene9334 | 1.995652283 | 2.46E-09 | 12.73 | 30.77 | 8.96 | 7.51 | 13.33 | 16.88 | 25.82 | 30.86 | 56.89 | 14.8 | 40.38 | 87.26 | LOC100628094 |
| gene9352 | 1.954998466 | 7.72E-08 | 0.77 | 1.46 | 1.69 | 1.72 | 1.42 | 1.62 | 1.55 | 6.68 | 2.91 | 3.52 | 5.73 | 8.47 | LOC100623625 |
| gene9355 | 2.619755429 | 2.23E-08 | 0.36 | 0.61 | 0.12 | 0.96 | 0.73 | 0.39 | 11.55 | 7.05 | 3.18 | 10.5 | 2.15 | 0.78 | LGALS13 |
| gene9361 | -1.282722863 | 0.000151327 | 1.87 | 1.72 | 1.73 | 2.19 | 1.76 | 3.73 | 1.62 | 0.92 | 0.95 | 0.88 | 0.73 | 1.41 |  |
| gene9377 | -2.728152724 | 0.000707479 | 0.12 | 0.1 | 0.11 | 0.37 | 0.1 | 0.15 | 0.15 | 0 | 0 | 0.03 | 0.03 | 0.02 | SPTBN4 |
| gene9384 | -4.75508837 | 0.003168798 | 0 | 0.61 | 1.15 | 0.9 | 0.22 | 0.28 | 0 | 0.1 | 0 | 0 | 0 | 0 | LOC100523846 |
| gene9403 | -1.450735772 | 0.002069172 | 0.41 | 1.29 | 1.76 | 1.79 | 3.29 | 1.85 | 1.01 | 1.56 | 0.81 | 0.88 | 1.18 | 0.62 | LOC100511932 |
| gene9418 | 4.157512022 | 7.83E-11 | 0 | 0.04 | 0 | 0.11 | 0.04 | 0 | 0 | 0.03 | 0 | 0 | 0.15 | 2.73 | LOC100512285 |
| gene9428 | -1.931880982 | 0.008227848 | 0.49 | 0.33 | 0.07 | 0.7 | 0.87 | 0.08 | 0.13 | 0.06 | 0.19 | 0.18 | 0.17 | 0.08 | LOC100514845 |
| gene943 | -1.335859717 | 0.004036632 | 0.86 | 2.86 | 0.4 | 0.88 | 1.99 | 3.53 | 2.59 | 1.14 | 2.37 | 1.09 | 0.64 | 0.88 | LOC100511489 |
| gene9434 | 1.077998966 | 0.00091833 | 252.5 | 58 | 91.68 | 73.92 | 78.11 | 53.43 | 52.89 | 86.76 | 100.3 | 206.15 | 90.3 | 130.72 | LOC100623158 |
| gene9448 | 3.991486066 | 2.00E-21 | 0.28 | 0.46 | 0.26 | 0.56 | 0.16 | 0.24 | 0.83 | 0.56 | 2.01 | 2.23 | 1.41 | 11.31 | LOC100519153 |
| gene9461 | 1.529526836 | 2.38E-06 | 200.5 | 142.92 | 150.14 | 132.17 | 75.63 | 74.26 | 164.35 | 271.02 | 160.14 | 141.69 | 335.14 | 299.49 | LOC100521017 |
| gene9470 | 5.345384452 | 0.000312409 |  |  |  |  |  |  |  |  |  |  |  |  |  |
| gene9484 | 1.02580431 | 0.009646915 | 0.79 | 1.9 | 2.22 | 0.81 | 1.04 | 1.08 | 0.92 | 1.39 | 0.69 | 1.91 | 1.53 | 2.3 | LOC100525346 |
| gene9491 | -2.004036531 | 1.08E-09 | 134.68 | 115.41 | 217.35 | 632.85 | 271.12 | 712.4 | 211.35 | 106.3 | 190.31 | 85.69 | 73.69 | 223.78 | APOE |
| gene9524 | -1.139432314 | 0.00121051 | 6.35 | 9.19 | 8.17 | 12.47 | 8.08 | 11.42 | 4.89 | 8.26 | 7.23 | 5 | 3.06 | 5.94 | LOC100514771 |
| gene9534 | 3.213724143 | 1.24E-20 | 7.39 | 9.88 | 4.73 | 11.3 | 8.92 | 8.2 | 109.84 | 118.01 | 60.04 | 119.92 | 85.04 | 52.38 | ZNF541 |
| gene9545 | 2.251340884 | 0.004204377 | 0.13 | 0.11 | 0 | 0.34 | 0 | 0 | 0.11 | 0.56 | 0.23 | 0.22 | 0.84 | 0.57 | LOC100622338 |
| gene9547 | 1.390382829 | 0.000243452 | 4.8 | 4.76 | 7.66 | 3.32 | 3.29 | 5.31 | 6.51 | 3.51 | 5.92 | 8.38 | 9.2 | 12.24 | LOC100520616 |
| gene9553 | -1.131212709 | 0.002951096 | 0.92 | 0.53 | 1.08 | 0.71 | 1.74 | 1.81 | 1.08 | 1.06 | 0.57 | 0.63 | 0.67 | 0.58 | LOC100622853 |
| gene9562 | -2.421035445 | 0.000130065 | 0.37 | 0.15 | 0.27 | 0.52 | 1.08 | 0.65 | 0.9 | 0.04 | 0.07 | 0.13 | 0.19 | 0.16 | LOC100521627 |
| gene9569 | -1.041803838 | 0.003057036 | 3.36 | 2.23 | 3.34 | 4.53 | 8.9 | 5.19 | 5.64 | 4.24 | 4.09 | 2.82 | 2.7 | 3.34 | SULT2B1 |
| gene957 | 1.012140124 | 0.001546102 | 243.99 | 431.07 | 367.52 | 226.81 | 269.3 | 219.12 | 324.59 | 289.09 | 445.14 | 523.53 | 426.05 | 453.63 | THBS1 |
| gene9574 | -1.842657691 | 0.003753698 | 0.43 | 0.17 | 0.31 | 0.77 | 0.54 | 0.5 | 0.34 | 0.45 | 0.68 | 0.13 | 0.18 | 0.17 | LOC100524747 |
| gene9587 | 1.198149884 | 0.000193315 | 150.76 | 73.82 | 108.83 | 73.79 | 48.45 | 78.79 | 92.81 | 129.31 | 117.9 | 84.24 | 169.9 | 183.66 | LOC100522679 |
| gene959 | -1.162650626 | 0.000424152 | 7.12 | 5.48 | 10.24 | 7.62 | 9.49 | 9.29 | 3.68 | 8.24 | 2.94 | 4.09 | 4.48 | 2.87 | RASGRP1 |
| gene9673 | 1.948951682 | 0.001506099 | 0 | 0.66 | 0.45 | 0.09 | 0.09 | 0.38 | 0.57 | 0.35 | 2.01 | 0.33 | 1.24 | 0.37 | LOC100512905 |
| gene9715 | -3.314714378 | 5.50E-06 | 0.48 | 0.35 | 0.54 | 0.27 | 0.38 | 0.34 | 0.06 | 0.06 | 0.06 | 0.07 | 0.03 | 0 |  |
| gene9734 | -1.344772078 | 0.007260216 | 0.87 | 0.8 | 0.73 | 1.48 | 0.74 | 1.41 | 0.68 | 1.87 | 0.57 | 1.14 | 0.23 | 0.2 | LOC100519779 |
| gene975 | 1.984523747 | 3.99E-09 | 12.73 | 47.55 | 13.33 | 5.94 | 8.12 | 13.34 | 7.42 | 13.48 | 9.07 | 48.77 | 33.25 | 22.8 |  |
| gene9780 | 2.040320256 | 3.14E-05 | 0.4 | 0.18 | 0.17 | 0.25 | 0.08 | 0.1 | 0.36 | 0.07 | 0.24 | 0.41 | 0.11 | 1.13 | PTPRH |
| gene9790 | -1.538049019 | 0.002687961 | 1.28 | 1.3 | 0.37 | 1.63 | 1.41 | 0.8 | 0.81 | 0.87 | 0.78 | 0.63 | 0.26 | 0.48 | TMEM86B |
| gene9892 | 1.560030345 | 2.48E-06 |  |  |  |  |  |  |  |  |  |  |  |  |  |
| gene9911 | -1.052251505 | 0.002679902 | 2.61 | 3.57 | 3.29 | 3.43 | 4.86 | 7.4 | 4.02 | 2.23 | 2.58 | 3.57 | 1.92 | 1.85 | LOC100525702 |
| gene9913 | -1.013728699 | 0.003634553 | 3.7 | 3.89 | 5.43 | 4.4 | 6.06 | 5.57 | 4.46 | 1.71 | 3.25 | 2.98 | 2.48 | 2.29 | LOC100511758 |
| gene992 | 1.749816332 | 0.000972778 | 0.39 | 0.54 | 1.06 | 0.23 | 0.15 | 0.28 | 0.24 | 0.75 | 0.57 | 0.43 | 0.85 | 0.93 | LASS3 |
| gene9930 | -1.185607714 | 0.000450245 | 13.7 | 16.52 | 16.55 | 17.65 | 15.61 | 23.79 | 9.52 | 7.52 | 12.08 | 5.52 | 9.14 | 9.35 | LOC100624036 |
| gene9949 | 1.592508579 | 0.005041377 | 0.29 | 0.3 | 0.38 | 0.34 | 0.19 | 0.12 | 2.02 | 0.69 | 1.15 | 1.47 | 0.45 | 0.12 | LOC100737527 |
| gene9958 | -1.384397248 | 5.90E-05 | 1.79 | 2.75 | 3.05 | 5.03 | 4.28 | 3.6 | 4.23 | 0.89 | 3.22 | 1.84 | 1.64 | 1.32 | LOC100515319 |
| gene9971 | 2.25228862 | 1.35E-11 | 140.4 | 59.18 | 94.69 | 23.68 | 40.91 | 27.49 | 69.75 | 77.62 | 91.74 | 75.67 | 146.96 | 199.14 | LOC100519197 |
| gene9975 | 1.989259362 | 1.43E-09 | 286.83 | 132.53 | 180.71 | 72.68 | 65.84 | 77.38 | 158.42 | 196.59 | 196.14 | 191.9 | 285.49 | 342.82 | LOC100738544 |
| gene9992 | -2.666624441 | 9.82E-15 | 54.92 | 12.14 | 40.64 | 64.42 | 192.11 | 76.72 | 42.22 | 5.39 | 18.36 | 17.2 | 20.59 | 13.77 | RBP7 |
| gene9996 | 1.603777992 | 1.20E-06 | 21 | 32.46 | 22.18 | 22.2 | 27.81 | 31.67 | 32.11 | 51.48 | 71.8 | 70.67 | 57.18 | 111.02 | APITD1 |

Supplementary Table S3: Differentially expressed genes (DEGs) in QS

| Gene | logFC | PValue | LL1 | LL2 | LL3 | LN1 | LN2 | LN3 | QS1 | QS2 | QS3 | QL1 | QL2 | QL3 | Name |
| --- | --- | --- | --- | --- | --- | --- | --- | --- | --- | --- | --- | --- | --- | --- | --- |
| gene10085 | -1.709110524 | 0.00016821 | 0.47 | 1.2 | 0.57 | 0.29 | 0.46 | 1.91 | 0.26 | 0.38 | 0.09 | 0.56 | 0.63 | 0.22 | LOC100621356 |
| gene10120 | 2.396573316 | 2.22E-05 | 0.23 | 0.14 | 0.41 | 0.09 | 0.04 | 0.5 | 0.34 | 1.93 | 0.81 | 1.69 | 0.97 | 0.33 | LOC100624917 |
| gene10121 | 1.23890059 | 0.001136077 | 101.64 | 64.59 | 55.3 | 51.22 | 52.85 | 39.63 | 59.22 | 159.12 | 110.23 | 73.67 | 218.33 | 110.44 | CDA |
| gene10124 | -1.388913004 | 0.001734875 | 0.14 | 0.37 | 0.19 | 0.95 | 1.2 | 0.56 | 0.49 | 0.15 | 0.35 | 0.23 | 0.71 | 0.87 | LOC100738531 |
| gene10144 | -1.07525833 | 0.00819006 | 1.53 | 2.03 | 3.5 | 4.99 | 2.48 | 8.92 | 2.06 | 3.48 | 1.88 | 2.58 | 2.99 | 4.54 | C1QB |
| gene10172 | 1.132167598 | 0.008731808 | 3.93 | 4.15 | 0.99 | 4.01 | 2.49 | 5.31 | 7.75 | 11.7 | 4.49 | 3.77 | 4.49 | 3.97 | LOC100521800 |
| gene10257 | -2.063501598 | 5.31E-06 | 0.14 | 0.49 | 0.37 | 0.59 | 1.75 | 1.79 | 0.59 | 0.23 | 0.05 | 0.13 | 0.13 | 0.32 | MATN1 |
| gene10274 | -2.670558689 | 5.02E-08 | 0.4 | 0.54 | 0.96 | 0.57 | 1.27 | 1.82 | 0.32 | 0.17 | 0 | 0.13 | 0.08 | 0.46 | LOC100737666 |
| gene10276 | -1.387123907 | 0.000272078 | 12.97 | 11.07 | 16.19 | 14.16 | 16.64 | 19.68 | 7.29 | 5.71 | 5.59 | 11.78 | 9.17 | 9.95 | LOC100518132 |
| gene10346 | -1.479099201 | 0.005257109 | 0.16 | 0.15 | 0.41 | 0.46 | 0.12 | 0.49 | 0.21 | 0.04 | 0.09 | 0.35 | 0.14 | 0.05 | LOC100525585 |
| gene10380 | -1.14320736 | 0.002571645 | 22.42 | 15.41 | 26.3 | 15.59 | 71.04 | 20.06 | 27.03 | 6.52 | 13.67 | 12.2 | 11.98 | 18.79 | MFSD2A |
| gene10431 | 2.230522063 | 0.000386079 |  |  |  |  |  |  |  |  |  |  |  |  |  |
| gene1044 | -1.806792207 | 2.95E-06 | 31.99 | 15.79 | 34.61 | 28.99 | 55.49 | 27.83 | 13.02 | 6.29 | 11.88 | 10.41 | 6.42 | 9.59 | LOC100516053 |
| gene10472 | 1.12720676 | 0.003015284 | 5.62 | 7.24 | 8.72 | 4.53 | 3.93 | 3.43 | 11.17 | 6.6 | 7.27 | 8.69 | 6.92 | 8.03 | LOC100739192 |
| gene10498 | -1.172965732 | 0.007451828 | 0.04 | 0.18 | 0.17 | 0.86 | 0.3 | 0.88 | 0.49 | 0.17 | 0.17 | 0.47 | 0 | 0.14 | LOC100515669 |
| gene10499 | -1.305936523 | 0.001208125 | 0.18 | 0.58 | 0.91 | 1.95 | 2.24 | 2.56 | 0.98 | 0.42 | 1.29 | 0.91 | 0.8 | 0.93 | DSG1 |
| gene10501 | 1.033672765 | 0.006923337 | 1.6 | 4.27 | 1.33 | 1.85 | 5.17 | 2.61 | 4.78 | 2.37 | 12.07 | 3.18 | 5.59 | 9.06 | LOC100625733 |
| gene10517 | -1.531619076 | 0.00056014 | 0.15 | 0.17 | 0.28 | 0.39 | 0.3 | 0.3 | 0.12 | 0.11 | 0.1 | 0.19 | 0.12 | 0.13 | ASXL3 |
| gene10536 | -1.628502047 | 0.000275341 | 0.76 | 0.89 | 0.3 | 0.87 | 0.5 | 0.89 | 0.29 | 0.22 | 0.2 | 0.26 | 0 | 0.1 | LOC100621611 |
| gene10549 | 2.399366444 | 5.26E-05 | 0.47 | 0.3 | 0 | 0.26 | 0.03 | 0 | 0.04 | 0.36 | 1.25 | 0.2 | 0.04 | 0.32 | LOC100522145 |
| gene10568 | -1.060107669 | 0.005541708 | 6.72 | 6.75 | 5.38 | 35.46 | 6.4 | 14.2 | 6.69 | 11.56 | 7.56 | 11.68 | 4.67 | 5.39 | IFI44 |
| gene10588 | -1.131201708 | 0.005535987 | 1.94 | 1.4 | 2.35 | 1.83 | 2.98 | 0.71 | 0.66 | 0.82 | 1.01 | 2.75 | 0.78 | 1.07 | MSH4 |
| gene10611 | 1.756562631 | 0.002314319 | 0.07 | 0.04 | 0.05 | 0.05 | 0.04 | 0.1 | 0.16 | 0.28 | 0.16 | 0.24 | 0.16 | 0.06 | LOC100738520 |
| gene10616 | -1.426303162 | 0.000224687 | 13.81 | 16.61 | 15.12 | 34.84 | 30.9 | 29.16 | 8.48 | 15.59 | 10.1 | 17.5 | 10.15 | 10.16 | DIRAS3 |
| gene1066 | 2.250510743 | 0.000751877 |  |  |  |  |  |  |  |  |  |  |  |  |  |
| gene10677 | 1.205492018 | 0.006817902 | 2.22 | 2.61 | 2.41 | 1.61 | 1.25 | 0.8 | 3.42 | 2.68 | 2.04 | 2.32 | 2.51 | 3.86 | TMEM61 |
| gene10689 | -3.338165771 | 6.07E-12 | 2.19 | 1.79 | 0.59 | 2.6 | 1.26 | 1.18 | 0.21 | 0.07 | 0.19 | 0.23 | 0.06 | 0.1 | LOC100622589 |
| gene1075 | -1.24760634 | 0.001393425 | 1.96 | 1.88 | 1.81 | 3.72 | 12.44 | 2.48 | 6.09 | 0.49 | 0.87 | 2.32 | 2.5 | 1.28 | SERPINB7 |
| gene10751 | 2.674674565 | 2.10E-05 | 0.23 | 0.02 | 0.1 | 0.06 | 0.06 | 0.11 | 0.25 | 0.88 | 0.37 | 0.17 | 0.17 | 0.26 | LOC100523742 |
| gene10753 | -1.812114097 | 5.39E-06 | 4.3 | 6.55 | 2.76 | 10.91 | 6.15 | 5.99 | 1.72 | 2.81 | 1.82 | 5.15 | 3.13 | 2.12 | LOC100523909 |
| gene10796 | -1.458563958 | 0.000620535 | 4.44 | 1.98 | 4.28 | 3.63 | 7.68 | 4.62 | 2.3 | 2.09 | 1.25 | 2.08 | 2.64 | 0.84 | LOC100739546 |
| gene10856 | -1.388896641 | 0.002820064 | 0.52 | 0.39 | 0.53 | 0.53 | 2.06 | 1.09 | 0.36 | 0.43 | 0.68 | 0.51 | 0.42 | 0.69 | LOC100526058 |
| gene10872 | -1.835423475 | 0.009589069 |  |  |  |  |  |  |  |  |  |  |  |  |  |
| gene10897 | 1.949676181 | 0.000564659 | 0.8 | 1.13 | 1.06 | 0.83 | 0.31 | 0.26 | 2.2 | 1.94 | 1.08 | 0.29 | 1.67 | 0.38 | LOC100737220 |
| gene10911 | -1.575753097 | 0.000139094 | 3.17 | 3.49 | 3.44 | 4.14 | 4.06 | 3.93 | 2.26 | 0.98 | 0.47 | 0.87 | 2.25 | 0.77 | LOC100627410 |
| gene10915 | 3.033004753 | 1.79E-10 | 0.09 | 0.1 | 0.1 | 0.08 | 0.26 | 0.12 | 0.59 | 2.37 | 0.88 | 1.54 | 2.19 | 0.84 | LOC100627687 |
| gene10942 | -1.286280913 | 0.002825705 | 4.14 | 5.99 | 3.84 | 5.33 | 8.57 | 5.97 | 3.49 | 1.73 | 2.53 | 3.7 | 0.81 | 2.96 | SIRT5 |
| gene10957 | -3.131309429 | 9.13E-09 | 1.57 | 0.97 | 1.31 | 2.09 | 14.19 | 7.06 | 1.26 | 0.83 | 0.35 | 0 | 1.87 | 0.85 | LOC100522879 |
| gene10959 | -1.705237377 | 0.000120459 | 2.31 | 2.77 | 1.53 | 1.95 | 2.79 | 3.69 | 0.88 | 0.75 | 0.91 | 4.94 | 1.1 | 2.85 | LOC100524942 |
| gene10969 | -1.003906341 | 0.007859676 | 113.51 | 89.4 | 89.99 | 66.59 | 142.45 | 82.8 | 66.95 | 36.9 | 37.4 | 45.67 | 43.67 | 35.71 | TPMT |
| gene11012 | 1.258221825 | 0.003792563 |  |  |  |  |  |  |  |  |  |  |  |  |  |
| gene11038 | 1.565448348 | 0.000882287 | 1.17 | 0.67 | 0.75 | 0.39 | 0.76 | 0.31 | 1.03 | 2.01 | 1.15 | 1.28 | 1.09 | 0.1 | LOC100153723 |
| gene11058 | 1.737252333 | 0.000288033 | 2.74 | 1.77 | 1.49 | 1.31 | 1.24 | 1.97 | 2.8 | 9.31 | 1.72 | 1.44 | 8.41 | 0.69 | LOC100621915 |
| gene11069 | 3.608521639 | 1.64E-05 | 0 | 0 | 0 | 0 | 0 | 0.12 | 0.06 | 0.89 | 0.49 | 0.98 | 0.25 | 0.35 | LOC100514657 |
| gene11113 | -2.606526562 | 0.009710715 | 0 | 0.05 | 0.11 | 0.66 | 0.1 | 0.31 | 0 | 0.05 | 0.15 | 0.07 | 0 | 0 | GPX5 |
| gene11125 | -1.026189549 | 0.00666432 | 58.82 | 55.79 | 49.45 | 71.54 | 47.86 | 67.94 | 24.04 | 39.26 | 25.28 | 36.13 | 32.98 | 51.24 | SLA-3 |
| gene11127 | -2.418757168 | 7.41E-10 | 64.78 | 126.71 | 222.93 | 269.92 | 212.56 | 152.33 | 39.12 | 59.46 | 16.32 | 75.7 | 35.79 | 28.5 | LOC100513601 |
| gene1113 | -1.47088838 | 0.001039203 | 0.3 | 0.13 | 0.23 | 0.99 | 0.5 | 0.66 | 0.19 | 0.19 | 0.39 | 0.14 | 0.2 | 0.44 | CILP |
| gene11130 | -1.197173872 | 0.001550358 | 74.5 | 94.14 | 111.49 | 165.89 | 99.83 | 211.47 | 78.14 | 92.09 | 29.47 | 103.78 | 60.95 | 49.18 | SLA-1 |
| gene11138 | 1.157001319 | 0.002924496 | 11.01 | 3.99 | 5.06 | 4.65 | 5.72 | 3.67 | 2.91 | 13.56 | 13.9 | 8.58 | 21.12 | 33.32 | RNF39 |
| gene11140 | 1.4623196 | 0.001179031 | 2.04 | 0.19 | 0.41 | 0.36 | 3.99 | 0.59 | 1.56 | 4.52 | 8.37 | 0.66 | 5.27 | 6.27 | LOC100155727 |
| gene11144 | 1.625300848 | 2.27E-05 | 27.1 | 17.35 | 10.36 | 10.95 | 9.57 | 13.64 | 40 | 24.46 | 36.72 | 9.63 | 34.45 | 35.37 |  |
| gene1116 | -1.161042393 | 0.005399165 | 0.54 | 0.52 | 0.76 | 0.84 | 0.88 | 1.19 | 0.54 | 0.26 | 0.46 | 0.6 | 0.27 | 0.46 | LOC100513900 |
| gene11255 | 1.391369135 | 0.000253342 | 162.88 | 571.14 | 355.48 | 176.26 | 78.32 | 110.85 | 160.69 | 375.75 | 385.05 | 297.79 | 465.03 | 445.5 | LOC100154770 |
| gene11270 | -1.619597648 | 3.21E-05 | 21.15 | 7.77 | 3.92 | 1.62 | 6.13 | 53.39 | 5.25 | 10.67 | 2.85 | 10.13 | 16.07 | 29.11 |  |
| gene11296 | -1.245647072 | 0.001094398 | 3.84 | 2.72 | 2.94 | 6.53 | 2.28 | 7.91 | 3.55 | 2 | 1.15 | 3.84 | 1.58 | 2.06 | C4 |
| gene11307 | -1.266500688 | 0.00094199 | 1.82 | 1.39 | 1.72 | 1.29 | 2.89 | 2.91 | 1.43 | 0.58 | 0.83 | 2.15 | 0.99 | 1.82 | TNXB |
| gene11315 | 1.299211094 | 0.001389658 | 0.96 | 1.55 | 2.23 | 2.42 | 1.09 | 1.58 | 1.91 | 3.7 | 6.5 | 1.16 | 4.36 | 2.23 | AGER |
| gene11336 | 4.900064661 | 0.003722371 | 0.13 | 0.03 | 0 | 0 | 0 | 0 | 0.35 | 0.15 | 0 | 0 | 0.1 | 0.13 |  |
| gene11338 | -1.273155877 | 0.000874521 | 5.91 | 2.54 | 3.56 | 41.08 | 40.14 | 7.59 | 10.76 | 20.76 | 4.29 | 7.77 | 5.52 | 10.07 | SLA-DRB1 |
| gene1137 | 1.084808691 | 0.005490045 | 38.88 | 35.33 | 34.86 | 20.05 | 14.78 | 19.84 | 38.86 | 20.43 | 52.23 | 22.37 | 25.43 | 25.66 | SMAD3 |
| gene1138 | 2.168235799 | 0.006809308 | 0 | 0.65 | 0.26 | 0.23 | 0 | 0.29 | 0.56 | 0.93 | 0.91 | 0.48 | 0.16 | 0.14 | LOC100517924 |
| gene11386 | 1.789304927 | 4.35E-06 | 63.15 | 23.34 | 48.95 | 14.9 | 14.17 | 11.7 | 71.62 | 31.5 | 32.61 | 35.67 | 42.77 | 32.42 | LOC100522554 |
| gene11417 | 1.132959838 | 0.002687901 | 1371.1 | 1213.6 | 692.49 | 532.34 | 733 | 617.85 | 893.88 | 981.8 | 2113.6 | 546.15 | 1536.3 | 1589.8 | HMGA1 |
| gene11421 | -1.115553807 | 0.005468333 | 28.16 | 40.17 | 43.89 | 46.67 | 58.97 | 62.19 | 23.7 | 17.65 | 33.99 | 23.79 | 30.52 | 54.71 | LOC100737619 |
| gene11427 | 5.151344721 | 0.001226127 | 0.5 | 0 | 0 | 0 | 0 | 0 | 0.73 | 0.32 | 0.35 | 0.16 | 0.32 | 0 |  |
| gene11469 | -1.473982697 | 0.000146896 | 4.74 | 3.96 | 11.11 | 13.2 | 12.94 | 9.49 | 6.6 | 1.98 | 3.77 | 4.73 | 4.66 | 7.32 | PI16 |
| gene1150 | -1.033947891 | 0.008063467 | 2.4 | 1.83 | 2.31 | 2.63 | 2.82 | 3.1 | 2.08 | 0.8 | 1.13 | 3.51 | 2.54 | 2.41 | ITGA11 |
| gene11579 | 1.466625988 | 0.003235838 | 0.23 | 0.22 | 0.21 | 0.19 | 0.27 | 0.2 | 0.74 | 0.91 | 0.06 | 0.29 | 0.28 | 0.13 | CAPN11 |
| gene11591 | 1.272248554 | 0.002007076 | 1.9 | 1.16 | 1.36 | 1.22 | 1.01 | 0.74 | 1.25 | 2.82 | 2.88 | 1.7 | 2.05 | 1.62 | RUNX2 |
| gene11592 | 1.615774624 | 0.000152985 | 2.35 | 1.4 | 1.13 | 0.94 | 0.8 | 1.58 | 2.22 | 5.07 | 2.36 | 1.92 | 4.44 | 1.62 | LOC100737965 |
| gene11609 | 3.323151325 | 0.004537517 | 0.05 | 0.04 | 0.02 | 0.02 | 0 | 0 | 0.09 | 0.14 | 0.03 | 0 | 0.03 | 0.02 | MEP1A |
| gene11618 | 1.06639345 | 0.006726875 | 6.02 | 6.16 | 5.08 | 3.42 | 2.75 | 3.57 | 5.03 | 8.31 | 6.27 | 4.75 | 7.74 | 3.05 | LOC100739210 |
| gene11619 | 2.28439503 | 0.000109347 | 0.27 | 0.31 | 0.38 | 0.2 | 0.14 | 0.08 | 0.33 | 1.06 | 0.41 | 0.78 | 0.68 | 0.17 | LOC100516620 |
| gene11624 | 1.768309957 | 0.001436622 | 1.02 | 1.44 | 0.2 | 0 | 0.77 | 0.07 | 0.76 | 0.98 | 1.43 | 0.67 | 0.41 | 4.36 | CRISP3 |
| gene11643 | 1.201208353 | 0.002152072 | 5.23 | 3.78 | 4.71 | 9.23 | 5.18 | 8.18 | 9.22 | 26.35 | 14.21 | 8.55 | 8.86 | 8.11 | LOC100517538 |
| gene11675 | -1.019982003 | 0.007083601 | 24.52 | 18.98 | 19.67 | 24.13 | 49.22 | 33.86 | 23.76 | 11.63 | 15.78 | 19.74 | 16.4 | 17.58 | LOC100623036 |
| gene11690 | 2.255776741 | 2.42E-08 | 1.19 | 0.4 | 0.81 | 0.43 | 0.63 | 0.76 | 3.33 | 1.71 | 3.31 | 1.18 | 3.23 | 1.06 | ADAMTSL3 |
| gene11691 | 1.466134033 | 0.001054191 | 1.58 | 0.31 | 0.5 | 0.93 | 0.96 | 0.66 | 2.74 | 2.14 | 2.04 | 0.88 | 1.97 | 0.81 | LOC100739211 |
| gene11705 | 1.736824981 | 7.75E-05 | 0.44 | 0.48 | 0.27 | 0.31 | 0.22 | 0.23 | 0.78 | 1.18 | 0.46 | 0.72 | 0.61 | 0.33 |  |
| gene11724 | -1.604173578 | 0.003975336 |  |  |  |  |  |  |  |  |  |  |  |  |  |
| gene11725 | -2.682192974 | 0.001230899 |  |  |  |  |  |  |  |  |  |  |  |  |  |
| gene11744 | 1.372561592 | 0.000645929 | 7.06 | 2.47 | 1.85 | 1.44 | 3.5 | 1.79 | 3.69 | 9.16 | 4.08 | 4.86 | 14.62 | 5.32 | LOC100157078 |
| gene11757 | -1.912497395 | 0.000395022 | 0.26 | 0.25 | 1.15 | 0.58 | 0.67 | 0.9 | 0.33 | 0.11 | 0.08 | 0.4 | 0.75 | 0.49 | LOC100153433 |
| gene11818 | -2.096916601 | 2.98E-07 | 1.94 | 2.56 | 1.5 | 1.4 | 11.07 | 2.37 | 1.16 | 0.37 | 1.97 | 1.43 | 0.58 | 1.51 | ISLR2 |
| gene11822 | -1.463798226 | 0.000331387 | 0.76 | 1.77 | 3.72 | 4.3 | 2.37 | 3.95 | 1.56 | 0.56 | 1.61 | 1.25 | 0.57 | 1.35 | LOXL1 |
| gene11864 | 4.797636708 | 0.002125234 | 0.02 | 0.04 | 0.03 | 0 | 0 | 0 | 0 | 0.14 | 0.16 | 0.04 | 0.04 | 0.03 | LOC100152739 |
| gene11872 | 1.113296327 | 0.003193416 | 32.7 | 43.94 | 36.19 | 26.11 | 17.64 | 29.43 | 21.4 | 75.06 | 55.83 | 40.67 | 89.82 | 69.89 | BAZ1A |
| gene11950 | -1.95696615 | 0.000445331 | 0.34 | 0.33 | 0.16 | 0.33 | 0.37 | 0.44 | 0.16 | 0.1 | 0 | 0.07 | 0.06 | 0.17 | JPH4 |
| gene12022 | 1.112637335 | 0.003288208 | 179.16 | 96.88 | 74.71 | 41.41 | 40.04 | 71.89 | 77.49 | 147.63 | 93.52 | 90.06 | 197.08 | 153.48 | PNP |
| gene12195 | -1.18668521 | 0.002379723 | 5.19 | 3.58 | 7.91 | 6.8 | 7.03 | 5.73 | 2.21 | 4 | 2.08 | 4.4 | 5.52 | 8.82 | C7H14orf50 |
| gene12224 | 1.283901329 | 0.000698281 | 453.18 | 498.32 | 405.06 | 258.69 | 272.91 | 249.3 | 571.34 | 652.69 | 612.64 | 715.16 | 762.57 | 529.94 | ZFP36L1 |
| gene12237 | -1.576635586 | 5.41E-05 | 1.18 | 4.17 | 4.24 | 10.48 | 7.38 | 8.68 | 3.54 | 0.96 | 4.08 | 1.84 | 1.19 | 2.02 | SMOC1 |
| gene12248 | 1.543332955 | 7.01E-05 | 4.76 | 3.89 | 2.7 | 3.15 | 2.05 | 3.84 | 6.9 | 10.99 | 7.36 | 6.36 | 10.31 | 10.95 |  |
| gene12249 | 1.151224377 | 0.002651919 | 18.56 | 14.79 | 10.56 | 9.43 | 5.43 | 12.12 | 16.59 | 18.88 | 21.92 | 17.18 | 24.31 | 31.63 |  |
| gene12286 | -1.36522122 | 0.000380793 | 4.14 | 4 | 7.07 | 4.2 | 11.13 | 8.68 | 2.05 | 0.82 | 6.19 | 5.58 | 2.97 | 3 | LOC100514300 |
| gene12300 | 1.418022633 | 0.00019232 | 162.82 | 207.56 | 197.92 | 52.67 | 138.1 | 76.88 | 316.59 | 251.38 | 128.23 | 251.07 | 277.55 | 568.05 | FOS |
| gene12312 | -1.446735568 | 0.000670056 | 0.75 | 0.46 | 0.88 | 1.45 | 3.16 | 1.16 | 1.18 | 0.35 | 0.49 | 0.68 | 0.64 | 0.6 | ESRRB |
| gene12340 | -2.214151788 | 6.38E-08 | 7.7 | 5.88 | 4.64 | 3.47 | 5.7 | 30.22 | 1.76 | 3.62 | 2.81 | 8.25 | 4.72 | 3.44 | DIO2 |
| gene12341 | -1.274110901 | 0.003298787 | 11.95 | 13.35 | 7.64 | 6.21 | 8.56 | 44.99 | 6.04 | 7.21 | 10.56 | 17.63 | 19.1 | 7.75 | LOC100737350 |
| gene12343 | -1.403190665 | 0.008374201 | 1.51 | 1.27 | 0.25 | 3.19 | 2.39 | 1.5 | 0.45 | 1.38 | 0.98 | 1.07 | 1.78 | 0 | LOC100157061 |
| gene12388 | 3.093517316 | 2.36E-13 | 0.78 | 1.19 | 1.76 | 0.21 | 0.25 | 1.42 | 4.87 | 7.42 | 2.78 | 2.09 | 4.04 | 3.73 | LOC100512175 |
| gene12400 | -1.202122145 | 0.001553102 | 146.69 | 97.02 | 146.99 | 39.52 | 70.63 | 93.58 | 5.32 | 37.84 | 42.4 | 57.82 | 86.93 | 43.53 | CHGA |
| gene12418 | -1.217256512 | 0.002045272 | 11.62 | 10.2 | 16.54 | 9.41 | 20.83 | 5.82 | 4.17 | 5.53 | 5.57 | 15.13 | 8.61 | 9.42 | LOC100151873 |
| gene12420 | -1.598698606 | 2.86E-05 | 487.73 | 338.68 | 493.58 | 833.15 | 609.85 | 351.54 | 200.4 | 211.07 | 161.91 | 166.08 | 209.61 | 102.92 | ISG12(A) |
| gene12423 | -1.446650706 | 0.000221007 | 7.88 | 8.43 | 8.92 | 7.06 | 9.34 | 9.88 | 2.07 | 5.44 | 1.79 | 5.21 | 7.91 | 3.13 | LOC100620190 |
| gene12427 | -2.196712137 | 6.15E-07 | 1.49 | 0.58 | 1.82 | 0.19 | 5.9 | 1.63 | 0.16 | 1.38 | 0.1 | 2.92 | 2.62 | 0.58 | SERPINA1 |
| gene12429 | -1.461668785 | 0.000120862 | 183.69 | 965.76 | 2014 | 1365.4 | 2375 | 4515.9 | 2505.8 | 138.43 | 226 | 225.45 | 486.07 | 174.87 | UFBP |
| gene12435 | 1.385634416 | 0.001603138 | 7.59 | 0.05 | 10.34 | 0.22 | 1.73 | 0.96 | 4.26 | 1.6 | 1.23 | 4.27 | 0.99 | 0.9 | SERPINA3-1 |
| gene12437 | 2.657719997 | 8.24E-10 | 0.93 | 0.07 | 1.55 | 0.9 | 0.33 | 1.1 | 7.42 | 4.36 | 2.08 | 19.5 | 1.55 | 4.58 | SERPINA3-3 |
| gene12438 | -2.427350216 | 0.000835618 | 0.12 | 0.04 | 0.16 | 0.52 | 0 | 0.82 | 0.07 | 0.13 | 0 | 1.32 | 0 | 0 |  |
| gene12439 | 2.273963437 | 2.80E-08 | 0.89 | 0.04 | 1.48 | 1.48 | 2.34 | 1.47 | 18.12 | 3.53 | 2.95 | 17.14 | 3.41 | 13.3 | LOC100156325 |
| gene12440 | 1.680004755 | 1.18E-05 | 112.78 | 183.88 | 162.31 | 50.09 | 53.83 | 9.89 | 192.07 | 82.21 | 80.6 | 216.34 | 99.74 | 146.15 | SERPINA3-2 |
| gene12466 | -1.496148457 | 9.32E-05 | 38.64 | 32.5 | 65.48 | 30.1 | 70.81 | 32.1 | 14.95 | 15.67 | 15.31 | 28.26 | 15.98 | 18.94 | LOC100625046 |
| gene12538 | -1.940353444 | 8.49E-06 | 0.37 | 0.35 | 1.34 | 1.93 | 0.53 | 2.37 | 0.94 | 0.09 | 0 | 0.27 | 0.14 | 0.25 | LOC100624633 |
| gene126 | -1.047053773 | 0.007214255 | 7.26 | 5.96 | 7.16 | 7.21 | 9.32 | 5.43 | 2.76 | 3.94 | 3.65 | 4.67 | 5.64 | 3.85 | LRP11 |
| gene12600 | 2.114918254 | 0.001232432 | 0.03 | 0.04 | 0.35 | 0.1 | 0.14 | 0.04 | 0.02 | 0.66 | 0.56 | 0.33 | 0.32 | 0 | LOC100523076 |
| gene12668 | -1.015376219 | 0.008036017 | 3.78 | 6.28 | 8.73 | 7.26 | 11.75 | 10.29 | 5.86 | 4.12 | 4.01 | 4.6 | 4.46 | 7.7 | LOC100620681 |
| gene12670 | 2.61545378 | 0.001802496 | 0.16 | 0.01 | 0.05 | 0.04 | 0 | 0.02 | 0.1 | 0.18 | 0.15 | 0.23 | 0.11 | 0.17 | CRMP1 |
| gene12673 | -1.277170782 | 0.001088029 | 24.41 | 22.12 | 25.43 | 26.91 | 33.89 | 35.67 | 14.55 | 7.41 | 16.63 | 11.5 | 17.38 | 38.34 | LOC100621070 |
| gene12695 | 2.074228939 | 0.003248517 | 0.12 | 0.05 | 0 | 0 | 0.14 | 0.23 | 0.54 | 0.33 | 0.62 | 0.26 | 0.12 | 0.11 | LOC100523559 |
| gene12736 | -2.552003201 | 1.57E-06 | 0.46 | 0.14 | 0.71 | 0.47 | 0.29 | 0.32 | 0.05 | 0.08 | 0.04 | 0.19 | 0.1 | 0.04 |  |
| gene12738 | -1.184368307 | 0.008816036 | 2.35 | 5.38 | 5.27 | 4.31 | 5.59 | 7.42 | 3.31 | 2 | 1.54 | 0.96 | 3.82 | 0.63 | LOC100513483 |
| gene1279 | 1.359259733 | 0.001384532 | 3.22 | 3.42 | 3.19 | 2.18 | 0.92 | 0.79 | 1.32 | 4.84 | 3.36 | 3.68 | 2.09 | 4.3 | LOC100513515 |
| gene12856 | 1.591143794 | 0.000210761 |  |  |  |  |  |  |  |  |  |  |  |  |  |
| gene12882 | 2.193025099 | 0.009710715 |  |  |  |  |  |  |  |  |  |  |  |  |  |
| gene12917 | -1.068841427 | 0.004863796 | 8.56 | 8.29 | 10.76 | 9.3 | 33.81 | 11.96 | 11.55 | 8.49 | 5.58 | 8.89 | 6.51 | 9.97 | GUCY1A3 |
| gene12974 | -1.848928491 | 1.28E-05 | 3.7 | 114.41 | 2.12 | 1.88 | 13.05 | 9.33 | 2.22 | 2.05 | 2.29 | 5.87 | 7.22 | 4.78 | LOC100523263 |
| gene12979 | -1.212205844 | 0.00621963 | 8.55 | 12.91 | 19.55 | 11.87 | 11.92 | 19.32 | 6.2 | 7.25 | 4.27 | 12.43 | 8.91 | 14.06 | LOC100736838 |
| gene13005 | -1.040324092 | 0.009375658 | 2.03 | 3.02 | 3.58 | 3.45 | 3.55 | 4.17 | 2.88 | 0.9 | 1.41 | 2.8 | 0.71 | 1.38 | TMPRSS11A |
| gene13011 | -1.083399686 | 0.004278345 | 6.19 | 21.66 | 25.99 | 37.58 | 33.72 | 29.66 | 27.62 | 6.9 | 11.33 | 13.87 | 12.28 | 20.79 | LOC100513671 |
| gene13012 | -3.082959859 | 0.000437901 | 0.03 | 0.07 | 0 | 0.58 | 0 | 0.04 | 0.04 | 0 | 0 | 0.09 | 0.04 | 0 | TMPRSS11B |
| gene13020 | 2.29047758 | 0.008369487 | 0 | 0.04 | 0 | 0.03 | 0 | 0.07 | 0.1 | 0.13 | 0.29 | 0.01 | 0.19 | 0.14 | LOC100738495 |
| gene13027 | 1.598107203 | 0.000585954 | 1.04 | 0.61 | 0.16 | 0.39 | 0.28 | 0.48 | 0.53 | 0.92 | 1.98 | 2.43 | 0.98 | 1.26 | UGT2A1 |
| gene13041 | 1.454318503 | 0.000674806 | 9.5 | 17.03 | 11.97 | 2.49 | 3.31 | 6.85 | 9.44 | 7.99 | 15.32 | 18.31 | 11.25 | 12.33 | LOC100739482 |
| gene13042 | 1.012832989 | 0.009919306 | 3.21 | 5.5 | 3.43 | 0.79 | 1.34 | 2.41 | 2.4 | 3.93 | 2.43 | 6.57 | 3.05 | 3.41 | SLC4A4 |
| gene13043 | -3.120391851 | 1.73E-11 | 0.18 | 0.2 | 0.05 | 2.53 | 2.08 | 3.51 | 0.69 | 0.04 | 0 | 0 | 0 | 0.1 | LOC100739516 |
| gene1305 | 1.769115291 | 0.00010026 | 2.13 | 0.77 | 0.77 | 0.68 | 0.54 | 0.73 | 2.06 | 2.44 | 1.9 | 0.88 | 1.41 | 1.44 | LOC100519236 |
| gene13050 | -6.200820209 | 1.13E-33 | 0.1 | 0.23 | 0.25 | 0.88 | 6.12 | 26.56 | 0.27 | 0.07 | 0.11 | 84.56 | 0.13 | 0.27 | ALB |
| gene13061 | 1.546236155 | 0.00014785 | 14.09 | 24.59 | 14.96 | 5.46 | 7.6 | 5.07 | 11.26 | 29.8 | 10.03 | 14.39 | 25.5 | 19.44 | CXCL2 |
| gene13068 | -1.817491067 | 0.001815319 | 2.35 | 1.72 | 5.13 | 0.78 | 3.04 | 2.59 | 0.4 | 0.4 | 1.15 | 0.92 | 0.74 | 0.94 | LOC100737222 |
| gene13075 | 1.306019034 | 0.007859988 | 1.64 | 0.48 | 0.97 | 0.21 | 0.63 | 1.86 | 1.51 | 2.47 | 2.34 | 2.77 | 1.64 | 1.04 | LOC100511764 |
| gene13088 | -4.096486017 | 0.000115349 | 0 | 0.05 | 0.11 | 0.32 | 0.16 | 0.24 | 0.02 | 0 | 0 | 0.13 | 0 | 0.08 | LOC100512296 |
| gene1309 | -1.946167071 | 6.61E-06 | 6.18 | 6.51 | 0.15 | 0 | 15.44 | 3.97 | 4.14 | 0 | 0.2 | 0 | 0 | 0 | MNAT1 |
| gene13109 | -1.480420815 | 0.000890226 | 1.48 | 2.26 | 2.4 | 1.61 | 2.83 | 5.29 | 0.92 | 1.38 | 0.96 | 2.22 | 2.42 | 0.63 | LRAT |
| gene13111 | 2.099558674 | 0.000194542 | 0.09 | 0.21 | 0.27 | 0.1 | 0.39 | 0 | 1.89 | 0 | 0 | 2.06 | 0.05 | 0.12 | FGG |
| gene13144 | 1.568665143 | 0.004350094 | 3.57 | 2.67 | 2.45 | 0.85 | 1.65 | 1.11 | 4.05 | 2.8 | 3.46 | 1.45 | 2.53 | 3.59 | LOC100620683 |
| gene13193 | -2.139886229 | 0.000352117 | 0.33 | 0.47 | 0.19 | 0.22 | 0.25 | 2.22 | 0.15 | 0.08 | 0.38 | 0.24 | 0.12 | 0.26 | LOC100512242 |
| gene13194 | -1.754749747 | 5.46E-06 | 10.61 | 24.61 | 7.81 | 9.63 | 13.58 | 60.4 | 8.76 | 8.53 | 6.28 | 12.05 | 17.33 | 18.4 | CLGN |
| gene13296 | 1.400116813 | 0.001099916 | 0.46 | 0.45 | 0.61 | 1.05 | 0.69 | 0.74 | 0.39 | 4.06 | 1.84 | 0.62 | 0.43 | 0.5 | NDST4 |
| gene13321 | -1.72719042 | 7.54E-06 | 23.26 | 18.86 | 8.8 | 13.58 | 16.43 | 52.05 | 6.7 | 11.29 | 5.71 | 11.85 | 9.54 | 7.89 | LOC100516921 |
| gene13327 | -1.052275783 | 0.005213297 | 67.96 | 11.13 | 82.67 | 222.25 | 554.77 | 178.11 | 374.38 | 44.68 | 29.8 | 15.03 | 24.01 | 27.48 | LOC100519324 |
| gene13330 | -1.777703635 | 5.89E-06 |  |  |  |  |  |  |  |  |  |  |  |  |  |
| gene1335 | -1.238351446 | 0.001170438 | 1.96 | 0.63 | 1.94 | 7.21 | 26.26 | 5.59 | 12.12 | 2.63 | 1.33 | 0.62 | 1.05 | 0.58 | ESR2 |
| gene13367 | 2.561027721 | 0.000835618 | 0.09 | 0 | 0.12 | 0.2 | 0.11 | 0.27 | 1.63 | 0.1 | 1.29 | 5.7 | 1.6 | 4.09 | UBE2D2 |
| gene13376 | -1.125030336 | 0.005009563 | 4.94 | 5.85 | 3.47 | 5.05 | 18.18 | 13.86 | 10.16 | 3.88 | 1.92 | 5.8 | 2.6 | 4.33 | LOC100628129 |
| gene13379 | -2.678072829 | 1.11E-10 | 15.08 | 18.59 | 9.91 | 8.78 | 35.55 | 118.16 | 17.31 | 4.11 | 1.38 | 4.27 | 5.4 | 1.88 | LOC100620154 |
| gene13401 | 2.221403646 | 1.89E-05 | 0.43 | 0.56 | 0.21 | 0.44 | 0.49 | 0.23 | 1.48 | 1.88 | 1.96 | 0.7 | 0.58 | 1.07 | LOC100737772 |
| gene13418 | -1.405330025 | 0.000286587 | 0.17 | 5.68 | 4.1 | 0.94 | 1.72 | 5.16 | 2.3 | 0.12 | 0.33 | 0.3 | 0.08 | 0.1 | MMRN1 |
| gene13428 | -1.347878341 | 0.000526699 | 13.47 | 17.7 | 12.08 | 30.37 | 15.41 | 33.05 | 12.09 | 9.81 | 7.72 | 12.24 | 9.07 | 5 | LOC100518083 |
| gene13429 | -3.314977835 | 4.72E-05 | 0.34 | 0.33 | 0.46 | 0.46 | 1.07 | 0.28 | 0 | 0.1 | 0.09 | 0.4 | 0.23 | 0 | LOC100739395 |
| gene13435 | -1.009323631 | 0.007306739 | 48621 | 33779 | 50794 | 31893 | 66258 | 67376 | 49481 | 13274 | 16602 | 18696 | 22008 | 21711 | SPP1 |
| gene13468 | 1.337040552 | 0.000432171 | 36.28 | 62.26 | 65.36 | 41.15 | 34.46 | 31.55 | 31.6 | 148.5 | 81.88 | 87.03 | 43.64 | 49.41 | HPSE |
| gene13479 | -1.010557233 | 0.009415994 | 5.9 | 3.77 | 10.24 | 14.89 | 7.24 | 9.13 | 2.29 | 9.13 | 3.58 | 6.07 | 5.17 | 4.91 | SCD5 |
| gene13488 | -1.559836938 | 0.000409845 | 0.45 | 1.02 | 0.74 | 2.63 | 0.64 | 2.18 | 0.64 | 0.48 | 0.69 | 0.75 | 0.42 | 0.19 | BMP3 |
| gene1353 | -1.388453476 | 0.003113289 | 1.43 | 1.58 | 1 | 1.53 | 1.44 | 2.37 | 1.15 | 0.37 | 0.32 | 0.2 | 0.39 | 0.48 | LOC100513000 |
| gene13533 | -1.887980722 | 0.000108278 | 0.25 | 0.55 | 0.46 | 0.92 | 1.26 | 0.61 | 0.34 | 0.13 | 0.26 | 0.16 | 0.31 | 0.64 | TUB |
| gene13567 | -1.279726308 | 0.001803865 | 0.67 | 0.19 | 1.01 | 1.29 | 1.56 | 2.4 | 1.16 | 0.6 | 0.21 | 0.7 | 0.75 | 1.17 | LOC100622122 |
| gene13573 | -2.331855021 | 0.000305042 | 0.06 | 0.17 | 0.28 | 0.41 | 0.35 | 0.6 | 0.09 | 0.09 | 0.06 | 0.05 | 0.05 | 0.08 |  |
| gene13603 | -1.702034763 | 2.76E-05 | 3.92 | 1.74 | 7.62 | 4.83 | 8.98 | 6.34 | 1.46 | 1.41 | 3.31 | 3.65 | 5.63 | 5.34 | HPX |
| gene13605 | -1.541389431 | 5.75E-05 | 18.52 | 8 | 26.91 | 24.21 | 44.04 | 21.56 | 11.84 | 8.4 | 9.76 | 9.31 | 8.16 | 11.1 | LOC100519163 |
| gene13612 | -1.815617283 | 0.000390665 | 0.46 | 0.41 | 0.65 | 0.65 | 0.97 | 0.58 | 0.11 | 0.34 | 0.16 | 0.48 | 0.43 | 0.24 | LOC100519325 |
| gene13613 | 2.510260587 | 0.001179647 | 0.06 | 0.11 | 0.15 | 0.14 | 0.14 | 0 | 0.38 | 0.74 | 0.72 | 0.76 | 0.46 | 0.5 | LOC100625698 |
| gene1362 | 1.481013642 | 0.001671752 | 1.99 | 4.54 | 2.14 | 3.58 | 2.79 | 1.18 | 3.34 | 9.24 | 8.24 | 5.26 | 9.09 | 4.53 | P14ARF |
| gene13699 | 2.501501484 | 0.005683782 | 0.08 | 0.02 | 0 | 0.05 | 0.04 | 0.06 | 0.4 | 0.36 | 0 | 0 | 0.24 | 0.22 | LOC100625080 |
| gene13781 | 5.761099717 | 3.39E-05 | 0 | 0 | 0 | 0 | 0 | 0 | 0.54 | 0.12 | 0.21 | 0.13 | 0 | 0 | LOC100518085 |
| gene13839 | -2.458716797 | 1.09E-09 | 8.16 | 2.56 | 18.75 | 10.26 | 36.5 | 17.7 | 4.79 | 3.27 | 3.37 | 6.96 | 2.28 | 3.15 | LOC100513029 |
| gene13840 | -3.096937459 | 4.12E-14 | 12.68 | 2.37 | 29.43 | 24.58 | 58.41 | 33.24 | 5.4 | 4.8 | 2.96 | 3.84 | 1.26 | 3.25 | P2RY6 |
| gene13901 | -1.176789303 | 0.003355794 | 0.37 | 0.63 | 0.64 | 0.5 | 0.55 | 0.8 | 0.31 | 0.39 | 0.06 | 0.33 | 0.29 | 0.45 | MYO7A |
| gene13933 | 1.842508965 | 0.006825417 |  |  |  |  |  |  |  |  |  |  |  |  |  |
| gene13935 | 2.358867465 | 6.40E-06 | 0.12 | 0.21 | 0.12 | 0.1 | 0.24 | 0.3 | 0.83 | 1.4 | 0.99 | 0.9 | 0.75 | 0.76 | LOC100517667 |
| gene13944 | -2.07529398 | 0.001666087 | 0.68 | 0.53 | 0.35 | 0.24 | 0.79 | 1.42 | 0.17 | 0.11 | 0.27 | 0.84 | 0.41 | 1.04 | LOC100516384 |
| gene13976 | 1.204662491 | 0.004671679 |  |  |  |  |  |  |  |  |  |  |  |  |  |
| gene14013 | -2.29844163 | 4.00E-07 | 4.78 | 6.23 | 14.38 | 0.99 | 0 | 4.57 | 0.02 | 0.93 | 0.08 | 0.68 | 1.06 | 1.37 | LOC100513220 |
| gene14029 | -1.044561575 | 0.007427008 | 6.8 | 3.02 | 3.69 | 4.09 | 10.06 | 8.51 | 3.8 | 4.68 | 2.07 | 2.78 | 3.97 | 1.59 | SESN3 |
| gene1404 | 3.16147494 | 0.000541295 | 0.13 | 0 | 0.36 | 0 | 0.16 | 0 | 1.34 | 0 | 0.34 | 0.74 | 0.2 | 1.01 | IFN-OMEGA-2 |
| gene14040 | 1.874251239 | 0.002390204 | 1.24 | 1.03 | 0.86 | 0.52 | 0.41 | 0.13 | 1.62 | 1.51 | 0.77 | 1.59 | 0.46 | 1.61 |  |
| gene14054 | -1.081240317 | 0.004257294 | 63.5 | 50.91 | 47.69 | 40.78 | 77.05 | 77.24 | 23.53 | 41.29 | 24.38 | 40.14 | 37.24 | 30.05 | LOC100622759 |
| gene14055 | 2.033393206 | 1.68E-07 | 80.42 | 110.76 | 182.76 | 125.77 | 5.25 | 39.71 | 64.66 | 497.41 | 107.98 | 187.66 | 154.47 | 70.24 | MMP7 |
| gene14058 | -1.587608291 | 4.38E-05 | 2.45 | 16.51 | 2.91 | 10.47 | 15.58 | 1.98 | 6.66 | 1.29 | 1.02 | 16.45 | 2.59 | 1.74 | MMP8 |
| gene14075 | -1.148906364 | 0.005472038 | 1.35 | 1.12 | 1.32 | 4.18 | 1.11 | 2.97 | 0.94 | 1.86 | 0.74 | 1.23 | 1.2 | 0.81 | CASP1 |
| gene14085 | -1.705406257 | 0.000550443 | 2.74 | 0.83 | 0.66 | 0.32 | 1.86 | 0.61 | 0.5 | 0.16 | 0.13 | 1.32 | 0.67 | 0.77 | LOC100626413 |
| gene14134 | 1.388500597 | 0.000274953 | 40.45 | 55.6 | 82.12 | 48.08 | 28.43 | 47.87 | 60.76 | 110.85 | 141.01 | 62.24 | 110.24 | 158.77 | IL18 |
| gene14142 | 2.082742596 | 1.17E-07 | 1.21 | 5.51 | 3.98 | 2.65 | 3.43 | 3.74 | 4.26 | 2.22 | 33.58 | 1.86 | 3.05 | 4.53 | LOC100515564 |
| gene14153 | -3.305927411 | 1.05E-06 | 0.21 | 0.19 | 0.69 | 2.4 | 0.12 | 5.54 | 0.17 | 0.11 | 0.55 | 0.34 | 0.49 | 0.15 | NNMT |
| gene14158 | -1.150175127 | 0.003548845 |  |  |  |  |  |  |  |  |  |  |  |  |  |
| gene14165 | -1.656818004 | 0.000422199 | 0.23 | 0.5 | 0.31 | 0.74 | 0.11 | 2.14 | 0.66 | 0.03 | 0.06 | 4.56 | 0.1 | 1.04 | APOA4 |
| gene14166 | -2.611887111 | 0.003867182 | 0 | 0 | 0.45 | 0.43 | 0.85 | 2.24 | 0.07 | 0.54 | 0 | 10.19 | 0 | 0.75 | APOC3 |
| gene14180 | -1.858773316 | 0.000910717 | 0.39 | 2.66 | 2.71 | 1.64 | 5.28 | 3.24 | 1.85 | 0.14 | 0.33 | 2.18 | 0.88 | 0.55 | FXYD2 |
| gene14226 | -2.774829173 | 0.000541295 | 0.04 | 0.05 | 0.09 | 0.05 | 0.15 | 0.39 | 0.02 | 0.05 | 0 | 0.11 | 0.07 | 0.06 | MFRP |
| gene14227 | -1.217597011 | 0.001552094 | 5.04 | 2.56 | 5.47 | 5.63 | 11.94 | 3.2 | 5.01 | 2.12 | 1.52 | 2.05 | 3.3 | 4 | LOC100520041 |
| gene14244 | 2.033289615 | 9.12E-06 | 0.16 | 0.48 | 0.17 | 0.32 | 0.48 | 0.93 | 1.25 | 3.64 | 1.63 | 1.85 | 2.48 | 1.3 | LOC100521388 |
| gene14369 | -1.12144932 | 0.003412145 | 1.74 | 0.36 | 2.24 | 7.58 | 9.72 | 3.32 | 6.12 | 0.99 | 2.05 | 2.23 | 1.2 | 1.31 | KIRREL3 |
| gene14373 | 2.594435467 | 1.49E-10 | 15.97 | 38.16 | 5.4 | 3.86 | 1.43 | 6.72 | 2.31 | 44.68 | 22.04 | 43.52 | 26.55 | 56.23 | KCNJ1 |
| gene14377 | 1.101210509 | 0.009165734 | 1.93 | 2.59 | 1.41 | 1.89 | 1.25 | 0.93 | 1.7 | 3.82 | 2.87 | 2.4 | 2.52 | 2.86 | LOC100737798 |
| gene14427 | -1.286472398 | 0.000710103 | 22.86 | 28.66 | 31.82 | 67.66 | 78.78 | 72.56 | 39.31 | 21.54 | 25.75 | 53.2 | 32.07 | 40.26 | LOC100526237 |
| gene14430 | -1.12075762 | 0.007089826 | 1.67 | 1.2 | 1.26 | 1.73 | 1 | 5 | 0.84 | 1.61 | 0.9 | 0.77 | 1.36 | 1.06 | LOC100511526 |
| gene14449 | 1.195929793 | 0.008093442 | 0.19 | 0.17 | 0.24 | 0.23 | 0.24 | 0.15 | 0.27 | 0.65 | 0.51 | 0.39 | 0.18 | 0.32 | LOC100626921 |
| gene14461 | -1.209138295 | 0.001779063 | 4.08 | 3.01 | 7.25 | 7.46 | 9.02 | 4.24 | 2.67 | 4.33 | 1.71 | 8.86 | 2.3 | 2.56 | SLC45A3 |
| gene14463 | -1.220655307 | 0.003790078 | 4.02 | 3.71 | 4.28 | 5.55 | 5.58 | 2.77 | 2.39 | 1.7 | 1.63 | 5.07 | 2.68 | 1.95 | LOC100626414 |
| gene14470 | 1.189332444 | 0.007074091 | 1.79 | 8.2 | 3.92 | 1.91 | 2.23 | 1.3 | 5.34 | 2.71 | 3.96 | 2.34 | 3.27 | 0.99 | LOC100518270 |
| gene14488 | -2.480255884 | 0.000154471 | 0.25 | 0.42 | 0.33 | 1.31 | 0.43 | 0.71 | 0.3 | 0.06 | 0 | 0 | 0.29 | 0.18 | LOC100739302 |
| gene14493 | 1.23139499 | 0.001663621 | 26.83 | 7.8 | 8.6 | 2.13 | 3.21 | 8.53 | 9.42 | 10.05 | 11.54 | 8.08 | 22.5 | 25.83 | YOD1 |
| gene14501 | 1.876480217 | 2.55E-06 | 0.46 | 0.7 | 0.68 | 1.28 | 0.73 | 1.59 | 5.47 | 5.28 | 1.8 | 3.24 | 5.16 | 3.68 | CR2 |
| gene14507 | -1.6253121 | 3.25E-05 | 6.57 | 18.66 | 9.49 | 6.05 | 12.33 | 11.54 | 3.41 | 4.25 | 1.67 | 5.99 | 2.29 | 2.17 | STEAP4 |
| gene14515 | 2.444791249 | 3.40E-09 | 11.43 | 27.93 | 12.88 | 3.38 | 3.92 | 4.3 | 2.48 | 44.91 | 13.77 | 25.22 | 5.3 | 13.1 | LOC100518208 |
| gene14549 | -1.067426031 | 0.004613879 | 129.93 | 131.44 | 167.4 | 210.85 | 207.6 | 279.08 | 130.55 | 103.32 | 86.49 | 168.04 | 112.35 | 127.3 | COL1A2 |
| gene1455 | 1.110856144 | 0.003879623 | 22.36 | 29.02 | 17.71 | 13.22 | 18.09 | 14.71 | 30.29 | 31.13 | 34.83 | 29.64 | 25.85 | 28.51 | LOC100737553 |
| gene14566 | 1.586094663 | 9.89E-05 | 0.88 | 1.11 | 0.39 | 1.3 | 2.66 | 0.85 | 5.12 | 4.68 | 4.14 | 2.96 | 2.11 | 3.08 | LOC100523505 |
| gene14624 | 3.116627917 | 0.007364993 | 0 | 0.09 | 0.19 | 0.08 | 0 | 0 | 0.23 | 0.15 | 0.74 | 0 | 0 | 0.3 | LOC100519327 |
| gene14660 | -1.41284787 | 0.000273663 | 4.22 | 3.18 | 4.58 | 5.75 | 3.69 | 9.51 | 2.57 | 2.17 | 2.07 | 2.32 | 3.99 | 1.76 | LOC100523199 |
| gene14691 | -1.173352686 | 0.004233274 | 1.41 | 1.14 | 4.23 | 4.82 | 3.25 | 9.57 | 2.05 | 2.85 | 2.59 | 2.23 | 2.32 | 1.4 | LOC100737774 |
| gene1470 | 1.083107781 | 0.007261618 | 2.38 | 0.53 | 1.05 | 0.77 | 0.57 | 1.46 | 1.97 | 3.07 | 0.56 | 2.38 | 3.02 | 0.32 | FREM1 |
| gene14710 | 1.734658048 | 0.002838964 | 0.68 | 0.12 | 0.26 | 0 | 1.01 | 0.42 | 2.32 | 0.2 | 2.55 | 0.48 | 2.31 | 0.56 | LOC100738576 |
| gene14720 | 1.249984627 | 0.001345947 | 30.3 | 28.2 | 23.91 | 27.7 | 37.42 | 19.08 | 58.17 | 67.78 | 68.07 | 54.18 | 84.13 | 66.9 | LOC100517670 |
| gene1473 | 1.171274151 | 0.00730374 | 51.54 | 9.93 | 16.09 | 15.46 | 6.67 | 30.03 | 55.36 | 36.95 | 16.58 | 25.65 | 45.44 | 8.29 |  |
| gene14743 | -1.265700744 | 0.00115552 | 1.44 | 1.02 | 0.98 | 0.65 | 1 | 3.96 | 1.76 | 0.09 | 0.3 | 0.85 | 0.82 | 0.39 | NRCAM |
| gene14751 | -1.414319878 | 0.000220933 | 3.17 | 15.18 | 11.35 | 32.15 | 6.72 | 19.47 | 2.73 | 14.69 | 3.59 | 4.39 | 5.44 | 2.31 | AMPH |
| gene14761 | -1.304287683 | 0.001409273 | 3.26 | 3.2 | 3.81 | 3.3 | 4.48 | 10.09 | 4.23 | 1.37 | 0.89 | 4.84 | 3.99 | 3.02 | LOC100625132 |
| gene14778 | 3.006371579 | 0.000814644 | 0 | 0.02 | 0.05 | 0.1 | 0 | 0 | 0 | 0.77 | 0.14 | 0.46 | 0.06 | 0 | LOC100511653 |
| gene14784 | 1.316237322 | 0.00563512 | 0.29 | 0.18 | 0.07 | 0.44 | 0.37 | 0.39 | 0.81 | 1.12 | 0.98 | 0.87 | 0.34 | 0.54 | LOC100513809 |
| gene14830 | -1.831905592 | 2.01E-06 | 4.26 | 13.93 | 8.27 | 13.64 | 33.14 | 17.04 | 12.93 | 0.97 | 3.45 | 4.32 | 2.71 | 4.1 | TNN |
| gene14832 | 2.03080683 | 0.009097428 | 0.02 | 0.1 | 0.07 | 0.02 | 0 | 0.05 | 0.13 | 0.1 | 0.05 | 0.02 | 0.01 | 0.03 | TNR |
| gene14840 | 4.667221308 | 0.00659106 |  |  |  |  |  |  |  |  |  |  |  |  |  |
| gene14848 | 1.039478113 | 0.008992489 | 5.13 | 11.04 | 7.54 | 4.94 | 8.02 | 6.16 | 13.27 | 8.58 | 16.1 | 16.04 | 7.78 | 10.7 | LOC100737775 |
| gene14849 | 1.317669426 | 0.000804742 | 1.78 | 2.35 | 2.6 | 1.62 | 1.41 | 1.69 | 3.21 | 2.7 | 5.46 | 5.32 | 2.6 | 6.73 | LOC100737809 |
| gene14851 | 1.58202804 | 9.54E-05 | 2.91 | 4.7 | 6.47 | 2.21 | 2.3 | 2.82 | 4.33 | 5.08 | 11.59 | 8.41 | 7.89 | 10.67 | RALGPS2 |
| gene14879 | 1.482558612 | 0.000299654 | 0.29 | 0.45 | 0.19 | 0.19 | 0.46 | 0.28 | 0.99 | 1 | 0.5 | 0.74 | 0.36 | 0.71 |  |
| gene14921 | 1.005118833 | 0.007630224 | 187.21 | 338.97 | 253.29 | 85.49 | 54.84 | 58.97 | 58.29 | 181.77 | 145.75 | 216.51 | 131.18 | 122.54 | PGHS-2 |
| gene14970 | -1.580064159 | 0.002558834 | 0.6 | 0.37 | 0.8 | 1.29 | 1.22 | 1.66 | 0.48 | 0.4 | 0.38 | 1.16 | 0.09 | 0.69 | SYT14 |
| gene14985 | -1.781321353 | 0.003543422 | 0.34 | 0.27 | 0.33 | 0.12 | 0.3 | 0.24 | 0.1 | 0 | 0.07 | 0.17 | 0.02 | 0.26 | LOC100736916 |
| gene14986 | -1.064880975 | 0.00564175 | 3.44 | 3.51 | 6.85 | 2.9 | 5.2 | 5.01 | 2.16 | 2.3 | 1.59 | 3.7 | 3.75 | 3.79 |  |
| gene14990 | -2.71967113 | 2.96E-11 | 1.46 | 3.33 | 4.05 | 2.76 | 4.75 | 27.59 | 4.12 | 0.41 | 0.2 | 0.32 | 1.03 | 0.55 | LOC100515158 |
| gene15002 | 1.64153777 | 0.001056438 | 2.32 | 1.78 | 0.77 | 1.14 | 2.69 | 2.52 | 7.49 | 5.5 | 5.76 | 2.85 | 4.29 | 3.06 | LOC100737649 |
| gene15018 | -2.572303876 | 6.49E-06 | 0.35 | 1.85 | 0.65 | 6.17 | 3.88 | 0.63 | 0.59 | 0.94 | 0 | 4.84 | 0.99 | 3.13 | LOC100628072 |
| gene15019 | -2.606643035 | 0.001179647 | 0.09 | 0.15 | 0.32 | 0 | 1.96 | 5.75 | 0.39 | 0.46 | 0.43 | 1.19 | 1.53 | 1.04 | LOC100738451 |
| gene15028 | 5.111184197 | 0.000714555 |  |  |  |  |  |  |  |  |  |  |  |  |  |
| gene15044 | 1.661178869 | 9.08E-05 | 24.92 | 6.34 | 4.1 | 3.11 | 1.34 | 2.73 | 5.99 | 11.05 | 4.04 | 4.54 | 2.67 | 4.83 | LOC100622556 |
| gene15064 | -1.14098009 | 0.002914851 | 80.45 | 100.67 | 84.8 | 93.03 | 107.77 | 118.98 | 24.18 | 73.87 | 42.3 | 44.02 | 47.32 | 8.76 | LOC100517050 |
| gene15088 | -1.251186678 | 0.002028598 | 1.48 | 3.21 | 2.13 | 2.23 | 2.16 | 4.77 | 1.39 | 1.38 | 0.88 | 4.41 | 1.53 | 2.24 | KMO |
| gene15089 | 2.145112038 | 0.004017409 |  |  |  |  |  |  |  |  |  |  |  |  |  |
| gene15160 | 2.109943437 | 1.83E-06 |  |  |  |  |  |  |  |  |  |  |  |  |  |
| gene15200 | 1.452866325 | 0.004332376 | 0.04 | 1.04 | 0.94 | 0.72 | 0.14 | 0.3 | 1.03 | 0.45 | 1.58 | 1.5 | 0.07 | 2.58 | LOC100622450 |
| gene15205 | -1.392256485 | 0.00046163 | 0.74 | 0.12 | 0.79 | 0.96 | 4.71 | 0.82 | 1.81 | 0.14 | 0.39 | 0.36 | 0.35 | 0.24 |  |
| gene15214 | 1.86865207 | 0.006825417 | 0.08 | 0.05 | 0.05 | 0.06 | 0.02 | 0.04 | 0.08 | 0.22 | 0.12 | 0.06 | 0.06 | 0 | LGR6 |
| gene15217 | -1.224543984 | 0.001626714 | 12.41 | 12.79 | 18.63 | 27.62 | 16.84 | 28.75 | 12.95 | 7.68 | 9.4 | 11.96 | 6.24 | 16.44 | LOC100739704 |
| gene15228 | 1.51864484 | 0.003690038 | 1.61 | 1.83 | 2.88 | 1.88 | 0.76 | 0.4 | 4.19 | 1.91 | 1.75 | 1.35 | 0.73 | 2.94 | SLC35D2 |
| gene15233 | 4.603986202 | 0.00659106 |  |  |  |  |  |  |  |  |  |  |  |  |  |
| gene15272 | 3.164221813 | 0.007364993 |  |  |  |  |  |  |  |  |  |  |  |  |  |
| gene15275 | -1.965209959 | 1.45E-05 | 0.21 | 3.03 | 2.82 | 2.55 | 3.18 | 8.95 | 2.63 | 0 | 0.65 | 0.19 | 0 | 0 | CCL21 |
| gene15290 | 2.046594717 | 1.08E-06 | 0.22 | 0.42 | 0.18 | 0.36 | 0.34 | 0.39 | 1.06 | 1.96 | 1.33 | 1.31 | 1.49 | 1.02 | KIF24 |
| gene15313 | -1.218185712 | 0.001566188 | 3.85 | 3.25 | 2.26 | 9.03 | 4.05 | 7.59 | 3.33 | 2.46 | 2.72 | 4.16 | 1.48 | 2.91 | DDX58 |
| gene15331 | -1.009897496 | 0.009710747 | 13.49 | 9.88 | 16.63 | 10.35 | 8.85 | 11.45 | 4.05 | 7.18 | 3.42 | 10.79 | 5.21 | 9.59 | BAMBI |
| gene15390 | 1.338271529 | 0.001752815 | 2.41 | 2.5 | 2.06 | 2.19 | 1.18 | 1.4 | 2.19 | 7.1 | 2.25 | 2.88 | 4.18 | 1.54 | LOC100513036 |
| gene15414 | 1.068979641 | 0.008858945 | 0.06 | 0.79 | 0.23 | 0.12 | 0.92 | 0.14 | 0.22 | 1.85 | 0.35 | 0.14 | 0.21 | 0.15 | MYO3A |
| gene15424 | 3.341379471 | 1.02E-08 | 5.52 | 2.23 | 1.69 | 0.17 | 1.01 | 0.26 | 5.25 | 3.02 | 4.86 | 2.81 | 5.34 | 1.15 | LOC100519970 |
| gene15426 | 1.690887897 | 0.000652877 | 0.4 | 0.26 | 0.06 | 0.41 | 0.04 | 0.32 | 1.35 | 0.52 | 0.39 | 0.73 | 0.58 | 0.22 | LOC100623853 |
| gene15432 | -2.085963086 | 4.00E-05 | 2.42 | 10.85 | 3.34 | 14.18 | 3.53 | 6.52 | 1.35 | 2.48 | 2.13 | 0.69 | 0.33 | 0 | LOC100520629 |
| gene15443 | -1.999849112 | 0.002889855 |  |  |  |  |  |  |  |  |  |  |  |  |  |
| gene15480 | 1.442863919 | 0.000150355 | 96.94 | 93.89 | 63.33 | 55.13 | 48.9 | 40.8 | 68.58 | 124.3 | 187.3 | 108.9 | 195.69 | 352.03 | LOC100516289 |
| gene15493 | -2.872589427 | 8.30E-12 | 14.56 | 14.89 | 17.17 | 24.41 | 30.34 | 11.32 | 4.53 | 1.44 | 2.66 | 1.73 | 1.92 | 5.01 | LOC100737810 |
| gene15495 | -2.342341876 | 0.000102488 | 0.09 | 0.04 | 0.33 | 0.24 | 0.96 | 0.04 | 0.16 | 0 | 0.04 | 0 | 0 | 0 | LOC100517730 |
| gene15500 | 4.020458835 | 1.01E-20 | 3.47 | 19.82 | 2.29 | 2.12 | 3.28 | 2.74 | 6.86 | 109.9 | 11.17 | 162.25 | 114.77 | 6.52 | LOC733634 |
| gene15501 | -3.417631209 | 0.004537517 |  |  |  |  |  |  |  |  |  |  |  |  |  |
| gene15515 | 1.384571522 | 0.000650382 |  |  |  |  |  |  |  |  |  |  |  |  |  |
| gene1562 | -2.216107861 | 1.74E-08 | 32.92 | 37.65 | 8.26 | 7.34 | 10.03 | 125.16 | 13.29 | 9.41 | 6.17 | 16.05 | 7.47 | 7.64 | LOC100517137 |
| gene15620 | 1.005105272 | 0.009317665 | 0.62 | 0.92 | 1.42 | 1.37 | 1.86 | 1.53 | 0.91 | 1.94 | 6.43 | 7.8 | 2.53 | 0.63 | ATP8A2 |
| gene15716 | -1.638470439 | 0.000233338 | 0.33 | 0.38 | 0.85 | 0.75 | 1 | 1.67 | 0.34 | 0.45 | 0.26 | 1.28 | 0.88 | 0.37 | NEK5 |
| gene15725 | -1.326458791 | 0.00152667 | 0.76 | 1.07 | 0.84 | 0.83 | 1.71 | 2.58 | 0.81 | 0.82 | 0.26 | 0.83 | 0.62 | 0.28 | LOC100152644 |
| gene15737 | -1.785161525 | 0.000177842 | 4.02 | 5.93 | 9.26 | 11.38 | 9.44 | 9.76 | 1.95 | 4.44 | 2.48 | 2.32 | 2.19 | 7.15 | LOC100738038 |
| gene15757 | -1.079481424 | 0.006551206 | 3.31 | 2.86 | 3.85 | 5.52 | 5.43 | 5.3 | 1.96 | 3.81 | 1.62 | 3.57 | 2.64 | 2.25 | LOC100522757 |
| gene15804 | 2.095301042 | 0.00010707 |  |  |  |  |  |  |  |  |  |  |  |  |  |
| gene15829 | 3.478588457 | 0.002810632 |  |  |  |  |  |  |  |  |  |  |  |  |  |
| gene15830 | 4.987878112 | 0.002125234 |  |  |  |  |  |  |  |  |  |  |  |  |  |
| gene15852 | 1.258949212 | 0.001762705 |  |  |  |  |  |  |  |  |  |  |  |  |  |
| gene15863 | 1.119276436 | 0.003027748 | 119.37 | 282.16 | 158.01 | 160.42 | 182.88 | 158.57 | 422.41 | 186.52 | 442.63 | 122.84 | 303.68 | 355.69 | LOC100525833 |
| gene15879 | 3.447838099 | 2.08E-14 | 3.24 | 5.74 | 2.58 | 6.47 | 6.72 | 3.27 | 67.72 | 61.11 | 48.17 | 52.17 | 38.84 | 31.08 | LOC100737161 |
| gene15882 | -2.132403051 | 5.48E-06 | 0.04 | 0.12 | 0.62 | 0.56 | 0.83 | 1.5 | 0.19 | 0.2 | 0.29 | 0.22 | 0.19 | 0.45 | SLITRK6 |
| gene15906 | 1.462666997 | 0.001779182 | 0.09 | 0.63 | 0.36 | 0.35 | 0.17 | 0.22 | 0.38 | 0.73 | 0.82 | 0.59 | 0.78 | 0.76 | LOC100620159 |
| gene15934 | 2.114874553 | 0.009710715 |  |  |  |  |  |  |  |  |  |  |  |  |  |
| gene15941 | -2.455363293 | 3.69E-06 | 0.08 | 0.09 | 0.05 | 0.33 | 0.21 | 0.32 | 0.07 | 0.04 | 0.02 | 0.02 | 0 | 0.01 | LOC100524825 |
| gene15970 | -1.851436157 | 0.000339282 | 0.44 | 1.55 | 1.73 | 2.9 | 2.14 | 2.3 | 0.9 | 0.7 | 0.45 | 2.21 | 1.5 | 1.69 | LOC100516692 |
| gene15981 | -1.834659121 | 3.15E-06 | 85.18 | 70.14 | 44.57 | 98.17 | 52.85 | 52.48 | 23.45 | 15.47 | 15.79 | 12.97 | 6.35 | 14.22 | LOC100620225 |
| gene15988 | 1.583756432 | 0.000149873 | 2.39 | 2.38 | 1.5 | 1.94 | 1.8 | 0.82 | 3.61 | 4.11 | 5.62 | 2.47 | 4.04 | 6.84 | ATP4B |
| gene15989 | 2.425280594 | 0.005683782 | 0.02 | 0 | 0.06 | 0 | 0 | 0.1 | 0.26 | 0.14 | 0.04 | 0.07 | 0 | 0.1 | LOC100620646 |
| gene16021 | -1.253352851 | 0.000968984 | 20.11 | 12.56 | 19.99 | 10.71 | 36.66 | 16.3 | 9.34 | 6.61 | 10.1 | 12.71 | 9.06 | 10.34 | FASN |
| gene16066 | 5.382187257 | 0.000249756 | 0.16 | 0.22 | 0.27 | 0 | 0 | 0 | 0.2 | 0.3 | 0.28 | 0.13 | 0.06 | 0.23 | LOC100738453 |
| gene1608 | -1.765101575 | 6.86E-05 | 0.25 | 0.28 | 1.96 | 0.56 | 0.18 | 1.15 | 0.14 | 0.24 | 0.14 | 0.15 | 0.22 | 0.37 | LOC100156421 |
| gene16147 | -1.218453099 | 0.005849689 | 0.26 | 0.19 | 0.43 | 0.36 | 0.24 | 1.42 | 0.35 | 0.32 | 0.11 | 0.26 | 0.15 | 0.4 | DNAI2 |
| gene16160 | -1.193739295 | 0.001708907 | 22.76 | 16.15 | 18.4 | 26.93 | 26.21 | 23.99 | 12.4 | 11.19 | 8.95 | 3.07 | 20.17 | 47.63 | SOX9 |
| gene16166 | -2.684770828 | 0.000814644 |  |  |  |  |  |  |  |  |  |  |  |  |  |
| gene16173 | -2.122211356 | 0.006825417 | 0.15 | 0 | 0.15 | 0.39 | 0.3 | 0.53 | 0 | 0.08 | 0.26 | 0.12 | 0.29 | 0.21 | LOC100521038 |
| gene16183 | -1.346821615 | 0.001233388 | 1.14 | 0.89 | 3.18 | 8.74 | 1.44 | 6.03 | 4.78 | 0.66 | 0 | 1.57 | 0.33 | 0.3 | LOC100522637 |
| gene16187 | -4.034428593 | 1.41E-09 | 0 | 0.04 | 0.05 | 0.81 | 0.34 | 2 | 0.03 | 0.15 | 0 | 3.85 | 0.06 | 0.05 | LOC100522100 |
| gene16235 | -1.447896885 | 0.006786543 | 0.5 | 0.38 | 1.1 | 0.81 | 1.96 | 1.54 | 0.39 | 0.79 | 0.44 | 1.4 | 0.2 | 0.09 | LOC100516998 |
| gene16263 | 1.037319598 | 0.006024679 | 29.55 | 30.82 | 27.79 | 20.44 | 25.26 | 16.48 | 37.04 | 40.75 | 45.81 | 27.34 | 44.38 | 47.53 | PLCD3 |
| gene16277 | -1.982979594 | 7.26E-05 | 0.59 | 0.57 | 1.09 | 1.95 | 1.13 | 1.13 | 0.37 | 0.45 | 0.14 | 0.45 | 0.93 | 0.27 | FZD2 |
| gene16316 | -1.513527801 | 0.000944593 | 1.61 | 1.33 | 1.39 | 2.44 | 6.63 | 1.87 | 1.9 | 1.19 | 0.5 | 3.39 | 1.03 | 2.89 | LOC100624863 |
| gene16364 | 2.017009265 | 1.17E-05 |  |  |  |  |  |  |  |  |  |  |  |  |  |
| gene16366 | 3.17989872 | 0.000361124 | 0 | 0.07 | 0.04 | 0.03 | 0.03 | 0 | 0.18 | 0.15 | 0.44 | 0.36 | 0.44 | 0.04 | LOC100737030 |
| gene16367 | 1.569668308 | 0.000161772 | 1.1 | 1.26 | 0.52 | 1.36 | 1.49 | 0.12 | 6.67 | 0.71 | 1.18 | 2.96 | 0.75 | 2.01 | LOC100737072 |
| gene16408 | 3.424700251 | 7.45E-05 | 0.09 | 0 | 0.12 | 0 | 0.03 | 0.04 | 0.38 | 0.24 | 0.31 | 0.14 | 0.32 | 0 | LOC100520330 |
| gene16440 | -1.395234916 | 0.007338552 | 0.25 | 0.73 | 0.33 | 0.47 | 0.8 | 1.36 | 0.56 | 0.16 | 0.09 | 0 | 0.24 | 0.14 | ZPBP2 |
| gene16470 | -1.009336648 | 0.008551712 | 49.78 | 31.56 | 23 | 30.73 | 82.77 | 30.28 | 35.24 | 15.2 | 19.13 | 19.78 | 15.12 | 30 | LOC100626612 |
| gene16489 | 3.085514918 | 0.007364993 | 0.1 | 0 | 0.14 | 0.12 | 0 | 0 | 0.17 | 0.84 | 0.55 | 0.37 | 0.49 | 0 | LOC100627478 |
| gene16494 | -1.128984592 | 0.004222835 | 7.05 | 6.95 | 4.97 | 5.28 | 9.27 | 8.31 | 4.1 | 3.71 | 2.13 | 3.34 | 2.66 | 4.69 | LOC100522700 |
| gene16500 | -1.298624364 | 0.000791453 | 71.37 | 66.75 | 41.57 | 57.49 | 67.19 | 35.03 | 13.25 | 43.92 | 5.39 | 20.06 | 29.66 | 3.97 | LOC100739319 |
| gene16529 | -1.360687885 | 0.000343988 | 69.21 | 55.06 | 67.57 | 81.49 | 91.47 | 113.48 | 44.92 | 34.71 | 27.76 | 88.1 | 45.71 | 42.24 | COL1A1 |
| gene16530 | -1.381481167 | 0.000274452 | 127.06 | 103.67 | 124.42 | 160.33 | 199.98 | 243.69 | 84.15 | 78.49 | 60.85 | 178.19 | 101.89 | 124.39 | LOC100738213 |
| gene16547 | -5.668363331 | 5.52E-05 | 0 | 0 | 0 | 0 | 0.41 | 0 | 0 | 0 | 0 | 0 | 0 | 0 | LOC100737860 |
| gene1657 | 1.000198624 | 0.008446209 | 1.62 | 3.16 | 2.78 | 3.79 | 3.91 | 2.39 | 6.32 | 2.37 | 10.85 | 2.89 | 3.71 | 6.85 | FRMPD1 |
| gene16670 | 2.484113059 | 0.000423681 |  |  |  |  |  |  |  |  |  |  |  |  |  |
| gene16712 | -1.664701283 | 0.008487894 | 0.21 | 0.09 | 0.31 | 0.11 | 0.11 | 0.2 | 0.03 | 0.07 | 0 | 0.11 | 0 | 0.04 | SLFN14 |
| gene16713 | -1.661906249 | 0.001011077 | 0.52 | 0.5 | 0.51 | 0.73 | 0.66 | 0.41 | 0.16 | 0.25 | 0.14 | 0.2 | 0.25 | 0.06 | SLFN11 |
| gene16727 | 1.671467193 | 1.99E-05 | 13.26 | 37.28 | 14.78 | 23.15 | 8.15 | 27.9 | 35.64 | 75.18 | 68.46 | 19.93 | 46.82 | 30.83 | CCL2 |
| gene16730 | 1.518907334 | 0.002598467 | 0.44 | 0.29 | 0.52 | 0.67 | 0.3 | 0.32 | 2.44 | 0.6 | 0.38 | 0.29 | 0.96 | 0.31 | LOC100628008 |
| gene16812 | -1.589135992 | 0.009384431 | 0.1 | 0.22 | 0.33 | 0.47 | 0.2 | 0.23 | 0.07 | 0.06 | 0.18 | 0.15 | 0.31 | 0.14 | ANKRD13B |
| gene16813 | -1.280759293 | 0.003559839 | 0.93 | 0.79 | 0.83 | 3.61 | 1.06 | 2.16 | 1.11 | 0.83 | 0.73 | 0.68 | 1.21 | 2.45 | LOC100516758 |
| gene16859 | 4.978380969 | 0.001226127 |  |  |  |  |  |  |  |  |  |  |  |  |  |
| gene16867 | 5.295683892 | 0.000420481 |  |  |  |  |  |  |  |  |  |  |  |  |  |
| gene16925 | -1.683618788 | 0.002815433 | 0.77 | 0.3 | 0.72 | 1.03 | 0.76 | 1.7 | 0.27 | 0.35 | 0.39 | 0.98 | 0.96 | 0.47 | LOC100521819 |
| gene16980 | 1.248175362 | 0.005341854 | 4.54 | 3.73 | 2.85 | 2.77 | 2.1 | 1.86 | 5.08 | 3.12 | 7.68 | 2.09 | 1.69 | 4.75 | GABARAP |
| gene16989 | 1.203887643 | 0.001579297 | 32.53 | 41.47 | 36.08 | 25.05 | 28.03 | 18.08 | 32.78 | 49.61 | 76.39 | 33.04 | 50.81 | 75.24 | LOC100622059 |
| gene17006 | -3.174427024 | 0.00027911 | 0.09 | 0.45 | 0 | 0.15 | 0.57 | 0.32 | 0.05 | 0.05 | 0 | 0.57 | 0 | 0.25 | LOC100522212 |
| gene17034 | 1.681485471 | 4.12E-05 | 0.15 | 1.18 | 0.65 | 1.16 | 0.4 | 0.78 | 2.05 | 3.55 | 1.56 | 1.14 | 7.04 | 5.05 | ALOXE3 |
| gene1706 | -1.537810196 | 0.001116647 | 0.93 | 2.01 | 0.86 | 1.62 | 1.86 | 1.66 | 0.68 | 0.41 | 0.61 | 0.67 | 0.24 | 0.88 | LOC100154648 |
| gene1707 | -1.45658773 | 0.001180571 | 0.06 | 0.46 | 0.65 | 0.07 | 1.75 | 1.33 | 0.51 | 0.5 | 0 | 0.49 | 0.63 | 0.28 | LOC100515185 |
| gene17116 | -2.235493725 | 2.61E-08 | 3.93 | 5.09 | 5.14 | 9.05 | 2.51 | 11.45 | 1.49 | 2.47 | 0.65 | 0.49 | 3.03 | 0.25 | ALDH3A1 |
| gene17120 | -1.49788595 | 0.003561512 | 1.22 | 0.59 | 0.86 | 0.98 | 1.66 | 2.06 | 0.78 | 0.34 | 0.45 | 0.21 | 0.2 | 0.37 | LOC100621187 |
| gene17153 | 1.731240398 | 5.94E-05 | 0.23 | 1.12 | 0.56 | 0.82 | 0.5 | 0.36 | 1.49 | 2.05 | 1.73 | 1.68 | 1.96 | 1 | LOC100736917 |
| gene17159 | 1.857306769 | 0.003303642 | 0.96 | 0.28 | 0.49 | 0.2 | 0.61 | 0.53 | 0.37 | 2.69 | 1.26 | 0.6 | 1.44 | 0.52 | LOC100625499 |
| gene17160 | 1.039727762 | 0.005894433 | 32.13 | 18.1 | 20.37 | 41.28 | 24.79 | 22.43 | 51.34 | 83.8 | 40.6 | 38.01 | 152.74 | 58.92 | GALNTL2 |
| gene17191 | -1.521149674 | 7.12E-05 | 53.97 | 76.73 | 71.2 | 98.76 | 72.4 | 136.85 | 33.13 | 43.59 | 26.24 | 50.09 | 35.11 | 22.86 | LOC100620195 |
| gene17194 | 1.195157326 | 0.001645062 | 86.51 | 59.93 | 33.66 | 38.16 | 72.93 | 27.41 | 133.37 | 95.32 | 80.21 | 98.54 | 77.48 | 67.43 | LOC100620439 |
| gene17198 | 4.686761086 | 0.00659106 |  |  |  |  |  |  |  |  |  |  |  |  |  |
| gene17266 | 1.777409812 | 3.60E-05 | 0.2 | 0.24 | 0.17 | 0.09 | 0.19 | 0.28 | 0.62 | 0.9 | 0.28 | 1.14 | 0.72 | 0.46 | LOC100152567 |
| gene17275 | 1.322869226 | 0.006243924 | 0.16 | 0.21 | 0.1 | 0.12 | 0.09 | 0.1 | 0.3 | 0.18 | 0.24 | 0.23 | 0.59 | 0.84 | XIRP1 |
| gene17322 | 1.650782158 | 0.002815433 | 1.13 | 0.69 | 2.05 | 1.52 | 0.49 | 0.62 | 0.8 | 5.51 | 2.23 | 2.88 | 0.9 | 1.81 | LOC100526248 |
| gene17334 | -1.122431099 | 0.007735094 | 1.72 | 2.98 | 2.6 | 2.85 | 2.19 | 4.89 | 1.71 | 1.46 | 1.15 | 2.32 | 0.77 | 1.61 | LOC100627100 |
| gene17364 | -1.078449316 | 0.006928889 | 0.78 | 1.16 | 1.69 | 0.71 | 1.92 | 3.12 | 1.4 | 0.79 | 0.35 | 0.75 | 0.45 | 0.11 | LTF |
| gene17370 | 1.473176181 | 0.009151741 |  |  |  |  |  |  |  |  |  |  |  |  |  |
| gene17377 | -1.751324128 | 0.0075192 | 0.64 | 0.74 | 1.02 | 0.97 | 1.07 | 1.47 | 0.1 | 0.27 | 0.78 | 0.31 | 0.5 | 0.91 | LOC100515755 |
| gene17417 | -1.416018735 | 0.002662627 | 0.51 | 1.02 | 1.07 | 1.09 | 1.52 | 3.34 | 0.35 | 0.96 | 0.98 | 0.78 | 0.13 | 0.69 | LOC100155669 |
| gene17437 | -1.2737853 | 0.001438821 | 3.3 | 2.55 | 3.19 | 2.92 | 5.8 | 5.79 | 2.4 | 2.2 | 1.15 | 1.85 | 2.37 | 1.25 | LOC100510980 |
| gene17453 | -1.480746679 | 0.002495841 | 0.03 | 0.22 | 0.28 | 0.43 | 0.44 | 0.48 | 0.2 | 0.15 | 0.08 | 0.47 | 0.08 | 0.18 | CDHR4 |
| gene175 | 1.794302745 | 0.001143928 |  |  |  |  |  |  |  |  |  |  |  |  |  |
| gene1753 | 3.733310239 | 4.90E-07 | 0.76 | 0.15 | 0 | 0 | 0.87 | 0 | 3.05 | 8.21 | 1.77 | 3.7 | 4.38 | 1.81 | LOC100736986 |
| gene17532 | -1.819773474 | 0.000457244 | 0.09 | 0.19 | 0.12 | 0.23 | 0.16 | 0.8 | 0.16 | 0.05 | 0.08 | 0.23 | 0.13 | 0.13 | ITIH1 |
| gene17534 | -1.748208382 | 0.002148629 | 0.25 | 0.3 | 0.35 | 3.65 | 0.69 | 1.41 | 0.82 | 0.51 | 0 | 3.66 | 0 | 0.7 | LOC100737917 |
| gene17536 | -1.467096319 | 0.001974927 | 0.21 | 0.28 | 0.28 | 0.67 | 0.4 | 0.86 | 0.23 | 0.27 | 0.15 | 1.68 | 0.27 | 0.25 | ITIH4 |
| gene17550 | -1.667897192 | 0.000743253 | 0.6 | 1.37 | 3.41 | 1.93 | 3.1 | 2.4 | 1.46 | 0.33 | 0.32 | 0.74 | 0.84 | 0.35 | LOC100737042 |
| gene17551 | -2.62978921 | 1.56E-09 | 0.54 | 0.67 | 1.72 | 2.41 | 5.42 | 2.25 | 0.51 | 0.56 | 0.54 | 0.53 | 0.69 | 0.34 | CACNA2D3 |
| gene17596 | -1.501861857 | 0.002177642 | 0.67 | 0.88 | 0.57 | 0.83 | 1.4 | 3.77 | 0.87 | 0.89 | 0 | 0.64 | 0.85 | 0.61 | LOC100519514 |
| gene17637 | 1.577207597 | 0.000206896 | 0.48 | 0.37 | 0.42 | 0.41 | 0.24 | 0.42 | 1.47 | 0.94 | 0.58 | 1.78 | 0.41 | 0.98 | CHL1 |
| gene17664 | 1.668593757 | 3.31E-05 | 11.28 | 18.7 | 8.86 | 1.36 | 1.58 | 7.4 | 1.49 | 18.36 | 11.07 | 21.41 | 6.81 | 16.68 | LOC100621522 |
| gene17695 | 3.2780593 | 7.99E-16 | 100.9 | 148.19 | 59.71 | 120.81 | 99.84 | 95.55 | 1067.5 | 1137.4 | 754.84 | 989.42 | 956.39 | 749.17 | LOC100739306 |
| gene17719 | -1.4389085 | 0.000707342 | 0.39 | 0.4 | 0.28 | 0.8 | 0.58 | 0.62 | 0.41 | 0.16 | 0.11 | 0.49 | 0.38 | 0.38 | CAND2 |
| gene17744 | -1.419040192 | 0.000239626 | 4.88 | 5.11 | 7.28 | 7.11 | 8.26 | 11.68 | 5.25 | 2.5 | 1.94 | 4.69 | 4.05 | 3.25 | LOC100621722 |
| gene17746 | -1.390876284 | 0.000319971 | 7.41 | 7.14 | 13.52 | 13.38 | 15.28 | 21.3 | 8.02 | 3.54 | 6.78 | 7.33 | 5.91 | 6.61 | LOC100738595 |
| gene17781 | -1.333842726 | 0.002091337 | 2.26 | 2.06 | 2.24 | 2.67 | 1.81 | 3.21 | 0.91 | 0.96 | 1.16 | 1.87 | 1.1 | 1.78 | LOC100519444 |
| gene17792 | 1.526864318 | 0.000606715 | 0.36 | 0.36 | 0.22 | 0.36 | 0.49 | 1.09 | 2.12 | 1.91 | 1.03 | 2.05 | 0.93 | 0.48 | LOC100522105 |
| gene17800 | -2.84504403 | 3.42E-10 | 0.99 | 0.85 | 0.56 | 1 | 2.82 | 3.5 | 0.16 | 0.37 | 0.52 | 7.7 | 0.53 | 0.2 | TF |
| gene17812 | 1.921951495 | 0.005122371 | 0.11 | 0.12 | 0.02 | 0.04 | 0.07 | 0.02 | 0.15 | 0.18 | 0.21 | 0.13 | 0.27 | 0.16 | LOC100524585 |
| gene17827 | 2.544812589 | 0.000309487 | 0.13 | 0.12 | 0 | 0 | 0.18 | 0.05 | 0.15 | 0.7 | 0.39 | 0.26 | 0.05 | 0.32 |  |
| gene17833 | -1.141566049 | 0.002854572 |  |  |  |  |  |  |  |  |  |  |  |  |  |
| gene17854 | 1.318688408 | 0.004579271 |  |  |  |  |  |  |  |  |  |  |  |  |  |
| gene17875 | -1.24313609 | 0.001075573 | 12.94 | 4.96 | 16.48 | 20.08 | 53.03 | 17.4 | 20.12 | 8.74 | 8.43 | 6.33 | 9.91 | 11.48 | LOC100626514 |
| gene17896 | -1.587194907 | 3.19E-05 | 118.41 | 100.53 | 225.94 | 176.28 | 141.84 | 676.56 | 264.51 | 19.78 | 30.07 | 41.27 | 121.55 | 24.19 | CP |
| gene1794 | 1.016721422 | 0.008309146 | 7.32 | 10.93 | 6.57 | 3.01 | 2.99 | 2.82 | 3.11 | 5.75 | 8.42 | 10.88 | 6.06 | 8.57 | SUSD1 |
| gene17955 | -1.078353357 | 0.004474213 | 8.3 | 11.46 | 19.23 | 31.41 | 15.97 | 28.24 | 5.07 | 15.78 | 13.59 | 13.86 | 13.43 | 7.71 | VEPH1 |
| gene17959 | -1.439555057 | 0.000576599 | 4.34 | 4.57 | 6.94 | 4.41 | 3.77 | 3.84 | 1.28 | 1.63 | 1.44 | 3.06 | 2.62 | 2.8 | SHOX2 |
| gene17988 | 2.44254899 | 2.16E-06 | 0.54 | 0.05 | 0.18 | 0.22 | 0 | 0.54 | 0.98 | 1.72 | 0.78 | 0.2 | 1.81 | 0 | OTOL1 |
| gene17991 | -2.046431211 | 0.000160954 | 3.49 | 3.82 | 7.53 | 18.6 | 4.73 | 8.45 | 1.09 | 2.91 | 4.53 | 3.74 | 3.11 | 1.21 | BST2 |
| gene18004 | -1.023647909 | 0.008062374 | 26.97 | 39.24 | 38.5 | 11.31 | 11.28 | 16.38 | 5.45 | 4.56 | 8.5 | 10.55 | 15.02 | 13.1 | LOC100154352 |
| gene18016 | 2.355732575 | 0.000108163 |  |  |  |  |  |  |  |  |  |  |  |  |  |
| gene1803 | -1.083431529 | 0.008858945 | 5.23 | 5.04 | 11.39 | 10.61 | 10.23 | 13.57 | 0.85 | 10.82 | 4.07 | 5.82 | 4.67 | 5.11 | LOC100516718 |
| gene18035 | -1.206043163 | 0.001459987 | 113.01 | 49.4 | 177.84 | 121.15 | 259.27 | 132.5 | 92.44 | 61.81 | 61.91 | 80.41 | 54.4 | 42.04 | TNFSF10 |
| gene18036 | -1.003811895 | 0.008444363 | 14.49 | 6.1 | 22.14 | 22.55 | 40.88 | 18.53 | 17.75 | 12.92 | 9 | 10.04 | 7.65 | 5.87 | GHSR |
| gene18052 | 1.498992534 | 0.001039821 | 0.75 | 0.85 | 0.57 | 1.13 | 0.92 | 1.23 | 1.16 | 5.48 | 1.87 | 4.26 | 2.02 | 0.83 | KCNMB2 |
| gene18073 | 2.02813527 | 0.000194542 | 5.82 | 4.97 | 5.53 | 0.52 | 0 | 0.21 | 0 | 2.17 | 0.66 | 1.07 | 2.16 | 3.97 | SOX2 |
| gene18083 | 1.315096444 | 0.00055607 | 41.77 | 16.75 | 14.16 | 13.27 | 9.75 | 14.68 | 22.76 | 43.64 | 23.84 | 26.75 | 54.46 | 32.74 | LOC100624866 |
| gene18105 | -1.943618271 | 0.000163749 | 0.11 | 0.13 | 0.04 | 0.14 | 0.47 | 0.1 | 0.08 | 0.08 | 0 | 0 | 0.05 | 0.12 | VWA5B2 |
| gene1812 | 2.203212171 | 1.48E-06 | 1.48 | 1.03 | 1.56 | 0.89 | 0.41 | 1.53 | 2.67 | 6.61 | 2.47 | 3.99 | 3.8 | 1.76 | LOC100622036 |
| gene18195 | -1.159041348 | 0.007148502 | 1.08 | 1.81 | 2.18 | 1.46 | 2.41 | 2.86 | 1.36 | 1.28 | 0 | 1.41 | 0.98 | 0.89 | LOC100511473 |
| gene18209 | 1.131971346 | 0.0087753 | 1.31 | 1.05 | 0.98 | 1.67 | 0.81 | 0.64 | 2.35 | 2.71 | 1.42 | 3.97 | 2.43 | 2.27 | LOC100512372 |
| gene18276 | 1.021146072 | 0.00887969 | 1.02 | 0.93 | 0.74 | 1.94 | 1.18 | 1.43 | 0.61 | 5.59 | 2.7 | 2.28 | 1.83 | 2.19 | STXBP5L |
| gene18280 | -1.041903625 | 0.006730917 | 1.76 | 2.67 | 5.39 | 4.89 | 0.91 | 10.24 | 3.16 | 2.59 | 1.6 | 1.46 | 3.99 | 2.6 | GPR156 |
| gene18281 | -1.688216405 | 4.05E-05 | 3.4 | 4.49 | 6.27 | 6.25 | 0.55 | 18.9 | 4.23 | 2.11 | 0.85 | 3.25 | 3.43 | 2.2 | LOC100523211 |
| gene18323 | -1.154263879 | 0.002706998 | 3.6 | 7.02 | 13.09 | 9.14 | 5.13 | 7.74 | 2.83 | 2.42 | 4.27 | 5.17 | 2.29 | 4.44 | BOC |
| gene18325 | -2.043664868 | 0.008487894 | 0.55 | 0.66 | 1.47 | 0.9 | 0.18 | 1.37 | 0.27 | 0 | 0.32 | 0.34 | 0 | 0.34 | LOC100739776 |
| gene18329 | -1.428427205 | 0.004146109 | 0.28 | 0 | 0.67 | 2.46 | 0.32 | 0.91 | 0.61 | 0.43 | 0.22 | 0.07 | 0.07 | 0.37 | CD200R1L |
| gene18339 | 1.109750275 | 0.00340434 | 29.32 | 22.05 | 28.14 | 33.04 | 41.16 | 25.5 | 44.95 | 90.49 | 73.39 | 49.51 | 43.63 | 78.66 | LOC100626182 |
| gene18381 | -1.879951936 | 1.59E-06 | 1.19 | 3.53 | 2.98 | 9 | 6.81 | 8.93 | 3.32 | 1.54 | 1.58 | 3.9 | 1.94 | 4.05 | ABI3BP |
| gene18382 | -1.373601099 | 0.000437715 | 6.9 | 12.76 | 11.08 | 33.01 | 20.4 | 31.01 | 11.74 | 7.86 | 11.77 | 12.29 | 8.75 | 10.63 | LOC100624376 |
| gene1845 | -2.64243704 | 7.83E-05 | 0.49 | 1.1 | 0.74 | 1.18 | 0.5 | 1.63 | 0.15 | 0.13 | 0.22 | 0.51 | 0.43 | 0.45 | LOC100523351 |
| gene1846 | 1.384961481 | 0.000507953 | 0.45 | 0.48 | 0.46 | 0.76 | 0.63 | 1.17 | 2.17 | 2.56 | 1.68 | 2.14 | 1.87 | 1.19 | ASTN2 |
| gene18474 | 2.88151009 | 8.91E-07 | 0.24 | 0.14 | 0.11 | 0.1 | 0.04 | 0.05 | 0.54 | 0.69 | 0.15 | 0.42 | 0.3 | 0.32 | EPHA3 |
| gene18480 | 2.849520799 | 0.000404617 | 0.39 | 0.16 | 0.26 | 0 | 0.1 | 0.06 | 0.41 | 0.36 | 0.53 | 0.7 | 0.61 | 0.12 | POU1F1 |
| gene18514 | -1.536804627 | 0.002903134 | 0.88 | 0.42 | 1.04 | 1.3 | 0.94 | 1.67 | 0.37 | 0.53 | 0.32 | 0.45 | 0.43 | 0.86 | LOC100520642 |
| gene18540 | -1.504563383 | 8.63E-05 | 5.76 | 9.96 | 13.77 | 26.84 | 21.75 | 17.57 | 9.44 | 2.87 | 10.19 | 5.71 | 4.41 | 10.31 | ADAMTS5 |
| gene18542 | -1.095921417 | 0.007058484 | 3.61 | 16.62 | 15.19 | 16.11 | 17.47 | 9.04 | 9.77 | 2.9 | 6.53 | 13.9 | 6.26 | 2.76 | LOC100738525 |
| gene18557 | -3.389754539 | 5.71E-09 | 0.27 | 0.37 | 1.19 | 0.65 | 3.15 | 2.8 | 0.33 | 0.14 | 0 | 0 | 0.22 | 0 | LOC100624997 |
| gene18559 | -2.667948015 | 0.002810632 |  |  |  |  |  |  |  |  |  |  |  |  |  |
| gene18602 | -1.226253827 | 0.005614941 | 1.84 | 0.95 | 1.42 | 4.54 | 6.91 | 1.95 | 2.98 | 0.81 | 1.53 | 1.74 | 2.18 | 1.62 | LOC100621502 |
| gene18612 | -1.028514609 | 0.006366055 | 237.37 | 183.83 | 188.49 | 211.19 | 315.83 | 223.93 | 132.58 | 112.9 | 111.14 | 185.84 | 84.64 | 111.75 | LOC100622246 |
| gene18628 | 2.222189086 | 0.000109772 | 0.15 | 0.25 | 0.3 | 0.31 | 0.09 | 0.16 | 0.7 | 1.03 | 0.79 | 0.31 | 0.3 | 0.11 | LOC100515928 |
| gene18641 | -2.246804867 | 8.98E-09 | 77.97 | 25.55 | 70.89 | 62.18 | 84.31 | 84.83 | 31.3 | 5.21 | 10.37 | 23.29 | 5.64 | 5.4 | LOC100517006 |
| gene18648 | -1.096181675 | 0.003819739 | 15 | 8.41 | 6.17 | 56.05 | 11.81 | 31.59 | 11.77 | 18.95 | 13.86 | 25.9 | 13.78 | 14.81 | MX1 |
| gene18653 | 4.696569895 | 0.00659106 | 0.2 | 0.24 | 0 | 0 | 0 | 0 | 0.26 | 0.28 | 0.12 | 0 | 0.1 | 0.09 | LOC100518095 |
| gene18663 | -2.79456187 | 4.43E-07 | 0.99 | 1.86 | 5.66 | 1.07 | 8.71 | 2.64 | 1.35 | 0 | 0 | 0 | 0.72 | 0 | TFF1 |
| gene18664 | -4.010948409 | 1.62E-16 | 0.77 | 0.07 | 1 | 1.37 | 1.74 | 6.4 | 0.45 | 0 | 0 | 0 | 0.44 | 0.04 | LOC100627826 |
| gene18669 | -1.094608984 | 0.004018934 | 20.64 | 26.07 | 24.13 | 32.47 | 33.41 | 30.17 | 15.4 | 10.74 | 17.3 | 10.94 | 11.75 | 19.07 | LOC100620809 |
| gene18712 | 1.431491331 | 0.007198721 | 0.47 | 0.93 | 0.29 | 0.21 | 0.46 | 0.37 | 0.93 | 0.99 | 0.7 | 1.06 | 0.34 | 0.83 | LOC100624867 |
| gene18722 | 2.475291487 | 1.71E-05 | 0.27 | 0.2 | 0.39 | 0.06 | 0.09 | 0.23 | 0.11 | 1.24 | 0.8 | 1.45 | 2.09 | 2.01 | LOC100520406 |
| gene1877 | -1.237543214 | 0.002899854 | 3.16 | 7.91 | 12.99 | 16.96 | 13.86 | 6.95 | 8.71 | 1.58 | 5 | 10.76 | 4.74 | 5.17 | LOC100620387 |
| gene18776 | 1.881722937 | 0.002275342 | 1.68 | 1.52 | 0.72 | 0.17 | 0.08 | 0.31 | 0.46 | 0.57 | 0.88 | 1.17 | 0.57 | 1.79 | EGR3 |
| gene18805 | 2.017217231 | 2.35E-06 | 0.04 | 0.19 | 0.3 | 0.61 | 0.33 | 0.43 | 0.79 | 2.44 | 2.01 | 0.24 | 2.2 | 1.88 | LOC100625407 |
| gene18826 | 2.321039914 | 2.70E-05 | 0.32 | 0.78 | 0.08 | 0.27 | 0.07 | 0.14 | 1.4 | 0.63 | 0.15 | 0.56 | 0.36 | 0.45 | GULO |
| gene18833 | -1.15079458 | 0.002423212 | 19.06 | 11.42 | 14.47 | 14.06 | 46.71 | 31.51 | 20.67 | 10.66 | 8.97 | 18.22 | 29.34 | 28.56 | SCARA5 |
| gene18838 | 2.161751341 | 4.52E-08 | 4.68 | 7.07 | 6.8 | 6.38 | 10.14 | 6.9 | 39.85 | 36.01 | 25.42 | 33.45 | 25.71 | 21.88 | LOC100154295 |
| gene18847 | 1.944767213 | 0.000188811 | 0.6 | 0.63 | 0.56 | 0.74 | 0.13 | 0.91 | 1.68 | 2.61 | 1.83 | 1.89 | 0.68 | 0.37 |  |
| gene18857 | 5.114241639 | 0.000714555 | 0.03 | 0 | 0 | 0 | 0 | 0 | 0.11 | 0.11 | 0.22 | 0.04 | 0.08 | 0 | BLK |
| gene18871 | -1.098355921 | 0.003592007 | 683.87 | 323.31 | 497.17 | 516.98 | 1684.6 | 951.09 | 731.44 | 244.9 | 454.48 | 362.81 | 340.74 | 488.3 | HPGD |
| gene18897 | -1.758692983 | 0.000107453 | 1.11 | 0.07 | 0.36 | 0.32 | 5.28 | 0.34 | 1.06 | 0.2 | 0.41 | 0.25 | 0.12 | 1.19 | LOC100627945 |
| gene18910 | -1.875579983 | 2.71E-05 | 2.78 | 2.16 | 0.87 | 1.33 | 3.08 | 1.42 | 1.1 | 0.04 | 0.26 | 0.77 | 0.23 | 0.49 | LOC100736581 |
| gene18911 | -1.374972428 | 0.002069121 | 0.81 | 1.1 | 1.56 | 2.28 | 0.91 | 2.22 | 1.03 | 0.2 | 0.74 | 2.31 | 1.04 | 1.91 | GALNT9 |
| gene18923 | -1.156092409 | 0.00543144 | 0.96 | 0.78 | 1.04 | 1.94 | 1.85 | 1.94 | 0.99 | 0.72 | 0.74 | 1.19 | 0.88 | 1.2 | LOC100157489 |
| gene18938 | 1.410143992 | 0.007109732 | 0.18 | 0.14 | 0.16 | 0.24 | 0.05 | 0.03 | 0.12 | 0.6 | 0.1 | 0.31 | 0.36 | 0.19 | TMEM132B |
| gene18994 | -1.722378825 | 7.02E-05 | 1.52 | 0.97 | 0.62 | 2.5 | 0.54 | 3.04 | 1.18 | 0.34 | 0.06 | 0.59 | 0.49 | 0.15 | HPD |
| gene19041 | 2.070893814 | 0.000780555 | 0.19 | 0.87 | 0.17 | 0.4 | 0.22 | 0.09 | 0.58 | 1.02 | 1.58 | 0.84 | 1.12 | 1.67 | LOC100156825 |
| gene19048 | -1.089811695 | 0.007456958 | 1.4 | 2.45 | 1.65 | 0.98 | 3.42 | 2.83 | 0.59 | 1.17 | 1.61 | 1.01 | 1.36 | 2.3 | KSR2 |
| gene19064 | -1.602846267 | 6.14E-05 | 3.34 | 4.15 | 6.37 | 5.05 | 9.63 | 5.94 | 4.31 | 0.71 | 1.45 | 1.04 | 1.78 | 1.4 | LOC100154365 |
| gene19072 | -1.229964816 | 0.001240067 | 6.82 | 11.93 | 16.63 | 42.91 | 6.64 | 25.06 | 11.11 | 11.38 | 7.91 | 18.31 | 8.71 | 6.96 | OAS2 |
| gene19115 | -1.601449855 | 0.002145956 | 0.18 | 0.33 | 0.1 | 0.13 | 0.33 | 2.72 | 0.23 | 0.45 | 0.32 | 0.41 | 0.17 | 0.51 | OASL |
| gene19153 | -2.317315364 | 0.002899788 | 0.6 | 0.54 | 0.93 | 0.18 | 0.21 | 1.55 | 0.1 | 0.12 | 0.1 | 1.45 | 0.19 | 1.53 | LOC100157434 |
| gene19157 | 2.075059102 | 0.000758033 | 0.12 | 0.14 | 0.23 | 0.03 | 0.04 | 0.12 | 0.34 | 0.27 | 0.06 | 0.14 | 0.2 | 0.05 | SEZ6L |
| gene19180 | 2.44168633 | 5.89E-06 | 0.1 | 0.18 | 0.08 | 0.08 | 0.03 | 0.1 | 0.08 | 0.11 | 1.07 | 0.14 | 0.09 | 0.15 | NEFH |
| gene19191 | 2.40145758 | 2.57E-09 | 5.84 | 10.67 | 23.8 | 4.09 | 3.02 | 6 | 1.75 | 50.52 | 13.96 | 24.48 | 18.07 | 20.79 | LOC100154446 |
| gene19206 | -1.133603338 | 0.004057875 | 4.03 | 3.1 | 4.62 | 3.43 | 4.38 | 7.03 | 2.49 | 2.46 | 1.51 | 3.04 | 3.1 | 3.21 | TCN2 |
| gene19209 | 1.058806096 | 0.00592912 | 2.77 | 2.82 | 3.23 | 2.29 | 2.2 | 1.77 | 3.86 | 3.84 | 4.92 | 4.06 | 3.3 | 5.34 | LOC100515970 |
| gene19243 | -1.727362184 | 0.000339282 | 2.81 | 5.15 | 2.44 | 5.45 | 1.99 | 1.4 | 1.82 | 0.32 | 0.19 | 0 | 0.34 | 0 | LOC100152327 |
| gene19286 | -1.732851402 | 9.05E-05 | 3.04 | 3.1 | 1.19 | 4.03 | 2.25 | 2.31 | 0.66 | 1.13 | 0.68 | 0.38 | 0.8 | 0.17 | YDJC |
| gene19324 | -1.348279176 | 0.000926941 | 1.72 | 2.39 | 2.1 | 2.76 | 7.33 | 7.37 | 3.67 | 1.62 | 1.19 | 1.14 | 0.74 | 2.17 | CLDN5 |
| gene19363 | 1.603527626 | 0.000154861 |  |  |  |  |  |  |  |  |  |  |  |  |  |
| gene19391 | -2.416238684 | 0.000402199 | 0.03 | 0.11 | 0.24 | 0.17 | 0.46 | 0.07 | 0 | 0.08 | 0.06 | 0.05 | 0 | 0.02 | LOC100153049 |
| gene19422 | -1.155809186 | 0.005073048 | 4.81 | 2.53 | 3.96 | 3.62 | 4.52 | 4.28 | 2.33 | 1.54 | 1.46 | 2.58 | 1.41 | 1.18 |  |
[truncated: 158,304 more chars]
